# Efficient and versatile multiplex prime editing in hexaploid wheat

**DOI:** 10.1186/s13059-023-02990-1

**Published:** 2023-06-29

**Authors:** Pei Ni, Yidi Zhao, Ximeng Zhou, Zehua Liu, Zhengwei Huang, Zhongfu Ni, Qixin Sun, Yuan Zong

**Affiliations:** Frontiers Science Center for Molecular Design Breeding (MOE), Key Laboratory of Crop Heterosis and Utilization (MOE), and Beijing Key Laboratory of Crop Genetic Improvement, China Agricultural University, Beijing, 100193 China

**Keywords:** Hexaploid wheat, Prime editing, Reverse transcriptase, Multiplex genome editing

## Abstract

Prime editing is limited by low efficiency in plants. Here, we develop an upgraded engineered plant prime editor in hexaploid wheat, ePPEplus, by introducing a V223A substitution into reverse transcriptase in the ePPEmax* architecture. ePPEplus enhances the efficiency by an average 33.0-fold and 6.4-fold compared to the original PPE and ePPE, respectively. Importantly, a robust multiplex prime editing platform is established for simultaneous editing of four to ten genes in protoplasts and up to eight genes in regenerated wheat plants at frequencies up to 74.5%, thus expanding the applicability of prime editors for stacking of multiple agronomic traits.

**Supplementary Information:**

The online version contains supplementary material available at 10.1186/s13059-023-02990-1.

## Background

Common wheat (*Triticum aestivum*, AABBDD, 2*n* = 6*x* = 42) is an allohexaploid species, comprising A, B, and D subgenomes. As a major staple crop worldwide, common wheat provides > 30% of dietary calories used by humans [1–3]. Maintaining and increasing wheat production in the face of climate change and the limited availability of arable land is thus a crucial challenge. However, the allohexaploidy and functional gene redundancy of wheat make it a daunting task to induce any mutation efficiently and precisely across gene homoeologs and/or in the multiple genes that may need to be altered to effect the enhancement and pyramiding of important agronomic traits. Genome editing technology has contributed significantly to crop improvement [4, 5]. Some reports describe the use of CRISPR-Cas9 and base editing systems for gene editing to improve various agronomic traits in common wheat [6–16]. However, CRISPR-Cas9 generates double-strand breaks (DSBs) in DNA that disrupt genes by inducing mixtures of random insertions and deletions (indels) at target sites. Base editing can install C•G-to-T•A, A•T-to-G•C and C•G-to-G•C point mutations without requiring DSBs [13–16], but it usually induces bystander mutations when more than one C or A is present in the deamination window, and importantly, base editing cannot currently generate most transversions in the wheat genome. Therefore, more powerful, precise genome editing tools are urgently needed for functional genomics and the genetic improvement of common wheat.

Prime editing (PE) is a newly developed, versatile genome editing technology that can enable the installation of all 12 possible nucleotide substitutions, as well as short insertions or deletions, using a Moloney-murine leukemia virus reverse transcriptase (M-MLV RT) paired with an altered CRISPR/Cas9 nickase, nCas9 (H840A), and a prime editing guide RNA (pegRNA) [17]. However, prime editing suffers from low editing efficiency in plants, which has stimulated considerable efforts for its improvement [5, 18–20]. Some studies have reported driving pegRNA expression with enhanced promoters [21, 22], using dual pegRNAs [23], designing the pegRNA sequence based on melting-temperature preferences [23], and optimizing pegRNA by adding RNA motifs at the 3′ terminus of pegRNA to enhance its stability in rice and maize [24–27]. In addition, engineering prime editor by deleting the RT RNase H domain and/or fusing the RT to functional proteins such as viral nucleocapsid protein [24] and DNA mismatch repair-inhibiting protein [25–27], as well as optimizing PE protein architecture through combining a PE with N-fusion M-MLV RT and synonymous mutations in the RT template [28], could also improve prime editing activity in plants. Nevertheless, the resulting engineered prime editors tend to exhibit highly variable efficiencies at different sites and poor capability for targeting multiple genes at the same time. Furthermore, their applicability is primarily restricted to rice and maize. These limitations underscore the necessity for significant advancements to be made to develop more efficient and universal prime editors in plants, including hexaploid wheat.

**Fig. 1:**
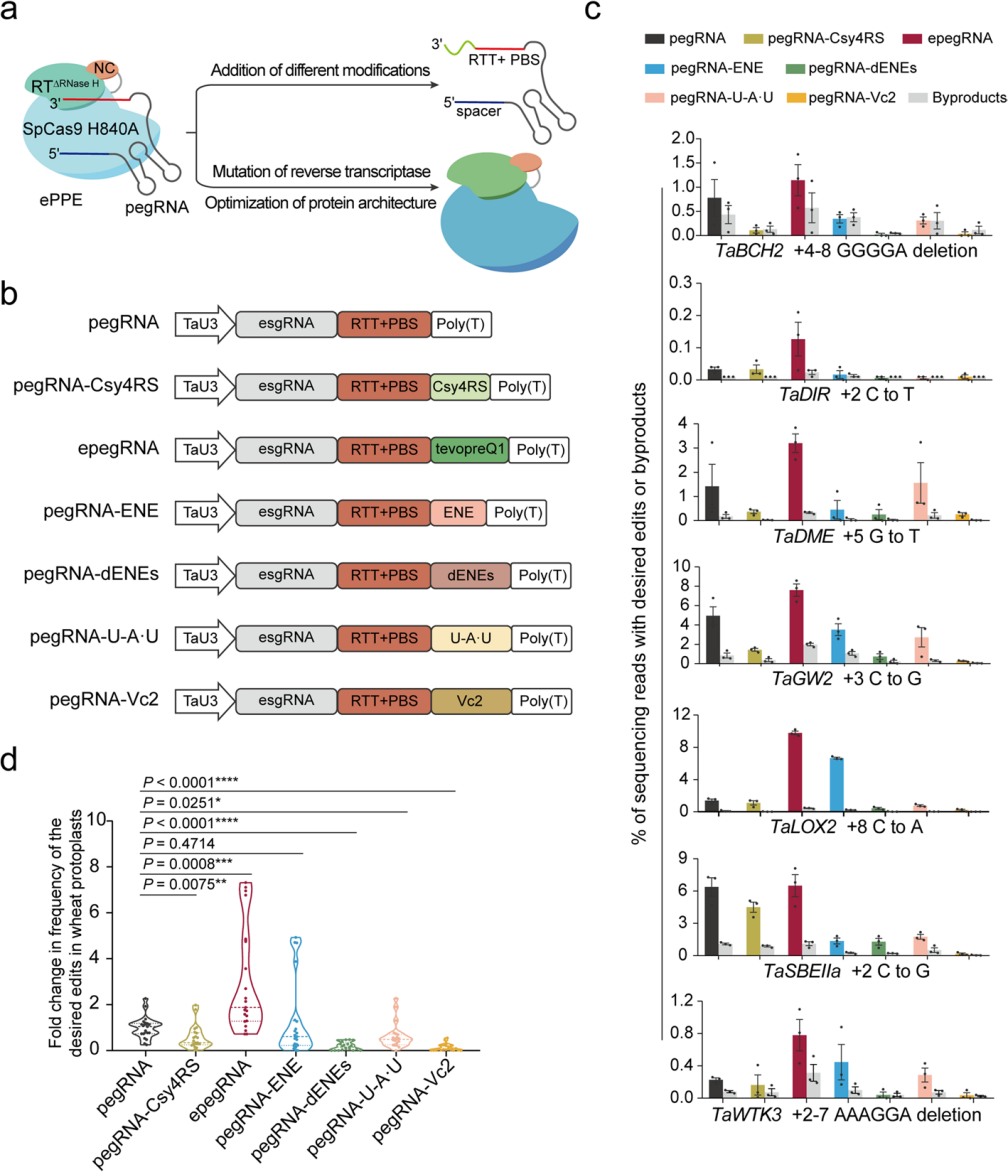
Optimized pegRNA by incorporation of different modifications at its 3′ extension. **a** Schematic diagram of engineering a prime editor by two aspects: optimizing pegRNA component by adding different modifications at 3′ extension of pegRNA, and engineering prime editor protein component by mutation of reverse transcriptase and optimization of prime editor architecture. NC, viral nucleocapsid protein. M-MLV RT, Moloney-murine leukemia virus reverse transcriptase. PBS, primer binding site. RTT, reverse transcription template. **b** Schematic representation of canonical pegRNA and six modified pegRNAs including pegRNA-Csy4RS, epegRNA, pegRNA-ENE, pegRNA-dENEs, pegRNA-U-A·U, and pegRNA-Vc2 constructs. **c** Frequencies of prime editing and byproducts induced by canonical pegRNAs and modified pegRNAs at seven wheat target sites. Frequencies (mean ± s.e.m.) were calculated from three independent experiments (*n* = 3). **d** Summary of the fold change in prime editing efficiencies for modified pegRNAs compared to canonical pegRNAs. Values were calculated from the data presented in **c**. The editing frequencies using canonical pegRNA for each target were normalized to 1, and the frequencies using other pegRNAs for each target were adjusted accordingly. *P* values were obtained using the two-tailed Student’s *t* test: *P < 0.05, **P < 0.01, ***P < 0.001 and ****P < 0.0001

Here, we developed a series of new prime editors by engineering both the pegRNA and the protein components of PE in common wheat (Fig. 1a). We found that the use of engineered pegRNA (epegRNA), along with the combination of introducing a V223A mutation into the M-MLV RT and updating the architecture of the PE protein by varying the SpCas9 activity and nuclear localization signals (NLSs), synergistically and significantly increased the efficiency of prime editing in wheat. Based on our upgraded PE, we established an efficient Csy4-endoribonuclease-mediated multiplex prime editing system with which we achieved simultaneous editing of up to ten genes in wheat protoplasts and up to eight genes in whole wheat plants with heritable mutations, thereby substantially increasing the flexibility and applicability of prime editing.

## Results

### Optimizing pegRNAs via 3' terminus modifications

Because pegRNA has an extended 3′ region containing a primer binding site (PBS) and reverse transcription template (RT template), it is susceptible to exonucleolytic degradation and the formation of unproductive secondary structures, which can undercut the performance of PE [29, 30]. Therefore, to enhance its stability, we engineered pegRNA through the addition of six different motifs at its 3′ terminus: two previously reported modifications [29, 30], the hairpin Csy4 recognition site (pegRNA-Csy4RS) and tevopreQ1 RNA motif (epegRNA), and four additional stabilizing RNA structural motifs [31–34], i.e., the element for nuclear expression (ENE), containing a U-rich internal loop flanked by short double helices (pegRNA-ENE); double ENEs (pegRNA-dENEs); the RNA triple-helical structure U-A·U (pegRNA-U-A·U); and the riboswitch aptamer from *Vibrio cholerae* (pegRNA-Vc2) (Fig. 1b and Additional file 1: Fig. S1). We used the wheat *U3* promoter (TaU3) to drive the expression of the original pegRNA and the six modified pegRNAs with the “flip and extension” (F + E) sgRNA (esgRNA) scaffold [35] (Fig. 1b), and then compared the editing activity of these seven pegRNAs at seven wheat endogenous sites (Additional file 2: Table S1). The appropriate pegRNAs were transformed into wheat protoplasts along with engineered plant prime editor (ePPE) [24], which was previously engineered by deleting M-MLV RT RNase H and adding a viral nucleocapsid (NC) protein and showed higher efficiency in plants than the traditional plant prime editor (PPE) [22]. Deep amplicon sequencing results showed that epegRNA gives the highest efficiency, up to 9.8%, with no obvious change in the ratio of the desired edits to editing byproducts (Fig. 1c and Additional file 1: Fig. S2); this efficiency was about 3.0-fold higher, on average, than that of the original pegRNA (Fig. 1d), consistent with previous results [25, 26, 30]. However, use of the five other modified pegRNAs had comparable or decreased editing efficiency as compared to that with the transitional pegRNA (Fig. 1c,d). Thus, addition of the tevopreQ1 RNA motif at the 3′ end of pegRNA enhanced prime editing efficiency in wheat.

### Mutating reverse transcriptase and optimizing prime editor architecture

**Fig. 2:**
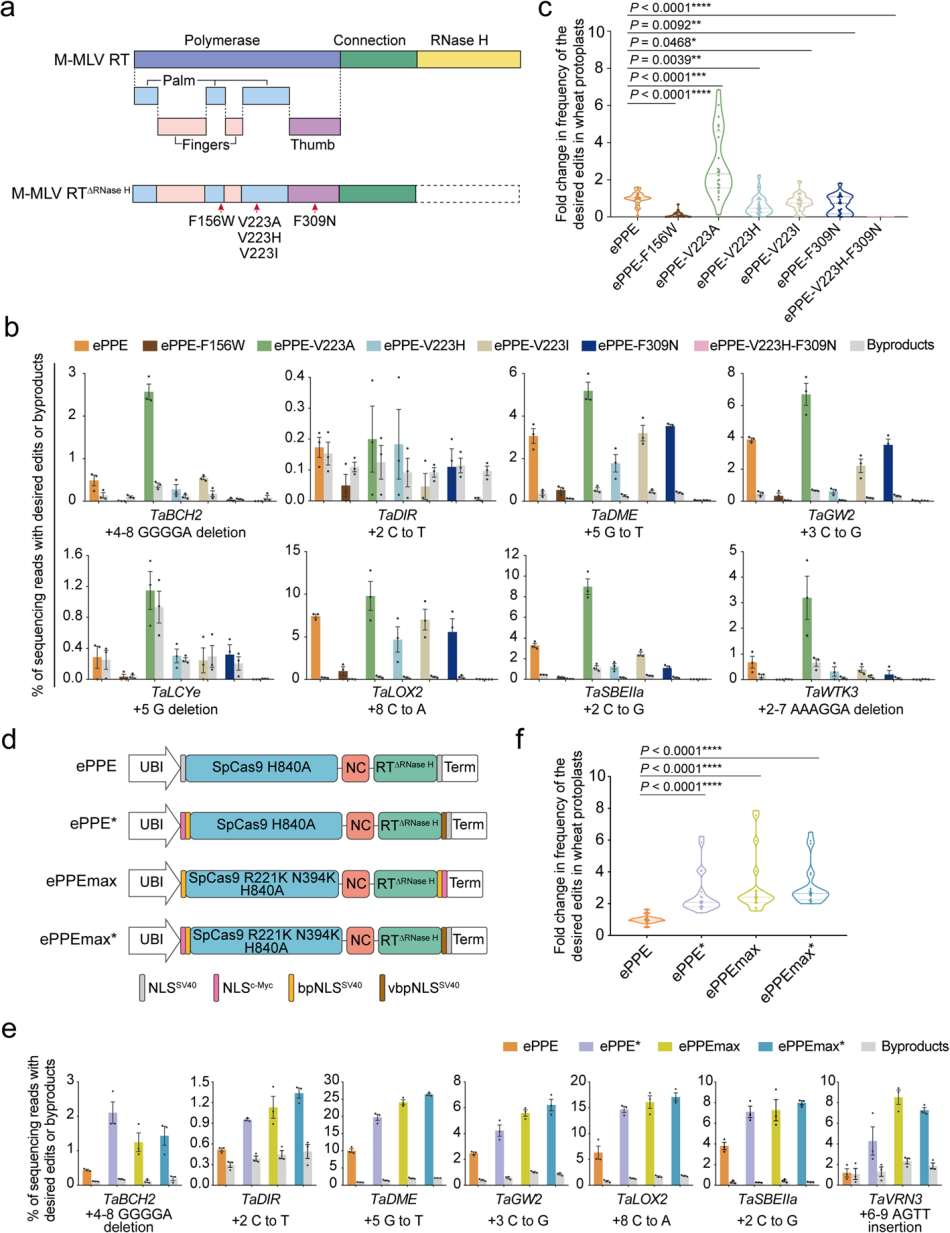
Engineered prime editors by mutating RT and changing the prime editor protein architecture. **a** Schematic representation of M-MLV RT without the RNase H domain structure showing the functionally important amino acids residues including F156 in the palm region, V223 in the palm region and F309 in the thumb region that we mutated. **b** Frequencies of prime editing and byproducts induced by ePPE, ePPE-F156W, ePPE-V223A, ePPE-V223H, ePPE-V223I, ePPE-F309N, and ePPE-V223H-F309N prime editors with epegRNAs. **c** Summary of the fold change in prime editing efficiencies for engineered ePPEs compared to ePPE. Values were calculated from the data presented in **b.** The editing frequencies using ePPE for each target were normalized to 1, and the frequencies using other ePPEs for each target were adjusted accordingly. **d** Schematic representation of ePPE, ePPE*, ePPEmax, and ePPEmax*. NLS^SV40^, the SV40 NLS; NLS^c−Myc^, the c-Myc NLS; bpNLS^SV40^, the bipartite SV40 NLS; vbpNLS^SV40^, a variant of bipartite SV40 NLS. **e** Frequencies of prime editing and byproducts induced by ePPE, ePPE*, ePPEmax, and ePPEmax*. **f** Summary of the fold change in prime editing efficiencies for ePPE*, ePPEmax and ePPEmax* compared to ePPE. Values were calculated from the data presented in **e**. The editing frequencies using ePPE for each target were normalized to 1, and the frequencies using other ePPEs for each target were adjusted accordingly. Frequencies (mean ± s.e.m.) were calculated from three independent experiments (*n* = 3) in **b** and **e**. *P* values were obtained using the two-tailed Student’s *t* test in **c** and **f**: *P < 0.05, **P < 0.01, ***P < 0.001 and ****P < 0.0001

As nCas9-RT is an important component of the prime editing system, optimizing this fusion protein is another promising approach to improve editing efficiency. Therefore, we attempted to engineer PE protein through two independent strategies, both based on the ePPE architecture [24], in parallel (Additional file 1: Fig. S3a). First, we hypothesized that engineering the RT to enhance DNA synthesis during prime editing might further improve the efficiency of ePPE. Previous studies have shown that several mutations in position F156 in the palm region of M-MLV RT, position V223 of the highly conserved YVDD motif in the palm region, and position F309N in the thumb region of M-MLV RT are important for the processivity and fidelity of RT [36–40] (Fig. 2a). Specifically, F156W could increase the fidelity of RT and might stabilize the interaction of Q190 residue with dNTP substrate, thus facilitating reverse transcription [37]; the V223A mutation has been demonstrated to enable the RT to perform faster and more efficient cDNA synthesis and higher processivity than the wild-type enzyme [38]; the V223H mutation increases the fidelity of RT and makes this enzyme more accurate [39]; the enzyme with V223I mutation increased polymerase activity compared to wild-type in the extension assay using specific template-primers [40]; RT containing F309N or V223H/F309N was less likely to incorporate incorrect nucleotides and thus had higher fidelity than the wild-type [39]. We therefore introduced these five single and one double amino acid substitutions to the corresponding positions, resulting in six new prime editors: ePPE-F156W, ePPE-V223A, ePPE-V223H, ePPE-V223I, ePPE-F309N, and ePPE-V223H-F309N (Fig. 2a and Additional file 1: Fig. S3b). Evaluating the efficiency of these six prime editors and ePPE with epegRNA at eight target sites showed that ePPE-V223A improves the editing efficiency of various base substitutions and small deletions by 1.2- to 5.3-fold (average 2.8-fold) compared to ePPE (Fig. 2b,c), without affecting the edit:byproduct ratio at most of the tested sites (Additional file 1: Fig. S3c). However, the five other point-mutant prime editors displayed lower or nearly no activity compared to ePPE (Fig. 2b,c). Therefore, these five amino-acid substitutions were not considered in further analysis. In particular, despite carrying substitutions affecting the same amino acid position, ePPE-V223H and ePPE-V223I impeded ePPE editing efficiency, whereas ePPE-V223A improved it (Fig. 2b,c), indicating that the conversion of codon 223 from valine to alanine plays an important role in improving prime editing, probably due to enhanced RT processivity.

Other recent studies had suggested that optimizing PE architecture by varying the nuclear localization signals (NLSs) and Cas activity could increase the editing efficiency in mammalian cells, rice and maize [25–27, 41, 42]. To examine whether prime editing could be improved via a similar strategy in wheat, we updated ePPE to three new architectures: ePPE*, which incorporates the c-Myc NLS and a bipartite SV40 NLS at the N terminus, and a variant bipartite SV40 NLS and SV40 NLS at the C terminus; ePPEmax, which contains an N-terminal bipartite SV40 NLS, a C-terminal bipartite SV40 NLS and c-Myc NLS, and R221K N394K mutations in SpCas9 H840A; and ePPEmax*, which introduces R221K N394K mutations into SpCas9 H840A in the ePPE* architecture (Fig. 2d). At the seven target sites tested, all three optimized prime editor architectures outperformed ePPE, with approximately 2.6 ~ 3.1-fold higher activity in wheat protoplasts (Fig. 2e,f), and with no apparent change in the proportion of byproducts (Additional file 1: Fig. S3d). Among these engineered ePPEs, ePPEmax* offered modestly higher editing efficiency, with an average of 9.7% across all sites tested, compared to ePPEmax (average of 9.1%) and ePPE* (average of 7.6%) (Fig. 2e,f and Additional file 1: Fig. S3e). These results confirm that optimization of the NLS and increased Cas9 activity can increase editing efficiency in wheat. Taken together, these results demonstrate that mutating reverse transcriptase or optimizing the prime editor architecture can each improve prime editing in plants.

### ePPEplus exhibits enhanced prime editing

**Fig. 3:**
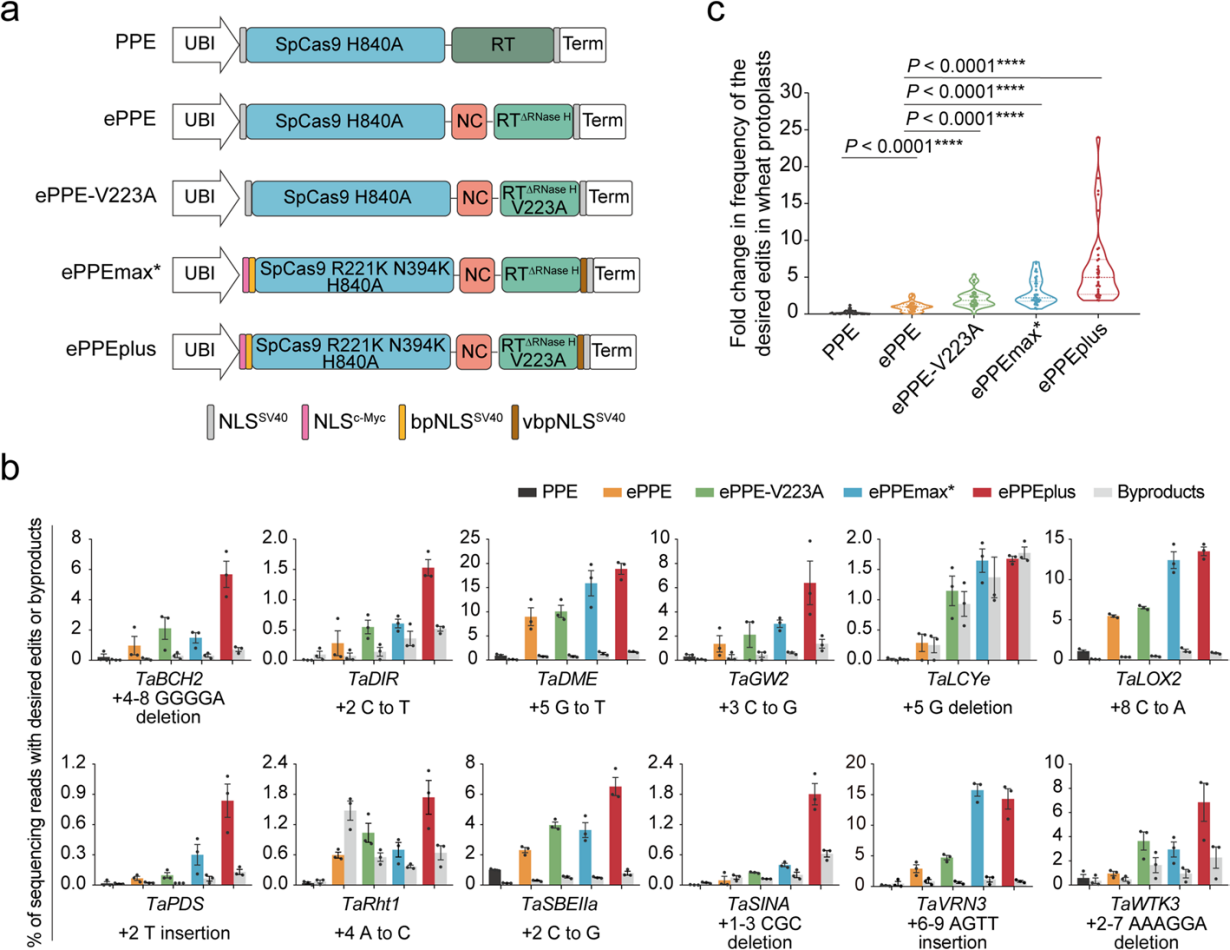
ePPEplus for improvement of prime editing efficiency. **a** Representation of the PPE, ePPE, ePPE-V223A, ePPEmax*, and ePPEplus constructs. **b** Comparison of the prime editing efficiencies and byproduct efficiencies of five different prime editors (PPE, ePPE, ePPE-V223A, ePPEmax*, and ePPEplus) at 12 target sites in wheat protoplasts. Frequencies (mean ± s.e.m.) were calculated from three independent experiments (*n* = 3). **c** Summary of the fold change in prime editing efficiencies for PPE, ePPE-V223A, ePPEmax*, and ePPEplus compared to ePPE. Values were calculated from the data presented in **b.** The editing frequencies using ePPE for each target were normalized to 1, and the frequencies using PPE and other ePPEs for each target were adjusted accordingly. *P* values were obtained using the two-tailed Student’s *t* test: ****P < 0.0001

Given that ePPE-V223A and ePPEmax* increased prime editing efficiency independently at the tested target sites, we speculated that combining these two approaches might further enhance editing activity. Therefore, we introduced the V223A mutation into the RT in the ePPEmax* architecture, producing a novel prime editor that we refer to as ePPEplus (Fig. 3a and Additional file 1: Fig. S4a). We then compared the activities of ePPEplus, ePPE-V223A, ePPEmax*, ePPE, and the original PPE across 12 wheat endogenous targets in wheat protoplasts. ePPEplus provided a substantial improvement and displayed the highest efficiency, demonstrating a 6.5- to 503.6-fold (average 33.0-fold) improvement in editing compared to PPE, a 2.1- to 19.5-fold (average 6.4-fold) compared to ePPE, up to 8.6-fold (average 3.1-fold) compared to ePPE-V223A and up to 4.6-fold (average 2.1-fold) compared to ePPEmax* (Fig. 3b,c). The frequency of intended edits, comprising C-to-T, G-to-T, C-to-G, C-to-A, A-to-C, 1–6 bp deletions and 1–4 bp insertions, introduced by ePPEplus was 6.6% on average and up to 18.9% (Fig. 3b,c). In particular, ePPEplus greatly enhanced the editing of some challenging sites, such as *TaSINA* (+ 1–3 CGC deletion, 1.8%), at which PPE and ePPE resulted in almost no editing (< 0.05%) (Fig. 3b). Furthermore, although the exact values of byproducts including pegRNA scaffold-derived byproducts, RT template-related byproducts, and some other random undesired mutations marginally increased at some target sites, the overall ratio of edit:byproduct at most tested sites was greater or comparable to that when using ePPEplus as compared to other prime editors (Fig. 3b, Additional file 1: Fig. S4b,c and Additional file 1: Fig. S5). Collectively, these results indicate that combining engineering of RT and PE protein architecture can synergistically enhance the efficiency of precise base substitution, small deletion and small insertion prime edits in plants.

### Prime-editor-mediated multiplex genome editing in wheat protoplasts

**Fig. 4:**
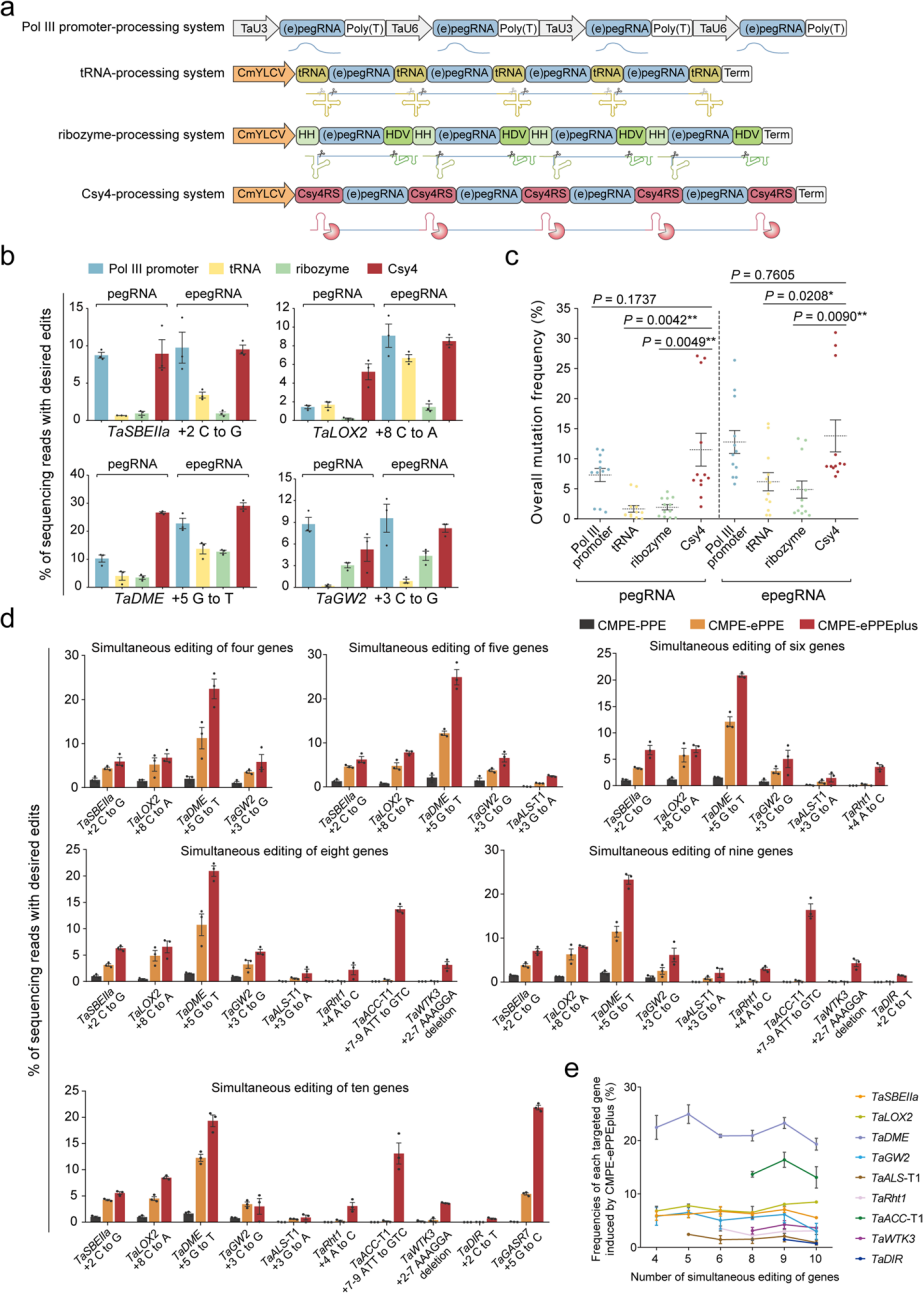
Multiplex precision gene editing mediated by prime editor in wheat protoplasts. **a** Schematic diagrams of the constructs used for four distinct multiple pegRNAs/epegRNAs processing strategies. For the Pol III promoter-processing system, the wheat *U3* (TaU3) and *U6* (TaU6) promoters were used to drive expression of each pegRNA or epegRNA. For the tRNA-, ribozyme-, and Csy4-processing systems, a Pol II promoter, the *Cestrum yellow leaf curling virus* (CmYLCV) promoter, was employed to process polycistronic pegRNA/epegRNA transcripts. Csy4RS, Csy4 recognition site; HDV, hepatitis delta virus ribozyme; HH, hammerhead ribozyme; tRNA, 77 bp pre-tRNA^Gly^ genes. **b** Comparison of the four multiplex editing systems across four genes. Both pegRNAs and epegRNAs were used. **c** Overall mutation frequencies mediated by the Pol III promoter-, tRNA-, ribozyme-, and Csy4-processing systems. *P* values were obtained using the two-tailed Student’s *t* test. *P < 0.05, **P < 0.01. **d** Multiplexed mutagenesis of four, five, six, eight, nine, and ten simultaneously edited genes using CMPE-PPE, CMPE-ePPE, and CMPE-ePPEplus. CMPE, Csy4-mediated multiplex prime editing. **e** Frequencies of each targeted gene (except for *TaGASR7*) in different arrays of epegRNAs induced by CMPE-ePPEplus. Values were calculated from the data presented in **d.** Frequencies (mean ± s.e.m.) were calculated from three independent experiments (*n* = 3) in **b**, **d,** and **e**

Given the complexity and redundancy of plant genomes, studying gene functions or deciphering a complex trait conferred by multiple genes/loci usually requires introducing multiple mutations simultaneously (“stacking” mutations), especially in polyploid species such as hexaploid wheat [4–7]. Prime editing, with its flexibility and robustness, provides a promising platform for editing multiplex genomes in a site-specific manner. To efficiently and simultaneously produce multiple pegRNAs, we first compared and evaluated the efficiency of targeted mutagenesis using four different processing strategies [43–50] (Fig. 4a): a procedure in which two separate Pol III promoters (*U3* and *U6*) were used to drive expression of each guide RNA, a polycistronic tRNA processing system, a self-cleaving ribozyme processing system, and a Csy-type ribonuclease 4 (Csy4) processing system that requires the simultaneous presence of the Csy4 protein. The latter three systems used a Pol II promoter from *Cestrum yellow leaf curling virus* (CmYLCV) to drive expression. We chose four endogenous genes (*TaSBEIIa*, *TaLOX2*, *TaDME*, and *TaGW2*) for simultaneous editing and arranged them in the same random order for testing using each processing system to provide a close comparison (Fig. 4b). pegRNAs or epegRNAs were co-transformed with ePPEplus into wheat protoplasts. Targeted amplicon sequencing demonstrated that epegRNA induces higher activity for multiplex genome editing, from 1.3-fold to 4.2-fold greater than with pegRNA regardless of the processing strategy used (Fig. 4b,c and Additional file 1: Fig. S6a,b), which was consistent with our above results for the editing of single sites (Fig. 1d). Among these four epegRNA-processing strategies, the Csy4 processing system had slightly higher efficiency (averaging 13.8%) than the use of individual Pol III promoter system (average 12.8%), and both of them performed much better than the tRNA system (average 6.2%) and the ribozyme system (average 4.9%) (Fig. 4b,c). In addition, there were no obvious differences in the edit:byproduct ratio among these systems (Fig. 4b and Additional file 1: Fig. S6c,d). Based on these results, we selected *C*sy4-mediated *m*ultiplex *p*rime *e*diting (CMPE) for further study.

To further explore the capacity and effectiveness of the CMPE system for multiplexing, we designed Csy4 arrays of four, five, six, eight, nine, and ten epegRNAs, respectively targeting four, five, six, eight, nine, and ten genes simultaneously (Fig. 4d). We transformed wheat protoplasts with these epegRNA arrays using CMPE-PPE, CMPE-ePPE, and CMPE-ePPEplus (Additional file 1: Fig. S7a). CMPE-ePPEplus showed an outstanding performance, introducing desired edits with 10.3% (5.8–22.5%), 9.6% (2.4–24.9%), 7.4% (1.5–20.9%), 7.5% (1.5–20.9%), 8.0% (1.5–23.3%), and 8.0% (0.7–21.9%) efficiencies at all four, five, six, eight, nine, and ten target genes, respectively (Fig. 4d and Additional file 1: Fig. S7b). In addition, we found no significant changes in editing efficiency at a given site in conjunction with the increased number of target genes (Fig. 4e). As compared to CMPE-PPE and CMPE-ePPE, CMPE-ePPEplus exhibited on average a 49.1-fold (up to 109.3-fold) and 10.5-fold (up to 22.9-fold) higher efficiency for editing of four to ten genes (Additional file 1: Fig. S7c,d), and a 95.6-fold (up to 1399.7-fold) and 17.2-fold (up to 67.3-fold) higher efficiency for a given gene, respectively (Additional file 1: Fig. S7e,f). These results highlight how CMPE could be broadly effective at boosting the targeting capability and editing efficiency of prime editing toolkits in wheat protoplasts.

### Efficient multiplex prime editing in transgenic wheat plants

**Fig. 5:**
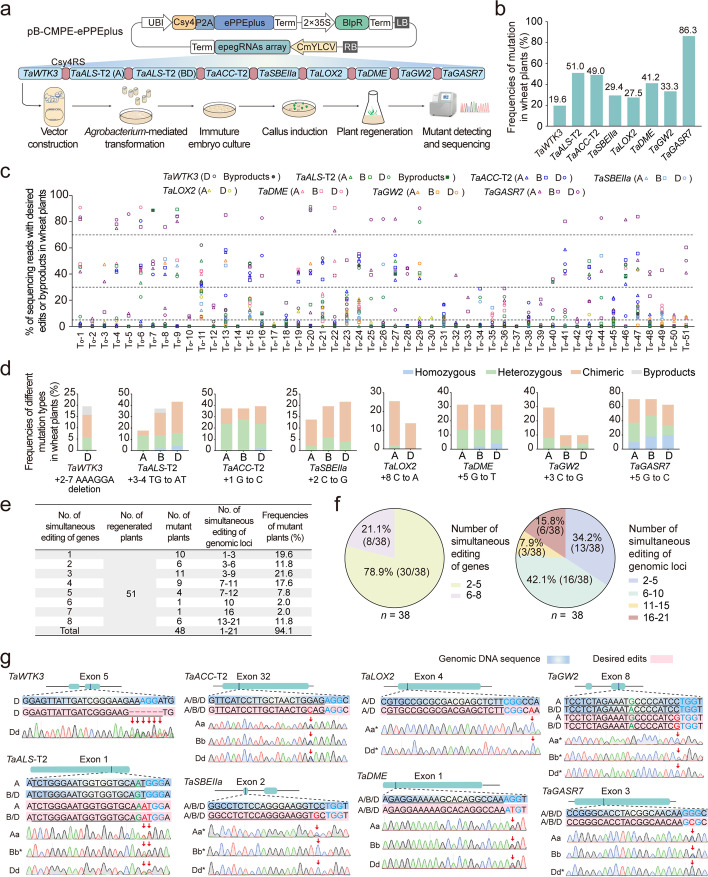
Prime-editor-mediated multiplex precision genome editing in transgenic wheat plants. **a** Pipeline for prime editing of multiple endogenous genes by pB-CMPE-ePPEplus in wheat. Nine epegRNAs targeting eight endogenous genes were assembled in one Csy4 array*.* Of these genes, *TaWTK3*, *TaACC-*T2, *TaSBEIIa*, *TaLOX2*, *TaDME, TaGW2,* and *TaGASR7* were targeted with a corresponding epegRNA, and with two epegRNAs respectively targeting copies of *TaALS*-T2 on the A and B/D subgenomes. *BlpR*, Bialaphos resistance. **b** Mutation frequencies of individual targeted genes in regenerated wheat plants. **c** Mutation frequencies of homoeologous genes in the A/B/D subgenomes for each targeted gene at each regenerated plant. Mutation efficiencies were examined by NGS with 5% threshold. Mutation frequency ≥ 70% was counted as homozygous mutation; mutation frequency ≥ 30 and < 70% was counted as heterozygous mutation; mutation frequency ≥ 5 and < 30% was counted as chimeric mutation; mutation frequency < 5% was counted as wild-type. When the main mutation type in a homozygous/ heterozygous/chimeric line contains undesired edits, it was counted as byproducts mutation. **d** Editing efficiencies of different mutation types of homoeologous genes in the A/B/D subgenomes for each targeted gene. **e** Frequencies of multiplex prime editing in regenerated wheat plants. **f** Ratio of simultaneous editing of different numbers of genes or genomic loci. *n* = 38 refers to the number of plants harboring two to eight genes mutated simultaneously. **g** Sanger sequencing chromatograms of the T_0_-11 mutant harboring the desired prime edits in all eight genes. “*” indicates that the mutation type is chimeric. The protospacer-adjacent motif (PAM) sequence is highlighted in blue. The SNPs in different subgenomes are highlighted in green. The desired edits are highlighted in red

To investigate the performance of CMPE-ePPEplus in whole wheat plants, we designed one array of nine epegRNAs (Fig. 5a), in a random order, to target eight endogenous wheat genes (*TaWTK3*, *TaALS-*T2, *TaACC-*T2, *TaSBEIIa, TaLOX2*, *TaDME, TaGW2,* and *TaGASR7*) that control important agronomic traits related to disease resistance, herbicide resistance, yield, and/or quality. We first constructed a binary expression vector, pB-CMPE-ePPEplus, carrying the epegRNA array, the ePPEplus-P2A-Csy4 expression cassette and the *bar* gene and then introduced this vector into wheat immature embryos by *Agrobacterium*-mediated transformation (Fig. 5a). By examining 51 regenerated individual plants through deep amplicon sequencing and Sanger sequencing, we identified 48 plants harboring mutations in at least one targeted gene (overall mutation frequency of 94.1% [48/51]) (Fig. 5b–e, Additional file 1: Fig. S8 and Additional file 2: Table S2). The mutagenesis efficiency was 19.6% at *TaWTK3* (+ 2–7 AAAGGA deletion), 51.0% at *TaALS-*T2 (+ 3–4 TG-to-AT), 49.0% at *TaACC-*T2 (+ 1 G-to-C), 29.4% at *TaSBEIIa* (+ 2 C-to-G), 27.5% at *TaLOX2* (+ 8 C-to-A), 41.2% at *TaDME* (+ 5 G-to-T), 33.3% at *TaGW2* (+ 3 C-to-G), and 86.3% at *TaGASR7* (+ 5 G-to-C) (Fig. 5b and Additional file 2: Table S3). Upon assessing the genotypes of the 51 transformed plants with regard to individual genes in the A, B, and D subgenomes, we identified all possible examples of desired homozygous (from 2.0 to 19.6%), heterozygous (from 2.0 to 29.4%), chimeric (from 3.9 to 33.3%) and byproducts (3.9%) for each targeted subgenome (Fig. 5c,d, Additional file 1: Figs. S8, S9 and Additional file 2: Table S3). In addition, mutations occurred in all three subgenomes, sometimes simultaneously, for each gene, at efficiencies ranging from 5.9 to 54.9% (Additional file 1: Fig. S10a and Additional file 2: Table S3). More importantly, we identified various combinations of mutants involving three homoeologs when targeting the conserved region they shared: for example, at the *TaGASR7* target, eight of 44 mutants carried the desired G-to-C mutation in one subgenome, eight in two subgenomes and 28 in all three subgenomes, and in particular, two plants had all six alleles simultaneously edited (Fig. 5c,d and Additional file 2: Table S3). These results suggest that the CMPE-ePPEplus system could induce efficient prime editing in all homoeologs of a single wheat gene.

Next, we investigated the ability of CMPE-ePPEplus to target multiple genes simultaneously. We obtained 38 mutants harboring multiplex genome editing of two to eight genes, with a simultaneous editing frequency of 74.5% (38/51) (Fig. 5e and Additional file 2: Table S4). Of 38 mutants, 30 plants had mutations in two to five genes and eight plants had mutations in more than five (six to eight) genes (Fig. 5f, Additional file 1: Fig. S10b and Additional file 2: Table S4). In addition, nine mutants had more than ten genomic loci and six had more than 15 genomic loci (up to 21 genomic loci) edited simultaneously (Fig. 5f, Additional file 1: Fig. S10c and Additional file 2: Table S4). Specifically, six, eleven, nine, four, one, one, and six plants harbored simultaneous mutations in two, three, four, five, six, seven, and eight genes, at frequencies of 11.8, 21.6, 17.6, 7.8, 2.0, 2.0, and 11.8%, respectively (Fig. 5e, Additional file 1: Fig. S10b and Additional file 2: Table S4). When editing two, three, four, and five genes simultaneously, we obtained mutants with a variety of different editing combinations (Additional file 2: Table S4). For example, when editing five genes, we obtained four mutants with four different combinations of edited genes: *TaALS*-T2 + *TaACC-*T2 + *TaDME* + *TaGW2* + *TaGASR7*, *TaALS*-T2 + *TaACC-*T2 + *TaSBEIIa* + *TaDME* + *TaGASR7*, *TaALS*-T2 + *TaACC-*T2 + *TaLOX2* + *TaGW2* + *TaGASR7* and *TaWTK3* + *TaALS*-T2 + *TaACC-*T2 + + *TaLOX2* + *TaGW2* (Additional file 2: Table S4). Importantly, six plants (T_0_-11, T_0_-13, T_0_-15, T_0_-21, T_0_-24, and T_0_-47) had undergone mutations in all eight genes, which introduced four types of single base substitutions (C-to-G, G-to-C, C-to-A and G-to-T), one type of double base substitution (TG-to-AT) and one type of small (6-bp) deletion simultaneously (Fig. 5e, Additional file 1: Fig. S10b and Additional file 2: Table S4). For example, T_0_-11 harbored desired mutations at 21 genomic loci, including heterozygous mutations at *TaWTK3* (Dd), *TaACC-*T2 (AaBbDd), and *TaDME* (AaBbDd) and chimeric mutations at *TaALS-*T2 (AaBbDd), *TaSBEIIa* (AaBbDd), *TaLOX2* (AaDd), *TaGW2* (AaBbDd), and *TaGASR7* (AaBbDd) (Fig. 5g and Additional file 2: Table S4). We also obtained ten independent lines with mutation of only one targeted endogenous gene (*TaLOX2* or *TaGASR7*), at an efficiency of 19.6% (Fig. 5e, Additional file 1: Fig. S10b and Additional file 2: Table S4). Collectively, these results demonstrate that CMPE-ePPEplus is an efficient and versatile platform for multiplex prime editing in wheat, providing great promise for the simultaneous manipulation of multiple agronomic traits.

### Effect of off-target prime editing in wheat plants

Off-target editing is another major concern with current prime editing methods. Thus, we examined the probability of off-target effect in plants for each target gene based on pegRNA-dependent off-target edits. We did not detect any mutations at potential off-target regions (defined as sites with no more than three mismatches in the spacer) in 51 wheat plants (Additional file 2: Table S5). Next, we examined off-target effects among highly similar common wheat homoeologs using *TaGW2-A*, *TaGW2-B* and *TaGW2-D* as an example. The spacer sequence of *TaGW2*-epegRNA was strictly conserved in *TaGW2-A* but had a 1-bp mismatch to the cognate target sites in *TaGW2-B* and *TaGW2-D* (Additional file 1: Fig. S9g and Additional file 2: Table S5). Off-target frequencies caused by this mismatch in *TaGW2-B* (5/51, 9.8%) and *TaGW2-D* (5/51, 9.8%) were lower than the on-target mutagenesis frequencies in *TaGW2-A* (15/51, 29.4%) (Fig. 5c,d, Additional file 1: Fig. S9g and Additional file 2: Tables S3,S5). The observed level of off-target effects may be reasonable, because the 1-bp mismatch was located at position 12 of the spacer, corresponding to position six of the PBS sequence counting distal to the nick site, which has been reported to easily lead to off-target mutagenesis [51].

### Mutation transmission and transgene-free analysis

To investigate whether the mutations could be transmitted to the next generation, we self-fertilized T_0_-1 (with mutations in three genes), T_0_-13 (with mutations in all eight genes), T_0_-20 and T_0_-29 (with mutations in five genes), and T_0_-43 and T_0_-46 plants (with mutations in four genes) (Additional file 2: Table S6). We screened 15 to 46 T_1_ seedlings from each T_0_ parent for mutations in the respective genes by PCR and deep amplicon sequencing. For homozygous mutations, the transmission rates were 100%; for the majority of heterozygous mutants, Mendelian segregation occurred; for chimeric mutations in the T_0_ plants, the transmission rates ranged from 0 to 63.2%. For example, in plant T_0_-1, mutations in *TaDME-D*, *TaGASR7-B* and *TaGASR7-D* that were homozygous in the T_0_ plant were present in all T_1_ progenies; mutations in *TaALS-*T2*-B*, *TaDME-A*, *TaDME-B*, and *TaGASR7-A* that were heterozygous in T_0_ plants segregated at a 1:2:1 ratio in T_1_ progeny. By contrast, mutation in *TaALS-*T2*-D* that was chimeric in T_0_ plants resulted in only two plants in the T_1_ generation harboring chimeric mutations (Additional file 2: Table S6). Notably, some new mutations were detected in the T_1_ plants for some targets whereas the T_0_ plants were wild-type (e.g., *TaACC-*T2*-B* site of the T_0_-20 line) (Additional file 2: Table S6), suggesting that the prime editors remained active in T_0_ and/or T_1_ plants. Furthermore, to examine the possibility of achieving targeted modifications without incorporating foreign DNA into the common wheat genome, we identified these T_1_ progenies with four primer sets specific for pB-CMPE-ePPEplus (Additional file 1: Fig. S11a), and found that the frequencies of mutants without detectable transgenes were 4.2% (T_0_-1), 15.8% (T_0_-13), 21.1% (T_0_-20), 0.0% (T_0_-29), 6.7% (T_0_-43), and 4.3% (T_0_-46) (Additional file 1: Fig. S11b-g and Additional file 2: Table S6). Collectively, these results demonstrate the feasibility of using the Csy4-mediated multiplex prime editing system to effectively generate heritable mutations in multiple genes, and a transgene-free plant carrying only the desired DNA sequence change can be obtained through genetic segregation.

## Discussion

Prime editing, a newly developed, versatile genome editing tool, has been used in plants, but is limited by its low efficiency, targeting of only a single site at a time and its applicability primarily in rice and maize. Here, we developed an upgraded version of prime editing in hexaploid wheat by engineering both the pegRNA and PE protein components of the prime editing system. First, by testing six different motifs appended to the 3′ end of pegRNA, we found that only the tevopreQ1 RNA structure (epegRNA) provided better editing efficiency than the original pegRNA, consistent with results in human cells, rice cells, and maize cells [24–27, 30]. There are several possible reasons why the five other motifs decreased the editing efficiency. Perhaps these motifs affect the secondary structure and stability of pegRNA, or influence the transcription of pegRNA (e.g., the presence of four or five consecutive Ts in ENE, dENEs and U-A·U motifs might terminate the transcription of the Pol III promoter), or perhaps the differences in editing efficiency reflect a difference in pegRNA processing between wheat cells and animal cells (e.g., the Csy4 motif is more efficient in human cells [29]). We also demonstrated two effective approaches to engineering PE components that enhance the overall activity of the system: mutating the reverse transcriptase and optimizing PE protein architecture. In addition, introducing a V223A substitution into the M-MLV RT in the ePPEmax* architecture containing heterogeneous tandem NLSs and R221K N394K mutations in SpCas9 H840A (resulting in ePPEplus) cooperatively and substantially improved prime editing efficiency in wheat as compared to that with either the original PPE or ePPE. Moreover, we tried to optimize PE through a previously reported strategy of fusing together three functional proteins: the chromatin remodeling factor HMGN1/H1G [52], the ssDNA binding protein Rad51 [53], and an engineered version of the DNA mismatch repair-inhibiting protein hMLH1dn [42]. However, the efficiencies of all these engineered PEs were comparable to or lower than that of ePPE (Additional file 1: Fig. S12), which contrasts with results in mouse and human cells but is consistent with results in rice [25, 26, 42, 52, 53]. These results may thus reflect differences in the cellular factors that influence prime editing outcomes in mammalian as compared to plant cells. Even so, the combination of epegRNA with ePPEplus developed in this study could make formerly challenging target sites editable, and thus it expands the scope and capabilities of prime editing.

Multiplexed genome editing that targets different genomic loci or multiple genes is highly desirable for regulating gene expression, stacking changes for multiple traits, and controlling regulatory pathways. Many convenient, efficient multiplexed sgRNA systems for CRISPR-Cas9 have been developed in plants involving the use of several Pol III promoters (*U3* and *U6*) to express multiple sgRNAs in a single construct, the production of numerous sgRNAs via the endogenous tRNA-processing system, and the use of self-cleaving ribozyme and Csy-type ribonuclease 4 (Csy4)-processing systems. Comparisons of these four strategies in plant cells revealed that the Csy4 and tRNA systems showed more robust genome editing efficiencies than the self-cleaving ribozyme and conventional mixed dual promoter systems [48, 49]. In the current study, in order to leverage the versatility of prime editing to achieve multiplex genome editing in wheat, after evaluating these four strategies, we developed an efficient Csy4-mediated multiplex prime editing platform (CMPE) with ePPEplus. Although the CMPE system requires expression of an extra gene encoding the Csy4 ribonuclease, the Csy4 protein is relatively short (187 amino acids), and we typically expressed the encoding protein as a P2A fusion. More importantly, we efficiently achieved simultaneous editing of up to ten genes in wheat protoplasts and up to eight genes in transgenic wheat plants using the CMPE-ePPEplus system. In addition, segregation analysis of six T_0_ lines indicated that the precisely edited genes could be inherited by the following subsequent generation and transgene-free prime-edited mutants could be obtained. To our knowledge, this is the first time that prime-edited plants and multiplex prime editing have been achieved in common wheat. Compared to previously reported Cas9-mediated multiplex genome editing [7, 9–12, 43–50], the prime-editor-mediated multiplex genome editing system in wheat (in this study) and in other species [54, 55] carries the advantages of more precise and more diversified mutation types and wider adaptability, paving the way to manipulating the genome in a synthetic manner. Nonetheless, the overall editing efficiency, the position effect of target sites, and the capacity of CMPE still need to be further explored.

## Conclusions

In conclusion, we developed upgraded prime editing systems by engineering both the pegRNA and the protein components of PE that enable the prime editing with improved efficiency and multiplex precision editing in common wheat. These efficient and versatile prime editing systems will expand the applicability of genomic engineering, and provides new and powerful technical options for the stacking of superior traits in plants, especially polyploid crops.

## Materials and methods

### Plasmid construction

The plasmids of ePPE-F156W, ePPE-V223A, ePPE-V223H, ePPE-V223I, ePPE-F309N, and ePPE-V223H-F309N were mutated by mismatch PCR and cloned into the ePPE construct backbone [24]. To construct vectors of ePPE*, ePPEmax, and ePPEmax*, double R221K/N394K mutations were introduced by mismatch PCR, and vbpNLS^SV40^, bpNLS^SV40^ and NLS^c−myc^ were codon-optimized for wheat and amplified using primer sets containing the relative sequences and cloned into the ePPE construct backbone. To construct the ePPEplus vector, the V223A mutation in M-MLV RT by mismatch PCR and cloned into the ePPEmax* vector backbone. To construct vectors of CMPE-PPE, CMPE-ePPE, and CMPE-ePPEplus, Csy4 protein sequences were cloned into the PPE [22], ePPE [24], and ePPEplus vector backbone, respectively. To construct the binary vector pB-CMPE-ePPEplus for *Agrobacterium*-mediated wheat transformation, ePPEplus-P2A-Csy4 protein and the epegRNAs array were cloned into the pBUE411 [56] backbone using a ClonExpressII One Step Cloning Kit (Vazyme). To construct vectors of ePPE-HMGN1-H1G, ePPE-Rad51-v1, ePPE-Rad51-v2, ePPE-hMLH1dn-v1, and ePPE-hMLH1dn-v2, HMGN1, H1G, Rad51, and hMLH1dn proteins were codon-optimized for wheat and synthesized commercially by GeneScript and the fusion protein sequences were cloned into the ePPE vector backbone. All vectors used in this study are listed in Additional file 3: Sequences S1-S4.

To construct vectors of pegRNA-Csy4RS, epegRNA, pegRNA, pegRNA-ENE, pegRNA-dENEs, pegRNA-U-A·U, and pegRNA-Vc2, the Csy4RS, tevopreQ1, ENE, dENEs, U-A·U, and Vc2 were amplified using primer sets containing relative sequences, and the resultant fragment was cloned into the TaU3-esgRNA vector, which was constructed by cloning the wheat *U3* promoter and esgRNA scaffold into the pUC57 backbone. We designed pegRNA sequences using PlantpegDesigner [23] and the different pegRNA expression vectors targeting single sites were constructed as reported previously [22]. To construct pegRNA or epegRNA vectors for different multiplex prime editing systems, each pegRNA or epegRNA was cloned into the pUC57-CmYLCV vector (cloning the CmYLCV promoter and CaMV terminator into the pUC57 backbone), one by one using a ClonExpressII One Step Cloning Kit (Vazyme). PCR was performed using TransStart FastPfu DNA Polymerase (TransGen Biotech). Primer sets used in this study are listed in Additional file 2: Table S7.

### Wheat protoplast transfection

We used the spring wheat variety Fielder to prepare protoplasts. Wheat protoplast isolation and transformation were performed as described [57]. We used the Wizard Plus Midipreps DNA Purification System (Promega) to extract plasmids for protoplast transformation. The plasmids (5 µg per construct) were introduced by PEG-mediated transfection. The average efficiency of transformation was about 40%. The transfected protoplasts were incubated at 25 °C for 48 h. The protoplast genomic DNA was extracted after incubation.

### DNA extraction

We used 2 × CTAB solution (Coolaber) to extract the genomic DNA of protoplasts and leaves of each plant. The genomic DNA was quantified with a NanoDrop 2000 spectrophotometer (Thermo Fisher Scientific).

### Amplicon deep sequencing and data analysis

We designed two rounds of PCR amplification. In the first round of PCR, we amplified the target site sequences from protoplast genomic DNA or plant genomic DNA with site-specific primers. In the second round, amplification primers containing forward and reverse barcodes were added to the PCR products for library construction. The amplified products were purified using the EasyPure PCR Purification Kit (TransGen Biotech) and quantified with a NanoDrop 2000 spectrophotometer (Thermo Fisher Scientific). Equal amounts of PCR product were pooled and sequenced commercially (Novogene) using the NovaSeq platform. For all prime editing yield quantification, prime editing efficiency was calculated as described previously [24]. The percentages of byproducts during the installation of point mutations and installation of deletions or insertions were calculated as described previously [24]. For each target site, amplicon sequencing was repeated three times using genomic DNA extracted from three independent protoplast samples. The primers are listed in Additional file 2: Table S7.

### Fold change analysis

As there are large differences in efficiencies across different target sites, when we calculated the fold change, we normalized these efficiencies. Specifically, in Fig. 1d and Additional file 1: Fig.S2b, the average editing efficiencies of three repeats of pegRNA were normalized to 1 for each target and then the frequencies using other pegRNA forms for each target were adjusted accordingly; the average editing efficiencies of three repeats of ePPE in Figs. 2c, f and 3c were normalized to 1, CMPE-PPE in Additional file 1: Fig.S7c,e were normalized to 1, CMPE-ePPE in Additional file 1: Fig.S7d,f were normalized to 1 for each target, and then the frequencies using other plant prime editors for each target were adjusted accordingly. The editing efficiencies of all three repeats are presented in related figures.

### *Agrobacterium*-mediated transformation of wheat immature embryos

Binary plasmid pB-CMPE-ePPEplus containing the epegRNAs array and ePPEplus-P2A-Csy4 expression cassette was transformed into bread wheat cultivar Fielder using *Agrobacterium*-mediated gene transformation [58].

### Genotyping of transgenic wheat plants

Mutant plant genomic DNA was extracted and PCR-amplified with 2 × Phanta Max Master Mix (Vazyme). Deep amplicon sequencing using universal primers and/or specific primers spanning the target sites was first used to detect the mutations of prime-edited wheat plants. These edited wheat plants were categorized into five genotypes including homozygous, heterozygous, chimeric, byproducts, and wild-type following these criteria [25, 59]: homozygous, mutation frequency ≥ 70% without undesired byproducts; heterozygous, mutation frequency ≥ 30% and < 70% without undesired byproducts; chimeric, mutation frequency ≥ 5% and < 30% without undesired byproducts according previous studies; byproducts, when the main mutation type in a homozygous/ heterozygous/chimeric line contains undesired edits, we counted it as byproduct lines; and wild-type, mutation frequency < 5%. Then, Sanger sequencing using primers specific for subgenome A, B, or D was used to confirm the genotype and its chromatograms were analyzed using SnapGene software at each target site.

### Prediction of epegRNA spacer-like off-target edits

The epegRNA spacer-like off-target sites were predicted with CRISPR-Cereal [60]. The maximum mismatch was set at three.

### Detection of transgenes

The transgenes were investigated by examining the presence of plasmid DNA in the T_1_ plants using PCR as reported previously [61]. Primer sets were designed specifically for four discrete regions in the pB-CMPE-ePPEplus construct, representing all major parts. None of the four primer sets yielded the expected PCR amplicon in related plant, indicating that they were transgene-free.

### Statistical analysis

The data were analyzed using GraphPad Prism 8 software. All numerical values are presented as mean ± s.e.m. Differences between control and treatments were tested using two-tailed Student’s *t* tests.

## Supplementary Information

**Additional file 1: Fig. S1.** The secondary structure of different modifications examined in this study. **Fig. S2.** Product purity for pegRNAs with different modifications. **Fig. S3.** The strategies and product purity for optimized prime editors. **Fig. S4.** Product purity for PPE, ePPE, ePPE-V223A, ePPEmax* and ePPEplus. **Fig. S5.** Mutation type and percentages of byproducts for PPE, ePPE, ePPE-V223A, ePPEmax* and ePPEplus. **Fig. S6.** Comparison of multiple pegRNAs processing strategies in wheat protoplasts. **Fig. S7.** CMPE-mediated multiplex prime editing in wheat protoplasts. **Fig. S8.** Mutation type of prime-edited wheat plants in T_0_ generation. **Fig. S9.** Sanger sequencing chromatograms of prime-edited wheat plants for each target gene in T_0 _generation. **Fig. S10.** Multiplex prime editing by CMPE-ePPEplus in transgenic wheat plants in T_0_ generation. **Fig. S11.** Construct used for multiplex prime editing and detection of transgene integration in the T_1_ generation. **Fig. S12.** Engineered prime editors by fusion of different proteins with ePPE in wheat protoplasts.

**Additional file 2: Table S1.** pegRNA target sites, RT templates and PBS sequences. **Table S2.** Summary of genotypes of individual plants induced by CMPE-ePPEplus in wheat plants in T_0_ generation. **Table S3.** Summary of mutations in each targeted gene induced by CMPE-ePPEplus in wheat plants in T_0_ generation. **Table S4.** Summary of simultaneous editing of multiple genes induced by CMPE-ePPEplus in wheat plants in T_0_ generation. **Table S5.** Analysis of potential off-target effects in regenerated wheat plants. **Table S6.** Segregation and transegene-free analysis of six T_0_ lines drived from CMPE-ePPEplus [63]. **Table S7.** PCR primers used in this study.

**Additional file 3: Sequences S1.** Complete sequences of Csy4RS, tevopreQ1, ENE, dENEs, U-A·U and Vc2 modifications in this study. **Sequences S2.** Complete coding sequences of CmYLCV promoter, Pol III promoter, tRNA and ribozyme in this study. **Sequences S3.** The full plasmid sequences of ePPE-V223A, ePPEmax*, ePPEplus, CMPE-ePPEplus, pUC57-CmYLCV and pB-CMPE-ePPEplus in this study. **Sequences S4.** Complete coding sequences of PPE, ePPE, ePPE-F156W, ePPE-V223H, ePPE-V223I, ePPE-V223H, ePPE-V223H-F309N, ePPE*, ePPEmax, ePPE-HMGN1-H1G, ePPE-Rad51-v1, ePPE-Rad51-v2, ePPE-hMLH1dn-v1, ePPE-hMLH1dn-v2, CMPE-PPE and CMPE-ePPE in this study.

**Additional file 4.** Review history.

## Data Availability

Deep sequencing data are available in the NCBI database under SRA accession numbers PRJNA917452 [62]. Plasmids of ePPE-V223A, ePPEmax*, ePPEplus, CMPE-ePPEplus, pUC57-CmYLCV, and pB-CMPE-ePPEplus will be available from addgene.
