# Supplementary material for: Efficient and versatile multiplex prime editing in hexaploid wheat
Source: Genome Biol. 2023 Jun 29;24:156. doi: 10.1186/s13059-023-02990-1 (PMC10308706; doi:10.1186/s13059-023-02990-1)
Supplement: Supplementary file 1 — Additional file 1: Fig. S1. The secondary structure of different modifications examined in this study. Fig. S2. Product purity for pegRNAs with different modifications. Fig. S3. The strategies and product purity for optimized prime editors. Fig. S4. Product purity for PPE, ePPE, ePPE-V223A, ePPEmax* and ePPEplus. Fig. S5. Mutation type and percentages of byproducts for PPE, ePPE, ePPE-V223A, ePPEmax* and ePPEplus. Fig. S6. Comparison of multiple pegRNAs processing strategies in wheat protoplasts. Fig. S7. CMPE-mediated multiplex prime editing in wheat protoplasts. Fig. S8. Mutation type of prime-edited wheat plants in T0 generation. Fig. S9. Sanger sequencing chromatograms of prime-edited wheat plants for each target gene in T0 generation. Fig. S10. Multiplex prime editing by CMPE-ePPEplus in transgenic wheat plants in T0 generation. Fig. S11. Construct used for multiplex prime editing and detection of transgene integration in the T1 generation. Fig. S12. Engineered prime editors by fusion of different proteins with ePPE in wheat protoplasts. [file 13059_2023_2990_MOESM1_ESM.docx]

**Additional file 1**

**Fig. S1** The secondary structure of different modifications examined in this study.

**Fig. S2** Product purity for pegRNAs with different modifications.

**Fig. S3** The strategies and product purity for optimized prime editors.

**Fig. S4** Product purity for PPE, ePPE, ePPE-V223A, ePPEmax* and ePPEplus.

**Fig. S5** Mutation type and percentages of byproducts for PPE, ePPE, ePPE-V223A, ePPEmax* and ePPEplus.

**Fig. S6** Comparison of multiple pegRNAs processing strategies in wheat protoplasts.

**Fig. S7** CMPE-mediated multiplex prime editing in wheat protoplasts.

**Fig. S8** Mutation type of prime-edited wheat plants in T_0_ generation.

**Fig. S9** Sanger sequencing chromatograms of prime-edited wheat plants for each target gene in T_0_ generation.

**Fig. S10** Multiplex prime editing by CMPE-ePPEplus in transgenic wheat plants in T_0_ generation.

**Fig. S11** Construct used for multiplex prime editing and detection of transgene integration in the T_1_ generation.

**Fig. S12** Engineered prime editors by fusion of different proteins with ePPE in wheat protoplasts.


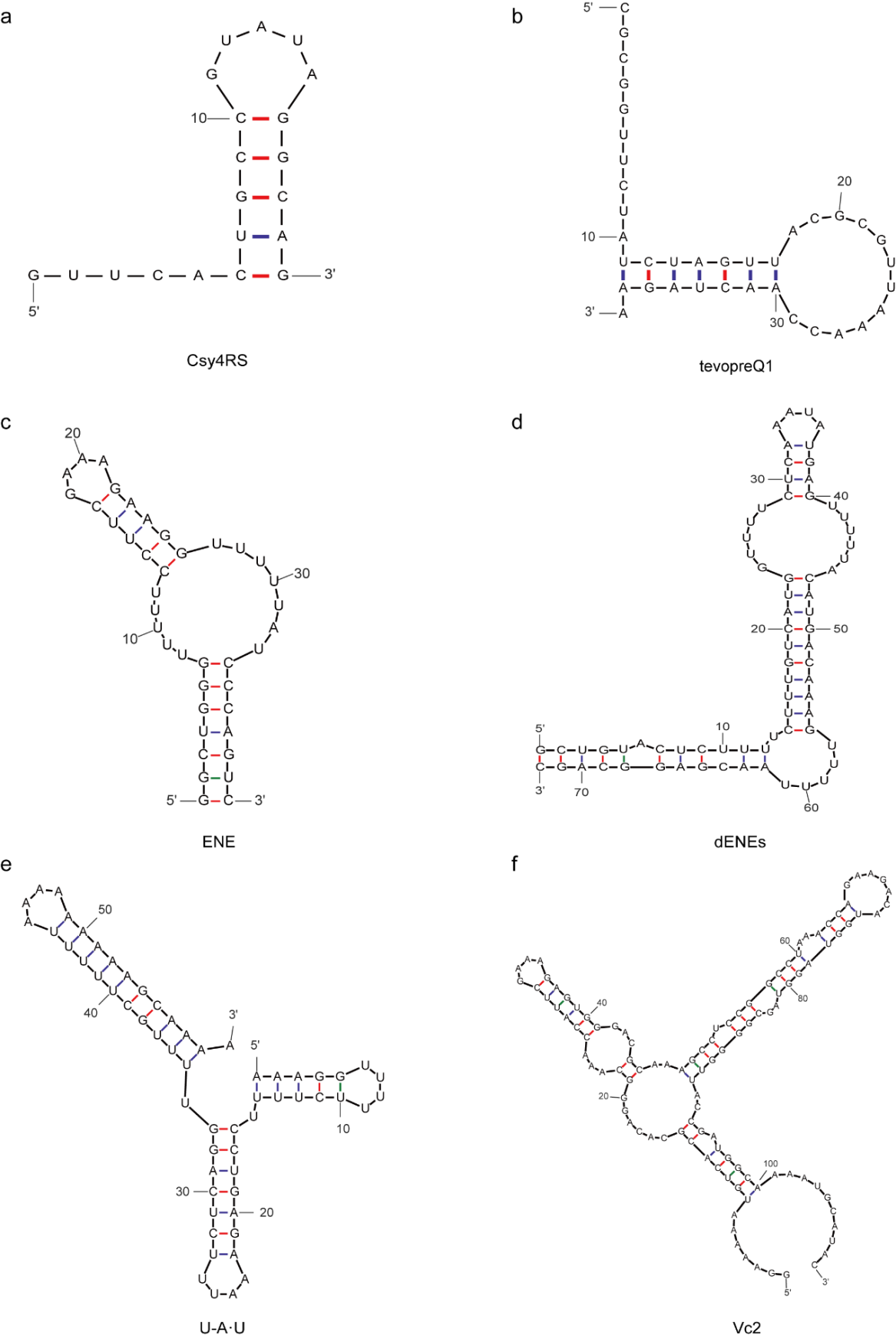


**Fig. S1 The secondary structure of different modifications examined in this study.**

The structures are based on predictions from previously published structural or bioinformatic analyses (<https://swissmodel.expasy.org>). **a-****f** The structure of Csy4 recognition site (Csy4RS) (**a**), tevopreQ1 RNA motif (**b**), elements for nuclear expression (ENE) (**c**), double ENEs (dENEs) (**d**), a triple helix structure (U-A·U) (**e**), and the riboswitch aptamer from *Vibrio cholerae* (Vc2) (**f**).


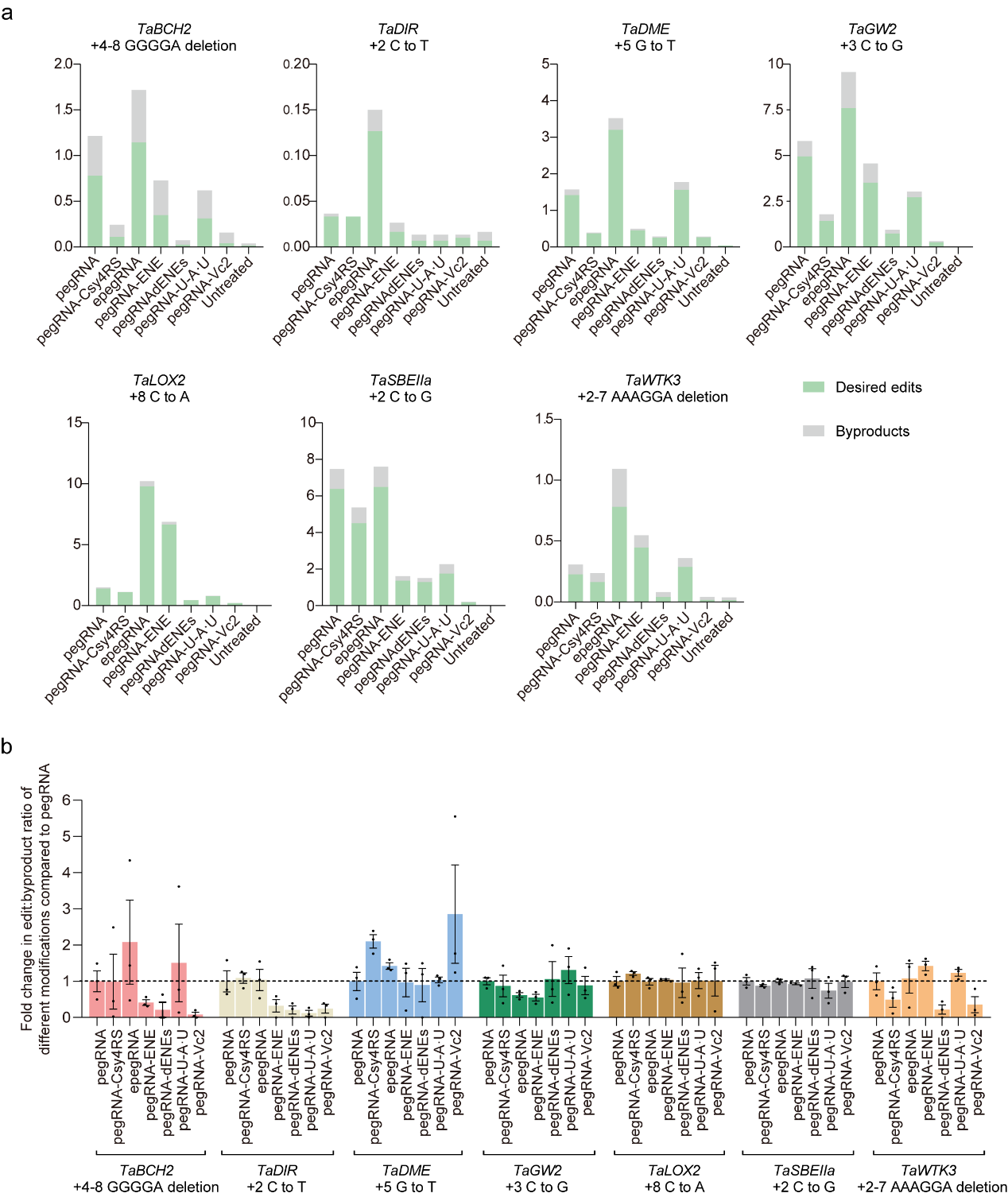


**Fig. S2 Product purity for pegRNAs with different modifications.**

**a** Product purity in prime editing induced by ePPE at seven wheat target sites using pegRNA with different modifications. **b** Fold change in the observed prime editing edit:byproduct ratio for seven wheat target sites. Values were calculated from the data presented in **Fig. 1c**. Frequencies (means ± s.e.m.) were calculated from three independent experiments (*n* = 3).


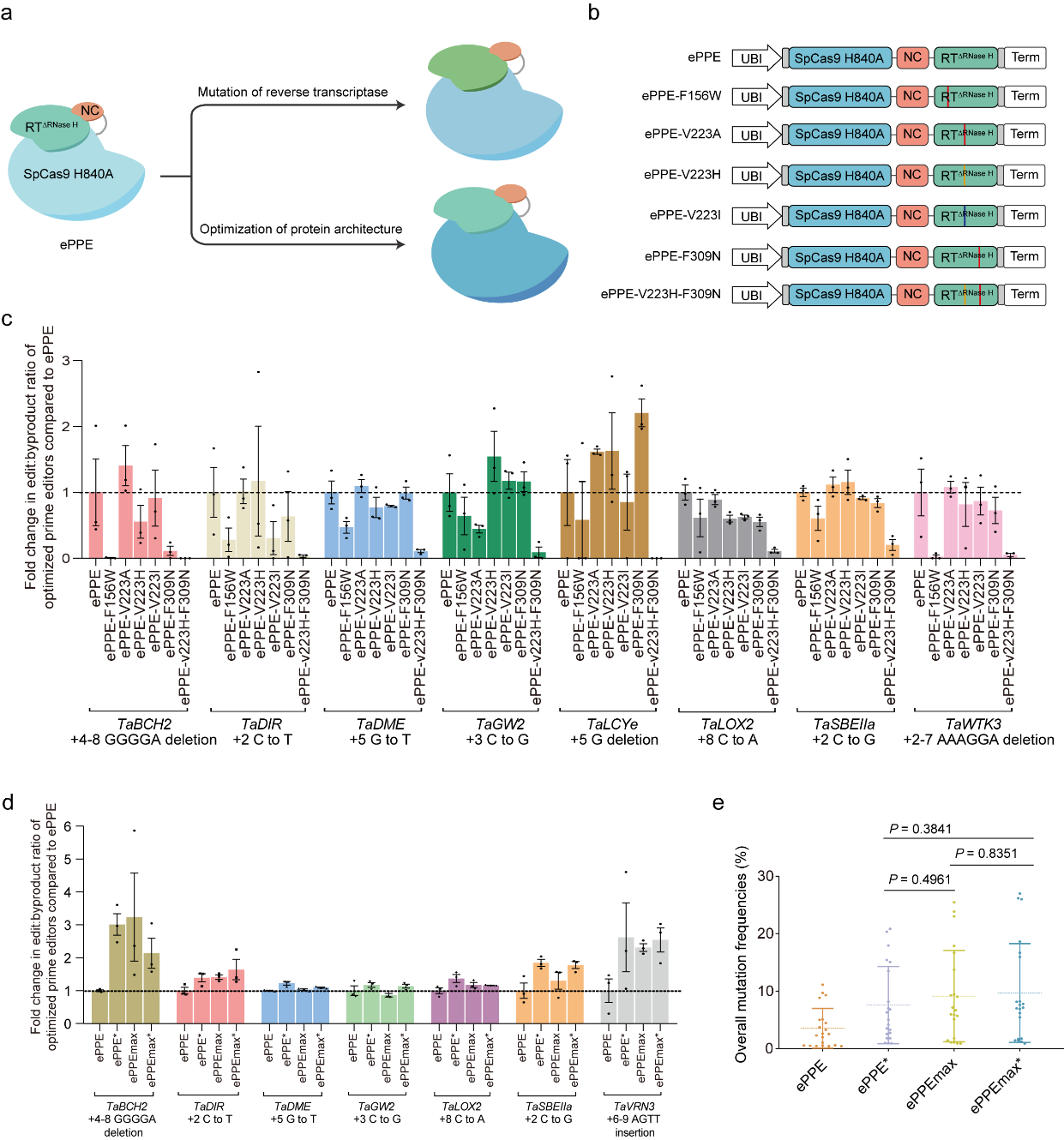


**Fig. S3 The strategies and product purity for optimized prime editors.**

**a** Schematic diagram of two optimization strategies, introducing point mutations in M-MLV RT^∆RNase H^ and optimization of protein architecture. **b** Constructs of different prime editors, ePPE, ePPE-F156W, ePPE-V223A, ePPE-V223H, ePPE-V223I, ePPE-F309N and ePPE-V223H-F309N. **c, d** Fold change in the observed prime editing edit:byproduct ratio for eight wheat target sites by ePPE, ePPE-F156W, ePPE-V223A, ePPE-V223H, ePPE-V223I, ePPE-F309N and ePPE-V223H-F309N (**c**), and for seven wheat target sites by ePPE, ePPE*, ePPEmax and ePPEmax*(**d**) . Values in (**c**) and (**d**) were calculated from the data presented in **Fig. 2b** and **Fig. 2e,** respectively. Frequencies (means ± s.e.m.) were calculated from three independent experiments (*n* = 3).**e** Overall mutation frequencies mediated by ePPE, ePPE*, ePPEmax and ePPEmax*. *P*-values were obtained using the two-tailed Student’s *t*-test.

**
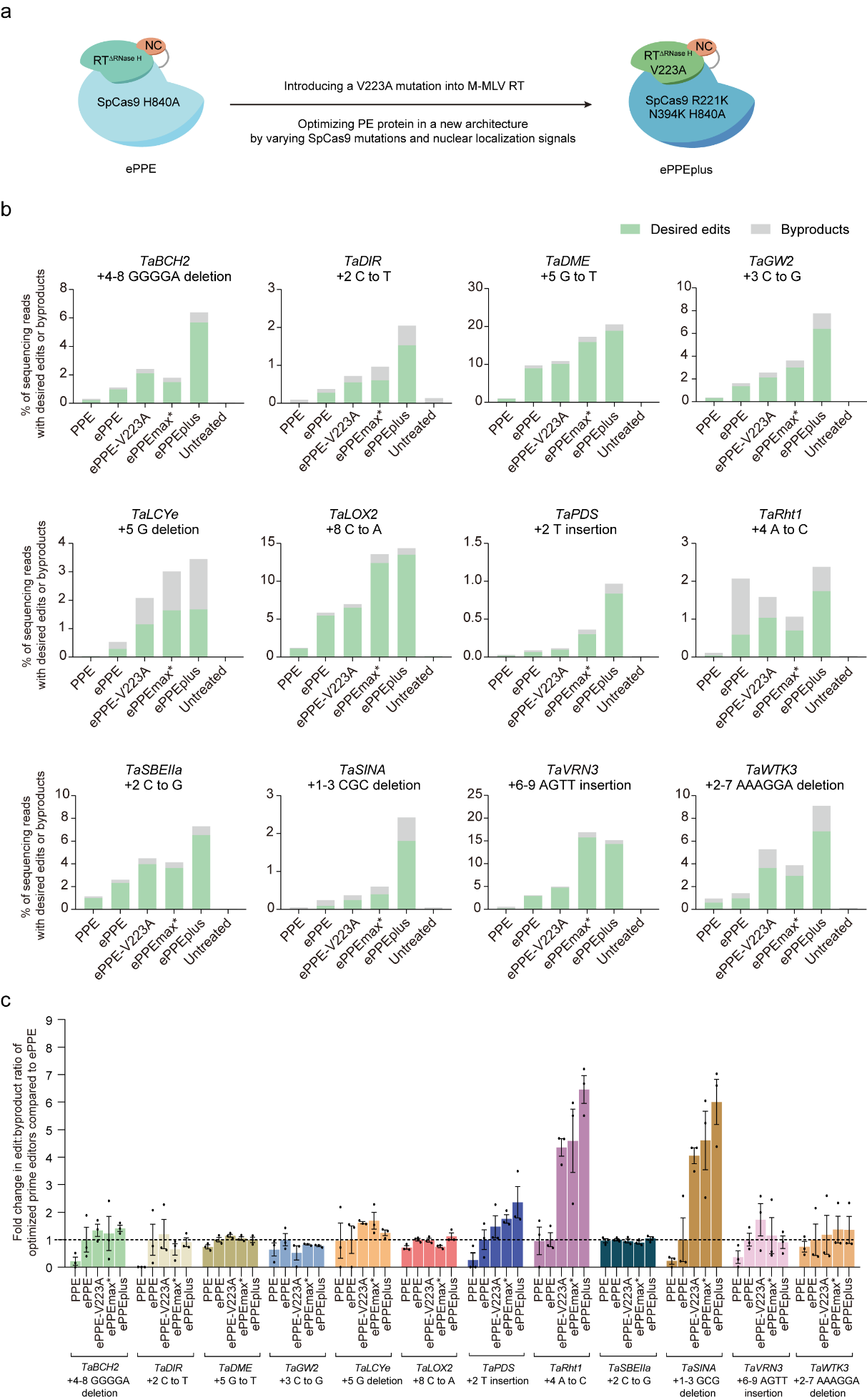
**

**Fig. S4 Product purity for PPE, ePPE, ePPE-V223A, ePPEmax* and ePPEplus.**

**a** Schematic diagram of the ePPEplus by introducing V223A mutation into M-MLV RT^∆RNase H^ under the new architecture by varying SpCas9 mutations and nuclear localization signals. **b** Frequencies of prime editing and undesired byproducts induced by PPE, ePPE, ePPE-V223A, ePPEmax* and ePPEplus at 12 endogenous sites in wheat protoplasts. **c** Fold change in the observed prime editing edit:byproduct ratio. Values were calculated from the data presented in **Fig. 3b**. Frequencies (means ± s.e.m.) were calculated from three independent experiments (*n* = 3).

**
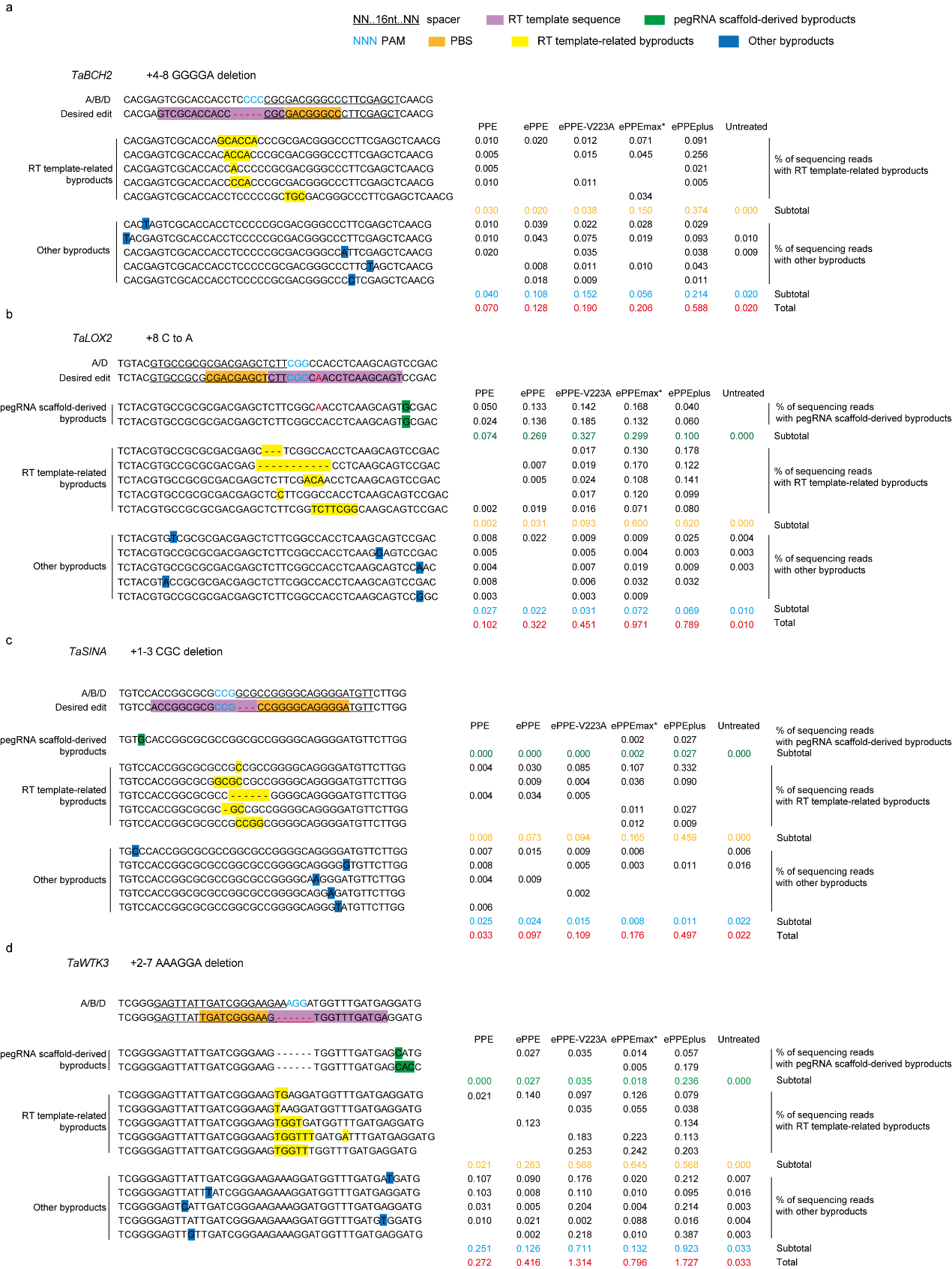
**

**Fig. S5 Mutation type and percentages of byproducts for PPE, ePPE, ePPE-V223A, ePPEmax* and ePPEplus.**

**a-d** Byproducts of prime editing induced by PPE, ePPE, ePPE-V223A, ePPEmax* and ePPEplus at the represent targeted sites *TaBCH2* (**a**), *TaLOX2* (**b**), *TaSINA* (**c**) and *TaWTK3* (**d**). pegRNA scaffold-derived byproducts, RT template-related byproducts (including RT template duplication, insertion, deletion or replacement) and other undesired byproducts (random SNPs at 2-15 bp upstream and downstream of RT template) accompanying the prime editing events were identified. The average frequencies of five most frequent of RT template-related mutations and other byproducts, and the average frequencies of all pegRNA scaffold-derived insertions were calculated from three biologically independent experiments.

**
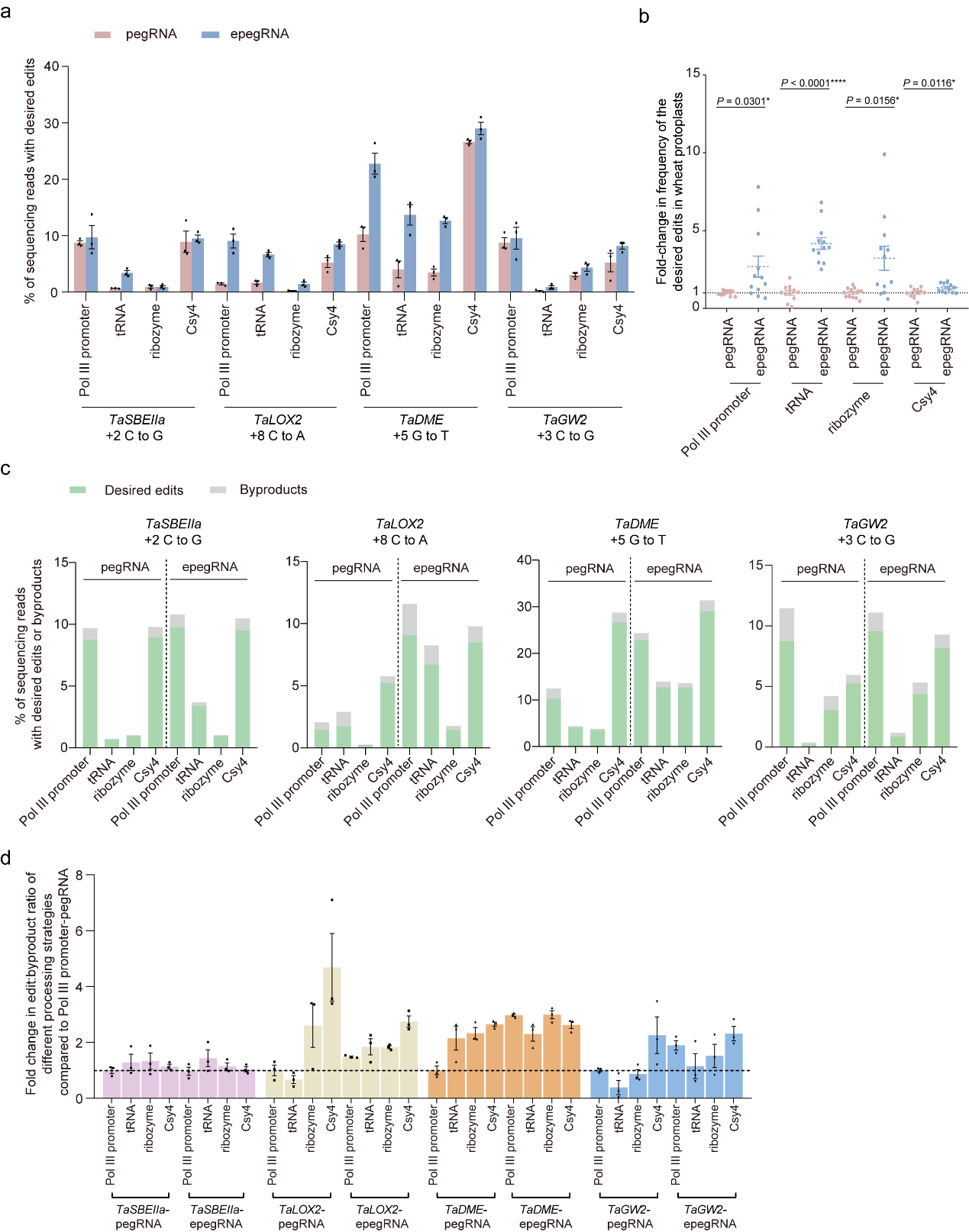
**

**Fig. S6 Comparison of multiple pegRNAs processing strategies in wheat protoplasts.**

**a** Comparison of the prime editing efficiencies induced by Pol III promoter-processing system, tRNA-processing system, ribozyme-processing system and Csy4-processing system with pegRNAs or epegRNAs. **b** Summary of the fold change in prime editing efficiencies for epegRNA compared to ePPE induced by Pol III promoter-processing system, tRNA-processing system, ribozyme-processing system and Csy4-processing system. Values were calculated from the data presented in **Fig.4b**. The average editing frequencies using pegRNA for each target induced by each processing strategy were normalized to 1, and the frequencies using epegRNA for corresponding target were adjusted accordingly. *P*-values were obtained using the two-tailed Student’s *t*-test: **P* < 0.05, ****P* < 0.0001. **c** Frequencies of prime editing and undesired byproducts induced by Pol III promoter-, tRNA-, ribozyme- and Csy4-processing systems. **d** Fold change in the observed prime editing edit:byproduct ratio for four wheat target sites. Values were calculated from the data presented in **Fig. 4b**. Frequencies (means ± s.e.m.) were calculated from three independent experiments (*n* = 3).

**
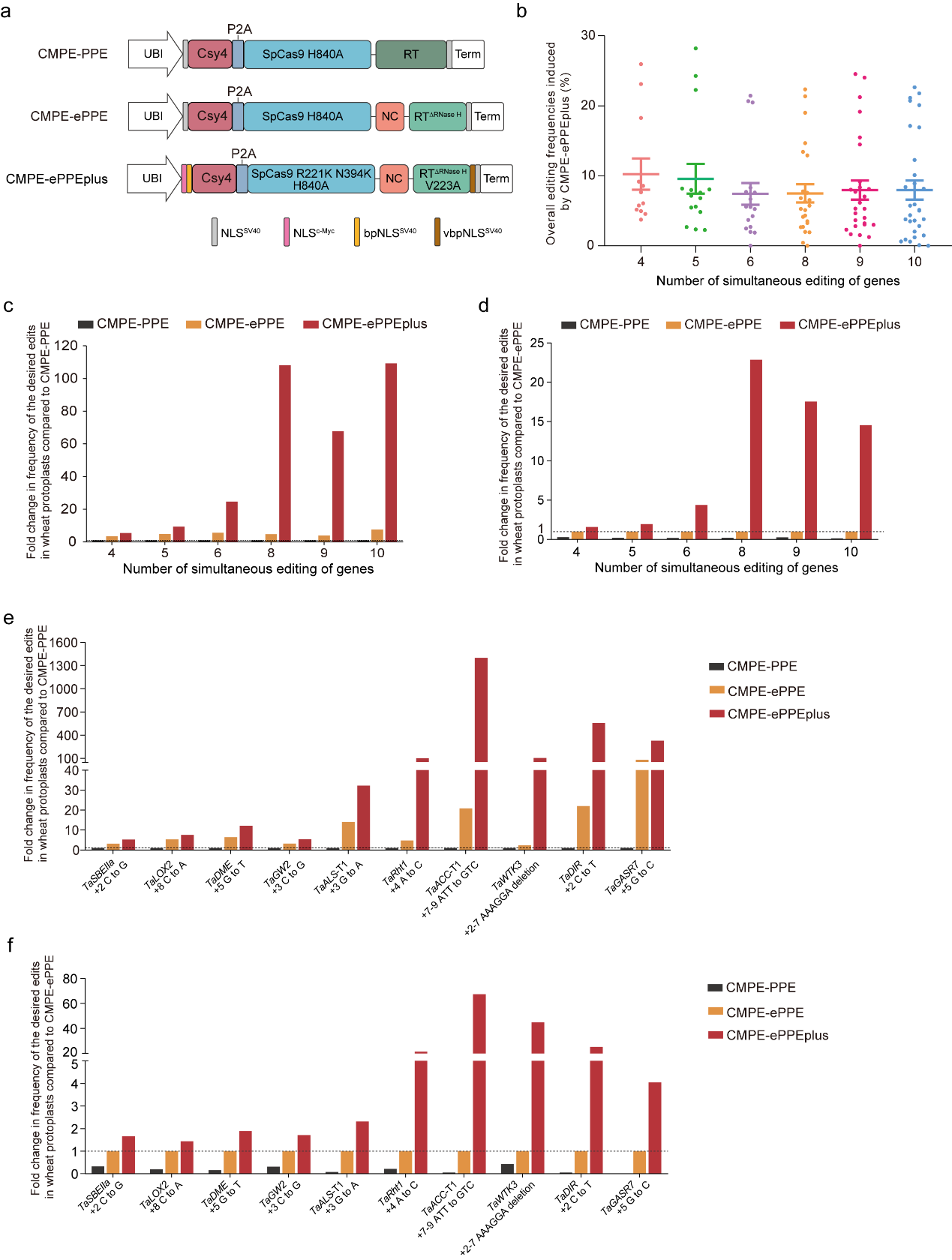
**

**Fig. S7 CMPE-mediated multiplex prime editing in wheat protoplasts.**

**a** Schematic representation of the CMPE-PPE, CMPE-ePPE, CMPE-ePPEplus. Csy4 protein were expressed as P2A fusion. **b** Overall editing frequencies induced by CMPE-ePPEplus for simultaneous editing of four, five, six, eight and nine genes. **c, d** Fold change in frequency of the desired edits for each array of epegRNAs induced by CMPE-ePPE and CMPE-ePPEplus compared to CMPE-PPE (**c**), and CMPE-PPE and CMPE-ePPEplus compared to CMPE-ePPE (**d**). **e, f** Fold change in frequency of the desired edits for individual genes across all multiplex prime editing events induced by CMPE-ePPE and CMPE-ePPEplus compared to CMPE-PPE (**e**), and CMPE-PPE and CMPE-ePPEplus compared to CMPE-ePPE (**f**). The average editing frequencies using CMPE-PPE were normalized to 1 in **c** and **e**, and the frequencies using CMPE-ePPE and CMPE-ePPEplus were adjusted accordingly; and the average editing frequencies using CMPE-ePPE were normalized to 1 in **d** and **f**, and the frequencies using CMPE-PPE and CMPE-ePPEplus were adjusted accordingly. Values were calculated from the data presented in **Fig. 4d**.


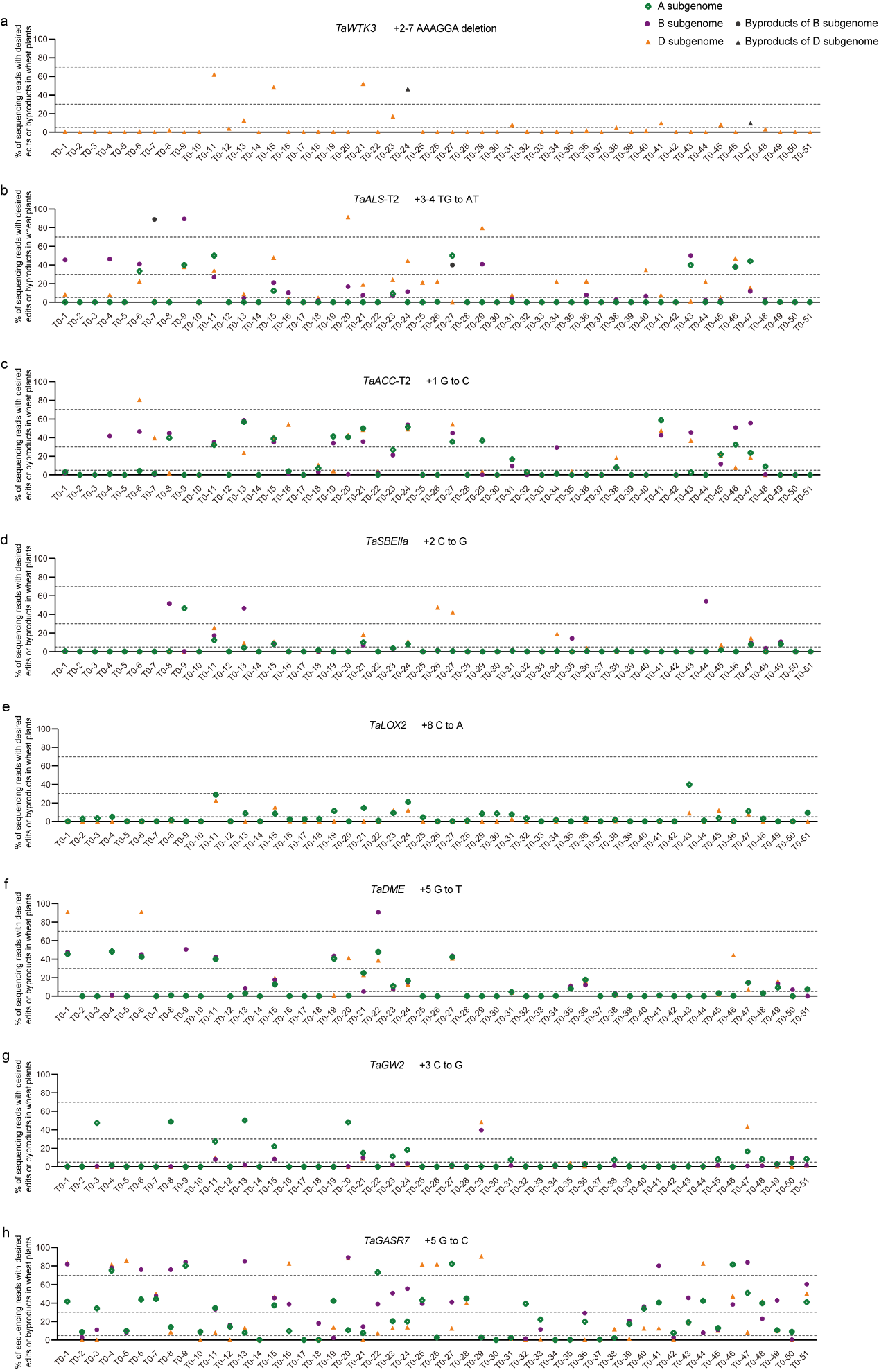


**Fig. S8 Mutation type of prime-edited wheat plants for each target gene** **in T_0_ generation.**

**a-h** Mutation type in A, B and D subgenomes of each plant for *TaWTK3* (**a**), *TaALS*-T2 (**b**), *TaACC-*T2 (**c**), *TaSBEIIa* (**d**)*, TaLOX2* (**e**), *TaDME* (**f**)*, TaGW2* (**g**), and *TaGASR7* (**h**) in T_0_ generation. Mutation efficiencies were examined by NGS with 5% threshold. These edited wheat plants were categorized into five genotypes including homozygous, heterozygous, chimeric, byproducts and wild-type following these criteria: homozygous, mutation frequency ≥ 70% without undesired byproducts; heterozygous, mutation frequency ≥ 30% and ＜ 70% without undesired byproducts; chimeric, mutation frequency ≥ 5% and＜ 30% without undesired byproducts according previous studies (Li et al., 2022 [25]; Pan et al., 2022 [59]); byproducts, when the main mutation type in a homozygous/ heterozygous/chimeric line contains undesired edits, we counted it as byproduct lines; and wild-type, mutation frequency ＜ 5%.

**
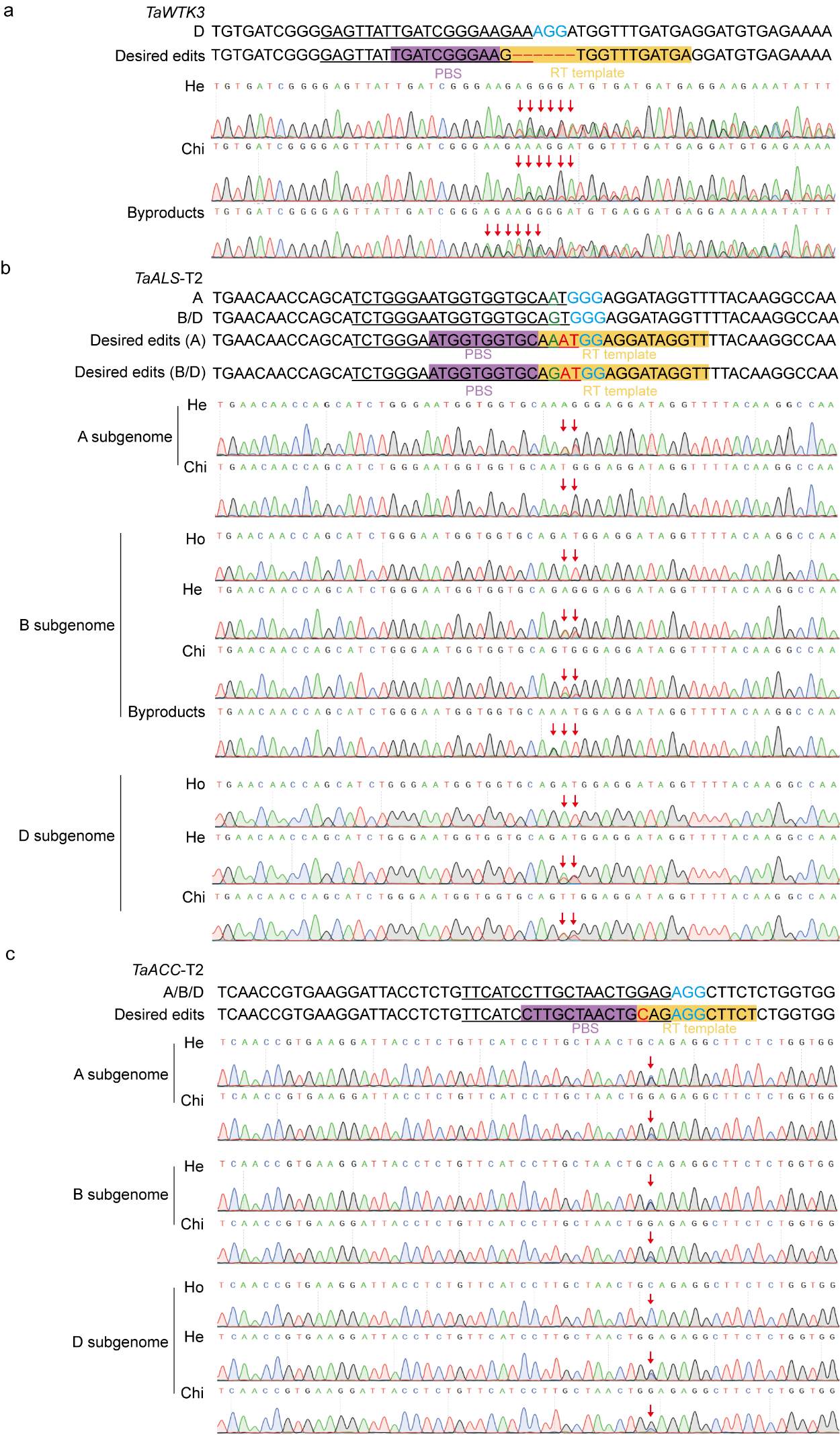

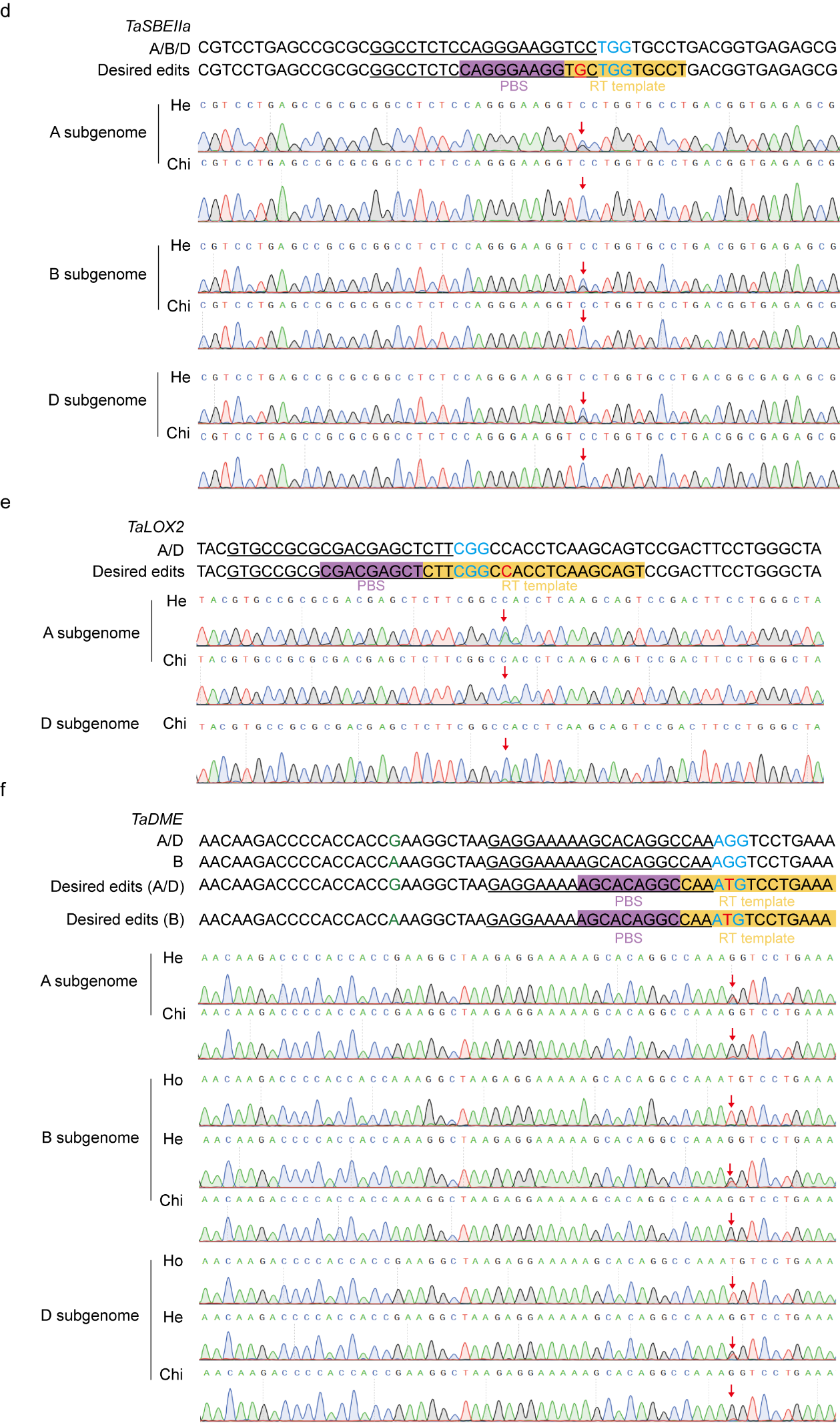

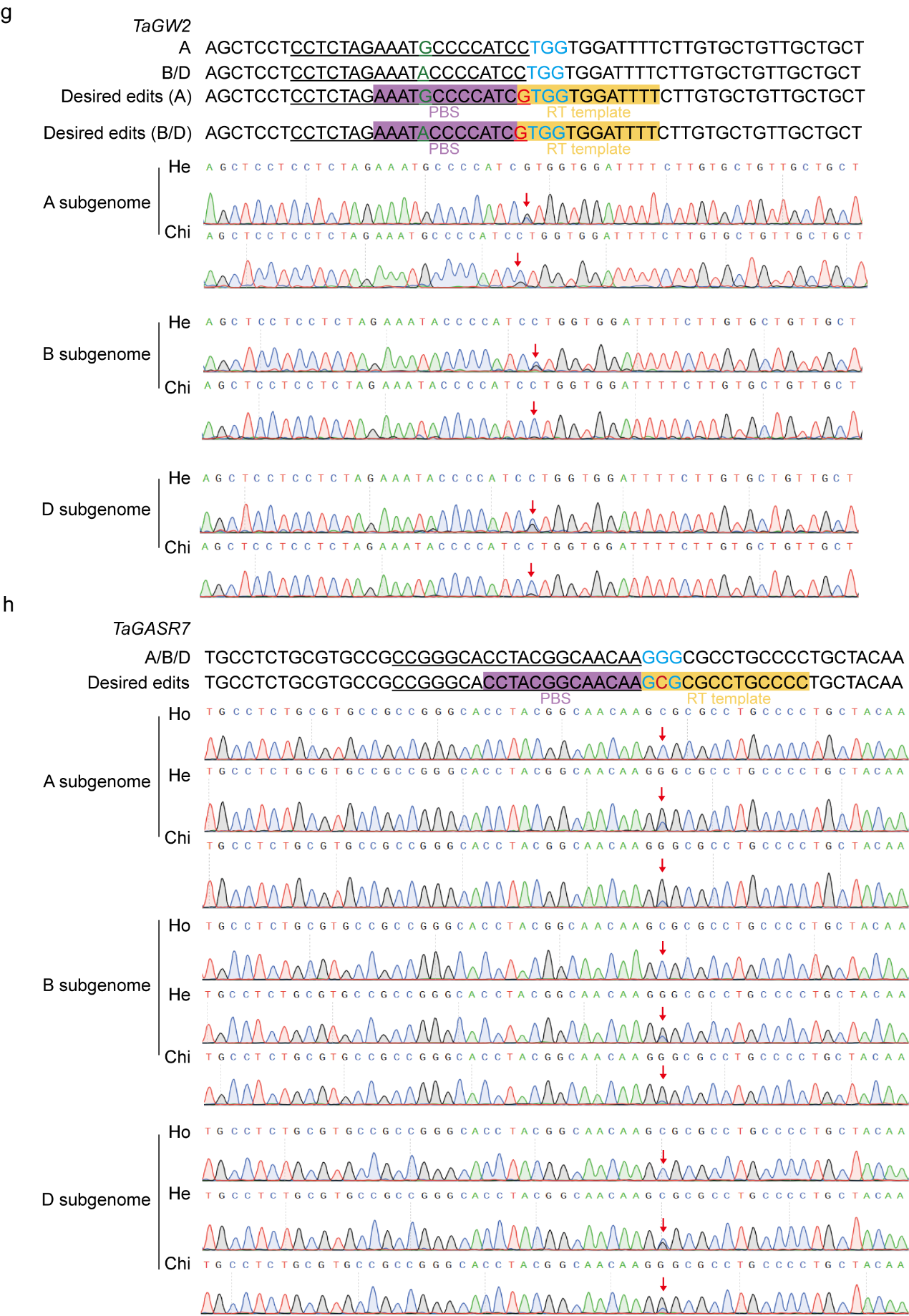
 Fig. S9 Sanger sequencing chromatograms of prime-edited wheat plants in T_0_ generation.**

**a-h** Represented Sanger sequencing chromatograms for *TaWTK3* (**a**), *TaALS*-T2 (**b**), *TaACC-*T2 (**c**), *TaSBEIIa* (**d**)*, TaLOX2* (**e**), *TaDME* (**f**)*, TaGW2* (**g**), *TaGASR7* (**h**). The spacer of pegRNAs is underlined. PBS sequences are highlighted in purple and RT template sequences are highlighted in yellow. The protospacer-adjacent motif (PAM) sequence is highlighted in blue. The SNPs in different subgenomes are highlighted in green. The desired edits are highlighted in red. Homozygous (Ho), Heterozygous (He), Chimeric (Chi), Byproducts, prime editing with undesired edits.


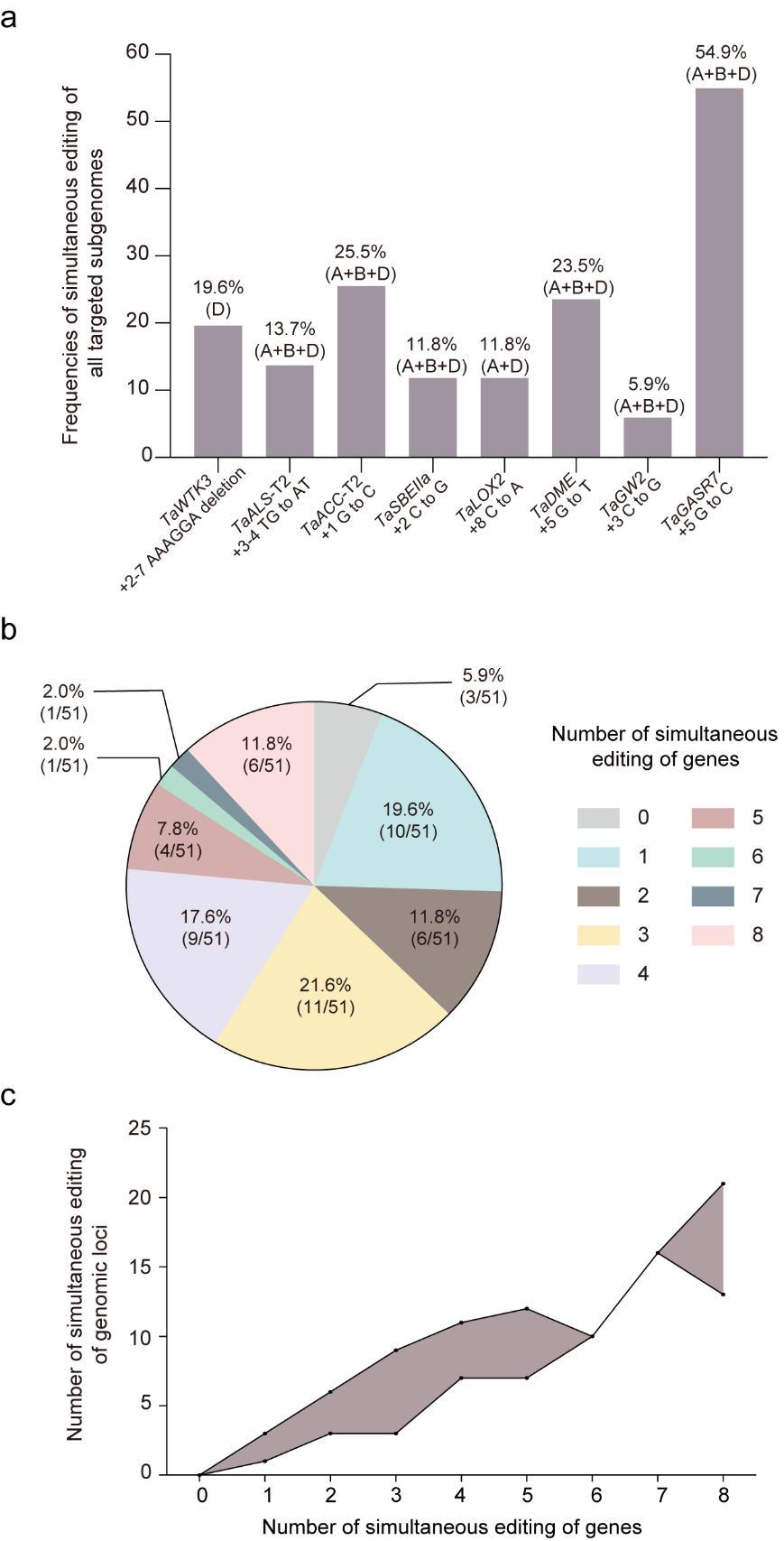


**Fig. S10 Multiplex prime editing by CMPE-ePPEplus in transgenic wheat plants in T_0_ generation.**

**a** Frequencies of each gene with mutations across all targeted subgenomes simultaneously among 51 regenerated plants. **b** Frequencies of simultaneous editing of different numbers of genes among 51 regenerated plants. **c** The range of edited genomic loci when targeting different numbers of genes simultaneously.


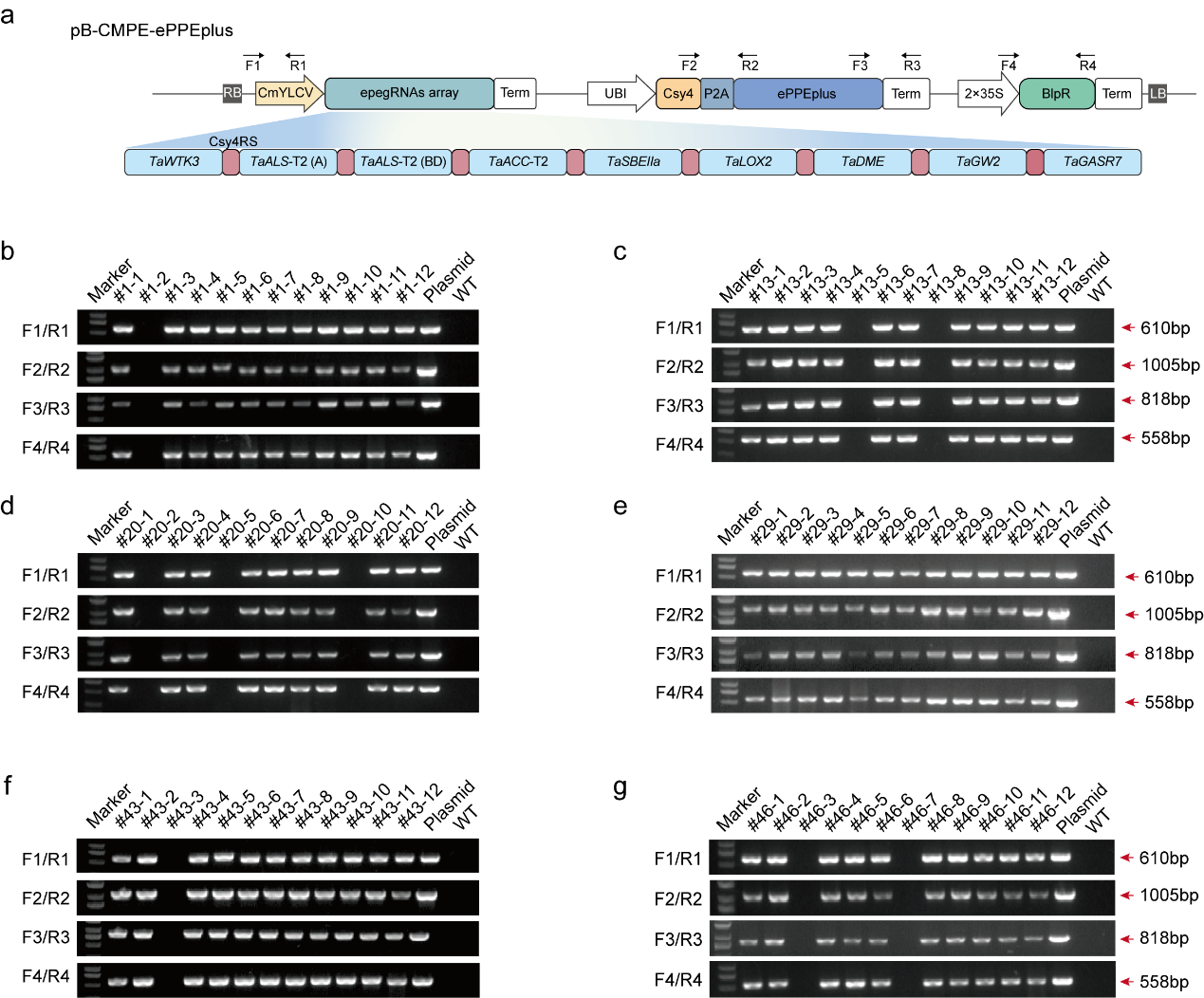


**Fig. S11 Construct used for multiplex prime editing and detection of transgene integration in the T_1_ generation.**

**a** Diagram of the pB-CMPE-ePPEplus vectors used for multiplex prime editing. The positions of the four primer sets (F1/R1, F2/R2, F3/R3 and F4/R4) used for detecting transgene integration are shown. **b-g** Outcome of the tests for transgene integration using four primer sets for twelve represented mutant plants (1 to 12) for six T_0_ mutant lines including T_0_-1 (**b**), T_0_-13 (**c**), T_0_-20 (**d**), T_0_-29 (**e**), T_0_-43 (**f**) and T_0_-46 (**g**). None of the four primer sets yielded the expected PCR amplicon in related plant, indicating that they were transgene-free. The negative control for the tests was performed using the genomic DNA extracted from wild type wheat plants. The positive control for the tests was conducted with the plasmid DNA of pB-CMPE-ePPEplus, with the anticipated products being indicated by red arrowheads.


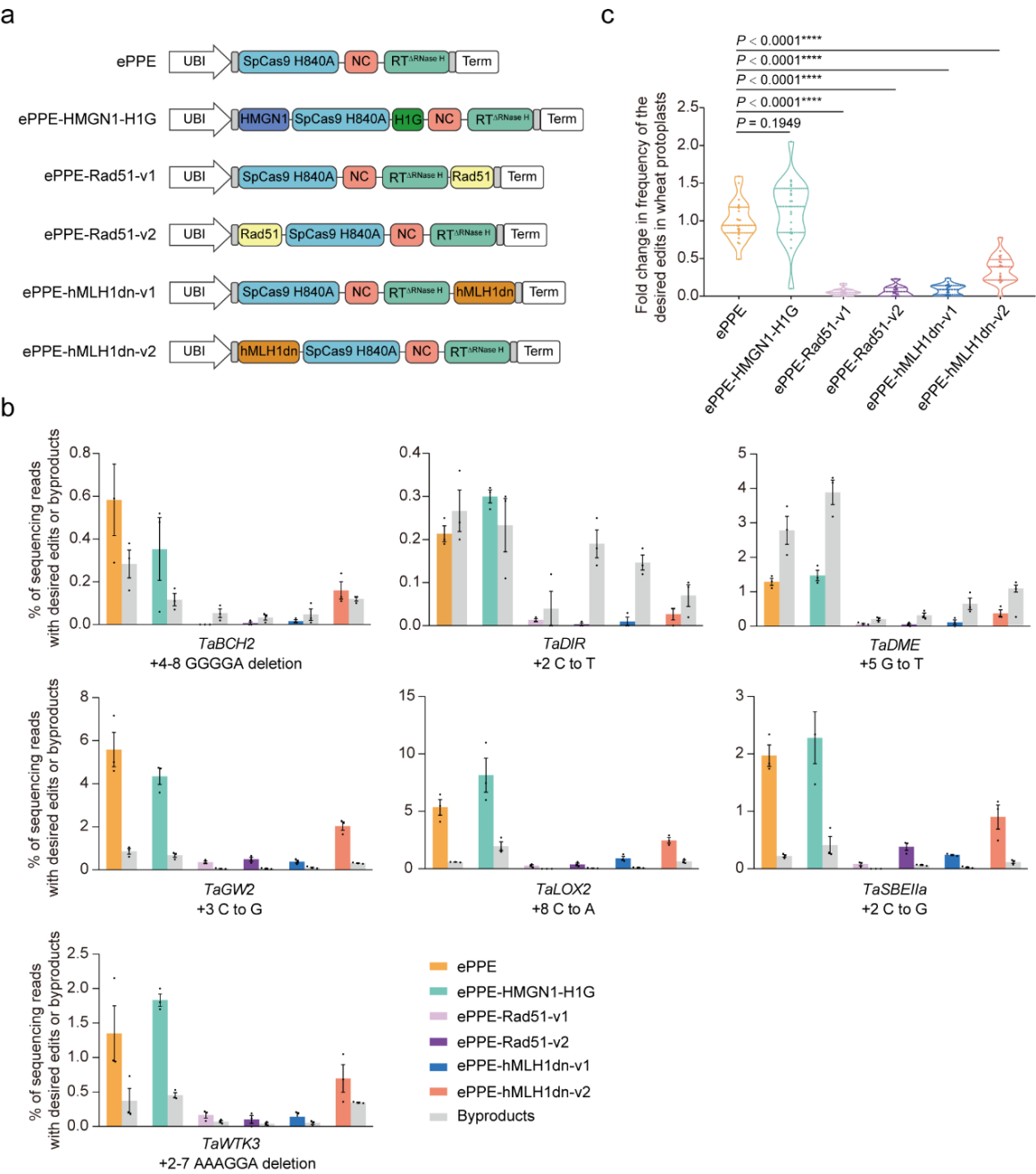


**Fig. S12 Engineered prime editors by fusion of different proteins with ePPE in wheat protoplasts.**

**a** Schematic representation of the ePPE, ePPE-HMGN1-H1G, ePPE-Rad51-v1, ePPE-Rad51-v2, ePPE-hMLH1dn-v1 and ePPE-hMLH1dn-v2. v1, fusion of Rad51 or hMLH1dn at the C terminus of RT; v2, fusion of Rad51 and hMLH1dn at the N terminus of nCas9. **b** Frequencies of prime editing and byproducts induced by ePPE, and other engineered prime editors at seven wheat target sites. Frequencies (mean ± s.e.m.) were calculated from three independent experiments (*n* = 3). **c** Summary of the fold change in prime editing efficiencies for ePPE and other engineered prime editors. The editing frequencies using ePPE for each target were normalized to 1, and the frequencies using other engineered prime editors for each target were adjusted accordingly. *P* values were obtained using the two-tailed Student’s *t*-test: *****P* < 0.0001.
