# Supplementary material for: Efficient and versatile multiplex prime editing in hexaploid wheat
Source: Genome Biol. 2023 Jun 29;24:156. doi: 10.1186/s13059-023-02990-1 (PMC10308706; doi:10.1186/s13059-023-02990-1)
Supplement: Supplementary file 3 — Additional file 3: Sequences S1. Complete sequences of Csy4RS, tevopreQ1, ENE, dENEs, U-A·U and Vc2 modifications in this study. Sequences S2. Complete coding sequences of CmYLCV promoter, Pol III promoter, tRNA and ribozyme in this study. Sequences S3. The full plasmid sequences of ePPE-V223A, ePPEmax*, ePPEplus, CMPE-ePPEplus, pUC57-CmYLCV and pB-CMPE-ePPEplus in this study. Sequences S4. Complete coding sequences of PPE, ePPE, ePPE-F156W, ePPE-V223H, ePPE-V223I, ePPE-V223H, ePPE-V223H-F309N, ePPE*, ePPEmax, ePPE-HMGN1-H1G, ePPE-Rad51-v1, ePPE-Rad51-v2, ePPE-hMLH1dn-v1, ePPE-hMLH1dn-v2, CMPE-PPE and CMPE-ePPE in this study. [file 13059_2023_2990_MOESM3_ESM.docx]

**Additional file 3**

**Sequences S1.** Complete sequences of Csy4RS, tevopreQ1, ENE, dENEs, U-A·U and Vc2 modifications in this study.

**Sequences S2.** Complete coding sequences of CmYLCV promoter, Pol III promoter (TaU3/TaU6), tRNA and ribozyme in this study.

**Sequences S3.** The full plasmid sequences of ePPE-V223A, ePPEmax*, ePPEplus, CMPE-ePPEplus, pUC57-CmYLCV and pB-CMPE-ePPEplus in this study.

**Sequences S4.** Complete coding sequences of PPE, ePPE, ePPE-F156W, ePPE-V223H, ePPE-V223I, ePPE-V223H, ePPE-V223H-F309N, ePPE*, ePPEmax, ePPE-HMGN1-H1G, ePPE-Rad51-v1, ePPE-Rad51-v2, ePPE-hMLH1dn-v1, ePPE-hMLH1dn-v2, CMPE-PPE and CMPE-ePPE in this study.

**Sequences S1.** Complete sequences of Csy4RS, tevopreQ1, ENE, dENEs, U-A·U and Vc2 modifications in this study.

Csy4 recognition site (Csy4RS)

GTTCACTGCCGTATAGGCAG

tevopreQ1

CGCGGTTCTATCTAGTTACGCGTTAAACCAACTAGAA

ENE

GGCTGGGTTTTTCCTTCGAAAGAAGGTTTTTATCCCAGTC

dENEs

GCTGTACTCTTTTCTTTGTCATGGTTTTCTCAAATATGAGTTTTTACATGACAAAGTTTTTAACGAGGCAGC

U-A·U

AAAGGTTTTTCTTTTCCTGAGAAATTTCTCAGGTTTTGCTTTTTAAAAAAAAAGCAAAA

Vc2

GGAAAAATGTCACGCACAGGGCAAACCATTCGAAAGAGTGGGACGCAAAGCCTCCGGCCTAAACCAGAAGACATGGTAGGTAGCGGGGTTACCGATGGCAAAATGCATAC

**Sequences S2.** Complete coding sequences of CmYLCV promoter, Pol III promoter (TaU3/TaU6), tRNA and ribozyme in this study.

**CmYLCV promoter**

TGGCAGACATACTGTCCCACAAATGAAGATGGAATCTGTAAAAGAAAACGCGTGAAATAATGCGTCTGACAAAGGTTAGGTCGGCTGCCTTTAATCAATACCAAAGTGGTCCCTACCACGATGGAAAAACTGTGCAGTCGGTTTGGCTTTTTCTGACGAACAAATAAGATTCGTGGCCGACAGGTGGGGGTCCACCATGTGAAGGCATCTTCAGACTCCAATAATGGAGCAATGACGTAAGGGCTTACGAAATAAGTAAGGGTAGTTTGGGAAATGTCCACTCACCCGTCAGTCTATAAATACTTAGCCCCTCCCTCATTGTTAAGGGAGCAAAATCTCAGAGAGATAGTCCTAGAGAGAGAAAGAGAGCAAGTAGCCTAGAAGTAGTCAAGGCGGCGAAGTATTCAGGCACGTGGCCAGGAAGAAGAAAAGCCAAGACGACGAAAACAGGTAAGAGCTAAGCTT

**TaU3 promoter**

CATGAATCCAAACCACACGGAGTTCAAATTCCCACAGATTAAGGCTCGTCCGTCGCACAAGGTAATGTGTGAATATTATATCTGTCGTGCAAAATTGCCTGGCCTGCACAATTGCTGTTATAGTTGGCGGCAGGGAGAGTTTTAACATTGACTAGCGTGCTGATAATTTGTGAGAAATAATAATTGACAAGTAGATACTGACATTTGAGAAGAGCTTCTGAACTGTTATTAGTAACAAAAATGGAAAGCTGATGCACGGAAAAAGGAAAGAAAAAGCCATACTTTTTTTTAGGTAGGAAAAGAAAAAGCCATACGAGACTGATGTCTCTCAGATGGGCCGGGATCTGTCTATCTAGCAGGCAGCAGCCCACCAACCTCACGGGCCAGCAATTACGAGTCCTTCTAAAAGCTCCCGCCGAGGGGCGCTGGCGCTGCTGTGCAGCAGCACGTCTAACATTAGTCCCACCTCGCCAGTTTACAGGGAGCAGAACCAGCTTATAAGCGGAGGCGCGGCACCAAGAAGCG

**TaU6 promoter**

GACCAAGCCCGTTATTCTGACAGTTCTGGTGCTCAACACATTTATATTTATCAAGGAGCACATTGTTACTCACTGCTAGGAGGGAATCGAACTAGGAATATTGATCAGAGGAACTACGAGAGAGCTGAAGATAACTGCCCTCTAGCTCTCACTGATCTGGGCGCATAGTGAGATGCAGCCCACGTGAGTTCAGCAACGGTCTAGCGCTGGGCTTTTAGGCCCGCATGATCGGGCTTTGTCGGGTGGTCGACGTGTTCACGATTGGGGAGAGCAACGCAGCAGTTCCTCTTAGTTTAGTCCCACCTCGCCTGTCCAGCAGAGTTCTGACCGGTTTATAAACTCGCTTGCTGCATCAGACTTG

**tRNA**

AACAAAGCACCAGTGGTCTAGTGGTAGAATAGTACCCTGCCACGGTACAGACCCGGGTTCGATTCCCGGCTGGTGCA

**ribozyme (HH-HDV)**

HH

CTGATGAGTCCGTGAGGACGAAACGAGTAAGCTCGTC

HDV

GGCCGGCATGGTCCCAGCCTCCTCGCTGGCGCCGGCTGGGCAACATGCTTCGGCATGGCGAATGGGAC

**Sequences S3.** The full plasmid sequences of ePPE-V223A, ePPEmax*, ePPEplus, CMPE-ePPEplus pUC57-CmYLCV and pB-CMPE-ePPEplus in this study. The NLS^SV40^, NLS^c-myc^, bpNLS^SV40^, vbpNLS^SV40^, nCas9 (H840A), NC protein, Csy4 endoribonuclease, M-MLV RT^∆RNase H^ Csy4RS, esgRNA scaffold and tevopreQ1 motif are highlighted in purple, pink, brown, dark blue, blue, green, dark green, orange, dark purple, gray and red, respectively. The introduced R221K/ N394K substitutions into nCas9 (H840A) and V223A substitution into M-MLV RT^∆RNase H^ are shown in lowercase and bold. The P2A is underlined. The *Ubi* promoter, CmYLCV promoter, CaMV terminator and E9 terminator are highlighted in gray, blue, dark yellow and pink, respectively.

**ePPE-V223A**

GAGCTCGGTACCTGACCCGGTCGTGCCCCTCTCTAGAGATAATGAGCATTGCATGTCTAAGTTATAAAAAATTACCACATATTTTTTTTGTCACACTTGTTTGAAGTGCAGTTTATCTATCTTTATACATATATTTAAACTTTACTCTACGAATAATATAATCTATAGTACTACAATAATATCAGTGTTTTAGAGAATCATATAAATGAACAGTTAGACATGGTCTAAAGGACAATTGAGTATTTTGACAACAGGACTCTACAGTTTTATCTTTTTAGTGTGCATGTGTTCTCCTTTTTTTTTGCAAATAGCTTCACCTATATAATACTTCATCCATTTTATTAGTACATCCATTTAGGGTTTAGGGTTAATGGTTTTTATAGACTAATTTTTTTAGTACATCTATTTTATTCTATTTTAGCCTCTAAATTAAGAAAACTAAAACTCTATTTTAGTTTTTTTATTTAATAATTTAGATATAAAATAGAATAAAATAAAGTGACTAAAAATTAAACAAATACCCTTTAAGAAATTAAAAAAACTAAGGAAACATTTTTCTTGTTTCGAGTAGATAATGCCAGCCTGTTAAACGCCGTCGACGAGTCTAACGGACACCAACCAGCGAACCAGCAGCGTCGCGTCGGGCCAAGCGAAGCAGACGGCACGGCATCTCTGTCGCTGCCTCTGGACCCCTCTCGATCGAGAGTTCCGCTCCACCGTTGGACTTGCTCCGCTGTCGGCATCCAGAAATTGCGTGGCGGAGCGGCAGACGTGAGCCGGCACGGCAGGCGGCCTCCTCCTCCTCTCACGGCACCGGCAGCTACGGGGGATTCCTTTCCCACCGCTCCTTCGCTTTCCCTTCCTCGCCCGCCGTAATAAATAGACACCCCCTCCACACCCTCTTTCCCCAACCTCGTGTTGTTCGGAGCGCACACACACACAACCAGATCTCCCCCAAATCCACCCGTCGGCACCTCCGCTTCAAGGTACGCCGCTCGTCCTCCCCCCCCCCCCCTCTCTACCTTCTCTAGATCGGCGTTCCGGTCCATGGTTAGGGCCCGGTAGTTCTACTTCTGTTCATGTTTGTGTTAGATCCGTGTTTGTGTTAGATCCGTGCTGCTAGCGTTCGTACACGGATGCGACCTGTACGTCAGACACGTTCTGATTGCTAACTTGCCAGTGTTTCTCTTTGGGGAATCCTGGGATGGCTCTAGCCGTTCCGCAGACGGGATCGATTTCATGATTTTTTTTGTTTCGTTGCATAGGGTTTGGTTTGCCCTTTTCCTTTATTTCAATATATGCCGTGCACTTGTTTGTCGGGTCATCTTTTCATGCTTTTTTTTGTCTTGGTTGTGATGATGTGGTCTGGTTGGGCGGTCGTTCTAGATCGGAGTAGAATTAATTCTGTTTCAAACTACCTGGTGGATTTATTAATTTTGGATCTGTATGTGTGTGCCATACATATTCATAGTTACGAATTGAAGATGATGGATGGAAATATCGATCTAGGATAGGTATACATGTTGATGCGGGTTTTACTGATGCATATACAGAGATGCTTTTTGTTCGCTTGGTTGTGATGATGTGGTGTGGTTGGGCGGTCGTTCATTCGTTCTAGATCGGAGTAGAATACTGTTTCAAACTACCTGGTGTATTTATTAATTTTGGAACTGTATGTGTGTGTCATACATCTTCATAGTTACGAGTTTAAGATGGATGGAAATATCGATCTAGGATAGGTATACATGTTGATGTGGGTTTTACTGATGCATATACATGATGGCATATGCAGCATCTATTCATATGCTCTAACCTTGAGTACCTATCTATTATAATAAACAAGTATGTTTTATAATTATTTTGATCTTGATATACTTGGATGATGGCATATGCAGCAGCTATATGTGGATTTTTTTAGCCCTGCCTTCATACGCTATTTATTTGCTTGGTACTGTTTCTTTTGTCGATGCTCACCCTGTTGTTTGGTGTTACTTCTGCAAAGCTTATGCCTAAGAAAAAGAGAAAAGTGGACAAGAAGTACTCGATCGGCCTCGATATTGGGACTAACTCTGTTGGCTGGGCCGTGATCACCGACGAGTACAAGGTGCCCTCAAAGAAGTTCAAGGTCCTGGGCAACACCGATCGGCATTCCATCAAGAAGAATCTCATTGGCGCTCTCCTGTTCGACAGCGGCGAGACGGCTGAGGCTACGCGGCTCAAGCGCACCGCCCGCAGGCGGTACACGCGCAGGAAGAATCGCATCTGCTACCTGCAGGAGATTTTCTCCAACGAGATGGCGAAGGTTGACGATTCTTTCTTCCACAGGCTGGAGGAGTCATTCCTCGTGGAGGAGGATAAGAAGCACGAGCGGCATCCAATCTTCGGCAACATTGTCGACGAGGTTGCCTACCACGAGAAGTACCCTACGATCTACCATCTGCGGAAGAAGCTCGTGGACTCCACAGATAAGGCGGACCTCCGCCTGATCTACCTCGCTCTGGCCCACATGATTAAGTTCAGGGGCCATTTCCTGATCGAGGGGGATCTCAACCCGGACAATAGCGATGTTGACAAGCTGTTCATCCAGCTCGTGCAGACGTACAACCAGCTCTTCGAGGAGAACCCCATTAATGCGTCAGGCGTCGACGCGAAGGCTATCCTGTCCGCTAGGCTCTCGAAGTCTCGGCGCCTCGAGAACCTGATCGCCCAGCTGCCGGGCGAGAAGAAGAACGGCCTGTTCGGGAATCTCATTGCGCTCAGCCTGGGGCTCACGCCCAACTTCAAGTCGAATTTCGATCTCGCTGAGGACGCCAAGCTGCAGCTCTCCAAGGACACATACGACGATGACCTGGATAACCTCCTGGCCCAGATCGGCGATCAGTACGCGGACCTGTTCCTCGCTGCCAAGAATCTGTCGGACGCCATCCTCCTGTCTGATATTCTCAGGGTGAACACCGAGATTACGAAGGCTCCGCTCTCAGCCTCCATGATCAAGCGCTACGACGAGCACCATCAGGATCTGACCCTCCTGAAGGCGCTGGTCAGGCAGCAGCTCCCCGAGAAGTACAAGGAGATCTTCTTCGATCAGTCGAAGAACGGCTACGCTGGGTACATTGACGGCGGGGCCTCTCAGGAGGAGTTCTACAAGTTCATCAAGCCGATTCTGGAGAAGATGGACGGCACGGAGGAGCTGCTGGTGAAGCTCAATCGCGAGGACCTCCTGAGGAAGCAGCGGACATTCGATAACGGCAGCATCCCACACCAGATTCATCTCGGGGAGCTGCACGCTATCCTGAGGAGGCAGGAGGACTTCTACCCTTTCCTCAAGGATAACCGCGAGAAGATCGAGAAGATTCTGACTTTCAGGATCCCGTACTACGTCGGCCCACTCGCTAGGGGCAACTCCCGCTTCGCTTGGATGACCCGCAAGTCAGAGGAGACGATCACGCCGTGGAACTTCGAGGAGGTGGTCGACAAGGGCGCTAGCGCTCAGTCGTTCATCGAGAGGATGACGAATTTCGACAAGAACCTGCCAAATGAGAAGGTGCTCCCTAAGCACTCGCTCCTGTACGAGTACTTCACAGTCTACAACGAGCTGACTAAGGTGAAGTATGTGACCGAGGGCATGAGGAAGCCGGCTTTCCTGTCTGGGGAGCAGAAGAAGGCCATCGTGGACCTCCTGTTCAAGACCAACCGGAAGGTCACGGTTAAGCAGCTCAAGGAGGACTACTTCAAGAAGATTGAGTGCTTCGATTCGGTCGAGATCTCTGGCGTTGAGGACCGCTTCAACGCCTCCCTGGGGACCTACCACGATCTCCTGAAGATCATTAAGGATAAGGACTTCCTGGACAACGAGGAGAATGAGGATATCCTCGAGGACATTGTGCTGACACTCACTCTGTTCGAGGACCGGGAGATGATCGAGGAGCGCCTGAAGACTTACGCCCATCTCTTCGATGACAAGGTCATGAAGCAGCTCAAGAGGAGGAGGTACACCGGCTGGGGGAGGCTGAGCAGGAAGCTCATCAACGGCATTCGGGACAAGCAGTCCGGGAAGACGATCCTCGACTTCCTGAAGAGCGATGGCTTCGCGAACCGCAATTTCATGCAGCTGATTCACGATGACAGCCTCACATTCAAGGAGGATATCCAGAAGGCTCAGGTGAGCGGCCAGGGGGACTCGCTGCACGAGCATATCGCGAACCTCGCTGGCTCGCCAGCTATCAAGAAGGGGATTCTGCAGACCGTGAAGGTTGTGGACGAGCTGGTGAAGGTCATGGGCAGGCACAAGCCTGAGAACATCGTCATTGAGATGGCCCGGGAGAATCAGACCACGCAGAAGGGCCAGAAGAACTCACGCGAGAGGATGAAGAGGATCGAGGAGGGCATTAAGGAGCTGGGGTCCCAGATCCTCAAGGAGCACCCGGTGGAGAACACGCAGCTGCAGAATGAGAAGCTCTACCTGTACTACCTCCAGAATGGCCGCGATATGTATGTGGACCAGGAGCTGGATATTAACAGGCTCAGCGATTACGACGTCGATGCCATCGTTCCACAGTCATTCCTGAAGGATGACTCCATTGACAACAAGGTCCTCACCAGGTCGGACAAGAACCGGGGCAAGTCTGATAATGTTCCTTCAGAGGAGGTCGTTAAGAAGATGAAGAACTACTGGCGCCAGCTCCTGAATGCCAAGCTGATCACGCAGCGGAAGTTCGATAACCTCACAAAGGCTGAGAGGGGCGGGCTCTCTGAGCTGGACAAGGCGGGCTTCATCAAGAGGCAGCTGGTCGAGACACGGCAGATCACTAAGCACGTTGCGCAGATTCTCGACTCACGGATGAACACTAAGTACGATGAGAATGACAAGCTGATCCGCGAGGTGAAGGTCATCACCCTGAAGTCAAAGCTCGTCTCCGACTTCAGGAAGGATTTCCAGTTCTACAAGGTTCGGGAGATCAACAATTACCACCATGCCCATGACGCGTACCTGAACGCGGTGGTCGGCACAGCTCTGATCAAGAAGTACCCAAAGCTCGAGAGCGAGTTCGTGTACGGGGACTACAAGGTTTACGATGTGAGGAAGATGATCGCCAAGTCGGAGCAGGAGATTGGCAAGGCTACCGCCAAGTACTTCTTCTACTCTAACATTATGAATTTCTTCAAGACAGAGATCACTCTGGCCAATGGCGAGATCCGGAAGCGCCCCCTCATCGAGACGAACGGCGAGACGGGGGAGATCGTGTGGGACAAGGGCAGGGATTTCGCGACCGTCAGGAAGGTTCTCTCCATGCCACAAGTGAATATCGTCAAGAAGACAGAGGTCCAGACTGGCGGGTTCTCTAAGGAGTCAATTCTGCCTAAGCGGAACAGCGACAAGCTCATCGCCCGCAAGAAGGACTGGGATCCGAAGAAGTACGGCGGGTTCGACAGCCCCACTGTGGCCTACTCGGTCCTGGTTGTGGCGAAGGTTGAGAAGGGCAAGTCCAAGAAGCTCAAGAGCGTGAAGGAGCTGCTGGGGATCACGATTATGGAGCGCTCCAGCTTCGAGAAGAACCCGATCGATTTCCTGGAGGCGAAGGGCTACAAGGAGGTGAAGAAGGACCTGATCATTAAGCTCCCCAAGTACTCACTCTTCGAGCTGGAGAACGGCAGGAAGCGGATGCTGGCTTCCGCTGGCGAGCTGCAGAAGGGGAACGAGCTGGCTCTGCCGTCCAAGTATGTGAACTTCCTCTACCTGGCCTCCCACTACGAGAAGCTCAAGGGCAGCCCCGAGGACAACGAGCAGAAGCAGCTGTTCGTCGAGCAGCACAAGCATTACCTCGACGAGATCATTGAGCAGATTTCCGAGTTCTCCAAGCGCGTGATCCTGGCCGACGCGAATCTGGATAAGGTCCTCTCCGCGTACAACAAGCACCGCGACAAGCCAATCAGGGAGCAGGCTGAGAATATCATTCATCTCTTCACCCTGACGAACCTCGGCGCCCCTGCTGCTTTCAAGTACTTCGACACAACTATCGATCGCAAGAGGTACACAAGCACTAAGGAGGTCCTGGACGCGACCCTCATCCACCAGTCGATTACCGGCCTCTACGAGACGCGCATCGACCTGTCTCAGCTCGGGGGCGACGAATTCTCCGGGAGCGAGACGCCAGGCACCTCCGAGTCGGCCACCCCAGAATCTGCCACAGTGGTGTCCGGCCAAAAGCAGGACCGCCAGGGCGGAGAACGCAGAAGGTCCCAGCTCGATAGGGATCAGTGTGCCTACTGCAAGGAGAAGGGCCACTGGGCCAAAGACTGCCCGAAAAAGCCGCGCGGCCCACGCGGCCCAAGGCCACAAACATCCCTCCTTCCAAAGAAGAAGCGGAAGGTGGAGCTCAGCGGAGGATCTTCCGGAGGATCTAGCGGCTCCGAGACACCAGGAACATCCGAAAGCGCTACACCAGAATCTAGCGGAGGCTCTTCCGGAGGATCTAGGCCTACCCTCAACATCGAGGATGAGTATCGCCTCCACGAAACCTCCAAAGAACCGGACGTGTCCCTCGGCAGCACATGGCTCAGCGACTTCCCACAAGCGTGGGCCGAAACCGGCGGCATGGGCCTCGCCGTCCGCCAAGCCCCACTCATTATCCCGCTGAAGGCGACCTCCACACCGGTGTCCATCAAGCAGTACCCGATGAGCCAAGAGGCGAGGCTCGGGATTAAGCCGCACATTCAGCGCCTCCTCGATCAAGGCATTCTCGTGCCGTGCCAATCCCCGTGGAATACACCACTCCTCCCGGTCAAAAAGCCGGGCACCAACGACTATCGCCCGGTCCAAGATCTCCGCGAGGTCAACAAGCGCGTGGAAGACATCCACCCGACCGTCCCGAACCCGTATAATCTGCTCTCCGGGCTCCCACCATCCCACCAGTGGTATACAGTGCTGGACCTCAAAGACGCCTTCTTCTGTCTCCGCCTCCACCCAACAAGCCAGCCGCTCTTCGCCTTCGAGTGGCGCGACCCGGAGATGGGCATCTCCGGCCAACTGACATGGACACGCCTCCCGCAAGGCTTCAAGAACAGCCCGACACTCTTCAACGAGGCGCTCCATAGGGACCTCGCGGATTTTCGCATCCAGCATCCGGACCTCATCCTCCTCCAGTAT**gcc**GATGATCTCCTCCTCGCCGCGACCTCCGAGCTGGATTGTCAACAAGGCACACGCGCGCTCCTCCAAACACTCGGGAACCTCGGCTATCGCGCGTCCGCGAAAAAGGCCCAAATCTGCCAGAAGCAAGTGAAGTACCTCGGGTATCTGCTCAAGGAAGGCCAACGCTGGCTCACCGAAGCGCGCAAAGAAACAGTGATGGGGCAACCGACACCGAAAACACCACGCCAGCTGCGCGAGTTTCTCGGCAAAGCCGGCTTCTGTCGCCTCTTCATCCCGGGCTTTGCCGAGATGGCCGCGCCACTCTACCCACTCACCAAGCCGGGCACACTGTTTAACTGGGGGCCGGATCAGCAGAAAGCCTACCAAGAGATCAAACAAGCGCTCCTCACCGCCCCAGCGCTCGGGCTCCCAGATCTCACAAAGCCGTTCGAGCTGTTCGTCGATGAGAAGCAAGGCTACGCGAAGGGCGTGCTCACACAGAAGCTCGGCCCGTGGAGGAGGCCAGTGGCCTATCTCTCCAAAAAACTCGATCCAGTGGCCGCCGGCTGGCCACCGTGTCTGCGCATGGTCGCCGCGATTGCCGTGCTCACAAAGGATGCCGGCAAACTCACAATGGGCCAGCCGCTGGTGATCCTCGCGCCACATGCCGTGGAAGCCCTCGTCAAACAGCCGCCGGATAGGTGGCTCTCCAATGCGCGCATGACCCATTACCAAGCGCTCCTCCTCGACACCGATCGCGTCCAGTTCGGCCCAGTGGTCGCCCTCAATCCGGCGACACTGCTGCCACTCCCAGAGGAGGGCCTCCAACACAACTGTCTGGATATTCTCGCGGAAGCGCATGGCACAAGGCCAGACCTCACAGATCAACCGCTCAGCGGCGGCAGCCCGAAGAAGAAAAGGAAGGTGTGAGCGGCCGCGGTACGCTGAAATCACCAGTCTCTCTCTACAAATCTATCTCTCTCTATTTTCTCCATAAATAATGTGTGAGTAGTTTCCCGATAAGGGAAATTAGGGTTCTTATAGGGTTTCGCTCATGTGTTGAGCATATAAGAAACCCTTAGTATGTATTTGTATTTGTAAAATACTTCTATCAATAAAATTTCTAATTCCTAAAACCAAAATCCAGTACTAAAATCCAGATCTCCTAAAGTCCCTATAGATCTTTGTCGTGAATATAAACCAGACACGAGACGACTAAACCTGGAGCCCAGACGCCGTTCGAAGCTAGAAGTACCGCTTAGGCAGGAGGCCGTTAGGGAAAAGATGCTAAGGCAGGGTTGGTTACGTTGACTCCCCCGTAGGTTTGGTTTAAATATGATGAAGTGGACGGAAGGAAGGAGGAAGACAAGGAAGGATAAGGTTGCAGGCCCTGTGCAAGGTAAGAAGATGGAAATTTGATAGAGGTACGCTACTATACTTATACTATACGCTAAGGGAATGCTTGTATTTATACCCTATACCCCCTAATAACCCCTTATCAATTTAAGAAATAATCCGCATAAGCCCCCGCTTAAAAATTGGTATCAGAGCCATGAATAGGTCTATGACCAAAACTCAAGAGGATAAAACCTCACCAAAATACGAAAGAGTTCTTAACTCTAAAGATAAAAGATCTTTCAAGATCAAAACTAGTTCCCTCACACCGGTGACGGGGATCGCATGCGATATCTCGAGATCTAGCTTGGCGTAATCATGGTCATAGCTGTTTCCTGTGTGAAATTGTTATCCGCTCACAATTCCACACAACATACGAGCCGGAAGCATAAAGTGTAAAGCCTGGGGTGCCTAATGAGTGAGCTAACTCACATTAATTGCGTTGCGCTCACTGCCCGCTTTCCAGTCGGGAAACCTGTCGTGCCAGCTGCATTAATGAATCGGCCAACGCGCGGGGAGAGGCGGTTTGCGTATTGGGCGCTCTTCCGCTTCCTCGCTCACTGACTCGCTGCGCTCGGTCGTTCGGCTGCGGCGAGCGGTATCAGCTCACTCAAAGGCGGTAATACGGTTATCCACAGAATCAGGGGATAACGCAGGAAAGAACATGTGAGCAAAAGGCCAGCAAAAGGCCAGGAACCGTAAAAAGGCCGCGTTGCTGGCGTTTTTCCATAGGCTCCGCCCCCCTGACGAGCATCACAAAAATCGACGCTCAAGTCAGAGGTGGCGAAACCCGACAGGACTATAAAGATACCAGGCGTTTCCCCCTGGAAGCTCCCTCGTGCGCTCTCCTGTTCCGACCCTGCCGCTTACCGGATACCTGTCCGCCTTTCTCCCTTCGGGAAGCGTGGCGCTTTCTCATAGCTCACGCTGTAGGTATCTCAGTTCGGTGTAGGTCGTTCGCTCCAAGCTGGGCTGTGTGCACGAACCCCCCGTTCAGCCCGACCGCTGCGCCTTATCCGGTAACTATCGTCTTGAGTCCAACCCGGTAAGACACGACTTATCGCCACTGGCAGCAGCCACTGGTAACAGGATTAGCAGAGCGAGGTATGTAGGCGGTGCTACAGAGTTCTTGAAGTGGTGGCCTAACTACGGCTACACTAGAAGAACAGTATTTGGTATCTGCGCTCTGCTGAAGCCAGTTACCTTCGGAAAAAGAGTTGGTAGCTCTTGATCCGGCAAACAAACCACCGCTGGTAGCGGTGGTTTTTTTGTTTGCAAGCAGCAGATTACGCGCAGAAAAAAAGGATCTCAAGAAGATCCTTTGATCTTTTCTACGGGGTCTGACGCTCAGTGGAACGAAAACTCACGTTAAGGGATTTTGGTCATGAGATTATCAAAAAGGATCTTCACCTAGATCCTTTTAAATTAAAAATGAAGTTTTAAATCAATCTAAAGTATATATGAGTAAACTTGGTCTGACAGTTACCAATGCTTAATCAGTGAGGCACCTATCTCAGCGATCTGTCTATTTCGTTCATCCATAGTTGCCTGACTCCCCGTCGTGTAGATAACTACGATACGGGAGGGCTTACCATCTGGCCCCAGTGCTGCAATGATACCGCGAGACCCACGCTCACCGGCTCCAGATTTATCAGCAATAAACCAGCCAGCCGGAAGGGCCGAGCGCAGAAGTGGTCCTGCAACTTTATCCGCCTCCATCCAGTCTATTAATTGTTGCCGGGAAGCTAGAGTAAGTAGTTCGCCAGTTAATAGTTTGCGCAACGTTGTTGCCATTGCTACAGGCATCGTGGTGTCACGCTCGTCGTTTGGTATGGCTTCATTCAGCTCCGGTTCCCAACGATCAAGGCGAGTTACATGATCCCCCATGTTGTGCAAAAAAGCGGTTAGCTCCTTCGGTCCTCCGATCGTTGTCAGAAGTAAGTTGGCCGCAGTGTTATCACTCATGGTTATGGCAGCACTGCATAATTCTCTTACTGTCATGCCATCCGTAAGATGCTTTTCTGTGACTGGTGAGTACTCAACCAAGTCATTCTGAGAATAGTGTATGCGGCGACCGAGTTGCTCTTGCCCGGCGTCAATACGGGATAATACCGCGCCACATAGCAGAACTTTAAAAGTGCTCATCATTGGAAAACGTTCTTCGGGGCGAAAACTCTCAAGGATCTTACCGCTGTTGAGATCCAGTTCGATGTAACCCACTCGTGCACCCAACTGATCTTCAGCATCTTTTACTTTCACCAGCGTTTCTGGGTGAGCAAAAACAGGAAGGCAAAATGCCGCAAAAAAGGGAATAAGGGCGACACGGAAATGTTGAATACTCATACTCTTCCTTTTTCAATATTATTGAAGCATTTATCAGGGTTATTGTCTCATGAGCGGATACATATTTGAATGTATTTAGAAAAATAAACAAATAGGGGTTCCGCGCACATTTCCCCGAAAAGTGCCACCTGCCAGTGCCAAGCTAATTC

**ePPEmax***

GAGCTCGGTACCTGACCCGGTCGTGCCCCTCTCTAGAGATAATGAGCATTGCATGTCTAAGTTATAAAAAATTACCACATATTTTTTTTGTCACACTTGTTTGAAGTGCAGTTTATCTATCTTTATACATATATTTAAACTTTACTCTACGAATAATATAATCTATAGTACTACAATAATATCAGTGTTTTAGAGAATCATATAAATGAACAGTTAGACATGGTCTAAAGGACAATTGAGTATTTTGACAACAGGACTCTACAGTTTTATCTTTTTAGTGTGCATGTGTTCTCCTTTTTTTTTGCAAATAGCTTCACCTATATAATACTTCATCCATTTTATTAGTACATCCATTTAGGGTTTAGGGTTAATGGTTTTTATAGACTAATTTTTTTAGTACATCTATTTTATTCTATTTTAGCCTCTAAATTAAGAAAACTAAAACTCTATTTTAGTTTTTTTATTTAATAATTTAGATATAAAATAGAATAAAATAAAGTGACTAAAAATTAAACAAATACCCTTTAAGAAATTAAAAAAACTAAGGAAACATTTTTCTTGTTTCGAGTAGATAATGCCAGCCTGTTAAACGCCGTCGACGAGTCTAACGGACACCAACCAGCGAACCAGCAGCGTCGCGTCGGGCCAAGCGAAGCAGACGGCACGGCATCTCTGTCGCTGCCTCTGGACCCCTCTCGATCGAGAGTTCCGCTCCACCGTTGGACTTGCTCCGCTGTCGGCATCCAGAAATTGCGTGGCGGAGCGGCAGACGTGAGCCGGCACGGCAGGCGGCCTCCTCCTCCTCTCACGGCACCGGCAGCTACGGGGGATTCCTTTCCCACCGCTCCTTCGCTTTCCCTTCCTCGCCCGCCGTAATAAATAGACACCCCCTCCACACCCTCTTTCCCCAACCTCGTGTTGTTCGGAGCGCACACACACACAACCAGATCTCCCCCAAATCCACCCGTCGGCACCTCCGCTTCAAGGTACGCCGCTCGTCCTCCCCCCCCCCCCCTCTCTACCTTCTCTAGATCGGCGTTCCGGTCCATGGTTAGGGCCCGGTAGTTCTACTTCTGTTCATGTTTGTGTTAGATCCGTGTTTGTGTTAGATCCGTGCTGCTAGCGTTCGTACACGGATGCGACCTGTACGTCAGACACGTTCTGATTGCTAACTTGCCAGTGTTTCTCTTTGGGGAATCCTGGGATGGCTCTAGCCGTTCCGCAGACGGGATCGATTTCATGATTTTTTTTGTTTCGTTGCATAGGGTTTGGTTTGCCCTTTTCCTTTATTTCAATATATGCCGTGCACTTGTTTGTCGGGTCATCTTTTCATGCTTTTTTTTGTCTTGGTTGTGATGATGTGGTCTGGTTGGGCGGTCGTTCTAGATCGGAGTAGAATTAATTCTGTTTCAAACTACCTGGTGGATTTATTAATTTTGGATCTGTATGTGTGTGCCATACATATTCATAGTTACGAATTGAAGATGATGGATGGAAATATCGATCTAGGATAGGTATACATGTTGATGCGGGTTTTACTGATGCATATACAGAGATGCTTTTTGTTCGCTTGGTTGTGATGATGTGGTGTGGTTGGGCGGTCGTTCATTCGTTCTAGATCGGAGTAGAATACTGTTTCAAACTACCTGGTGTATTTATTAATTTTGGAACTGTATGTGTGTGTCATACATCTTCATAGTTACGAGTTTAAGATGGATGGAAATATCGATCTAGGATAGGTATACATGTTGATGTGGGTTTTACTGATGCATATACATGATGGCATATGCAGCATCTATTCATATGCTCTAACCTTGAGTACCTATCTATTATAATAAACAAGTATGTTTTATAATTATTTTGATCTTGATATACTTGGATGATGGCATATGCAGCAGCTATATGTGGATTTTTTTAGCCCTGCCTTCATACGCTATTTATTTGCTTGGTACTGTTTCTTTTGTCGATGCTCACCCTGTTGTTTGGTGTTACTTCTGCAAAGCTTATGCCAGCCGCCAAGCGCGTCAAGCTGGACGGCGGAAAGCGCACCGCCGACGGATCCGAGTTCGAGAGCCCTAAGAAAAAGAGAAAAGTGGACAAGAAGTACTCGATCGGCCTCGATATTGGGACTAACTCTGTTGGCTGGGCCGTGATCACCGACGAGTACAAGGTGCCCTCAAAGAAGTTCAAGGTCCTGGGCAACACCGATCGGCATTCCATCAAGAAGAATCTCATTGGCGCTCTCCTGTTCGACAGCGGCGAGACGGCTGAGGCTACGCGGCTCAAGCGCACCGCCCGCAGGCGGTACACGCGCAGGAAGAATCGCATCTGCTACCTGCAGGAGATTTTCTCCAACGAGATGGCGAAGGTTGACGATTCTTTCTTCCACAGGCTGGAGGAGTCATTCCTCGTGGAGGAGGATAAGAAGCACGAGCGGCATCCAATCTTCGGCAACATTGTCGACGAGGTTGCCTACCACGAGAAGTACCCTACGATCTACCATCTGCGGAAGAAGCTCGTGGACTCCACAGATAAGGCGGACCTCCGCCTGATCTACCTCGCTCTGGCCCACATGATTAAGTTCAGGGGCCATTTCCTGATCGAGGGGGATCTCAACCCGGACAATAGCGATGTTGACAAGCTGTTCATCCAGCTCGTGCAGACGTACAACCAGCTCTTCGAGGAGAACCCCATTAATGCGTCAGGCGTCGACGCGAAGGCTATCCTGTCCGCTAGGCTCTCGAAGTCTCGGaagCTCGAGAACCTGATCGCCCAGCTGCCGGGCGAGAAGAAGAACGGCCTGTTCGGGAATCTCATTGCGCTCAGCCTGGGGCTCACGCCCAACTTCAAGTCGAATTTCGATCTCGCTGAGGACGCCAAGCTGCAGCTCTCCAAGGACACATACGACGATGACCTGGATAACCTCCTGGCCCAGATCGGCGATCAGTACGCGGACCTGTTCCTCGCTGCCAAGAATCTGTCGGACGCCATCCTCCTGTCTGATATTCTCAGGGTGAACACCGAGATTACGAAGGCTCCGCTCTCAGCCTCCATGATCAAGCGCTACGACGAGCACCATCAGGATCTGACCCTCCTGAAGGCGCTGGTCAGGCAGCAGCTCCCCGAGAAGTACAAGGAGATCTTCTTCGATCAGTCGAAGAACGGCTACGCTGGGTACATTGACGGCGGGGCCTCTCAGGAGGAGTTCTACAAGTTCATCAAGCCGATTCTGGAGAAGATGGACGGCACGGAGGAGCTGCTGGTGAAGCTCaagCGCGAGGACCTCCTGAGGAAGCAGCGGACATTCGATAACGGCAGCATCCCACACCAGATTCATCTCGGGGAGCTGCACGCTATCCTGAGGAGGCAGGAGGACTTCTACCCTTTCCTCAAGGATAACCGCGAGAAGATCGAGAAGATTCTGACTTTCAGGATCCCGTACTACGTCGGCCCACTCGCTAGGGGCAACTCCCGCTTCGCTTGGATGACCCGCAAGTCAGAGGAGACGATCACGCCGTGGAACTTCGAGGAGGTGGTCGACAAGGGCGCTAGCGCTCAGTCGTTCATCGAGAGGATGACGAATTTCGACAAGAACCTGCCAAATGAGAAGGTGCTCCCTAAGCACTCGCTCCTGTACGAGTACTTCACAGTCTACAACGAGCTGACTAAGGTGAAGTATGTGACCGAGGGCATGAGGAAGCCGGCTTTCCTGTCTGGGGAGCAGAAGAAGGCCATCGTGGACCTCCTGTTCAAGACCAACCGGAAGGTCACGGTTAAGCAGCTCAAGGAGGACTACTTCAAGAAGATTGAGTGCTTCGATTCGGTCGAGATCTCTGGCGTTGAGGACCGCTTCAACGCCTCCCTGGGGACCTACCACGATCTCCTGAAGATCATTAAGGATAAGGACTTCCTGGACAACGAGGAGAATGAGGATATCCTCGAGGACATTGTGCTGACACTCACTCTGTTCGAGGACCGGGAGATGATCGAGGAGCGCCTGAAGACTTACGCCCATCTCTTCGATGACAAGGTCATGAAGCAGCTCAAGAGGAGGAGGTACACCGGCTGGGGGAGGCTGAGCAGGAAGCTCATCAACGGCATTCGGGACAAGCAGTCCGGGAAGACGATCCTCGACTTCCTGAAGAGCGATGGCTTCGCGAACCGCAATTTCATGCAGCTGATTCACGATGACAGCCTCACATTCAAGGAGGATATCCAGAAGGCTCAGGTGAGCGGCCAGGGGGACTCGCTGCACGAGCATATCGCGAACCTCGCTGGCTCGCCAGCTATCAAGAAGGGGATTCTGCAGACCGTGAAGGTTGTGGACGAGCTGGTGAAGGTCATGGGCAGGCACAAGCCTGAGAACATCGTCATTGAGATGGCCCGGGAGAATCAGACCACGCAGAAGGGCCAGAAGAACTCACGCGAGAGGATGAAGAGGATCGAGGAGGGCATTAAGGAGCTGGGGTCCCAGATCCTCAAGGAGCACCCGGTGGAGAACACGCAGCTGCAGAATGAGAAGCTCTACCTGTACTACCTCCAGAATGGCCGCGATATGTATGTGGACCAGGAGCTGGATATTAACAGGCTCAGCGATTACGACGTCGATGCCATCGTTCCACAGTCATTCCTGAAGGATGACTCCATTGACAACAAGGTCCTCACCAGGTCGGACAAGAACCGGGGCAAGTCTGATAATGTTCCTTCAGAGGAGGTCGTTAAGAAGATGAAGAACTACTGGCGCCAGCTCCTGAATGCCAAGCTGATCACGCAGCGGAAGTTCGATAACCTCACAAAGGCTGAGAGGGGCGGGCTCTCTGAGCTGGACAAGGCGGGCTTCATCAAGAGGCAGCTGGTCGAGACACGGCAGATCACTAAGCACGTTGCGCAGATTCTCGACTCACGGATGAACACTAAGTACGATGAGAATGACAAGCTGATCCGCGAGGTGAAGGTCATCACCCTGAAGTCAAAGCTCGTCTCCGACTTCAGGAAGGATTTCCAGTTCTACAAGGTTCGGGAGATCAACAATTACCACCATGCCCATGACGCGTACCTGAACGCGGTGGTCGGCACAGCTCTGATCAAGAAGTACCCAAAGCTCGAGAGCGAGTTCGTGTACGGGGACTACAAGGTTTACGATGTGAGGAAGATGATCGCCAAGTCGGAGCAGGAGATTGGCAAGGCTACCGCCAAGTACTTCTTCTACTCTAACATTATGAATTTCTTCAAGACAGAGATCACTCTGGCCAATGGCGAGATCCGGAAGCGCCCCCTCATCGAGACGAACGGCGAGACGGGGGAGATCGTGTGGGACAAGGGCAGGGATTTCGCGACCGTCAGGAAGGTTCTCTCCATGCCACAAGTGAATATCGTCAAGAAGACAGAGGTCCAGACTGGCGGGTTCTCTAAGGAGTCAATTCTGCCTAAGCGGAACAGCGACAAGCTCATCGCCCGCAAGAAGGACTGGGATCCGAAGAAGTACGGCGGGTTCGACAGCCCCACTGTGGCCTACTCGGTCCTGGTTGTGGCGAAGGTTGAGAAGGGCAAGTCCAAGAAGCTCAAGAGCGTGAAGGAGCTGCTGGGGATCACGATTATGGAGCGCTCCAGCTTCGAGAAGAACCCGATCGATTTCCTGGAGGCGAAGGGCTACAAGGAGGTGAAGAAGGACCTGATCATTAAGCTCCCCAAGTACTCACTCTTCGAGCTGGAGAACGGCAGGAAGCGGATGCTGGCTTCCGCTGGCGAGCTGCAGAAGGGGAACGAGCTGGCTCTGCCGTCCAAGTATGTGAACTTCCTCTACCTGGCCTCCCACTACGAGAAGCTCAAGGGCAGCCCCGAGGACAACGAGCAGAAGCAGCTGTTCGTCGAGCAGCACAAGCATTACCTCGACGAGATCATTGAGCAGATTTCCGAGTTCTCCAAGCGCGTGATCCTGGCCGACGCGAATCTGGATAAGGTCCTCTCCGCGTACAACAAGCACCGCGACAAGCCAATCAGGGAGCAGGCTGAGAATATCATTCATCTCTTCACCCTGACGAACCTCGGCGCCCCTGCTGCTTTCAAGTACTTCGACACAACTATCGATCGCAAGAGGTACACAAGCACTAAGGAGGTCCTGGACGCGACCCTCATCCACCAGTCGATTACCGGCCTCTACGAGACGCGCATCGACCTGTCTCAGCTCGGGGGCGACGAATTCTCCGGGAGCGAGACGCCAGGCACCTCCGAGTCGGCCACCCCAGAATCTGCCACAGTGGTGTCCGGCCAAAAGCAGGACCGCCAGGGCGGAGAACGCAGAAGGTCCCAGCTCGATAGGGATCAGTGTGCCTACTGCAAGGAGAAGGGCCACTGGGCCAAAGACTGCCCGAAAAAGCCGCGCGGCCCACGCGGCCCAAGGCCACAAACATCCCTCCTTCCAAAGAAGAAGCGGAAGGTGGAGCTCAGCGGAGGATCTTCCGGAGGATCTAGCGGCTCCGAGACACCAGGAACATCCGAAAGCGCTACACCAGAATCTAGCGGAGGCTCTTCCGGAGGATCTAGGCCTACCCTCAACATCGAGGATGAGTATCGCCTCCACGAAACCTCCAAAGAACCGGACGTGTCCCTCGGCAGCACATGGCTCAGCGACTTCCCACAAGCGTGGGCCGAAACCGGCGGCATGGGCCTCGCCGTCCGCCAAGCCCCACTCATTATCCCGCTGAAGGCGACCTCCACACCGGTGTCCATCAAGCAGTACCCGATGAGCCAAGAGGCGAGGCTCGGGATTAAGCCGCACATTCAGCGCCTCCTCGATCAAGGCATTCTCGTGCCGTGCCAATCCCCGTGGAATACACCACTCCTCCCGGTCAAAAAGCCGGGCACCAACGACTATCGCCCGGTCCAAGATCTCCGCGAGGTCAACAAGCGCGTGGAAGACATCCACCCGACCGTCCCGAACCCGTATAATCTGCTCTCCGGGCTCCCACCATCCCACCAGTGGTATACAGTGCTGGACCTCAAAGACGCCTTCTTCTGTCTCCGCCTCCACCCAACAAGCCAGCCGCTCTTCGCCTTCGAGTGGCGCGACCCGGAGATGGGCATCTCCGGCCAACTGACATGGACACGCCTCCCGCAAGGCTTCAAGAACAGCCCGACACTCTTCAACGAGGCGCTCCATAGGGACCTCGCGGATTTTCGCATCCAGCATCCGGACCTCATCCTCCTCCAGTATGTGGATGATCTCCTCCTCGCCGCGACCTCCGAGCTGGATTGTCAACAAGGCACACGCGCGCTCCTCCAAACACTCGGGAACCTCGGCTATCGCGCGTCCGCGAAAAAGGCCCAAATCTGCCAGAAGCAAGTGAAGTACCTCGGGTATCTGCTCAAGGAAGGCCAACGCTGGCTCACCGAAGCGCGCAAAGAAACAGTGATGGGGCAACCGACACCGAAAACACCACGCCAGCTGCGCGAGTTTCTCGGCAAAGCCGGCTTCTGTCGCCTCTTCATCCCGGGCTTTGCCGAGATGGCCGCGCCACTCTACCCACTCACCAAGCCGGGCACACTGTTTAACTGGGGGCCGGATCAGCAGAAAGCCTACCAAGAGATCAAACAAGCGCTCCTCACCGCCCCAGCGCTCGGGCTCCCAGATCTCACAAAGCCGTTCGAGCTGTTCGTCGATGAGAAGCAAGGCTACGCGAAGGGCGTGCTCACACAGAAGCTCGGCCCGTGGAGGAGGCCAGTGGCCTATCTCTCCAAAAAACTCGATCCAGTGGCCGCCGGCTGGCCACCGTGTCTGCGCATGGTCGCCGCGATTGCCGTGCTCACAAAGGATGCCGGCAAACTCACAATGGGCCAGCCGCTGGTGATCCTCGCGCCACATGCCGTGGAAGCCCTCGTCAAACAGCCGCCGGATAGGTGGCTCTCCAATGCGCGCATGACCCATTACCAAGCGCTCCTCCTCGACACCGAAGCGGCGGCAGCAAAAGGACCGCCGATTCCCAACATTCCACCCCACCAAAAACCAAGAGGAAGGTGGGCTCTGGCCCAAAGAAGAAGAGGAAGGTCTGAGCGGCCGCGGTACGCTGAAATCACCAGTCTCTCTCTACAAATCTATCTCTCTCTATTTTCTCCATAAATAATGTGTGAGTAGTTTCCCGATAAGGGAAATTAGGGTTCTTATAGGGTTTCGCTCATGTGTTGAGCATATAAGAAACCCTTAGTATGTATTTGTATTTGTAAAATACTTCTATCAATAAAATTTCTAATTCCTAAAACCAAAATCCAGTACTAAAATCCAGATCTCCTAAAGTCCCTATAGATCTTTGTCGTGAATATAAACCAGACACGAGACGACTAAACCTGGAGCCCAGACGCCGTTCGAAGCTAGAAGTACCGCTTAGGCAGGAGGCCGTTAGGGAAAAGATGCTAAGGCAGGGTTGGTTACGTTGACTCCCCCGTAGGTTTGGTTTAAATATGATGAAGTGGACGGAAGGAAGGAGGAAGACAAGGAAGGATAAGGTTGCAGGCCCTGTGCAAGGTAAGAAGATGGAAATTTGATAGAGGTACGCTACTATACTTATACTATACGCTAAGGGAATGCTTGTATTTATACCCTATACCCCCTAATAACCCCTTATCAATTTAAGAAATAATCCGCATAAGCCCCCGCTTAAAAATTGGTATCAGAGCCATGAATAGGTCTATGACCAAAACTCAAGAGGATAAAACCTCACCAAAATACGAAAGAGTTCTTAACTCTAAAGATAAAAGATCTTTCAAGATCAAAACTAGTTCCCTCACACCGGTGACGGGGATCGCATGCGATATCTCGAGATCTAGCTTGGCGTAATCATGGTCATAGCTGTTTCCTGTGTGAAATTGTTATCCGCTCACAATTCCACACAACATACGAGCCGGAAGCATAAAGTGTAAAGCCTGGGGTGCCTAATGAGTGAGCTAACTCACATTAATTGCGTTGCGCTCACTGCCCGCTTTCCAGTCGGGAAACCTGTCGTGCCAGCTGCATTAATGAATCGGCCAACGCGCGGGGAGAGGCGGTTTGCGTATTGGGCGCTCTTCCGCTTCCTCGCTCACTGACTCGCTGCGCTCGGTCGTTCGGCTGCGGCGAGCGGTATCAGCTCACTCAAAGGCGGTAATACGGTTATCCACAGAATCAGGGGATAACGCAGGAAAGAACATGTGAGCAAAAGGCCAGCAAAAGGCCAGGAACCGTAAAAAGGCCGCGTTGCTGGCGTTTTTCCATAGGCTCCGCCCCCCTGACGAGCATCACAAAAATCGACGCTCAAGTCAGAGGTGGCGAAACCCGACAGGACTATAAAGATACCAGGCGTTTCCCCCTGGAAGCTCCCTCGTGCGCTCTCCTGTTCCGACCCTGCCGCTTACCGGATACCTGTCCGCCTTTCTCCCTTCGGGAAGCGTGGCGCTTTCTCATAGCTCACGCTGTAGGTATCTCAGTTCGGTGTAGGTCGTTCGCTCCAAGCTGGGCTGTGTGCACGAACCCCCCGTTCAGCCCGACCGCTGCGCCTTATCCGGTAACTATCGTCTTGAGTCCAACCCGGTAAGACACGACTTATCGCCACTGGCAGCAGCCACTGGTAACAGGATTAGCAGAGCGAGGTATGTAGGCGGTGCTACAGAGTTCTTGAAGTGGTGGCCTAACTACGGCTACACTAGAAGAACAGTATTTGGTATCTGCGCTCTGCTGAAGCCAGTTACCTTCGGAAAAAGAGTTGGTAGCTCTTGATCCGGCAAACAAACCACCGCTGGTAGCGGTGGTTTTTTTGTTTGCAAGCAGCAGATTACGCGCAGAAAAAAAGGATCTCAAGAAGATCCTTTGATCTTTTCTACGGGGTCTGACGCTCAGTGGAACGAAAACTCACGTTAAGGGATTTTGGTCATGAGATTATCAAAAAGGATCTTCACCTAGATCCTTTTAAATTAAAAATGAAGTTTTAAATCAATCTAAAGTATATATGAGTAAACTTGGTCTGACAGTTACCAATGCTTAATCAGTGAGGCACCTATCTCAGCGATCTGTCTATTTCGTTCATCCATAGTTGCCTGACTCCCCGTCGTGTAGATAACTACGATACGGGAGGGCTTACCATCTGGCCCCAGTGCTGCAATGATACCGCGAGACCCACGCTCACCGGCTCCAGATTTATCAGCAATAAACCAGCCAGCCGGAAGGGCCGAGCGCAGAAGTGGTCCTGCAACTTTATCCGCCTCCATCCAGTCTATTAATTGTTGCCGGGAAGCTAGAGTAAGTAGTTCGCCAGTTAATAGTTTGCGCAACGTTGTTGCCATTGCTACAGGCATCGTGGTGTCACGCTCGTCGTTTGGTATGGCTTCATTCAGCTCCGGTTCCCAACGATCAAGGCGAGTTACATGATCCCCCATGTTGTGCAAAAAAGCGGTTAGCTCCTTCGGTCCTCCGATCGTTGTCAGAAGTAAGTTGGCCGCAGTGTTATCACTCATGGTTATGGCAGCACTGCATAATTCTCTTACTGTCATGCCATCCGTAAGATGCTTTTCTGTGACTGGTGAGTACTCAACCAAGTCATTCTGAGAATAGTGTATGCGGCGACCGAGTTGCTCTTGCCCGGCGTCAATACGGGATAATACCGCGCCACATAGCAGAACTTTAAAAGTGCTCATCATTGGAAAACGTTCTTCGGGGCGAAAACTCTCAAGGATCTTACCGCTGTTGAGATCCAGTTCGATGTAACCCACTCGTGCACCCAACTGATCTTCAGCATCTTTTACTTTCACCAGCGTTTCTGGGTGAGCAAAAACAGGAAGGCAAAATGCCGCAAAAAAGGGAATAAGGGCGACACGGAAATGTTGAATACTCATACTCTTCCTTTTTCAATATTATTGAAGCATTTATCAGGGTTATTGTCTCATGAGCGGATACATATTTGAATGTATTTAGAAAAATAAACAAATAGGGGTTCCGCGCACATTTCCCCGAAAAGTGCCACCTGCCAGTGCCAAGCTAATTC

**ePPEplus**

GAGCTCGGTACCTGACCCGGTCGTGCCCCTCTCTAGAGATAATGAGCATTGCATGTCTAAGTTATAAAAAATTACCACATATTTTTTTTGTCACACTTGTTTGAAGTGCAGTTTATCTATCTTTATACATATATTTAAACTTTACTCTACGAATAATATAATCTATAGTACTACAATAATATCAGTGTTTTAGAGAATCATATAAATGAACAGTTAGACATGGTCTAAAGGACAATTGAGTATTTTGACAACAGGACTCTACAGTTTTATCTTTTTAGTGTGCATGTGTTCTCCTTTTTTTTTGCAAATAGCTTCACCTATATAATACTTCATCCATTTTATTAGTACATCCATTTAGGGTTTAGGGTTAATGGTTTTTATAGACTAATTTTTTTAGTACATCTATTTTATTCTATTTTAGCCTCTAAATTAAGAAAACTAAAACTCTATTTTAGTTTTTTTATTTAATAATTTAGATATAAAATAGAATAAAATAAAGTGACTAAAAATTAAACAAATACCCTTTAAGAAATTAAAAAAACTAAGGAAACATTTTTCTTGTTTCGAGTAGATAATGCCAGCCTGTTAAACGCCGTCGACGAGTCTAACGGACACCAACCAGCGAACCAGCAGCGTCGCGTCGGGCCAAGCGAAGCAGACGGCACGGCATCTCTGTCGCTGCCTCTGGACCCCTCTCGATCGAGAGTTCCGCTCCACCGTTGGACTTGCTCCGCTGTCGGCATCCAGAAATTGCGTGGCGGAGCGGCAGACGTGAGCCGGCACGGCAGGCGGCCTCCTCCTCCTCTCACGGCACCGGCAGCTACGGGGGATTCCTTTCCCACCGCTCCTTCGCTTTCCCTTCCTCGCCCGCCGTAATAAATAGACACCCCCTCCACACCCTCTTTCCCCAACCTCGTGTTGTTCGGAGCGCACACACACACAACCAGATCTCCCCCAAATCCACCCGTCGGCACCTCCGCTTCAAGGTACGCCGCTCGTCCTCCCCCCCCCCCCCTCTCTACCTTCTCTAGATCGGCGTTCCGGTCCATGGTTAGGGCCCGGTAGTTCTACTTCTGTTCATGTTTGTGTTAGATCCGTGTTTGTGTTAGATCCGTGCTGCTAGCGTTCGTACACGGATGCGACCTGTACGTCAGACACGTTCTGATTGCTAACTTGCCAGTGTTTCTCTTTGGGGAATCCTGGGATGGCTCTAGCCGTTCCGCAGACGGGATCGATTTCATGATTTTTTTTGTTTCGTTGCATAGGGTTTGGTTTGCCCTTTTCCTTTATTTCAATATATGCCGTGCACTTGTTTGTCGGGTCATCTTTTCATGCTTTTTTTTGTCTTGGTTGTGATGATGTGGTCTGGTTGGGCGGTCGTTCTAGATCGGAGTAGAATTAATTCTGTTTCAAACTACCTGGTGGATTTATTAATTTTGGATCTGTATGTGTGTGCCATACATATTCATAGTTACGAATTGAAGATGATGGATGGAAATATCGATCTAGGATAGGTATACATGTTGATGCGGGTTTTACTGATGCATATACAGAGATGCTTTTTGTTCGCTTGGTTGTGATGATGTGGTGTGGTTGGGCGGTCGTTCATTCGTTCTAGATCGGAGTAGAATACTGTTTCAAACTACCTGGTGTATTTATTAATTTTGGAACTGTATGTGTGTGTCATACATCTTCATAGTTACGAGTTTAAGATGGATGGAAATATCGATCTAGGATAGGTATACATGTTGATGTGGGTTTTACTGATGCATATACATGATGGCATATGCAGCATCTATTCATATGCTCTAACCTTGAGTACCTATCTATTATAATAAACAAGTATGTTTTATAATTATTTTGATCTTGATATACTTGGATGATGGCATATGCAGCAGCTATATGTGGATTTTTTTAGCCCTGCCTTCATACGCTATTTATTTGCTTGGTACTGTTTCTTTTGTCGATGCTCACCCTGTTGTTTGGTGTTACTTCTGCAAAGCTTATGCCAGCCGCCAAGCGCGTCAAGCTGGACGGCGGAAAGCGCACCGCCGACGGATCCGAGTTCGAGAGCCCTAAGAAAAAGAGAAAAGTGGACAAGAAGTACTCGATCGGCCTCGATATTGGGACTAACTCTGTTGGCTGGGCCGTGATCACCGACGAGTACAAGGTGCCCTCAAAGAAGTTCAAGGTCCTGGGCAACACCGATCGGCATTCCATCAAGAAGAATCTCATTGGCGCTCTCCTGTTCGACAGCGGCGAGACGGCTGAGGCTACGCGGCTCAAGCGCACCGCCCGCAGGCGGTACACGCGCAGGAAGAATCGCATCTGCTACCTGCAGGAGATTTTCTCCAACGAGATGGCGAAGGTTGACGATTCTTTCTTCCACAGGCTGGAGGAGTCATTCCTCGTGGAGGAGGATAAGAAGCACGAGCGGCATCCAATCTTCGGCAACATTGTCGACGAGGTTGCCTACCACGAGAAGTACCCTACGATCTACCATCTGCGGAAGAAGCTCGTGGACTCCACAGATAAGGCGGACCTCCGCCTGATCTACCTCGCTCTGGCCCACATGATTAAGTTCAGGGGCCATTTCCTGATCGAGGGGGATCTCAACCCGGACAATAGCGATGTTGACAAGCTGTTCATCCAGCTCGTGCAGACGTACAACCAGCTCTTCGAGGAGAACCCCATTAATGCGTCAGGCGTCGACGCGAAGGCTATCCTGTCCGCTAGGCTCTCGAAGTCTCGGaagCTCGAGAACCTGATCGCCCAGCTGCCGGGCGAGAAGAAGAACGGCCTGTTCGGGAATCTCATTGCGCTCAGCCTGGGGCTCACGCCCAACTTCAAGTCGAATTTCGATCTCGCTGAGGACGCCAAGCTGCAGCTCTCCAAGGACACATACGACGATGACCTGGATAACCTCCTGGCCCAGATCGGCGATCAGTACGCGGACCTGTTCCTCGCTGCCAAGAATCTGTCGGACGCCATCCTCCTGTCTGATATTCTCAGGGTGAACACCGAGATTACGAAGGCTCCGCTCTCAGCCTCCATGATCAAGCGCTACGACGAGCACCATCAGGATCTGACCCTCCTGAAGGCGCTGGTCAGGCAGCAGCTCCCCGAGAAGTACAAGGAGATCTTCTTCGATCAGTCGAAGAACGGCTACGCTGGGTACATTGACGGCGGGGCCTCTCAGGAGGAGTTCTACAAGTTCATCAAGCCGATTCTGGAGAAGATGGACGGCACGGAGGAGCTGCTGGTGAAGCTCaagCGCGAGGACCTCCTGAGGAAGCAGCGGACATTCGATAACGGCAGCATCCCACACCAGATTCATCTCGGGGAGCTGCACGCTATCCTGAGGAGGCAGGAGGACTTCTACCCTTTCCTCAAGGATAACCGCGAGAAGATCGAGAAGATTCTGACTTTCAGGATCCCGTACTACGTCGGCCCACTCGCTAGGGGCAACTCCCGCTTCGCTTGGATGACCCGCAAGTCAGAGGAGACGATCACGCCGTGGAACTTCGAGGAGGTGGTCGACAAGGGCGCTAGCGCTCAGTCGTTCATCGAGAGGATGACGAATTTCGACAAGAACCTGCCAAATGAGAAGGTGCTCCCTAAGCACTCGCTCCTGTACGAGTACTTCACAGTCTACAACGAGCTGACTAAGGTGAAGTATGTGACCGAGGGCATGAGGAAGCCGGCTTTCCTGTCTGGGGAGCAGAAGAAGGCCATCGTGGACCTCCTGTTCAAGACCAACCGGAAGGTCACGGTTAAGCAGCTCAAGGAGGACTACTTCAAGAAGATTGAGTGCTTCGATTCGGTCGAGATCTCTGGCGTTGAGGACCGCTTCAACGCCTCCCTGGGGACCTACCACGATCTCCTGAAGATCATTAAGGATAAGGACTTCCTGGACAACGAGGAGAATGAGGATATCCTCGAGGACATTGTGCTGACACTCACTCTGTTCGAGGACCGGGAGATGATCGAGGAGCGCCTGAAGACTTACGCCCATCTCTTCGATGACAAGGTCATGAAGCAGCTCAAGAGGAGGAGGTACACCGGCTGGGGGAGGCTGAGCAGGAAGCTCATCAACGGCATTCGGGACAAGCAGTCCGGGAAGACGATCCTCGACTTCCTGAAGAGCGATGGCTTCGCGAACCGCAATTTCATGCAGCTGATTCACGATGACAGCCTCACATTCAAGGAGGATATCCAGAAGGCTCAGGTGAGCGGCCAGGGGGACTCGCTGCACGAGCATATCGCGAACCTCGCTGGCTCGCCAGCTATCAAGAAGGGGATTCTGCAGACCGTGAAGGTTGTGGACGAGCTGGTGAAGGTCATGGGCAGGCACAAGCCTGAGAACATCGTCATTGAGATGGCCCGGGAGAATCAGACCACGCAGAAGGGCCAGAAGAACTCACGCGAGAGGATGAAGAGGATCGAGGAGGGCATTAAGGAGCTGGGGTCCCAGATCCTCAAGGAGCACCCGGTGGAGAACACGCAGCTGCAGAATGAGAAGCTCTACCTGTACTACCTCCAGAATGGCCGCGATATGTATGTGGACCAGGAGCTGGATATTAACAGGCTCAGCGATTACGACGTCGATGCCATCGTTCCACAGTCATTCCTGAAGGATGACTCCATTGACAACAAGGTCCTCACCAGGTCGGACAAGAACCGGGGCAAGTCTGATAATGTTCCTTCAGAGGAGGTCGTTAAGAAGATGAAGAACTACTGGCGCCAGCTCCTGAATGCCAAGCTGATCACGCAGCGGAAGTTCGATAACCTCACAAAGGCTGAGAGGGGCGGGCTCTCTGAGCTGGACAAGGCGGGCTTCATCAAGAGGCAGCTGGTCGAGACACGGCAGATCACTAAGCACGTTGCGCAGATTCTCGACTCACGGATGAACACTAAGTACGATGAGAATGACAAGCTGATCCGCGAGGTGAAGGTCATCACCCTGAAGTCAAAGCTCGTCTCCGACTTCAGGAAGGATTTCCAGTTCTACAAGGTTCGGGAGATCAACAATTACCACCATGCCCATGACGCGTACCTGAACGCGGTGGTCGGCACAGCTCTGATCAAGAAGTACCCAAAGCTCGAGAGCGAGTTCGTGTACGGGGACTACAAGGTTTACGATGTGAGGAAGATGATCGCCAAGTCGGAGCAGGAGATTGGCAAGGCTACCGCCAAGTACTTCTTCTACTCTAACATTATGAATTTCTTCAAGACAGAGATCACTCTGGCCAATGGCGAGATCCGGAAGCGCCCCCTCATCGAGACGAACGGCGAGACGGGGGAGATCGTGTGGGACAAGGGCAGGGATTTCGCGACCGTCAGGAAGGTTCTCTCCATGCCACAAGTGAATATCGTCAAGAAGACAGAGGTCCAGACTGGCGGGTTCTCTAAGGAGTCAATTCTGCCTAAGCGGAACAGCGACAAGCTCATCGCCCGCAAGAAGGACTGGGATCCGAAGAAGTACGGCGGGTTCGACAGCCCCACTGTGGCCTACTCGGTCCTGGTTGTGGCGAAGGTTGAGAAGGGCAAGTCCAAGAAGCTCAAGAGCGTGAAGGAGCTGCTGGGGATCACGATTATGGAGCGCTCCAGCTTCGAGAAGAACCCGATCGATTTCCTGGAGGCGAAGGGCTACAAGGAGGTGAAGAAGGACCTGATCATTAAGCTCCCCAAGTACTCACTCTTCGAGCTGGAGAACGGCAGGAAGCGGATGCTGGCTTCCGCTGGCGAGCTGCAGAAGGGGAACGAGCTGGCTCTGCCGTCCAAGTATGTGAACTTCCTCTACCTGGCCTCCCACTACGAGAAGCTCAAGGGCAGCCCCGAGGACAACGAGCAGAAGCAGCTGTTCGTCGAGCAGCACAAGCATTACCTCGACGAGATCATTGAGCAGATTTCCGAGTTCTCCAAGCGCGTGATCCTGGCCGACGCGAATCTGGATAAGGTCCTCTCCGCGTACAACAAGCACCGCGACAAGCCAATCAGGGAGCAGGCTGAGAATATCATTCATCTCTTCACCCTGACGAACCTCGGCGCCCCTGCTGCTTTCAAGTACTTCGACACAACTATCGATCGCAAGAGGTACACAAGCACTAAGGAGGTCCTGGACGCGACCCTCATCCACCAGTCGATTACCGGCCTCTACGAGACGCGCATCGACCTGTCTCAGCTCGGGGGCGACGAATTCTCCGGGAGCGAGACGCCAGGCACCTCCGAGTCGGCCACCCCAGAATCTGCCACAGTGGTGTCCGGCCAAAAGCAGGACCGCCAGGGCGGAGAACGCAGAAGGTCCCAGCTCGATAGGGATCAGTGTGCCTACTGCAAGGAGAAGGGCCACTGGGCCAAAGACTGCCCGAAAAAGCCGCGCGGCCCACGCGGCCCAAGGCCACAAACATCCCTCCTTCCAAAGAAGAAGCGGAAGGTGGAGCTCAGCGGAGGATCTTCCGGAGGATCTAGCGGCTCCGAGACACCAGGAACATCCGAAAGCGCTACACCAGAATCTAGCGGAGGCTCTTCCGGAGGATCTAGGCCTACCCTCAACATCGAGGATGAGTATCGCCTCCACGAAACCTCCAAAGAACCGGACGTGTCCCTCGGCAGCACATGGCTCAGCGACTTCCCACAAGCGTGGGCCGAAACCGGCGGCATGGGCCTCGCCGTCCGCCAAGCCCCACTCATTATCCCGCTGAAGGCGACCTCCACACCGGTGTCCATCAAGCAGTACCCGATGAGCCAAGAGGCGAGGCTCGGGATTAAGCCGCACATTCAGCGCCTCCTCGATCAAGGCATTCTCGTGCCGTGCCAATCCCCGTGGAATACACCACTCCTCCCGGTCAAAAAGCCGGGCACCAACGACTATCGCCCGGTCCAAGATCTCCGCGAGGTCAACAAGCGCGTGGAAGACATCCACCCGACCGTCCCGAACCCGTATAATCTGCTCTCCGGGCTCCCACCATCCCACCAGTGGTATACAGTGCTGGACCTCAAAGACGCCTTCTTCTGTCTCCGCCTCCACCCAACAAGCCAGCCGCTCTTCGCCTTCGAGTGGCGCGACCCGGAGATGGGCATCTCCGGCCAACTGACATGGACACGCCTCCCGCAAGGCTTCAAGAACAGCCCGACACTCTTCAACGAGGCGCTCCATAGGGACCTCGCGGATTTTCGCATCCAGCATCCGGACCTCATCCTCCTCCAGTAT**gcc**GATGATCTCCTCCTCGCCGCGACCTCCGAGCTGGATTGTCAACAAGGCACACGCGCGCTCCTCCAAACACTCGGGAACCTCGGCTATCGCGCGTCCGCGAAAAAGGCCCAAATCTGCCAGAAGCAAGTGAAGTACCTCGGGTATCTGCTCAAGGAAGGCCAACGCTGGCTCACCGAAGCGCGCAAAGAAACAGTGATGGGGCAACCGACACCGAAAACACCACGCCAGCTGCGCGAGTTTCTCGGCAAAGCCGGCTTCTGTCGCCTCTTCATCCCGGGCTTTGCCGAGATGGCCGCGCCACTCTACCCACTCACCAAGCCGGGCACACTGTTTAACTGGGGGCCGGATCAGCAGAAAGCCTACCAAGAGATCAAACAAGCGCTCCTCACCGCCCCAGCGCTCGGGCTCCCAGATCTCACAAAGCCGTTCGAGCTGTTCGTCGATGAGAAGCAAGGCTACGCGAAGGGCGTGCTCACACAGAAGCTCGGCCCGTGGAGGAGGCCAGTGGCCTATCTCTCCAAAAAACTCGATCCAGTGGCCGCCGGCTGGCCACCGTGTCTGCGCATGGTCGCCGCGATTGCCGTGCTCACAAAGGATGCCGGCAAACTCACAATGGGCCAGCCGCTGGTGATCCTCGCGCCACATGCCGTGGAAGCCCTCGTCAAACAGCCGCCGGATAGGTGGCTCTCCAATGCGCGCATGACCCATTACCAAGCGCTCCTCCTCGACACCGATCGCGTCCAGTTCGGCCCAGTGGTCGCCCTCAATCCGGCGACACTGCTGCCACTCCCAGAGGAGGGCCTCCAACACAACTGTCTGGATATTCTCGCGGAAGCGCATGGCACAAGGCCAGACCTCACAGATCAACCGCTCAGCGGCGGCAGCAAAAGGACCGCCGATTCCCAACATTCCACCCCACCAAAAACCAAGAGGAAGGTGGGCTCTGGCCCAAAGAAGAAGAGGAAGGTCTGAGCGGCCGCGGTACGCTGAAATCACCAGTCTCTCTCTACAAATCTATCTCTCTCTATTTTCTCCATAAATAATGTGTGAGTAGTTTCCCGATAAGGGAAATTAGGGTTCTTATAGGGTTTCGCTCATGTGTTGAGCATATAAGAAACCCTTAGTATGTATTTGTATTTGTAAAATACTTCTATCAATAAAATTTCTAATTCCTAAAACCAAAATCCAGTACTAAAATCCAGATCTCCTAAAGTCCCTATAGATCTTTGTCGTGAATATAAACCAGACACGAGACGACTAAACCTGGAGCCCAGACGCCGTTCGAAGCTAGAAGTACCGCTTAGGCAGGAGGCCGTTAGGGAAAAGATGCTAAGGCAGGGTTGGTTACGTTGACTCCCCCGTAGGTTTGGTTTAAATATGATGAAGTGGACGGAAGGAAGGAGGAAGACAAGGAAGGATAAGGTTGCAGGCCCTGTGCAAGGTAAGAAGATGGAAATTTGATAGAGGTACGCTACTATACTTATACTATACGCTAAGGGAATGCTTGTATTTATACCCTATACCCCCTAATAACCCCTTATCAATTTAAGAAATAATCCGCATAAGCCCCCGCTTAAAAATTGGTATCAGAGCCATGAATAGGTCTATGACCAAAACTCAAGAGGATAAAACCTCACCAAAATACGAAAGAGTTCTTAACTCTAAAGATAAAAGATCTTTCAAGATCAAAACTAGTTCCCTCACACCGGTGACGGGGATCGCATGCGATATCTCGAGATCTAGCTTGGCGTAATCATGGTCATAGCTGTTTCCTGTGTGAAATTGTTATCCGCTCACAATTCCACACAACATACGAGCCGGAAGCATAAAGTGTAAAGCCTGGGGTGCCTAATGAGTGAGCTAACTCACATTAATTGCGTTGCGCTCACTGCCCGCTTTCCAGTCGGGAAACCTGTCGTGCCAGCTGCATTAATGAATCGGCCAACGCGCGGGGAGAGGCGGTTTGCGTATTGGGCGCTCTTCCGCTTCCTCGCTCACTGACTCGCTGCGCTCGGTCGTTCGGCTGCGGCGAGCGGTATCAGCTCACTCAAAGGCGGTAATACGGTTATCCACAGAATCAGGGGATAACGCAGGAAAGAACATGTGAGCAAAAGGCCAGCAAAAGGCCAGGAACCGTAAAAAGGCCGCGTTGCTGGCGTTTTTCCATAGGCTCCGCCCCCCTGACGAGCATCACAAAAATCGACGCTCAAGTCAGAGGTGGCGAAACCCGACAGGACTATAAAGATACCAGGCGTTTCCCCCTGGAAGCTCCCTCGTGCGCTCTCCTGTTCCGACCCTGCCGCTTACCGGATACCTGTCCGCCTTTCTCCCTTCGGGAAGCGTGGCGCTTTCTCATAGCTCACGCTGTAGGTATCTCAGTTCGGTGTAGGTCGTTCGCTCCAAGCTGGGCTGTGTGCACGAACCCCCCGTTCAGCCCGACCGCTGCGCCTTATCCGGTAACTATCGTCTTGAGTCCAACCCGGTAAGACACGACTTATCGCCACTGGCAGCAGCCACTGGTAACAGGATTAGCAGAGCGAGGTATGTAGGCGGTGCTACAGAGTTCTTGAAGTGGTGGCCTAACTACGGCTACACTAGAAGAACAGTATTTGGTATCTGCGCTCTGCTGAAGCCAGTTACCTTCGGAAAAAGAGTTGGTAGCTCTTGATCCGGCAAACAAACCACCGCTGGTAGCGGTGGTTTTTTTGTTTGCAAGCAGCAGATTACGCGCAGAAAAAAAGGATCTCAAGAAGATCCTTTGATCTTTTCTACGGGGTCTGACGCTCAGTGGAACGAAAACTCACGTTAAGGGATTTTGGTCATGAGATTATCAAAAAGGATCTTCACCTAGATCCTTTTAAATTAAAAATGAAGTTTTAAATCAATCTAAAGTATATATGAGTAAACTTGGTCTGACAGTTACCAATGCTTAATCAGTGAGGCACCTATCTCAGCGATCTGTCTATTTCGTTCATCCATAGTTGCCTGACTCCCCGTCGTGTAGATAACTACGATACGGGAGGGCTTACCATCTGGCCCCAGTGCTGCAATGATACCGCGAGACCCACGCTCACCGGCTCCAGATTTATCAGCAATAAACCAGCCAGCCGGAAGGGCCGAGCGCAGAAGTGGTCCTGCAACTTTATCCGCCTCCATCCAGTCTATTAATTGTTGCCGGGAAGCTAGAGTAAGTAGTTCGCCAGTTAATAGTTTGCGCAACGTTGTTGCCATTGCTACAGGCATCGTGGTGTCACGCTCGTCGTTTGGTATGGCTTCATTCAGCTCCGGTTCCCAACGATCAAGGCGAGTTACATGATCCCCCATGTTGTGCAAAAAAGCGGTTAGCTCCTTCGGTCCTCCGATCGTTGTCAGAAGTAAGTTGGCCGCAGTGTTATCACTCATGGTTATGGCAGCACTGCATAATTCTCTTACTGTCATGCCATCCGTAAGATGCTTTTCTGTGACTGGTGAGTACTCAACCAAGTCATTCTGAGAATAGTGTATGCGGCGACCGAGTTGCTCTTGCCCGGCGTCAATACGGGATAATACCGCGCCACATAGCAGAACTTTAAAAGTGCTCATCATTGGAAAACGTTCTTCGGGGCGAAAACTCTCAAGGATCTTACCGCTGTTGAGATCCAGTTCGATGTAACCCACTCGTGCACCCAACTGATCTTCAGCATCTTTTACTTTCACCAGCGTTTCTGGGTGAGCAAAAACAGGAAGGCAAAATGCCGCAAAAAAGGGAATAAGGGCGACACGGAAATGTTGAATACTCATACTCTTCCTTTTTCAATATTATTGAAGCATTTATCAGGGTTATTGTCTCATGAGCGGATACATATTTGAATGTATTTAGAAAAATAAACAAATAGGGGTTCCGCGCACATTTCCCCGAAAAGTGCCACCTGCCAGTGCCAAGCTAATTC

**CMPE-ePPEplus**

GAGCTCGGTACCTGACCCGGTCGTGCCCCTCTCTAGAGATAATGAGCATTGCATGTCTAAGTTATAAAAAATTACCACATATTTTTTTTGTCACACTTGTTTGAAGTGCAGTTTATCTATCTTTATACATATATTTAAACTTTACTCTACGAATAATATAATCTATAGTACTACAATAATATCAGTGTTTTAGAGAATCATATAAATGAACAGTTAGACATGGTCTAAAGGACAATTGAGTATTTTGACAACAGGACTCTACAGTTTTATCTTTTTAGTGTGCATGTGTTCTCCTTTTTTTTTGCAAATAGCTTCACCTATATAATACTTCATCCATTTTATTAGTACATCCATTTAGGGTTTAGGGTTAATGGTTTTTATAGACTAATTTTTTTAGTACATCTATTTTATTCTATTTTAGCCTCTAAATTAAGAAAACTAAAACTCTATTTTAGTTTTTTTATTTAATAATTTAGATATAAAATAGAATAAAATAAAGTGACTAAAAATTAAACAAATACCCTTTAAGAAATTAAAAAAACTAAGGAAACATTTTTCTTGTTTCGAGTAGATAATGCCAGCCTGTTAAACGCCGTCGACGAGTCTAACGGACACCAACCAGCGAACCAGCAGCGTCGCGTCGGGCCAAGCGAAGCAGACGGCACGGCATCTCTGTCGCTGCCTCTGGACCCCTCTCGATCGAGAGTTCCGCTCCACCGTTGGACTTGCTCCGCTGTCGGCATCCAGAAATTGCGTGGCGGAGCGGCAGACGTGAGCCGGCACGGCAGGCGGCCTCCTCCTCCTCTCACGGCACCGGCAGCTACGGGGGATTCCTTTCCCACCGCTCCTTCGCTTTCCCTTCCTCGCCCGCCGTAATAAATAGACACCCCCTCCACACCCTCTTTCCCCAACCTCGTGTTGTTCGGAGCGCACACACACACAACCAGATCTCCCCCAAATCCACCCGTCGGCACCTCCGCTTCAAGGTACGCCGCTCGTCCTCCCCCCCCCCCCCTCTCTACCTTCTCTAGATCGGCGTTCCGGTCCATGGTTAGGGCCCGGTAGTTCTACTTCTGTTCATGTTTGTGTTAGATCCGTGTTTGTGTTAGATCCGTGCTGCTAGCGTTCGTACACGGATGCGACCTGTACGTCAGACACGTTCTGATTGCTAACTTGCCAGTGTTTCTCTTTGGGGAATCCTGGGATGGCTCTAGCCGTTCCGCAGACGGGATCGATTTCATGATTTTTTTTGTTTCGTTGCATAGGGTTTGGTTTGCCCTTTTCCTTTATTTCAATATATGCCGTGCACTTGTTTGTCGGGTCATCTTTTCATGCTTTTTTTTGTCTTGGTTGTGATGATGTGGTCTGGTTGGGCGGTCGTTCTAGATCGGAGTAGAATTAATTCTGTTTCAAACTACCTGGTGGATTTATTAATTTTGGATCTGTATGTGTGTGCCATACATATTCATAGTTACGAATTGAAGATGATGGATGGAAATATCGATCTAGGATAGGTATACATGTTGATGCGGGTTTTACTGATGCATATACAGAGATGCTTTTTGTTCGCTTGGTTGTGATGATGTGGTGTGGTTGGGCGGTCGTTCATTCGTTCTAGATCGGAGTAGAATACTGTTTCAAACTACCTGGTGTATTTATTAATTTTGGAACTGTATGTGTGTGTCATACATCTTCATAGTTACGAGTTTAAGATGGATGGAAATATCGATCTAGGATAGGTATACATGTTGATGTGGGTTTTACTGATGCATATACATGATGGCATATGCAGCATCTATTCATATGCTCTAACCTTGAGTACCTATCTATTATAATAAACAAGTATGTTTTATAATTATTTTGATCTTGATATACTTGGATGATGGCATATGCAGCAGCTATATGTGGATTTTTTTAGCCCTGCCTTCATACGCTATTTATTTGCTTGGTACTGTTTCTTTTGTCGATGCTCACCCTGTTGTTTGGTGTTACTTCTGCAAAGCTTATGATGGACCACTACCTCGACATCAGGCTCAGGCCAGACCCAGAGTTCCCACCAGCCCAGCTCATGTCCGTCCTCTTCGGCAAGCTCCACCAGGCCCTCGTGGCCCAGGGCGGCGACAGGATCGGCGTGTCCTTCCCAGACCTCGACGAGTCCAGGTCCAGGCTCGGCGAGAGGCTCCGCATCCACGCCTCCGCCGACGACCTCAGGGCCCTCCTCGCCAGGCCGTGGCTGGAGGGCCTCAGGGACCACCTCCAGTTCGGCGAGCCAGCCGTGGTGCCACACCCAACCCCATACAGGCAAGTGTCCAGGGTGCAAGCCAAGTCCAACCCAGAGAGGCTCAGGAGGAGGCTCATGAGGAGGCACGACCTCTCCGAGGAAGAGGCCAGGAAGCGCATCCCAGACACCGTGGCCAGGGCCCTCGACCTCCCATTCGTGACCCTCAGGTCCCAGTCCACCGGCCAGCACTTCCGCCTCTTCATCAGGCACGGCCCACTCCAGGTGACCGCCGAGGAGGGCGGCTTTACCTGCTACGGCCTCTCCAAGGGCGGCTTCGTGCCGTGGTTCGGCTCCGGCGCCACCAACTTCTCCCTCCTCAAGCAAGCCGGCGACGTGGAGGAGAACCCAGGCCCAATGCCAGCCGCCAAGCGCGTCAAGCTGGACGGCGGAAAGCGCACCGCCGACGGATCCGAGTTCGAGAGCCCTAAGAAAAAGAGAAAAGTGGACAAGAAGTACTCGATCGGCCTCGATATTGGGACTAACTCTGTTGGCTGGGCCGTGATCACCGACGAGTACAAGGTGCCCTCAAAGAAGTTCAAGGTCCTGGGCAACACCGATCGGCATTCCATCAAGAAGAATCTCATTGGCGCTCTCCTGTTCGACAGCGGCGAGACGGCTGAGGCTACGCGGCTCAAGCGCACCGCCCGCAGGCGGTACACGCGCAGGAAGAATCGCATCTGCTACCTGCAGGAGATTTTCTCCAACGAGATGGCGAAGGTTGACGATTCTTTCTTCCACAGGCTGGAGGAGTCATTCCTCGTGGAGGAGGATAAGAAGCACGAGCGGCATCCAATCTTCGGCAACATTGTCGACGAGGTTGCCTACCACGAGAAGTACCCTACGATCTACCATCTGCGGAAGAAGCTCGTGGACTCCACAGATAAGGCGGACCTCCGCCTGATCTACCTCGCTCTGGCCCACATGATTAAGTTCAGGGGCCATTTCCTGATCGAGGGGGATCTCAACCCGGACAATAGCGATGTTGACAAGCTGTTCATCCAGCTCGTGCAGACGTACAACCAGCTCTTCGAGGAGAACCCCATTAATGCGTCAGGCGTCGACGCGAAGGCTATCCTGTCCGCTAGGCTCTCGAAGTCTCGGaagCTCGAGAACCTGATCGCCCAGCTGCCGGGCGAGAAGAAGAACGGCCTGTTCGGGAATCTCATTGCGCTCAGCCTGGGGCTCACGCCCAACTTCAAGTCGAATTTCGATCTCGCTGAGGACGCCAAGCTGCAGCTCTCCAAGGACACATACGACGATGACCTGGATAACCTCCTGGCCCAGATCGGCGATCAGTACGCGGACCTGTTCCTCGCTGCCAAGAATCTGTCGGACGCCATCCTCCTGTCTGATATTCTCAGGGTGAACACCGAGATTACGAAGGCTCCGCTCTCAGCCTCCATGATCAAGCGCTACGACGAGCACCATCAGGATCTGACCCTCCTGAAGGCGCTGGTCAGGCAGCAGCTCCCCGAGAAGTACAAGGAGATCTTCTTCGATCAGTCGAAGAACGGCTACGCTGGGTACATTGACGGCGGGGCCTCTCAGGAGGAGTTCTACAAGTTCATCAAGCCGATTCTGGAGAAGATGGACGGCACGGAGGAGCTGCTGGTGAAGCTCaagCGCGAGGACCTCCTGAGGAAGCAGCGGACATTCGATAACGGCAGCATCCCACACCAGATTCATCTCGGGGAGCTGCACGCTATCCTGAGGAGGCAGGAGGACTTCTACCCTTTCCTCAAGGATAACCGCGAGAAGATCGAGAAGATTCTGACTTTCAGGATCCCGTACTACGTCGGCCCACTCGCTAGGGGCAACTCCCGCTTCGCTTGGATGACCCGCAAGTCAGAGGAGACGATCACGCCGTGGAACTTCGAGGAGGTGGTCGACAAGGGCGCTAGCGCTCAGTCGTTCATCGAGAGGATGACGAATTTCGACAAGAACCTGCCAAATGAGAAGGTGCTCCCTAAGCACTCGCTCCTGTACGAGTACTTCACAGTCTACAACGAGCTGACTAAGGTGAAGTATGTGACCGAGGGCATGAGGAAGCCGGCTTTCCTGTCTGGGGAGCAGAAGAAGGCCATCGTGGACCTCCTGTTCAAGACCAACCGGAAGGTCACGGTTAAGCAGCTCAAGGAGGACTACTTCAAGAAGATTGAGTGCTTCGATTCGGTCGAGATCTCTGGCGTTGAGGACCGCTTCAACGCCTCCCTGGGGACCTACCACGATCTCCTGAAGATCATTAAGGATAAGGACTTCCTGGACAACGAGGAGAATGAGGATATCCTCGAGGACATTGTGCTGACACTCACTCTGTTCGAGGACCGGGAGATGATCGAGGAGCGCCTGAAGACTTACGCCCATCTCTTCGATGACAAGGTCATGAAGCAGCTCAAGAGGAGGAGGTACACCGGCTGGGGGAGGCTGAGCAGGAAGCTCATCAACGGCATTCGGGACAAGCAGTCCGGGAAGACGATCCTCGACTTCCTGAAGAGCGATGGCTTCGCGAACCGCAATTTCATGCAGCTGATTCACGATGACAGCCTCACATTCAAGGAGGATATCCAGAAGGCTCAGGTGAGCGGCCAGGGGGACTCGCTGCACGAGCATATCGCGAACCTCGCTGGCTCGCCAGCTATCAAGAAGGGGATTCTGCAGACCGTGAAGGTTGTGGACGAGCTGGTGAAGGTCATGGGCAGGCACAAGCCTGAGAACATCGTCATTGAGATGGCCCGGGAGAATCAGACCACGCAGAAGGGCCAGAAGAACTCACGCGAGAGGATGAAGAGGATCGAGGAGGGCATTAAGGAGCTGGGGTCCCAGATCCTCAAGGAGCACCCGGTGGAGAACACGCAGCTGCAGAATGAGAAGCTCTACCTGTACTACCTCCAGAATGGCCGCGATATGTATGTGGACCAGGAGCTGGATATTAACAGGCTCAGCGATTACGACGTCGATGCCATCGTTCCACAGTCATTCCTGAAGGATGACTCCATTGACAACAAGGTCCTCACCAGGTCGGACAAGAACCGGGGCAAGTCTGATAATGTTCCTTCAGAGGAGGTCGTTAAGAAGATGAAGAACTACTGGCGCCAGCTCCTGAATGCCAAGCTGATCACGCAGCGGAAGTTCGATAACCTCACAAAGGCTGAGAGGGGCGGGCTCTCTGAGCTGGACAAGGCGGGCTTCATCAAGAGGCAGCTGGTCGAGACACGGCAGATCACTAAGCACGTTGCGCAGATTCTCGACTCACGGATGAACACTAAGTACGATGAGAATGACAAGCTGATCCGCGAGGTGAAGGTCATCACCCTGAAGTCAAAGCTCGTCTCCGACTTCAGGAAGGATTTCCAGTTCTACAAGGTTCGGGAGATCAACAATTACCACCATGCCCATGACGCGTACCTGAACGCGGTGGTCGGCACAGCTCTGATCAAGAAGTACCCAAAGCTCGAGAGCGAGTTCGTGTACGGGGACTACAAGGTTTACGATGTGAGGAAGATGATCGCCAAGTCGGAGCAGGAGATTGGCAAGGCTACCGCCAAGTACTTCTTCTACTCTAACATTATGAATTTCTTCAAGACAGAGATCACTCTGGCCAATGGCGAGATCCGGAAGCGCCCCCTCATCGAGACGAACGGCGAGACGGGGGAGATCGTGTGGGACAAGGGCAGGGATTTCGCGACCGTCAGGAAGGTTCTCTCCATGCCACAAGTGAATATCGTCAAGAAGACAGAGGTCCAGACTGGCGGGTTCTCTAAGGAGTCAATTCTGCCTAAGCGGAACAGCGACAAGCTCATCGCCCGCAAGAAGGACTGGGATCCGAAGAAGTACGGCGGGTTCGACAGCCCCACTGTGGCCTACTCGGTCCTGGTTGTGGCGAAGGTTGAGAAGGGCAAGTCCAAGAAGCTCAAGAGCGTGAAGGAGCTGCTGGGGATCACGATTATGGAGCGCTCCAGCTTCGAGAAGAACCCGATCGATTTCCTGGAGGCGAAGGGCTACAAGGAGGTGAAGAAGGACCTGATCATTAAGCTCCCCAAGTACTCACTCTTCGAGCTGGAGAACGGCAGGAAGCGGATGCTGGCTTCCGCTGGCGAGCTGCAGAAGGGGAACGAGCTGGCTCTGCCGTCCAAGTATGTGAACTTCCTCTACCTGGCCTCCCACTACGAGAAGCTCAAGGGCAGCCCCGAGGACAACGAGCAGAAGCAGCTGTTCGTCGAGCAGCACAAGCATTACCTCGACGAGATCATTGAGCAGATTTCCGAGTTCTCCAAGCGCGTGATCCTGGCCGACGCGAATCTGGATAAGGTCCTCTCCGCGTACAACAAGCACCGCGACAAGCCAATCAGGGAGCAGGCTGAGAATATCATTCATCTCTTCACCCTGACGAACCTCGGCGCCCCTGCTGCTTTCAAGTACTTCGACACAACTATCGATCGCAAGAGGTACACAAGCACTAAGGAGGTCCTGGACGCGACCCTCATCCACCAGTCGATTACCGGCCTCTACGAGACGCGCATCGACCTGTCTCAGCTCGGGGGCGACGAATTCTCCGGGAGCGAGACGCCAGGCACCTCCGAGTCGGCCACCCCAGAATCTGCCACAGTGGTGTCCGGCCAAAAGCAGGACCGCCAGGGCGGAGAACGCAGAAGGTCCCAGCTCGATAGGGATCAGTGTGCCTACTGCAAGGAGAAGGGCCACTGGGCCAAAGACTGCCCGAAAAAGCCGCGCGGCCCACGCGGCCCAAGGCCACAAACATCCCTCCTTCCAAAGAAGAAGCGGAAGGTGGAGCTCAGCGGAGGATCTTCCGGAGGATCTAGCGGCTCCGAGACACCAGGAACATCCGAAAGCGCTACACCAGAATCTAGCGGAGGCTCTTCCGGAGGATCTAGGCCTACCCTCAACATCGAGGATGAGTATCGCCTCCACGAAACCTCCAAAGAACCGGACGTGTCCCTCGGCAGCACATGGCTCAGCGACTTCCCACAAGCGTGGGCCGAAACCGGCGGCATGGGCCTCGCCGTCCGCCAAGCCCCACTCATTATCCCGCTGAAGGCGACCTCCACACCGGTGTCCATCAAGCAGTACCCGATGAGCCAAGAGGCGAGGCTCGGGATTAAGCCGCACATTCAGCGCCTCCTCGATCAAGGCATTCTCGTGCCGTGCCAATCCCCGTGGAATACACCACTCCTCCCGGTCAAAAAGCCGGGCACCAACGACTATCGCCCGGTCCAAGATCTCCGCGAGGTCAACAAGCGCGTGGAAGACATCCACCCGACCGTCCCGAACCCGTATAATCTGCTCTCCGGGCTCCCACCATCCCACCAGTGGTATACAGTGCTGGACCTCAAAGACGCCTTCTTCTGTCTCCGCCTCCACCCAACAAGCCAGCCGCTCTTCGCCTTCGAGTGGCGCGACCCGGAGATGGGCATCTCCGGCCAACTGACATGGACACGCCTCCCGCAAGGCTTCAAGAACAGCCCGACACTCTTCAACGAGGCGCTCCATAGGGACCTCGCGGATTTTCGCATCCAGCATCCGGACCTCATCCTCCTCCAGTAT**gcc**GATGATCTCCTCCTCGCCGCGACCTCCGAGCTGGATTGTCAACAAGGCACACGCGCGCTCCTCCAAACACTCGGGAACCTCGGCTATCGCGCGTCCGCGAAAAAGGCCCAAATCTGCCAGAAGCAAGTGAAGTACCTCGGGTATCTGCTCAAGGAAGGCCAACGCTGGCTCACCGAAGCGCGCAAAGAAACAGTGATGGGGCAACCGACACCGAAAACACCACGCCAGCTGCGCGAGTTTCTCGGCAAAGCCGGCTTCTGTCGCCTCTTCATCCCGGGCTTTGCCGAGATGGCCGCGCCACTCTACCCACTCACCAAGCCGGGCACACTGTTTAACTGGGGGCCGGATCAGCAGAAAGCCTACCAAGAGATCAAACAAGCGCTCCTCACCGCCCCAGCGCTCGGGCTCCCAGATCTCACAAAGCCGTTCGAGCTGTTCGTCGATGAGAAGCAAGGCTACGCGAAGGGCGTGCTCACACAGAAGCTCGGCCCGTGGAGGAGGCCAGTGGCCTATCTCTCCAAAAAACTCGATCCAGTGGCCGCCGGCTGGCCACCGTGTCTGCGCATGGTCGCCGCGATTGCCGTGCTCACAAAGGATGCCGGCAAACTCACAATGGGCCAGCCGCTGGTGATCCTCGCGCCACATGCCGTGGAAGCCCTCGTCAAACAGCCGCCGGATAGGTGGCTCTCCAATGCGCGCATGACCCATTACCAAGCGCTCCTCCTCGACACCGATCGCGTCCAGTTCGGCCCAGTGGTCGCCCTCAATCCGGCGACACTGCTGCCACTCCCAGAGGAGGGCCTCCAACACAACTGTCTGGATATTCTCGCGGAAGCGCATGGCACAAGGCCAGACCTCACAGATCAACCGCTCAGCGGCGGCAGCAAAAGGACCGCCGATTCCCAACATTCCACCCCACCAAAAACCAAGAGGAAGGTGGGCTCTGGCCCAAAGAAGAAGAGGAAGGTCTGAGCGGCCGCGGTACGCTGAAATCACCAGTCTCTCTCTACAAATCTATCTCTCTCTATTTTCTCCATAAATAATGTGTGAGTAGTTTCCCGATAAGGGAAATTAGGGTTCTTATAGGGTTTCGCTCATGTGTTGAGCATATAAGAAACCCTTAGTATGTATTTGTATTTGTAAAATACTTCTATCAATAAAATTTCTAATTCCTAAAACCAAAATCCAGTACTAAAATCCAGATCTCCTAAAGTCCCTATAGATCTTTGTCGTGAATATAAACCAGACACGAGACGACTAAACCTGGAGCCCAGACGCCGTTCGAAGCTAGAAGTACCGCTTAGGCAGGAGGCCGTTAGGGAAAAGATGCTAAGGCAGGGTTGGTTACGTTGACTCCCCCGTAGGTTTGGTTTAAATATGATGAAGTGGACGGAAGGAAGGAGGAAGACAAGGAAGGATAAGGTTGCAGGCCCTGTGCAAGGTAAGAAGATGGAAATTTGATAGAGGTACGCTACTATACTTATACTATACGCTAAGGGAATGCTTGTATTTATACCCTATACCCCCTAATAACCCCTTATCAATTTAAGAAATAATCCGCATAAGCCCCCGCTTAAAAATTGGTATCAGAGCCATGAATAGGTCTATGACCAAAACTCAAGAGGATAAAACCTCACCAAAATACGAAAGAGTTCTTAACTCTAAAGATAAAAGATCTTTCAAGATCAAAACTAGTTCCCTCACACCGGTGACGGGGATCGCATGCGATATCTCGAGATCTAGCTTGGCGTAATCATGGTCATAGCTGTTTCCTGTGTGAAATTGTTATCCGCTCACAATTCCACACAACATACGAGCCGGAAGCATAAAGTGTAAAGCCTGGGGTGCCTAATGAGTGAGCTAACTCACATTAATTGCGTTGCGCTCACTGCCCGCTTTCCAGTCGGGAAACCTGTCGTGCCAGCTGCATTAATGAATCGGCCAACGCGCGGGGAGAGGCGGTTTGCGTATTGGGCGCTCTTCCGCTTCCTCGCTCACTGACTCGCTGCGCTCGGTCGTTCGGCTGCGGCGAGCGGTATCAGCTCACTCAAAGGCGGTAATACGGTTATCCACAGAATCAGGGGATAACGCAGGAAAGAACATGTGAGCAAAAGGCCAGCAAAAGGCCAGGAACCGTAAAAAGGCCGCGTTGCTGGCGTTTTTCCATAGGCTCCGCCCCCCTGACGAGCATCACAAAAATCGACGCTCAAGTCAGAGGTGGCGAAACCCGACAGGACTATAAAGATACCAGGCGTTTCCCCCTGGAAGCTCCCTCGTGCGCTCTCCTGTTCCGACCCTGCCGCTTACCGGATACCTGTCCGCCTTTCTCCCTTCGGGAAGCGTGGCGCTTTCTCATAGCTCACGCTGTAGGTATCTCAGTTCGGTGTAGGTCGTTCGCTCCAAGCTGGGCTGTGTGCACGAACCCCCCGTTCAGCCCGACCGCTGCGCCTTATCCGGTAACTATCGTCTTGAGTCCAACCCGGTAAGACACGACTTATCGCCACTGGCAGCAGCCACTGGTAACAGGATTAGCAGAGCGAGGTATGTAGGCGGTGCTACAGAGTTCTTGAAGTGGTGGCCTAACTACGGCTACACTAGAAGAACAGTATTTGGTATCTGCGCTCTGCTGAAGCCAGTTACCTTCGGAAAAAGAGTTGGTAGCTCTTGATCCGGCAAACAAACCACCGCTGGTAGCGGTGGTTTTTTTGTTTGCAAGCAGCAGATTACGCGCAGAAAAAAAGGATCTCAAGAAGATCCTTTGATCTTTTCTACGGGGTCTGACGCTCAGTGGAACGAAAACTCACGTTAAGGGATTTTGGTCATGAGATTATCAAAAAGGATCTTCACCTAGATCCTTTTAAATTAAAAATGAAGTTTTAAATCAATCTAAAGTATATATGAGTAAACTTGGTCTGACAGTTACCAATGCTTAATCAGTGAGGCACCTATCTCAGCGATCTGTCTATTTCGTTCATCCATAGTTGCCTGACTCCCCGTCGTGTAGATAACTACGATACGGGAGGGCTTACCATCTGGCCCCAGTGCTGCAATGATACCGCGAGACCCACGCTCACCGGCTCCAGATTTATCAGCAATAAACCAGCCAGCCGGAAGGGCCGAGCGCAGAAGTGGTCCTGCAACTTTATCCGCCTCCATCCAGTCTATTAATTGTTGCCGGGAAGCTAGAGTAAGTAGTTCGCCAGTTAATAGTTTGCGCAACGTTGTTGCCATTGCTACAGGCATCGTGGTGTCACGCTCGTCGTTTGGTATGGCTTCATTCAGCTCCGGTTCCCAACGATCAAGGCGAGTTACATGATCCCCCATGTTGTGCAAAAAAGCGGTTAGCTCCTTCGGTCCTCCGATCGTTGTCAGAAGTAAGTTGGCCGCAGTGTTATCACTCATGGTTATGGCAGCACTGCATAATTCTCTTACTGTCATGCCATCCGTAAGATGCTTTTCTGTGACTGGTGAGTACTCAACCAAGTCATTCTGAGAATAGTGTATGCGGCGACCGAGTTGCTCTTGCCCGGCGTCAATACGGGATAATACCGCGCCACATAGCAGAACTTTAAAAGTGCTCATCATTGGAAAACGTTCTTCGGGGCGAAAACTCTCAAGGATCTTACCGCTGTTGAGATCCAGTTCGATGTAACCCACTCGTGCACCCAACTGATCTTCAGCATCTTTTACTTTCACCAGCGTTTCTGGGTGAGCAAAAACAGGAAGGCAAAATGCCGCAAAAAAGGGAATAAGGGCGACACGGAAATGTTGAATACTCATACTCTTCCTTTTTCAATATTATTGAAGCATTTATCAGGGTTATTGTCTCATGAGCGGATACATATTTGAATGTATTTAGAAAAATAAACAAATAGGGGTTCCGCGCACATTTCCCCGAAAAGTGCCACCTGCCAGTGCCAAGCTAATTC

**pUC57-CmYLCV**

TCGCGCGTTTCGGTGATGACGGTGAAAACCTCTGACACATGCAGCTCCCGGAGACGGTCACAGCTTGTCTGTAAGCGGATGCCGGGAGCAGACAAGCCCGTCAGGGCGCGTCAGCGGGTGTTGGCGGGTGTCGGGGCTGGCTTAACTATGCGGCATCAGAGCAGATTGTACTGAGAGTGCACCATATGCGGTGTGAAATACCGCACAGATGCGTAAGGAGAAAATACCGCATCAGGCGCCATTCGCCATTCAGGCTGCGCAACTGTTGGGAAGGGCGATCGGTGCGGGCCTCTTCGCTATTACGCCAGCTGGCGAAAGGGGGATGTGCTGCAAGGCGATTAAGTTGGGTAACGCCAGGGTTTTCCCAGTCACGACGTTGTAAAACGACGGCCAGTGAATTCTGGCAGACATACTGTCCCACAAATGAAGATGGAATCTGTAAAAGAAAACGCGTGAAATAATGCGTCTGACAAAGGTTAGGTCGGCTGCCTTTAATCAATACCAAAGTGGTCCCTACCACGATGGAAAAACTGTGCAGTCGGTTTGGCTTTTTCTGACGAACAAATAAGATTCGTGGCCGACAGGTGGGGGTCCACCATGTGAAGGCATCTTCAGACTCCAATAATGGAGCAATGACGTAAGGGCTTACGAAATAAGTAAGGGTAGTTTGGGAAATGTCCACTCACCCGTCAGTCTATAAATACTTAGCCCCTCCCTCATTGTTAAGGGAGCAAAATCTCAGAGAGATAGTCCTAGAGAGAGAAAGAGAGCAAGTAGCCTAGAAGTAGTCAAGGCGGCGAAGTATTCAGGCACGTGGCCAGGAAGAAGAAAAGCCAAGACGACGAAAACAGGTAAGAGCTAAGCTTCCTGCAGGGTCTCGTTCACTGCCGTATAGGCAGGTTTAAGAGCTATGCTGGAAACAGCATAGCAAGTTTAAATAAGGCTAGTCCGTTATCAACTTGAAAAAGTGGCACCGAGTCGGTGCGGTCTCCGCGGTTCTATCTAGTTACGCGTTAAACCAACTAGAAGTTCACTGCCGTATAGGCAGGGATCCTTTCTCCATAATAATGTGTGAGTAGTTCCCAGATAAGGGAATTAGGGTTCCTATAGGGTTTCGCTCATGTGTTGAGCATATAAGAAACCCTTAGTATGTATTTGTATTTGTAAAATACTTCTATCAATAAAATTTCTAATTCCTAAAACCAAAATCCAGTACTAAAATCCAGATCAAGCTTGGCGTAATCATGGTCATAGCTGTTTCCTGTGTGAAATTGTTATCCGCTCACAATTCCACACAACATACGAGCCGGAAGCATAAAGTGTAAAGCCTGGGGTGCCTAATGAGTGAGCTAACTCACATTAATTGCGTTGCGCTCACTGCCCGCTTTCCAGTCGGGAAACCTGTCGTGCCAGCTGCATTAATGAATCGGCCAACGCGCGGGGAGAGGCGGTTTGCGTATTGGGCGCTCTTCCGCTTCCTCGCTCACTGACTCGCTGCGCTCGGTCGTTCGGCTGCGGCGAGCGGTATCAGCTCACTCAAAGGCGGTAATACGGTTATCCACAGAATCAGGGGATAACGCAGGAAAGAACATGTGAGCAAAAGGCCAGCAAAAGGCCAGGAACCGTAAAAAGGCCGCGTTGCTGGCGTTTTTCCATAGGCTCCGCCCCCCTGACGAGCATCACAAAAATCGACGCTCAAGTCAGAGGTGGCGAAACCCGACAGGACTATAAAGATACCAGGCGTTTCCCCCTGGAAGCTCCCTCGTGCGCTCTCCTGTTCCGACCCTGCCGCTTACCGGATACCTGTCCGCCTTTCTCCCTTCGGGAAGCGTGGCGCTTTCTCATAGCTCACGCTGTAGGTATCTCAGTTCGGTGTAGGTCGTTCGCTCCAAGCTGGGCTGTGTGCACGAACCCCCCGTTCAGCCCGACCGCTGCGCCTTATCCGGTAACTATCGTCTTGAGTCCAACCCGGTAAGACACGACTTATCGCCACTGGCAGCAGCCACTGGTAACAGGATTAGCAGAGCGAGGTATGTAGGCGGTGCTACAGAGTTCTTGAAGTGGTGGCCTAACTACGGCTACACTAGAAGAACAGTATTTGGTATCTGCGCTCTGCTGAAGCCAGTTACCTTCGGAAAAAGAGTTGGTAGCTCTTGATCCGGCAAACAAACCACCGCTGGTAGCGGTGGTTTTTTTGTTTGCAAGCAGCAGATTACGCGCAGAAAAAAAGGATCTCAAGAAGATCCTTTGATCTTTTCTACGGGGTCTGACGCTCAGTGGAACGAAAACTCACGTTAAGGGATTTTGGTCATGAGATTATCAAAAAGGATCTTCACCTAGATCCTTTTAAATTAAAAATGAAGTTTTAAATCAATCTAAAGTATATATGAGTAAACTTGGTCTGACAGTTACCAATGCTTAATCAGTGAGGCACCTATCTCAGCGATCTGTCTATTTCGTTCATCCATAGTTGCCTGACTCCCCGTCGTGTAGATAACTACGATACGGGAGGGCTTACCATCTGGCCCCAGTGCTGCAATGATACCGCGAGAACCACGCTCACCGGCTCCAGATTTATCAGCAATAAACCAGCCAGCCGGAAGGGCCGAGCGCAGAAGTGGTCCTGCAACTTTATCCGCCTCCATCCAGTCTATTAATTGTTGCCGGGAAGCTAGAGTAAGTAGTTCGCCAGTTAATAGTTTGCGCAACGTTGTTGCCATTGCTACAGGCATCGTGGTGTCACGCTCGTCGTTTGGTATGGCTTCATTCAGCTCCGGTTCCCAACGATCAAGGCGAGTTACATGATCCCCCATGTTGTGCAAAAAAGCGGTTAGCTCCTTCGGTCCTCCGATCGTTGTCAGAAGTAAGTTGGCCGCAGTGTTATCACTCATGGTTATGGCAGCACTGCATAATTCTCTTACTGTCATGCCATCCGTAAGATGCTTTTCTGTGACTGGTGAGTACTCAACCAAGTCATTCTGAGAATAGTGTATGCGGCGACCGAGTTGCTCTTGCCCGGCGTCAATACGGGATAATACCGCGCCACATAGCAGAACTTTAAAAGTGCTCATCATTGGAAAACGTTCTTCGGGGCGAAAACTCTCAAGGATCTTACCGCTGTTGAGATCCAGTTCGATGTAACCCACTCGTGCACCCAACTGATCTTCAGCATCTTTTACTTTCACCAGCGTTTCTGGGTGAGCAAAAACAGGAAGGCAAAATGCCGCAAAAAAGGGAATAAGGGCGACACGGAAATGTTGAATACTCATACTCTTCCTTTTTCAATATTATTGAAGCATTTATCAGGGTTATTGTCTCATGAGCGGATACATATTTGAATGTATTTAGAAAAATAAACAAATAGGGGTTCCGCGCACATTTCCCCGAAAAGTGCCACCTGACGTCTAAGAAACCATTATTATCATGACATTAACCTATAAAAATAGGCGTATCACGAGGCCCTTTCGTC

**pB-CMPE-ePPEplus**

TAAACGCTCTTTTCTCTTAGGTTTACCCGCCAATATATCCTGTCAAACACTGATAGTTTAAACTGAAGGCGGGAAACGACAATCTGATCCAAGCTCAAGCTGCTCTAGCATTCGCCATTCAGGCTGCGCAACTGTTGGGAAGGGCGATCGGTGCGGGCCTCTTCGCTATTACGCCAGCTGGCGAAAGGGGGATGTGCTGCAAGGCGATTAAGTTGGGTAACGCCAGGGTTTTCCCAGTCACGACGTTGTAAAACGACGGCCAGTGCCTGGCAGACATACTGTCCCACAAATGAAGATGGAATCTGTAAAAGAAAACGCGTGAAATAATGCGTCTGACAAAGGTTAGGTCGGCTGCCTTTAATCAATACCAAAGTGGTCCCTACCACGATGGAAAAACTGTGCAGTCGGTTTGGCTTTTTCTGACGAACAAATAAGATTCGTGGCCGACAGGTGGGGGTCCACCATGTGAAGGCATCTTCAGACTCCAATAATGGAGCAATGACGTAAGGGCTTACGAAATAAGTAAGGGTAGTTTGGGAAATGTCCACTCACCCGTCAGTCTATAAATACTTAGCCCCTCCCTCATTGTTAAGGGAGCAAAATCTCAGAGAGATAGTCCTAGAGAGAGAAAGAGAGCAAGTAGCCTAGAAGTAGTCAAGGCGGCGAAGTATTCAGGCACGTGGCCAGGAAGAAGAAAAGCCAAGACGACGAAAACAGGTAAGAGCTAAGCTTCCTGCAGGGTCTCGTTCACTGCCGTATAGGCAGGTTTAAGAGCTATGCTGGAAACAGCATAGCAAGTTTAAATAAGGCTAGTCCGTTATCAACTTGAAAAAGTGGCACCGAGTCGGTGCGGTCTCCGCGGTTCTATCTAGTTACGCGTTAAACCAACTAGAAGTTCACTGCCGTATAGGCAGGGATCCTTTCTCCATAATAATGTGTGAGTAGTTCCCAGATAAGGGAATTAGGGTTCCTATAGGGTTTCGCTCATGTGTTGAGCATATAAGAAACCCTTAGTATGTATTTGTATTTGTAAAATACTTCTATCAATAAAATTTCTAATTCCTAAAACCAAAATCCAGTACTAAAATCCAGATCAAGCTTGATCGTTCAAACATTTGGCAATAAAGTTTCTTAAGATTGAATCCTGTTGCCGGTCTTGCGATGATTATCATATAATTTCTGTTGAATTACGTTAAGCATGTAATAATTAACATGTAATGCATGACGTTATTTATGAGATGGGTTTTTATGATTAGAGTCCCGCAATTATACATTTAATACGCGATAGAAAACAAAATATAGCGCGCAAACTAGGATAAATTATCGCGCGCGGTGTCATCTATGTTACTAGATCTAAGATCCCCGGGTACCGAGCTCGAATTTCCCCGATCGTTCAAACATTTGGCAATAAAGTTTCTTAAGATTGAATCCTGTTGCCGGTCTTGCGATGATTATCATATAATTTCTGTTGAATTACGTTAAGCATGTAATAATTAACATGTAATGCATGACGTTATTTATGAGATGGGTTTTTATGATTAGAGTCCCGCAATTATACATTTAATACGCGATAGAAAACAAAATATAGCGCGCAAACTAGGATAAATTATCGCGCGCGGTGTCATCTATGTTACTAGATCGGGCATGCCTGCAGTGCAGCGTGACCCGGTCGTGCCCCTCTCTAGAGATAATGAGCATTGCATGTCTAAGTTATAAAAAATTACCACATATTTTTTTTGTCACACTTGTTTGAAGTGCAGTTTATCTATCTTTATACATATATTTAAACTTTACTCTACGAATAATATAATCTATAGTACTACAATAATATCAGTGTTTTAGAGAATCATATAAATGAACAGTTAGACATGGTCTAAAGGACAATTGAGTATTTTGACAACAGGACTCTACAGTTTTATCTTTTTAGTGTGCATGTGTTCTCCTTTTTTTTTGCAAATAGCTTCACCTATATAATACTTCATCCATTTTATTAGTACATCCATTTAGGGTTTAGGGTTAATGGTTTTTATAGACTAATTTTTTTAGTACATCTATTTTATTCTATTTTAGCCTCTAAATTAAGAAAACTAAAACTCTATTTTAGTTTTTTTATTTAATAATTTAGATATAAAATAGAATAAAATAAAGTGACTAAAAATTAAACAAATACCCTTTAAGAAATTAAAAAAACTAAGGAAACATTTTTCTTGTTTCGAGTAGATAATGCCAGCCTGTTAAACGCCGTCGACGAGTCTAACGGACACCAACCAGCGAACCAGCAGCGTCGCGTCGGGCCAAGCGAAGCAGACGGCACGGCATCTCTGTCGCTGCCTCTGGACCCCTCTCGAGAGTTCCGCTCCACCGTTGGACTTGCTCCGCTGTCGGCATCCAGAAATTGCGTGGCGGAGCGGCAGACGTGAGCCGGCACGGCAGGCGGCCTCCTCCTCCTCTCACGGCACGGCAGCTACGGGGGATTCCTTTCCCACCGCTCCTTCGCTTTCCCTTCCTCGCCCGCCGTAATAAATAGACACCCCCTCCACACCCTCTTTCCCCAACCTCGTGTTGTTCGGAGCGCACACACACACAACCAGATCTCCCCCAAATCCACCCGTCGGCACCTCCGCTTCAAGGTACGCCGCTCGTCCTCCCCCCCCCCCCCTCTCTACCTTCTCTAGATCGGCGTTCCGGTCCATGGTTAGGGCCCGGTAGTTCTACTTCTGTTCATGTTTGTGTTAGATCCGTGTTTGTGTTAGATCCGTGCTGCTAGCGTTCGTACACGGATGCGACCTGTACGTCAGACACGTTCTGATTGCTAACTTGCCAGTGTTTCTCTTTGGGGAATCCTGGGATGGCTCTAGCCGTTCCGCAGACGGGATCGATTTCATGATTTTTTTTGTTTCGTTGCATAGGGTTTGGTTTGCCCTTTTCCTTTATTTCAATATATGCCGTGCACTTGTTTGTCGGGTCATCTTTTCATGCTTTTTTTTGTCTTGGTTGTGATGATGTGGTCTGGTTGGGCGGTCGTTCTAGATCGGAGTAGAATTCTGTTTCAAACTACCTGGTGGATTTATTAATTTTGGATCTGTATGTGTGTGCCATACATATTCATAGTTACGAATTGAAGATGATGGATGGAAATATCGATCTAGGATAGGTATACATGTTGATGCGGGTTTTACTGATGCATATACAGAGATGCTTTTTGTTCGCTTGGTTGTGATGATGTGGTGTGGTTGGGCGGTCGTTCATTCGTTCTAGATCGGAGTAGAATACTGTTTCAAACTACCTGGTGTATTTATTAATTTTGGAACTGTATGTGTGTGTCATACATCTTCATAGTTACGAGTTTAAGATGGATGGAAATATCGATCTAGGATAGGTATACATGTTGATGTGGGTTTTACTGATGCATATACATGATGGCATATGCAGCATCTATTCATATGCTCTAACCTTGAGTACCTATCTATTATAATAAACAAGTATGTTTTATAATTATTTTGATCTTGATATACTTGGATGATGGCATATGCAGCAGCTATATGTGGATTTTTTTAGCCCTGCCTTCATACGCTATTTATTTGCTTGGTACTGTTTCTTTTGTCGATGCTCACCCTGTTGTTTGGTGTTACTTCTGCAGCCCTAGGCTTAAGTCATGGACCACTACCTCGACATCAGGCTCAGGCCAGACCCAGAGTTCCCACCAGCCCAGCTCATGTCCGTCCTCTTCGGCAAGCTCCACCAGGCCCTCGTGGCCCAGGGCGGCGACAGGATCGGCGTGTCCTTCCCAGACCTCGACGAGTCCAGGTCCAGGCTCGGCGAGAGGCTCCGCATCCACGCCTCCGCCGACGACCTCAGGGCCCTCCTCGCCAGGCCGTGGCTGGAGGGCCTCAGGGACCACCTCCAGTTCGGCGAGCCAGCCGTGGTGCCACACCCAACCCCATACAGGCAAGTGTCCAGGGTGCAAGCCAAGTCCAACCCAGAGAGGCTCAGGAGGAGGCTCATGAGGAGGCACGACCTCTCCGAGGAAGAGGCCAGGAAGCGCATCCCAGACACCGTGGCCAGGGCCCTCGACCTCCCATTCGTGACCCTCAGGTCCCAGTCCACCGGCCAGCACTTCCGCCTCTTCATCAGGCACGGCCCACTCCAGGTGACCGCCGAGGAGGGCGGCTTTACCTGCTACGGCCTCTCCAAGGGCGGCTTCGTGCCGTGGTTCGGCTCCGGCGCCACCAACTTCTCCCTCCTCAAGCAAGCCGGCGACGTGGAGGAGAACCCAGGCCCAATGCCAGCCGCCAAGCGCGTCAAGCTGGACGGCGGAAAGCGCACCGCCGACGGATCCGAGTTCGAGAGCCCTAAGAAAAAGAGAAAAGTGGACAAGAAGTACTCGATCGGCCTCGATATTGGGACTAACTCTGTTGGCTGGGCCGTGATCACCGACGAGTACAAGGTGCCCTCAAAGAAGTTCAAGGTCCTGGGCAACACCGATCGGCATTCCATCAAGAAGAATCTCATTGGCGCTCTCCTGTTCGACAGCGGCGAGACGGCTGAGGCTACGCGGCTCAAGCGCACCGCCCGCAGGCGGTACACGCGCAGGAAGAATCGCATCTGCTACCTGCAGGAGATTTTCTCCAACGAGATGGCGAAGGTTGACGATTCTTTCTTCCACAGGCTGGAGGAGTCATTCCTCGTGGAGGAGGATAAGAAGCACGAGCGGCATCCAATCTTCGGCAACATTGTCGACGAGGTTGCCTACCACGAGAAGTACCCTACGATCTACCATCTGCGGAAGAAGCTCGTGGACTCCACAGATAAGGCGGACCTCCGCCTGATCTACCTCGCTCTGGCCCACATGATTAAGTTCAGGGGCCATTTCCTGATCGAGGGGGATCTCAACCCGGACAATAGCGATGTTGACAAGCTGTTCATCCAGCTCGTGCAGACGTACAACCAGCTCTTCGAGGAGAACCCCATTAATGCGTCAGGCGTCGACGCGAAGGCTATCCTGTCCGCTAGGCTCTCGAAGTCTCGGaagCTCGAGAACCTGATCGCCCAGCTGCCGGGCGAGAAGAAGAACGGCCTGTTCGGGAATCTCATTGCGCTCAGCCTGGGGCTCACGCCCAACTTCAAGTCGAATTTCGATCTCGCTGAGGACGCCAAGCTGCAGCTCTCCAAGGACACATACGACGATGACCTGGATAACCTCCTGGCCCAGATCGGCGATCAGTACGCGGACCTGTTCCTCGCTGCCAAGAATCTGTCGGACGCCATCCTCCTGTCTGATATTCTCAGGGTGAACACCGAGATTACGAAGGCTCCGCTCTCAGCCTCCATGATCAAGCGCTACGACGAGCACCATCAGGATCTGACCCTCCTGAAGGCGCTGGTCAGGCAGCAGCTCCCCGAGAAGTACAAGGAGATCTTCTTCGATCAGTCGAAGAACGGCTACGCTGGGTACATTGACGGCGGGGCCTCTCAGGAGGAGTTCTACAAGTTCATCAAGCCGATTCTGGAGAAGATGGACGGCACGGAGGAGCTGCTGGTGAAGCTCaagCGCGAGGACCTCCTGAGGAAGCAGCGGACATTCGATAACGGCAGCATCCCACACCAGATTCATCTCGGGGAGCTGCACGCTATCCTGAGGAGGCAGGAGGACTTCTACCCTTTCCTCAAGGATAACCGCGAGAAGATCGAGAAGATTCTGACTTTCAGGATCCCGTACTACGTCGGCCCACTCGCTAGGGGCAACTCCCGCTTCGCTTGGATGACCCGCAAGTCAGAGGAGACGATCACGCCGTGGAACTTCGAGGAGGTGGTCGACAAGGGCGCTAGCGCTCAGTCGTTCATCGAGAGGATGACGAATTTCGACAAGAACCTGCCAAATGAGAAGGTGCTCCCTAAGCACTCGCTCCTGTACGAGTACTTCACAGTCTACAACGAGCTGACTAAGGTGAAGTATGTGACCGAGGGCATGAGGAAGCCGGCTTTCCTGTCTGGGGAGCAGAAGAAGGCCATCGTGGACCTCCTGTTCAAGACCAACCGGAAGGTCACGGTTAAGCAGCTCAAGGAGGACTACTTCAAGAAGATTGAGTGCTTCGATTCGGTCGAGATCTCTGGCGTTGAGGACCGCTTCAACGCCTCCCTGGGGACCTACCACGATCTCCTGAAGATCATTAAGGATAAGGACTTCCTGGACAACGAGGAGAATGAGGATATCCTCGAGGACATTGTGCTGACACTCACTCTGTTCGAGGACCGGGAGATGATCGAGGAGCGCCTGAAGACTTACGCCCATCTCTTCGATGACAAGGTCATGAAGCAGCTCAAGAGGAGGAGGTACACCGGCTGGGGGAGGCTGAGCAGGAAGCTCATCAACGGCATTCGGGACAAGCAGTCCGGGAAGACGATCCTCGACTTCCTGAAGAGCGATGGCTTCGCGAACCGCAATTTCATGCAGCTGATTCACGATGACAGCCTCACATTCAAGGAGGATATCCAGAAGGCTCAGGTGAGCGGCCAGGGGGACTCGCTGCACGAGCATATCGCGAACCTCGCTGGCTCGCCAGCTATCAAGAAGGGGATTCTGCAGACCGTGAAGGTTGTGGACGAGCTGGTGAAGGTCATGGGCAGGCACAAGCCTGAGAACATCGTCATTGAGATGGCCCGGGAGAATCAGACCACGCAGAAGGGCCAGAAGAACTCACGCGAGAGGATGAAGAGGATCGAGGAGGGCATTAAGGAGCTGGGGTCCCAGATCCTCAAGGAGCACCCGGTGGAGAACACGCAGCTGCAGAATGAGAAGCTCTACCTGTACTACCTCCAGAATGGCCGCGATATGTATGTGGACCAGGAGCTGGATATTAACAGGCTCAGCGATTACGACGTCGATGCCATCGTTCCACAGTCATTCCTGAAGGATGACTCCATTGACAACAAGGTCCTCACCAGGTCGGACAAGAACCGGGGCAAGTCTGATAATGTTCCTTCAGAGGAGGTCGTTAAGAAGATGAAGAACTACTGGCGCCAGCTCCTGAATGCCAAGCTGATCACGCAGCGGAAGTTCGATAACCTCACAAAGGCTGAGAGGGGCGGGCTCTCTGAGCTGGACAAGGCGGGCTTCATCAAGAGGCAGCTGGTCGAGACACGGCAGATCACTAAGCACGTTGCGCAGATTCTCGACTCACGGATGAACACTAAGTACGATGAGAATGACAAGCTGATCCGCGAGGTGAAGGTCATCACCCTGAAGTCAAAGCTCGTCTCCGACTTCAGGAAGGATTTCCAGTTCTACAAGGTTCGGGAGATCAACAATTACCACCATGCCCATGACGCGTACCTGAACGCGGTGGTCGGCACAGCTCTGATCAAGAAGTACCCAAAGCTCGAGAGCGAGTTCGTGTACGGGGACTACAAGGTTTACGATGTGAGGAAGATGATCGCCAAGTCGGAGCAGGAGATTGGCAAGGCTACCGCCAAGTACTTCTTCTACTCTAACATTATGAATTTCTTCAAGACAGAGATCACTCTGGCCAATGGCGAGATCCGGAAGCGCCCCCTCATCGAGACGAACGGCGAGACGGGGGAGATCGTGTGGGACAAGGGCAGGGATTTCGCGACCGTCAGGAAGGTTCTCTCCATGCCACAAGTGAATATCGTCAAGAAGACAGAGGTCCAGACTGGCGGGTTCTCTAAGGAGTCAATTCTGCCTAAGCGGAACAGCGACAAGCTCATCGCCCGCAAGAAGGACTGGGATCCGAAGAAGTACGGCGGGTTCGACAGCCCCACTGTGGCCTACTCGGTCCTGGTTGTGGCGAAGGTTGAGAAGGGCAAGTCCAAGAAGCTCAAGAGCGTGAAGGAGCTGCTGGGGATCACGATTATGGAGCGCTCCAGCTTCGAGAAGAACCCGATCGATTTCCTGGAGGCGAAGGGCTACAAGGAGGTGAAGAAGGACCTGATCATTAAGCTCCCCAAGTACTCACTCTTCGAGCTGGAGAACGGCAGGAAGCGGATGCTGGCTTCCGCTGGCGAGCTGCAGAAGGGGAACGAGCTGGCTCTGCCGTCCAAGTATGTGAACTTCCTCTACCTGGCCTCCCACTACGAGAAGCTCAAGGGCAGCCCCGAGGACAACGAGCAGAAGCAGCTGTTCGTCGAGCAGCACAAGCATTACCTCGACGAGATCATTGAGCAGATTTCCGAGTTCTCCAAGCGCGTGATCCTGGCCGACGCGAATCTGGATAAGGTCCTCTCCGCGTACAACAAGCACCGCGACAAGCCAATCAGGGAGCAGGCTGAGAATATCATTCATCTCTTCACCCTGACGAACCTCGGCGCCCCTGCTGCTTTCAAGTACTTCGACACAACTATCGATCGCAAGAGGTACACAAGCACTAAGGAGGTCCTGGACGCGACCCTCATCCACCAGTCGATTACCGGCCTCTACGAGACGCGCATCGACCTGTCTCAGCTCGGGGGCGACGAATTCTCCGGGAGCGAGACGCCAGGCACCTCCGAGTCGGCCACCCCAGAATCTGCCACAGTGGTGTCCGGCCAAAAGCAGGACCGCCAGGGCGGAGAACGCAGAAGGTCCCAGCTCGATAGGGATCAGTGTGCCTACTGCAAGGAGAAGGGCCACTGGGCCAAAGACTGCCCGAAAAAGCCGCGCGGCCCACGCGGCCCAAGGCCACAAACATCCCTCCTTCCAAAGAAGAAGCGGAAGGTGGAGCTCAGCGGAGGATCTTCCGGAGGATCTAGCGGCTCCGAGACACCAGGAACATCCGAAAGCGCTACACCAGAATCTAGCGGAGGCTCTTCCGGAGGATCTAGGCCTACCCTCAACATCGAGGATGAGTATCGCCTCCACGAAACCTCCAAAGAACCGGACGTGTCCCTCGGCAGCACATGGCTCAGCGACTTCCCACAAGCGTGGGCCGAAACCGGCGGCATGGGCCTCGCCGTCCGCCAAGCCCCACTCATTATCCCGCTGAAGGCGACCTCCACACCGGTGTCCATCAAGCAGTACCCGATGAGCCAAGAGGCGAGGCTCGGGATTAAGCCGCACATTCAGCGCCTCCTCGATCAAGGCATTCTCGTGCCGTGCCAATCCCCGTGGAATACACCACTCCTCCCGGTCAAAAAGCCGGGCACCAACGACTATCGCCCGGTCCAAGATCTCCGCGAGGTCAACAAGCGCGTGGAAGACATCCACCCGACCGTCCCGAACCCGTATAATCTGCTCTCCGGGCTCCCACCATCCCACCAGTGGTATACAGTGCTGGACCTCAAAGACGCCTTCTTCTGTCTCCGCCTCCACCCAACAAGCCAGCCGCTCTTCGCCTTCGAGTGGCGCGACCCGGAGATGGGCATCTCCGGCCAACTGACATGGACACGCCTCCCGCAAGGCTTCAAGAACAGCCCGACACTCTTCAACGAGGCGCTCCATAGGGACCTCGCGGATTTTCGCATCCAGCATCCGGACCTCATCCTCCTCCAGTAT**gcc**GATGATCTCCTCCTCGCCGCGACCTCCGAGCTGGATTGTCAACAAGGCACACGCGCGCTCCTCCAAACACTCGGGAACCTCGGCTATCGCGCGTCCGCGAAAAAGGCCCAAATCTGCCAGAAGCAAGTGAAGTACCTCGGGTATCTGCTCAAGGAAGGCCAACGCTGGCTCACCGAAGCGCGCAAAGAAACAGTGATGGGGCAACCGACACCGAAAACACCACGCCAGCTGCGCGAGTTTCTCGGCAAAGCCGGCTTCTGTCGCCTCTTCATCCCGGGCTTTGCCGAGATGGCCGCGCCACTCTACCCACTCACCAAGCCGGGCACACTGTTTAACTGGGGGCCGGATCAGCAGAAAGCCTACCAAGAGATCAAACAAGCGCTCCTCACCGCCCCAGCGCTCGGGCTCCCAGATCTCACAAAGCCGTTCGAGCTGTTCGTCGATGAGAAGCAAGGCTACGCGAAGGGCGTGCTCACACAGAAGCTCGGCCCGTGGAGGAGGCCAGTGGCCTATCTCTCCAAAAAACTCGATCCAGTGGCCGCCGGCTGGCCACCGTGTCTGCGCATGGTCGCCGCGATTGCCGTGCTCACAAAGGATGCCGGCAAACTCACAATGGGCCAGCCGCTGGTGATCCTCGCGCCACATGCCGTGGAAGCCCTCGTCAAACAGCCGCCGGATAGGTGGCTCTCCAATGCGCGCATGACCCATTACCAAGCGCTCCTCCTCGACACCGATCGCGTCCAGTTCGGCCCAGTGGTCGCCCTCAATCCGGCGACACTGCTGCCACTCCCAGAGGAGGGCCTCCAACACAACTGTCTGGATATTCTCGCGGAAGCGCATGGCACAAGGCCAGACCTCACAGATCAACCGCTCAGCGGCGGCAGCAAAAGGACCGCCGATTCCCAACATTCCACCCCACCAAAAACCAAGAGGAAGGTGGGCTCTGGCCCAAAGAAGAAGAGGAAGGTCTGAAGGCCTAGCTCAGAGCTTTCGTTCGTATCATCGGTTTCGACAACGTTCGTCAAGTTCAATGCATCAGTTTCATTGCGCACACACCAGAATCCTACTGAGTTTGAGTATTATGGCATTGGGAAAACTGTTTTTCTTGTACCATTTGTTGTGCTTGTAATTTACTGTGTTTTTTATTCGGTTTTCGCTATCGAACTGTGAAATGGAAATGGATGGAGAAGAGTTAATGAATGATATGGTCCTTTTGTTCATTCTCAAATTAATATTATTTGTTTTTTCTCTTATTTGTTGTGTGTTGAATTTGAAATTATAAGAGATATGCAAACATTTTGTTTTGAGTAAAAATGTGTCAAATCGTGGCCTCTAATGACCGAAGTTAATATGAGGAGTAAAACACTTGTAGTTGTACCATTATGCTTATTCACTAGGCAACAAATATATTTTCAGACCTAGAAAAGCTGCAAATGTTACTGAATACAAGTATGTCCTCTTGTGTTTTAGACATTTATGAACTTTCCTTTATGTAATTTTCCAGAATCCTTGTCAGATTCTAATCATTGCTTTATAATTATAGTTATACTCATGGATTTGTAGTTGAGTATGAAAATATTTTTTAATGCATTTTATGACTTGCCAATTGATTGACAACGAATTCGTAATCATGGTCATAGCTGTTTCCTGTGTGAAATTGTTATCCGCTCACAATTCCACACAACATACGAGCCGGAAGCATAAAGTGTAAAGCCTGGGGTGCCTAATGAGTGAGCTAACTCACATTAATTGCGTTGCGCTCACTGCCCGCTTTCCAGTCGGGAAACCTGTCGTGCCAGCTGCATTAATGAATCGGCCAACGCGCGGGGAGAGGCGGTTTGCGTATTGGCTAGAGCAGCTTGCCAACATGGTGGAGCACGACACTCTCGTCTACTCCAAGAATATCAAAGATACAGTCTCAGAAGACCAAAGGGCTATTGAGACTTTTCAACAAAGGGTAATATCGGGAAACCTCCTCGGATTCCATTGCCCAGCTATCTGTCACTTCATCAAAAGGACAGTAGAAAAGGAAGGTGGCACCTACAAATGCCATCATTGCGATAAAGGAAAGGCTATCGTTCAAGATGCCTCTGCCGACAGTGGTCCCAAAGATGGACCCCCACCCACGAGGAGCATCGTGGAAAAAGAAGACGTTCCAACCACGTCTTCAAAGCAAGTGGATTGATGTGATAACATGGTGGAGCACGACACTCTCGTCTACTCCAAGAATATCAAAGATACAGTCTCAGAAGACCAAAGGGCTATTGAGACTTTTCAACAAAGGGTAATATCGGGAAACCTCCTCGGATTCCATTGCCCAGCTATCTGTCACTTCATCAAAAGGACAGTAGAAAAGGAAGGTGGCACCTACAAATGCCATCATTGCGATAAAGGAAAGGCTATCGTTCAAGATGCCTCTGCCGACAGTGGTCCCAAAGATGGACCCCCACCCACGAGGAGCATCGTGGAAAAAGAAGACGTTCCAACCACGTCTTCAAAGCAAGTGGATTGATGTGATATCTCCACTGACGTAAGGGATGACGCACAATCCCACTATCCTTCGCAAGACCTTCCTCTATATAAGGAAGTTCATTTCATTTGGAGAGGACACGCTGAAATCACCAGTCTCTCTCTACAAATCTATCTCTCTCGAGTCTACCATGAGCCCAGAACGACGCCCGGCCGACATCCGCCGTGCCACCGAGGCGGACATGCCGGCGGTCTGCACCATCGTCAACCACTACATCGAGACAAGCACGGTCAACTTCCGTACCGAGCCGCAGGAACCGCAGGAGTGGACGGACGACCTCGTCCGTCTGCGGGAGCGCTATCCCTGGCTCGTCGCCGAGGTGGACGGCGAGGTCGCCGGCATCGCCTACGCGGGCCCCTGGAAGGCACGCAACGCCTACGACTGGACGGCCGAGTCGACCGTGTACGTCTCCCCCCGCCACCAGCGGACGGGACTGGGCTCCACGCTCTACACCCACCTGCTGAAGTCCCTGGAGGCACAGGGCTTCAAGAGCGTGGTCGCTGTCATCGGGCTGCCCAACGACCCGAGCGTGCGCATGCACGAGGCGCTCGGATATGCCCCCCGCGGCATGCTGCGGGCGGCCGGCTTCAAGCACGGGAACTGGCATGACGTGGGTTTCTGGCAGCTGGACTTCAGCCTGCCGGTACCGCCCCGTCCGGTCCTGCCCGTCACCGAGATTTGACTCGAGTTTCTCCATAATAATGTGTGAGTAGTTCCCAGATAAGGGAATTAGGGTTCCTATAGGGTTTCGCTCATGTGTTGAGCATATAAGAAACCCTTAGTATGTATTTGTATTTGTAAAATACTTCTATCAATAAAATTTCTAATTCCTAAAACCAAAATCCAGTACTAAAATCCAGATCCCCCGAATTAATTCGGCGTTAATTCAGTACATTAAAAACGTCCGCAATGTGTTATTAAGTTGTCTAAGCGTCAATTTGTTTACACCACAATATATCCTGCCACCAGCCAGCCAACAGCTCCCCGACCGGCAGCTCGGCACAAAATCACCACTCGATACAGGCAGCCCATCAGTCCGGGACGGCGTCAGCGGGAGAGCCGTTGTAAGGCGGCAGACTTTGCTCATGTTACCGATGCTATTCGGAAGAACGGCAACTAAGCTGCCGGGTTTGAAACACGGATGATCTCGCGGAGGGTAGCATGTTGATTGTAACGATGACAGAGCGTTGCTGCCTGTGATCACCGCGGTTTCAAAATCGGCTCCGTCGATACTATGTTATACGCCAACTTTGAAAACAACTTTGAAAAAGCTGTTTTCTGGTATTTAAGGTTTTAGAATGCAAGGAACAGTGAATTGGAGTTCGTCTTGTTATAATTAGCTTCTTGGGGTATCTTTAAATACTGTAGAAAAGAGGAAGGAAATAATAAATGGCTAAAATGAGAATATCACCGGAATTGAAAAAACTGATCGAAAAATACCGCTGCGTAAAAGATACGGAAGGAATGTCTCCTGCTAAGGTATATAAGCTGGTGGGAGAAAATGAAAACCTATATTTAAAAATGACGGACAGCCGGTATAAAGGGACCACCTATGATGTGGAACGGGAAAAGGACATGATGCTATGGCTGGAAGGAAAGCTGCCTGTTCCAAAGGTCCTGCACTTTGAACGGCATGATGGCTGGAGCAATCTGCTCATGAGTGAGGCCGATGGCGTCCTTTGCTCGGAAGAGTATGAAGATGAACAAAGCCCTGAAAAGATTATCGAGCTGTATGCGGAGTGCATCAGGCTCTTTCACTCCATCGACATATCGGATTGTCCCTATACGAATAGCTTAGACAGCCGCTTAGCCGAATTGGATTACTTACTGAATAACGATCTGGCCGATGTGGATTGCGAAAACTGGGAAGAAGACACTCCATTTAAAGATCCGCGCGAGCTGTATGATTTTTTAAAGACGGAAAAGCCCGAAGAGGAACTTGTCTTTTCCCACGGCGACCTGGGAGACAGCAACATCTTTGTGAAAGATGGCAAAGTAAGTGGCTTTATTGATCTTGGGAGAAGCGGCAGGGCGGACAAGTGGTATGACATTGCCTTCTGCGTCCGGTCGATCAGGGAGGATATCGGGGAAGAACAGTATGTCGAGCTATTTTTTGACTTACTGGGGATCAAGCCTGATTGGGAGAAAATAAAATATTATATTTTACTGGATGAATTGTTTTAGTACCTAGAATGCATGACCAAAATCCCTTAACGTGAGTTTTCGTTCCACTGAGCGTCAGACCCCGTAGAAAAGATCAAAGGATCTTCTTGAGATCCTTTTTTTCTGCGCGTAATCTGCTGCTTGCAAACAAAAAAACCACCGCTACCAGCGGTGGTTTGTTTGCCGGATCAAGAGCTACCAACTCTTTTTCCGAAGGTAACTGGCTTCAGCAGAGCGCAGATACCAAATACTGTCCTTCTAGTGTAGCCGTAGTTAGGCCACCACTTCAAGAACTCTGTAGCACCGCCTACATACCTCGCTCTGCTAATCCTGTTACCAGTGGCTGCTGCCAGTGGCGATAAGTCGTGTCTTACCGGGTTGGACTCAAGACGATAGTTACCGGATAAGGCGCAGCGGTCGGGCTGAACGGGGGGTTCGTGCACACAGCCCAGCTTGGAGCGAACGACCTACACCGAACTGAGATACCTACAGCGTGAGCTATGAGAAAGCGCCACGCTTCCCGAAGGGAGAAAGGCGGACAGGTATCCGGTAAGCGGCAGGGTCGGAACAGGAGAGCGCACGAGGGAGCTTCCAGGGGGAAACGCCTGGTATCTTTATAGTCCTGTCGGGTTTCGCCACCTCTGACTTGAGCGTCGATTTTTGTGATGCTCGTCAGGGGGGCGGAGCCTATGGAAAAACGCCAGCAACGCGGCCTTTTTACGGTTCCTGGCCTTTTGCTGGCCTTTTGCTCACATGTTCTTTCCTGCGTTATCCCCTGATTCTGTGGATAACCGTATTACCGCCTTTGAGTGAGCTGATACCGCTCGCCGCAGCCGAACGACCGAGCGCAGCGAGTCAGTGAGCGAGGAAGCGGAAGAGCGCCTGATGCGGTATTTTCTCCTTACGCATCTGTGCGGTATTTCACACCGCATATGGTGCACTCTCAGTACAATCTGCTCTGATGCCGCATAGTTAAGCCAGTATACACTCCGCTATCGCTACGTGACTGGGTCATGGCTGCGCCCCGACACCCGCCAACACCCGCTGACGCGCCCTGACGGGCTTGTCTGCTCCCGGCATCCGCTTACAGACAAGCTGTGACCGTCTCCGGGAGCTGCATGTGTCAGAGGTTTTCACCGTCATCACCGAAACGCGCGAGGCAGGGTGCCTTGATGTGGGCGCCGGCGGTCGAGTGGCGACGGCGCGGCTTGTCCGCGCCCTGGTAGATTGCCTGGCCGTAGGCCAGCCATTTTTGAGCGGCCAGCGGCCGCGATAGGCCGACGCGAAGCGGCGGGGCGTAGGGAGCGCAGCGACCGAAGGGTAGGCGCTTTTTGCAGCTCTTCGGCTGTGCGCTGGCCAGACAGTTATGCACAGGCCAGGCGGGTTTTAAGAGTTTTAATAAGTTTTAAAGAGTTTTAGGCGGAAAAATCGCCTTTTTTCTCTTTTATATCAGTCACTTACATGTGTGACCGGTTCCCAATGTACGGCTTTGGGTTCCCAATGTACGGGTTCCGGTTCCCAATGTACGGCTTTGGGTTCCCAATGTACGTGCTATCCACAGGAAACAGACCTTTTCGACCTTTTTCCCCTGCTAGGGCAATTTGCCCTAGCATCTGCTCCGTACATTAGGAACCGGCGGATGCTTCGCCCTCGATCAGGTTGCGGTAGCGCATGACTAGGATCGGGCCAGCCTGCCCCGCCTCCTCCTTCAAATCGTACTCCGGCAGGTCATTTGACCCGATCAGCTTGCGCACGGTGAAACAGAACTTCTTGAACTCTCCGGCGCTGCCACTGCGTTCGTAGATCGTCTTGAACAACCATCTGGCTTCTGCCTTGCCTGCGGCGCGGCGTGCCAGGCGGTAGAGAAAACGGCCGATGCCGGGATCGATCAAAAAGTAATCGGGGTGAACCGTCAGCACGTCCGGGTTCTTGCCTTCTGTGATCTCGCGGTACATCCAATCAGCTAGCTCGATCTCGATGTACTCCGGCCGCCCGGTTTCGCTCTTTACGATCTTGTAGCGGCTAATCAAGGCTTCACCCTCGGATACCGTCACCAGGCGGCCGTTCTTGGCCTTCTTCGTACGCTGCATGGCAACGTGCGTGGTGTTTAACCGAATGCAGGTTTCTACCAGGTCGTCTTTCTGCTTTCCGCCATCGGCTCGCCGGCAGAACTTGAGTACGTCCGCAACGTGTGGACGGAACACGCGGCCGGGCTTGTCTCCCTTCCCTTCCCGGTATCGGTTCATGGATTCGGTTAGATGGGAAACCGCCATCAGTACCAGGTCGTAATCCCACACACTGGCCATGCCGGCCGGCCCTGCGGAAACCTCTACGTGCCCGTCTGGAAGCTCGTAGCGGATCACCTCGCCAGCTCGTCGGTCACGCTTCGACAGACGGAAAACGGCCACGTCCATGATGCTGCGACTATCGCGGGTGCCCACGTCATAGAGCATCGGAACGAAAAAATCTGGTTGCTCGTCGCCCTTGGGCGGCTTCCTAATCGACGGCGCACCGGCTGCCGGCGGTTGCCGGGATTCTTTGCGGATTCGATCAGCGGCCGCTTGCCACGATTCACCGGGGCGTGCTTCTGCCTCGATGCGTTGCCGCTGGGCGGCCTGCGCGGCCTTCAACTTCTCCACCAGGTCATCACCCAGCGCCGCGCCGATTTGTACCGGGCCGGATGGTTTGCGACCGCTCACGCCGATTCCTCGGGCTTGGGGGTTCCAGTGCCATTGCAGGGCCGGCAGACAACCCAGCCGCTTACGCCTGGCCAACCGCCCGTTCCTCCACACATGGGGCATTCCACGGCGTCGGTGCCTGGTTGTTCTTGATTTTCCATGCCGCCTCCTTTAGCCGCTAAAATTCATCTACTCATTTATTCATTTGCTCATTTACTCTGGTAGCTGCGCGATGTATTCAGATAGCAGCTCGGTAATGGTCTTGCCTTGGCGTACCGCGTACATCTTCAGCTTGGTGTGATCCTCCGCCGGCAACTGAAAGTTGACCCGCTTCATGGCTGGCGTGTCTGCCAGGCTGGCCAACGTTGCAGCCTTGCTGCTGCGTGCGCTCGGACGGCCGGCACTTAGCGTGTTTGTGCTTTTGCTCATTTTCTCTTTACCTCATTAACTCAAATGAGTTTTGATTTAATTTCAGCGGCCAGCGCCTGGACCTCGCGGGCAGCGTCGCCCTCGGGTTCTGATTCAAGAACGGTTGTGCCGGCGGCGGCAGTGCCTGGGTAGCTCACGCGCTGCGTGATACGGGACTCAAGAATGGGCAGCTCGTACCCGGCCAGCGCCTCGGCAACCTCACCGCCGATGCGCGTGCCTTTGATCGCCCGCGACACGACAAAGGCCGCTTGTAGCCTTCCATCCGTGACCTCAATGCGCTGCTTAACCAGCTCCACCAGGTCGGCGGTGGCCCATATGTCGTAAGGGCTTGGCTGCACCGGAATCAGCACGAAGTCGGCTGCCTTGATCGCGGACACAGCCAAGTCCGCCGCCTGGGGCGCTCCGTCGATCACTACGAAGTCGCGCCGGCCGATGGCCTTCACGTCGCGGTCAATCGTCGGGCGGTCGATGCCGACAACGGTTAGCGGTTGATCTTCCCGCACGGCCGCCCAATCGCGGGCACTGCCCTGGGGATCGGAATCGACTAACAGAACATCGGCCCCGGCGAGTTGCAGGGCGCGGGCTAGATGGGTTGCGATGGTCGTCTTGCCTGACCCGCCTTTCTGGTTAAGTACAGCGATAACCTTCATGCGTTCCCCTTGCGTATTTGTTTATTTACTCATCGCATCATATACGCAGCGACCGCATGACGCAAGCTGTTTTACTCAAATACACATCACCTTTTTAGACGGCGGCGCTCGGTTTCTTCAGCGGCCAAGCTGGCCGGCCAGGCCGCCAGCTTGGCATCAGACAAACCGGCCAGGATTTCATGCAGCCGCACGGTTGAGACGTGCGCGGGCGGCTCGAACACGTACCCGGCCGCGATCATCTCCGCCTCGATCTCTTCGGTAATGAAAAACGGTTCGTCCTGGCCGTCCTGGTGCGGTTTCATGCTTGTTCCTCTTGGCGTTCATTCTCGGCGGCCGCCAGGGCGTCGGCCTCGGTCAATGCGTCCTCACGGAAGGCACCGCGCCGCCTGGCCTCGGTGGGCGTCACTTCCTCGCTGCGCTCAAGTGCGCGGTACAGGGTCGAGCGATGCACGCCAAGCAGTGCAGCCGCCTCTTTCACGGTGCGGCCTTCCTGGTCGATCAGCTCGCGGGCGTGCGCGATCTGTGCCGGGGTGAGGGTAGGGCGGGGGCCAAACTTCACGCCTCGGGCCTTGGCGGCCTCGCGCCCGCTCCGGGTGCGGTCGATGATTAGGGAACGCTCGAACTCGGCAATGCCGGCGAACACGGTCAACACCATGCGGCCGGCCGGCGTGGTGGTGTCGGCCCACGGCTCTGCCAGGCTACGCAGGCCCGCGCCGGCCTCCTGGATGCGCTCGGCAATGTCCAGTAGGTCGCGGGTGCTGCGGGCCAGGCGGTCTAGCCTGGTCACTGTCACAACGTCGCCAGGGCGTAGGTGGTCAAGCATCCTGGCCAGCTCCGGGCGGTCGCGCCTGGTGCCGGTGATCTTCTCGGAAAACAGCTTGGTGCAGCCGGCCGCGTGCAGTTCGGCCCGTTGGTTGGTCAAGTCCTGGTCGTCGGTGCTGACGCGGGCATAGCCCAGCAGGCCAGCGGCGGCGCTCTTGTTCATGGCGTAATGTCTCCGGTTCTAGTCGCAAGTATTCTACTTTATGCGACTAAAACACGCGACAAGAAAACGCCAGGAAAAGGGCAGGGCGGCAGCCTGTCGCGTAACTTAGGACTTGTGCGACATGTCGTTTTCAGAAGACGGCTGCACTGAACGTCAGAAGCCGACTGCACTATAGCAGCGGAGGGGTTGGATCAAAGTACTTTGATCCCGAGGGGAACCCTGTGGTTGGCATGCACATACAAATGGACGAACGGATAAACCTTTTCACGCCCTTTTAAATATCCGTTATTCTAA

**Sequences S4.** Complete coding sequences of PPE, ePPE, ePPE-F156W, ePPE-V223I, ePPE-V223H, ePPE-F309N, ePPE-V223H-F309N, ePPE*, ePPEmax, CMPE-PPE and CMPE-ePPE in this study. The NLS^SV40^, NLS^c-myc^, bpNLS^SV40^, vbpNLS^SV40^, nCas9 (H840A), NC protein, Csy4 endoribonuclease, M-MLV RT and M-MLV RT^∆RNase H^ are highlighted in purple, pink, brown, dark blue, blue, green, dark green, yellow and orange, respectively. The introduced R221K/ N394K substitutions into nCas9 (H840A) and F156W/V223H/V223I/F309N/V223H-F309N substitutions into M-MLV RT^∆RNase H^ are shown in lowercase and bold. The P2A is underlined.

**PPE**

CCTAAGAAAAAGAGAAAAGTGGACAAGAAGTACTCGATCGGCCTCGATATTGGGACTAACTCTGTTGGCTGGGCCGTGATCACCGACGAGTACAAGGTGCCCTCAAAGAAGTTCAAGGTCCTGGGCAACACCGATCGGCATTCCATCAAGAAGAATCTCATTGGCGCTCTCCTGTTCGACAGCGGCGAGACGGCTGAGGCTACGCGGCTCAAGCGCACCGCCCGCAGGCGGTACACGCGCAGGAAGAATCGCATCTGCTACCTGCAGGAGATTTTCTCCAACGAGATGGCGAAGGTTGACGATTCTTTCTTCCACAGGCTGGAGGAGTCATTCCTCGTGGAGGAGGATAAGAAGCACGAGCGGCATCCAATCTTCGGCAACATTGTCGACGAGGTTGCCTACCACGAGAAGTACCCTACGATCTACCATCTGCGGAAGAAGCTCGTGGACTCCACAGATAAGGCGGACCTCCGCCTGATCTACCTCGCTCTGGCCCACATGATTAAGTTCAGGGGCCATTTCCTGATCGAGGGGGATCTCAACCCGGACAATAGCGATGTTGACAAGCTGTTCATCCAGCTCGTGCAGACGTACAACCAGCTCTTCGAGGAGAACCCCATTAATGCGTCAGGCGTCGACGCGAAGGCTATCCTGTCCGCTAGGCTCTCGAAGTCTCGGCGCCTCGAGAACCTGATCGCCCAGCTGCCGGGCGAGAAGAAGAACGGCCTGTTCGGGAATCTCATTGCGCTCAGCCTGGGGCTCACGCCCAACTTCAAGTCGAATTTCGATCTCGCTGAGGACGCCAAGCTGCAGCTCTCCAAGGACACATACGACGATGACCTGGATAACCTCCTGGCCCAGATCGGCGATCAGTACGCGGACCTGTTCCTCGCTGCCAAGAATCTGTCGGACGCCATCCTCCTGTCTGATATTCTCAGGGTGAACACCGAGATTACGAAGGCTCCGCTCTCAGCCTCCATGATCAAGCGCTACGACGAGCACCATCAGGATCTGACCCTCCTGAAGGCGCTGGTCAGGCAGCAGCTCCCCGAGAAGTACAAGGAGATCTTCTTCGATCAGTCGAAGAACGGCTACGCTGGGTACATTGACGGCGGGGCCTCTCAGGAGGAGTTCTACAAGTTCATCAAGCCGATTCTGGAGAAGATGGACGGCACGGAGGAGCTGCTGGTGAAGCTCAATCGCGAGGACCTCCTGAGGAAGCAGCGGACATTCGATAACGGCAGCATCCCACACCAGATTCATCTCGGGGAGCTGCACGCTATCCTGAGGAGGCAGGAGGACTTCTACCCTTTCCTCAAGGATAACCGCGAGAAGATCGAGAAGATTCTGACTTTCAGGATCCCGTACTACGTCGGCCCACTCGCTAGGGGCAACTCCCGCTTCGCTTGGATGACCCGCAAGTCAGAGGAGACGATCACGCCGTGGAACTTCGAGGAGGTGGTCGACAAGGGCGCTAGCGCTCAGTCGTTCATCGAGAGGATGACGAATTTCGACAAGAACCTGCCAAATGAGAAGGTGCTCCCTAAGCACTCGCTCCTGTACGAGTACTTCACAGTCTACAACGAGCTGACTAAGGTGAAGTATGTGACCGAGGGCATGAGGAAGCCGGCTTTCCTGTCTGGGGAGCAGAAGAAGGCCATCGTGGACCTCCTGTTCAAGACCAACCGGAAGGTCACGGTTAAGCAGCTCAAGGAGGACTACTTCAAGAAGATTGAGTGCTTCGATTCGGTCGAGATCTCTGGCGTTGAGGACCGCTTCAACGCCTCCCTGGGGACCTACCACGATCTCCTGAAGATCATTAAGGATAAGGACTTCCTGGACAACGAGGAGAATGAGGATATCCTCGAGGACATTGTGCTGACACTCACTCTGTTCGAGGACCGGGAGATGATCGAGGAGCGCCTGAAGACTTACGCCCATCTCTTCGATGACAAGGTCATGAAGCAGCTCAAGAGGAGGAGGTACACCGGCTGGGGGAGGCTGAGCAGGAAGCTCATCAACGGCATTCGGGACAAGCAGTCCGGGAAGACGATCCTCGACTTCCTGAAGAGCGATGGCTTCGCGAACCGCAATTTCATGCAGCTGATTCACGATGACAGCCTCACATTCAAGGAGGATATCCAGAAGGCTCAGGTGAGCGGCCAGGGGGACTCGCTGCACGAGCATATCGCGAACCTCGCTGGCTCGCCAGCTATCAAGAAGGGGATTCTGCAGACCGTGAAGGTTGTGGACGAGCTGGTGAAGGTCATGGGCAGGCACAAGCCTGAGAACATCGTCATTGAGATGGCCCGGGAGAATCAGACCACGCAGAAGGGCCAGAAGAACTCACGCGAGAGGATGAAGAGGATCGAGGAGGGCATTAAGGAGCTGGGGTCCCAGATCCTCAAGGAGCACCCGGTGGAGAACACGCAGCTGCAGAATGAGAAGCTCTACCTGTACTACCTCCAGAATGGCCGCGATATGTATGTGGACCAGGAGCTGGATATTAACAGGCTCAGCGATTACGACGTCGATGCCATCGTTCCACAGTCATTCCTGAAGGATGACTCCATTGACAACAAGGTCCTCACCAGGTCGGACAAGAACCGGGGCAAGTCTGATAATGTTCCTTCAGAGGAGGTCGTTAAGAAGATGAAGAACTACTGGCGCCAGCTCCTGAATGCCAAGCTGATCACGCAGCGGAAGTTCGATAACCTCACAAAGGCTGAGAGGGGCGGGCTCTCTGAGCTGGACAAGGCGGGCTTCATCAAGAGGCAGCTGGTCGAGACACGGCAGATCACTAAGCACGTTGCGCAGATTCTCGACTCACGGATGAACACTAAGTACGATGAGAATGACAAGCTGATCCGCGAGGTGAAGGTCATCACCCTGAAGTCAAAGCTCGTCTCCGACTTCAGGAAGGATTTCCAGTTCTACAAGGTTCGGGAGATCAACAATTACCACCATGCCCATGACGCGTACCTGAACGCGGTGGTCGGCACAGCTCTGATCAAGAAGTACCCAAAGCTCGAGAGCGAGTTCGTGTACGGGGACTACAAGGTTTACGATGTGAGGAAGATGATCGCCAAGTCGGAGCAGGAGATTGGCAAGGCTACCGCCAAGTACTTCTTCTACTCTAACATTATGAATTTCTTCAAGACAGAGATCACTCTGGCCAATGGCGAGATCCGGAAGCGCCCCCTCATCGAGACGAACGGCGAGACGGGGGAGATCGTGTGGGACAAGGGCAGGGATTTCGCGACCGTCAGGAAGGTTCTCTCCATGCCACAAGTGAATATCGTCAAGAAGACAGAGGTCCAGACTGGCGGGTTCTCTAAGGAGTCAATTCTGCCTAAGCGGAACAGCGACAAGCTCATCGCCCGCAAGAAGGACTGGGATCCGAAGAAGTACGGCGGGTTCGACAGCCCCACTGTGGCCTACTCGGTCCTGGTTGTGGCGAAGGTTGAGAAGGGCAAGTCCAAGAAGCTCAAGAGCGTGAAGGAGCTGCTGGGGATCACGATTATGGAGCGCTCCAGCTTCGAGAAGAACCCGATCGATTTCCTGGAGGCGAAGGGCTACAAGGAGGTGAAGAAGGACCTGATCATTAAGCTCCCCAAGTACTCACTCTTCGAGCTGGAGAACGGCAGGAAGCGGATGCTGGCTTCCGCTGGCGAGCTGCAGAAGGGGAACGAGCTGGCTCTGCCGTCCAAGTATGTGAACTTCCTCTACCTGGCCTCCCACTACGAGAAGCTCAAGGGCAGCCCCGAGGACAACGAGCAGAAGCAGCTGTTCGTCGAGCAGCACAAGCATTACCTCGACGAGATCATTGAGCAGATTTCCGAGTTCTCCAAGCGCGTGATCCTGGCCGACGCGAATCTGGATAAGGTCCTCTCCGCGTACAACAAGCACCGCGACAAGCCAATCAGGGAGCAGGCTGAGAATATCATTCATCTCTTCACCCTGACGAACCTCGGCGCCCCTGCTGCTTTCAAGTACTTCGACACAACTATCGATCGCAAGAGGTACACAAGCACTAAGGAGGTCCTGGACGCGACCCTCATCCACCAGTCGATTACCGGCCTCTACGAGACGCGCATCGACCTGTCTCAGCTCGGGGGCGACGAATTCCCAAAGAAGAAGCGGAAGGTGGAGCTCAGCGGAGGATCTTCCGGAGGATCTAGCGGCTCCGAGACACCAGGAACATCCGAAAGCGCTACACCAGAATCTAGCGGAGGCTCTTCCGGAGGATCTAGGCCTACCCTCAACATCGAGGATGAGTATCGCCTCCACGAAACCTCCAAAGAACCGGACGTGTCCCTCGGCAGCACATGGCTCAGCGACTTCCCACAAGCGTGGGCCGAAACCGGCGGCATGGGCCTCGCCGTCCGCCAAGCCCCACTCATTATCCCGCTGAAGGCGACCTCCACACCGGTGTCCATCAAGCAGTACCCGATGAGCCAAGAGGCGAGGCTCGGGATTAAGCCGCACATTCAGCGCCTCCTCGATCAAGGCATTCTCGTGCCGTGCCAATCCCCGTGGAATACACCACTCCTCCCGGTCAAAAAGCCGGGCACCAACGACTATCGCCCGGTCCAAGATCTCCGCGAGGTCAACAAGCGCGTGGAAGACATCCACCCGACCGTCCCGAACCCGTATAATCTGCTCTCCGGGCTCCCACCATCCCACCAGTGGTATACAGTGCTGGACCTCAAAGACGCCTTCTTCTGTCTCCGCCTCCACCCAACAAGCCAGCCGCTCTTCGCCTTCGAGTGGCGCGACCCGGAGATGGGCATCTCCGGCCAACTGACATGGACACGCCTCCCGCAAGGCTTCAAGAACAGCCCGACACTCTTCAACGAGGCGCTCCATAGGGACCTCGCGGATTTTCGCATCCAGCATCCGGACCTCATCCTCCTCCAGTATGTGGATGATCTCCTCCTCGCCGCGACCTCCGAGCTGGATTGTCAACAAGGCACACGCGCGCTCCTCCAAACACTCGGGAACCTCGGCTATCGCGCGTCCGCGAAAAAGGCCCAAATCTGCCAGAAGCAAGTGAAGTACCTCGGGTATCTGCTCAAGGAAGGCCAACGCTGGCTCACCGAAGCGCGCAAAGAAACAGTGATGGGGCAACCGACACCGAAAACACCACGCCAGCTGCGCGAGTTTCTCGGCAAAGCCGGCTTCTGTCGCCTCTTCATCCCGGGCTTTGCCGAGATGGCCGCGCCACTCTACCCACTCACCAAGCCGGGCACACTGTTTAACTGGGGGCCGGATCAGCAGAAAGCCTACCAAGAGATCAAACAAGCGCTCCTCACCGCCCCAGCGCTCGGGCTCCCAGATCTCACAAAGCCGTTCGAGCTGTTCGTCGATGAGAAGCAAGGCTACGCGAAGGGCGTGCTCACACAGAAGCTCGGCCCGTGGAGGAGGCCAGTGGCCTATCTCTCCAAAAAACTCGATCCAGTGGCCGCCGGCTGGCCACCGTGTCTGCGCATGGTCGCCGCGATTGCCGTGCTCACAAAGGATGCCGGCAAACTCACAATGGGCCAGCCGCTGGTGATCCTCGCGCCACATGCCGTGGAAGCCCTCGTCAAACAGCCGCCGGATAGGTGGCTCTCCAATGCGCGCATGACCCATTACCAAGCGCTCCTCCTCGACACCGATCGCGTCCAGTTCGGCCCAGTGGTCGCCCTCAATCCGGCGACACTGCTGCCACTCCCAGAGGAGGGCCTCCAACACAACTGTCTGGATATTCTCGCGGAAGCGCATGGCACAAGGCCAGACCTCACAGATCAACCGCTCCCGGATGCGGATCACACATGGTATACCGACGGCTCCTCTCTGCTCCAAGAGGGCCAAAGGAAAGCCGGCGCCGCGGTGACCACAGAAACAGAAGTGATCTGGGCCAAGGCCCTCCCAGCCGGCACATCCGCGCAAAGGGCGGAACTCATCGCGCTCACACAAGCCCTCAAGATGGCCGAGGGCAAGAAGCTCAACGTCTACACAGACTCCCGCTATGCCTTCGCCACCGCCCACATTCACGGCGAAATCTATAGGAGGCGCGGCTGGCTCACAAGCGAGGGGAAGGAGATCAAGAACAAGGATGAGATCCTCGCGCTGCTCAAGGCCCTCTTTCTCCCGAAGCGCCTCTCCATCATCCACTGTCCGGGCCACCAAAAGGGGCACTCCGCGGAAGCGAGGGGCAATAGGATGGCCGATCAAGCCGCGCGCAAAGCCGCGATTACCGAAACCCCAGACACATCCACCCTCCTCATCGAAAACTCCTCCCCAAAGCGGCGGCAGCCCGAAGAAGAAAAGGAAGGTGTGA

**ePPE**

CCTAAGAAAAAGAGAAAAGTGGACAAGAAGTACTCGATCGGCCTCGATATTGGGACTAACTCTGTTGGCTGGGCCGTGATCACCGACGAGTACAAGGTGCCCTCAAAGAAGTTCAAGGTCCTGGGCAACACCGATCGGCATTCCATCAAGAAGAATCTCATTGGCGCTCTCCTGTTCGACAGCGGCGAGACGGCTGAGGCTACGCGGCTCAAGCGCACCGCCCGCAGGCGGTACACGCGCAGGAAGAATCGCATCTGCTACCTGCAGGAGATTTTCTCCAACGAGATGGCGAAGGTTGACGATTCTTTCTTCCACAGGCTGGAGGAGTCATTCCTCGTGGAGGAGGATAAGAAGCACGAGCGGCATCCAATCTTCGGCAACATTGTCGACGAGGTTGCCTACCACGAGAAGTACCCTACGATCTACCATCTGCGGAAGAAGCTCGTGGACTCCACAGATAAGGCGGACCTCCGCCTGATCTACCTCGCTCTGGCCCACATGATTAAGTTCAGGGGCCATTTCCTGATCGAGGGGGATCTCAACCCGGACAATAGCGATGTTGACAAGCTGTTCATCCAGCTCGTGCAGACGTACAACCAGCTCTTCGAGGAGAACCCCATTAATGCGTCAGGCGTCGACGCGAAGGCTATCCTGTCCGCTAGGCTCTCGAAGTCTCGGCGCCTCGAGAACCTGATCGCCCAGCTGCCGGGCGAGAAGAAGAACGGCCTGTTCGGGAATCTCATTGCGCTCAGCCTGGGGCTCACGCCCAACTTCAAGTCGAATTTCGATCTCGCTGAGGACGCCAAGCTGCAGCTCTCCAAGGACACATACGACGATGACCTGGATAACCTCCTGGCCCAGATCGGCGATCAGTACGCGGACCTGTTCCTCGCTGCCAAGAATCTGTCGGACGCCATCCTCCTGTCTGATATTCTCAGGGTGAACACCGAGATTACGAAGGCTCCGCTCTCAGCCTCCATGATCAAGCGCTACGACGAGCACCATCAGGATCTGACCCTCCTGAAGGCGCTGGTCAGGCAGCAGCTCCCCGAGAAGTACAAGGAGATCTTCTTCGATCAGTCGAAGAACGGCTACGCTGGGTACATTGACGGCGGGGCCTCTCAGGAGGAGTTCTACAAGTTCATCAAGCCGATTCTGGAGAAGATGGACGGCACGGAGGAGCTGCTGGTGAAGCTCAATCGCGAGGACCTCCTGAGGAAGCAGCGGACATTCGATAACGGCAGCATCCCACACCAGATTCATCTCGGGGAGCTGCACGCTATCCTGAGGAGGCAGGAGGACTTCTACCCTTTCCTCAAGGATAACCGCGAGAAGATCGAGAAGATTCTGACTTTCAGGATCCCGTACTACGTCGGCCCACTCGCTAGGGGCAACTCCCGCTTCGCTTGGATGACCCGCAAGTCAGAGGAGACGATCACGCCGTGGAACTTCGAGGAGGTGGTCGACAAGGGCGCTAGCGCTCAGTCGTTCATCGAGAGGATGACGAATTTCGACAAGAACCTGCCAAATGAGAAGGTGCTCCCTAAGCACTCGCTCCTGTACGAGTACTTCACAGTCTACAACGAGCTGACTAAGGTGAAGTATGTGACCGAGGGCATGAGGAAGCCGGCTTTCCTGTCTGGGGAGCAGAAGAAGGCCATCGTGGACCTCCTGTTCAAGACCAACCGGAAGGTCACGGTTAAGCAGCTCAAGGAGGACTACTTCAAGAAGATTGAGTGCTTCGATTCGGTCGAGATCTCTGGCGTTGAGGACCGCTTCAACGCCTCCCTGGGGACCTACCACGATCTCCTGAAGATCATTAAGGATAAGGACTTCCTGGACAACGAGGAGAATGAGGATATCCTCGAGGACATTGTGCTGACACTCACTCTGTTCGAGGACCGGGAGATGATCGAGGAGCGCCTGAAGACTTACGCCCATCTCTTCGATGACAAGGTCATGAAGCAGCTCAAGAGGAGGAGGTACACCGGCTGGGGGAGGCTGAGCAGGAAGCTCATCAACGGCATTCGGGACAAGCAGTCCGGGAAGACGATCCTCGACTTCCTGAAGAGCGATGGCTTCGCGAACCGCAATTTCATGCAGCTGATTCACGATGACAGCCTCACATTCAAGGAGGATATCCAGAAGGCTCAGGTGAGCGGCCAGGGGGACTCGCTGCACGAGCATATCGCGAACCTCGCTGGCTCGCCAGCTATCAAGAAGGGGATTCTGCAGACCGTGAAGGTTGTGGACGAGCTGGTGAAGGTCATGGGCAGGCACAAGCCTGAGAACATCGTCATTGAGATGGCCCGGGAGAATCAGACCACGCAGAAGGGCCAGAAGAACTCACGCGAGAGGATGAAGAGGATCGAGGAGGGCATTAAGGAGCTGGGGTCCCAGATCCTCAAGGAGCACCCGGTGGAGAACACGCAGCTGCAGAATGAGAAGCTCTACCTGTACTACCTCCAGAATGGCCGCGATATGTATGTGGACCAGGAGCTGGATATTAACAGGCTCAGCGATTACGACGTCGATGCCATCGTTCCACAGTCATTCCTGAAGGATGACTCCATTGACAACAAGGTCCTCACCAGGTCGGACAAGAACCGGGGCAAGTCTGATAATGTTCCTTCAGAGGAGGTCGTTAAGAAGATGAAGAACTACTGGCGCCAGCTCCTGAATGCCAAGCTGATCACGCAGCGGAAGTTCGATAACCTCACAAAGGCTGAGAGGGGCGGGCTCTCTGAGCTGGACAAGGCGGGCTTCATCAAGAGGCAGCTGGTCGAGACACGGCAGATCACTAAGCACGTTGCGCAGATTCTCGACTCACGGATGAACACTAAGTACGATGAGAATGACAAGCTGATCCGCGAGGTGAAGGTCATCACCCTGAAGTCAAAGCTCGTCTCCGACTTCAGGAAGGATTTCCAGTTCTACAAGGTTCGGGAGATCAACAATTACCACCATGCCCATGACGCGTACCTGAACGCGGTGGTCGGCACAGCTCTGATCAAGAAGTACCCAAAGCTCGAGAGCGAGTTCGTGTACGGGGACTACAAGGTTTACGATGTGAGGAAGATGATCGCCAAGTCGGAGCAGGAGATTGGCAAGGCTACCGCCAAGTACTTCTTCTACTCTAACATTATGAATTTCTTCAAGACAGAGATCACTCTGGCCAATGGCGAGATCCGGAAGCGCCCCCTCATCGAGACGAACGGCGAGACGGGGGAGATCGTGTGGGACAAGGGCAGGGATTTCGCGACCGTCAGGAAGGTTCTCTCCATGCCACAAGTGAATATCGTCAAGAAGACAGAGGTCCAGACTGGCGGGTTCTCTAAGGAGTCAATTCTGCCTAAGCGGAACAGCGACAAGCTCATCGCCCGCAAGAAGGACTGGGATCCGAAGAAGTACGGCGGGTTCGACAGCCCCACTGTGGCCTACTCGGTCCTGGTTGTGGCGAAGGTTGAGAAGGGCAAGTCCAAGAAGCTCAAGAGCGTGAAGGAGCTGCTGGGGATCACGATTATGGAGCGCTCCAGCTTCGAGAAGAACCCGATCGATTTCCTGGAGGCGAAGGGCTACAAGGAGGTGAAGAAGGACCTGATCATTAAGCTCCCCAAGTACTCACTCTTCGAGCTGGAGAACGGCAGGAAGCGGATGCTGGCTTCCGCTGGCGAGCTGCAGAAGGGGAACGAGCTGGCTCTGCCGTCCAAGTATGTGAACTTCCTCTACCTGGCCTCCCACTACGAGAAGCTCAAGGGCAGCCCCGAGGACAACGAGCAGAAGCAGCTGTTCGTCGAGCAGCACAAGCATTACCTCGACGAGATCATTGAGCAGATTTCCGAGTTCTCCAAGCGCGTGATCCTGGCCGACGCGAATCTGGATAAGGTCCTCTCCGCGTACAACAAGCACCGCGACAAGCCAATCAGGGAGCAGGCTGAGAATATCATTCATCTCTTCACCCTGACGAACCTCGGCGCCCCTGCTGCTTTCAAGTACTTCGACACAACTATCGATCGCAAGAGGTACACAAGCACTAAGGAGGTCCTGGACGCGACCCTCATCCACCAGTCGATTACCGGCCTCTACGAGACGCGCATCGACCTGTCTCAGCTCGGGGGCGACGAATTCTCCGGGAGCGAGACGCCAGGCACCTCCGAGTCGGCCACCCCAGAATCTGCCACAGTGGTGTCCGGCCAAAAGCAGGACCGCCAGGGCGGAGAACGCAGAAGGTCCCAGCTCGATAGGGATCAGTGTGCCTACTGCAAGGAGAAGGGCCACTGGGCCAAAGACTGCCCGAAAAAGCCGCGCGGCCCACGCGGCCCAAGGCCACAAACATCCCTCCTTCCAAAGAAGAAGCGGAAGGTGGAGCTCAGCGGAGGATCTTCCGGAGGATCTAGCGGCTCCGAGACACCAGGAACATCCGAAAGCGCTACACCAGAATCTAGCGGAGGCTCTTCCGGAGGATCTAGGCCTACCCTCAACATCGAGGATGAGTATCGCCTCCACGAAACCTCCAAAGAACCGGACGTGTCCCTCGGCAGCACATGGCTCAGCGACTTCCCACAAGCGTGGGCCGAAACCGGCGGCATGGGCCTCGCCGTCCGCCAAGCCCCACTCATTATCCCGCTGAAGGCGACCTCCACACCGGTGTCCATCAAGCAGTACCCGATGAGCCAAGAGGCGAGGCTCGGGATTAAGCCGCACATTCAGCGCCTCCTCGATCAAGGCATTCTCGTGCCGTGCCAATCCCCGTGGAATACACCACTCCTCCCGGTCAAAAAGCCGGGCACCAACGACTATCGCCCGGTCCAAGATCTCCGCGAGGTCAACAAGCGCGTGGAAGACATCCACCCGACCGTCCCGAACCCGTATAATCTGCTCTCCGGGCTCCCACCATCCCACCAGTGGTATACAGTGCTGGACCTCAAAGACGCCTTCTTCTGTCTCCGCCTCCACCCAACAAGCCAGCCGCTCTTCGCCTTCGAGTGGCGCGACCCGGAGATGGGCATCTCCGGCCAACTGACATGGACACGCCTCCCGCAAGGCTTCAAGAACAGCCCGACACTCTTCAACGAGGCGCTCCATAGGGACCTCGCGGATTTTCGCATCCAGCATCCGGACCTCATCCTCCTCCAGTATGTGGATGATCTCCTCCTCGCCGCGACCTCCGAGCTGGATTGTCAACAAGGCACACGCGCGCTCCTCCAAACACTCGGGAACCTCGGCTATCGCGCGTCCGCGAAAAAGGCCCAAATCTGCCAGAAGCAAGTGAAGTACCTCGGGTATCTGCTCAAGGAAGGCCAACGCTGGCTCACCGAAGCGCGCAAAGAAACAGTGATGGGGCAACCGACACCGAAAACACCACGCCAGCTGCGCGAGTTTCTCGGCAAAGCCGGCTTCTGTCGCCTCTTCATCCCGGGCTTTGCCGAGATGGCCGCGCCACTCTACCCACTCACCAAGCCGGGCACACTGTTTAACTGGGGGCCGGATCAGCAGAAAGCCTACCAAGAGATCAAACAAGCGCTCCTCACCGCCCCAGCGCTCGGGCTCCCAGATCTCACAAAGCCGTTCGAGCTGTTCGTCGATGAGAAGCAAGGCTACGCGAAGGGCGTGCTCACACAGAAGCTCGGCCCGTGGAGGAGGCCAGTGGCCTATCTCTCCAAAAAACTCGATCCAGTGGCCGCCGGCTGGCCACCGTGTCTGCGCATGGTCGCCGCGATTGCCGTGCTCACAAAGGATGCCGGCAAACTCACAATGGGCCAGCCGCTGGTGATCCTCGCGCCACATGCCGTGGAAGCCCTCGTCAAACAGCCGCCGGATAGGTGGCTCTCCAATGCGCGCATGACCCATTACCAAGCGCTCCTCCTCGACACCGATCGCGTCCAGTTCGGCCCAGTGGTCGCCCTCAATCCGGCGACACTGCTGCCACTCCCAGAGGAGGGCCTCCAACACAACTGTCTGGATATTCTCGCGGAAGCGCATGGCACAAGGCCAGACCTCACAGATCAACCGCTCAGCGGCGGCAGCCCGAAGAAGAAAAGGAAGGTGTGA

**ePPE-F156W**

ACCCTCAACATCGAGGATGAGTATCGCCTCCACGAAACCTCCAAAGAACCGGACGTGTCCCTCGGCAGCACATGGCTCAGCGACTTCCCACAAGCGTGGGCCGAAACCGGCGGCATGGGCCTCGCCGTCCGCCAAGCCCCACTCATTATCCCGCTGAAGGCGACCTCCACACCGGTGTCCATCAAGCAGTACCCGATGAGCCAAGAGGCGAGGCTCGGGATTAAGCCGCACATTCAGCGCCTCCTCGATCAAGGCATTCTCGTGCCGTGCCAATCCCCGTGGAATACACCACTCCTCCCGGTCAAAAAGCCGGGCACCAACGACTATCGCCCGGTCCAAGATCTCCGCGAGGTCAACAAGCGCGTGGAAGACATCCACCCGACCGTCCCGAACCCGTATAATCTGCTCTCCGGGCTCCCACCATCCCACCAGTGGTATACAGTGCTGGACCTCAAAGACGCCTTC**tgg**TGTCTCCGCCTCCACCCAACAAGCCAGCCGCTCTTCGCCTTCGAGTGGCGCGACCCGGAGATGGGCATCTCCGGCCAACTGACATGGACACGCCTCCCGCAAGGCTTCAAGAACAGCCCGACACTCTTCAACGAGGCGCTCCATAGGGACCTCGCGGATTTTCGCATCCAGCATCCGGACCTCATCCTCCTCCAGTATGTGGATGATCTCCTCCTCGCCGCGACCTCCGAGCTGGATTGTCAACAAGGCACACGCGCGCTCCTCCAAACACTCGGGAACCTCGGCTATCGCGCGTCCGCGAAAAAGGCCCAAATCTGCCAGAAGCAAGTGAAGTACCTCGGGTATCTGCTCAAGGAAGGCCAACGCTGGCTCACCGAAGCGCGCAAAGAAACAGTGATGGGGCAACCGACACCGAAAACACCACGCCAGCTGCGCGAGTTTCTCGGCAAAGCCGGCTTCTGTCGCCTCTTCATCCCGGGCTTTGCCGAGATGGCCGCGCCACTCTACCCACTCACCAAGCCGGGCACACTGTTTAACTGGGGGCCGGATCAGCAGAAAGCCTACCAAGAGATCAAACAAGCGCTCCTCACCGCCCCAGCGCTCGGGCTCCCAGATCTCACAAAGCCGTTCGAGCTGTTCGTCGATGAGAAGCAAGGCTACGCGAAGGGCGTGCTCACACAGAAGCTCGGCCCGTGGAGGAGGCCAGTGGCCTATCTCTCCAAAAAACTCGATCCAGTGGCCGCCGGCTGGCCACCGTGTCTGCGCATGGTCGCCGCGATTGCCGTGCTCACAAAGGATGCCGGCAAACTCACAATGGGCCAGCCGCTGGTGATCCTCGCGCCACATGCCGTGGAAGCCCTCGTCAAACAGCCGCCGGATAGGTGGCTCTCCAATGCGCGCATGACCCATTACCAAGCGCTCCTCCTCGACACCGATCGCGTCCAGTTCGGCCCAGTGGTCGCCCTCAATCCGGCGACACTGCTGCCACTCCCAGAGGAGGGCCTCCAACACAACTGTCTGGATATTCTCGCGGAAGCGCATGGCACAAGGCCAGACCTCACAGATCAACCGCTC

**ePPE-V223H**

ACCCTCAACATCGAGGATGAGTATCGCCTCCACGAAACCTCCAAAGAACCGGACGTGTCCCTCGGCAGCACATGGCTCAGCGACTTCCCACAAGCGTGGGCCGAAACCGGCGGCATGGGCCTCGCCGTCCGCCAAGCCCCACTCATTATCCCGCTGAAGGCGACCTCCACACCGGTGTCCATCAAGCAGTACCCGATGAGCCAAGAGGCGAGGCTCGGGATTAAGCCGCACATTCAGCGCCTCCTCGATCAAGGCATTCTCGTGCCGTGCCAATCCCCGTGGAATACACCACTCCTCCCGGTCAAAAAGCCGGGCACCAACGACTATCGCCCGGTCCAAGATCTCCGCGAGGTCAACAAGCGCGTGGAAGACATCCACCCGACCGTCCCGAACCCGTATAATCTGCTCTCCGGGCTCCCACCATCCCACCAGTGGTATACAGTGCTGGACCTCAAAGACGCCTTCTTCTGTCTCCGCCTCCACCCAACAAGCCAGCCGCTCTTCGCCTTCGAGTGGCGCGACCCGGAGATGGGCATCTCCGGCCAACTGACATGGACACGCCTCCCGCAAGGCTTCAAGAACAGCCCGACACTCTTCAACGAGGCGCTCCATAGGGACCTCGCGGATTTTCGCATCCAGCATCCGGACCTCATCCTCCTCCAGTAT**cac**GATGATCTCCTCCTCGCCGCGACCTCCGAGCTGGATTGTCAACAAGGCACACGCGCGCTCCTCCAAACACTCGGGAACCTCGGCTATCGCGCGTCCGCGAAAAAGGCCCAAATCTGCCAGAAGCAAGTGAAGTACCTCGGGTATCTGCTCAAGGAAGGCCAACGCTGGCTCACCGAAGCGCGCAAAGAAACAGTGATGGGGCAACCGACACCGAAAACACCACGCCAGCTGCGCGAGTTTCTCGGCAAAGCCGGCTTCTGTCGCCTCTTCATCCCGGGCTTTGCCGAGATGGCCGCGCCACTCTACCCACTCACCAAGCCGGGCACACTGTTTAACTGGGGGCCGGATCAGCAGAAAGCCTACCAAGAGATCAAACAAGCGCTCCTCACCGCCCCAGCGCTCGGGCTCCCAGATCTCACAAAGCCGTTCGAGCTGTTCGTCGATGAGAAGCAAGGCTACGCGAAGGGCGTGCTCACACAGAAGCTCGGCCCGTGGAGGAGGCCAGTGGCCTATCTCTCCAAAAAACTCGATCCAGTGGCCGCCGGCTGGCCACCGTGTCTGCGCATGGTCGCCGCGATTGCCGTGCTCACAAAGGATGCCGGCAAACTCACAATGGGCCAGCCGCTGGTGATCCTCGCGCCACATGCCGTGGAAGCCCTCGTCAAACAGCCGCCGGATAGGTGGCTCTCCAATGCGCGCATGACCCATTACCAAGCGCTCCTCCTCGACACCGATCGCGTCCAGTTCGGCCCAGTGGTCGCCCTCAATCCGGCGACACTGCTGCCACTCCCAGAGGAGGGCCTCCAACACAACTGTCTGGATATTCTCGCGGAAGCGCATGGCACAAGGCCAGACCTCACAGATCAACCGCTC

**ePPE-V223I**

ACCCTCAACATCGAGGATGAGTATCGCCTCCACGAAACCTCCAAAGAACCGGACGTGTCCCTCGGCAGCACATGGCTCAGCGACTTCCCACAAGCGTGGGCCGAAACCGGCGGCATGGGCCTCGCCGTCCGCCAAGCCCCACTCATTATCCCGCTGAAGGCGACCTCCACACCGGTGTCCATCAAGCAGTACCCGATGAGCCAAGAGGCGAGGCTCGGGATTAAGCCGCACATTCAGCGCCTCCTCGATCAAGGCATTCTCGTGCCGTGCCAATCCCCGTGGAATACACCACTCCTCCCGGTCAAAAAGCCGGGCACCAACGACTATCGCCCGGTCCAAGATCTCCGCGAGGTCAACAAGCGCGTGGAAGACATCCACCCGACCGTCCCGAACCCGTATAATCTGCTCTCCGGGCTCCCACCATCCCACCAGTGGTATACAGTGCTGGACCTCAAAGACGCCTTCTTCTGTCTCCGCCTCCACCCAACAAGCCAGCCGCTCTTCGCCTTCGAGTGGCGCGACCCGGAGATGGGCATCTCCGGCCAACTGACATGGACACGCCTCCCGCAAGGCTTCAAGAACAGCCCGACACTCTTCAACGAGGCGCTCCATAGGGACCTCGCGGATTTTCGCATCCAGCATCCGGACCTCATCCTCCTCCAGTAT**atc**GATGATCTCCTCCTCGCCGCGACCTCCGAGCTGGATTGTCAACAAGGCACACGCGCGCTCCTCCAAACACTCGGGAACCTCGGCTATCGCGCGTCCGCGAAAAAGGCCCAAATCTGCCAGAAGCAAGTGAAGTACCTCGGGTATCTGCTCAAGGAAGGCCAACGCTGGCTCACCGAAGCGCGCAAAGAAACAGTGATGGGGCAACCGACACCGAAAACACCACGCCAGCTGCGCGAGTTTCTCGGCAAAGCCGGCTTCTGTCGCCTCTTCATCCCGGGCTTTGCCGAGATGGCCGCGCCACTCTACCCACTCACCAAGCCGGGCACACTGTTTAACTGGGGGCCGGATCAGCAGAAAGCCTACCAAGAGATCAAACAAGCGCTCCTCACCGCCCCAGCGCTCGGGCTCCCAGATCTCACAAAGCCGTTCGAGCTGTTCGTCGATGAGAAGCAAGGCTACGCGAAGGGCGTGCTCACACAGAAGCTCGGCCCGTGGAGGAGGCCAGTGGCCTATCTCTCCAAAAAACTCGATCCAGTGGCCGCCGGCTGGCCACCGTGTCTGCGCATGGTCGCCGCGATTGCCGTGCTCACAAAGGATGCCGGCAAACTCACAATGGGCCAGCCGCTGGTGATCCTCGCGCCACATGCCGTGGAAGCCCTCGTCAAACAGCCGCCGGATAGGTGGCTCTCCAATGCGCGCATGACCCATTACCAAGCGCTCCTCCTCGACACCGATCGCGTCCAGTTCGGCCCAGTGGTCGCCCTCAATCCGGCGACACTGCTGCCACTCCCAGAGGAGGGCCTCCAACACAACTGTCTGGATATTCTCGCGGAAGCGCATGGCACAAGGCCAGACCTCACAGATCAACCGCTC

**ePPE-F309N**

ACCCTCAACATCGAGGATGAGTATCGCCTCCACGAAACCTCCAAAGAACCGGACGTGTCCCTCGGCAGCACATGGCTCAGCGACTTCCCACAAGCGTGGGCCGAAACCGGCGGCATGGGCCTCGCCGTCCGCCAAGCCCCACTCATTATCCCGCTGAAGGCGACCTCCACACCGGTGTCCATCAAGCAGTACCCGATGAGCCAAGAGGCGAGGCTCGGGATTAAGCCGCACATTCAGCGCCTCCTCGATCAAGGCATTCTCGTGCCGTGCCAATCCCCGTGGAATACACCACTCCTCCCGGTCAAAAAGCCGGGCACCAACGACTATCGCCCGGTCCAAGATCTCCGCGAGGTCAACAAGCGCGTGGAAGACATCCACCCGACCGTCCCGAACCCGTATAATCTGCTCTCCGGGCTCCCACCATCCCACCAGTGGTATACAGTGCTGGACCTCAAAGACGCCTTCTTCTGTCTCCGCCTCCACCCAACAAGCCAGCCGCTCTTCGCCTTCGAGTGGCGCGACCCGGAGATGGGCATCTCCGGCCAACTGACATGGACACGCCTCCCGCAAGGCTTCAAGAACAGCCCGACACTCTTCAACGAGGCGCTCCATAGGGACCTCGCGGATTTTCGCATCCAGCATCCGGACCTCATCCTCCTCCAGTATGTGGATGATCTCCTCCTCGCCGCGACCTCCGAGCTGGATTGTCAACAAGGCACACGCGCGCTCCTCCAAACACTCGGGAACCTCGGCTATCGCGCGTCCGCGAAAAAGGCCCAAATCTGCCAGAAGCAAGTGAAGTACCTCGGGTATCTGCTCAAGGAAGGCCAACGCTGGCTCACCGAAGCGCGCAAAGAAACAGTGATGGGGCAACCGACACCGAAAACACCACGCCAGCTGCGCGAGTTTCTCGGCAAAGCCGGC**aac**TGTCGCCTCTTCATCCCGGGCTTTGCCGAGATGGCCGCGCCACTCTACCCACTCACCAAGCCGGGCACACTGTTTAACTGGGGGCCGGATCAGCAGAAAGCCTACCAAGAGATCAAACAAGCGCTCCTCACCGCCCCAGCGCTCGGGCTCCCAGATCTCACAAAGCCGTTCGAGCTGTTCGTCGATGAGAAGCAAGGCTACGCGAAGGGCGTGCTCACACAGAAGCTCGGCCCGTGGAGGAGGCCAGTGGCCTATCTCTCCAAAAAACTCGATCCAGTGGCCGCCGGCTGGCCACCGTGTCTGCGCATGGTCGCCGCGATTGCCGTGCTCACAAAGGATGCCGGCAAACTCACAATGGGCCAGCCGCTGGTGATCCTCGCGCCACATGCCGTGGAAGCCCTCGTCAAACAGCCGCCGGATAGGTGGCTCTCCAATGCGCGCATGACCCATTACCAAGCGCTCCTCCTCGACACCGATCGCGTCCAGTTCGGCCCAGTGGTCGCCCTCAATCCGGCGACACTGCTGCCACTCCCAGAGGAGGGCCTCCAACACAACTGTCTGGATATTCTCGCGGAAGCGCATGGCACAAGGCCAGACCTCACAGATCAACCGCTC

**ePPE-V223H-F309N**

ACCCTCAACATCGAGGATGAGTATCGCCTCCACGAAACCTCCAAAGAACCGGACGTGTCCCTCGGCAGCACATGGCTCAGCGACTTCCCACAAGCGTGGGCCGAAACCGGCGGCATGGGCCTCGCCGTCCGCCAAGCCCCACTCATTATCCCGCTGAAGGCGACCTCCACACCGGTGTCCATCAAGCAGTACCCGATGAGCCAAGAGGCGAGGCTCGGGATTAAGCCGCACATTCAGCGCCTCCTCGATCAAGGCATTCTCGTGCCGTGCCAATCCCCGTGGAATACACCACTCCTCCCGGTCAAAAAGCCGGGCACCAACGACTATCGCCCGGTCCAAGATCTCCGCGAGGTCAACAAGCGCGTGGAAGACATCCACCCGACCGTCCCGAACCCGTATAATCTGCTCTCCGGGCTCCCACCATCCCACCAGTGGTATACAGTGCTGGACCTCAAAGACGCCTTCTTCTGTCTCCGCCTCCACCCAACAAGCCAGCCGCTCTTCGCCTTCGAGTGGCGCGACCCGGAGATGGGCATCTCCGGCCAACTGACATGGACACGCCTCCCGCAAGGCTTCAAGAACAGCCCGACACTCTTCAACGAGGCGCTCCATAGGGACCTCGCGGATTTTCGCATCCAGCATCCGGACCTCATCCTCCTCCAGTAT**cac**GATGATCTCCTCCTCGCCGCGACCTCCGAGCTGGATTGTCAACAAGGCACACGCGCGCTCCTCCAAACACTCGGGAACCTCGGCTATCGCGCGTCCGCGAAAAAGGCCCAAATCTGCCAGAAGCAAGTGAAGTACCTCGGGTATCTGCTCAAGGAAGGCCAACGCTGGCTCACCGAAGCGCGCAAAGAAACAGTGATGGGGCAACCGACACCGAAAACACCACGCCAGCTGCGCGAGTTTCTCGGCAAAGCCGGC**aac**TGTCGCCTCTTCATCCCGGGCTTTGCCGAGATGGCCGCGCCACTCTACCCACTCACCAAGCCGGGCACACTGTTTAACTGGGGGCCGGATCAGCAGAAAGCCTACCAAGAGATCAAACAAGCGCTCCTCACCGCCCCAGCGCTCGGGCTCCCAGATCTCACAAAGCCGTTCGAGCTGTTCGTCGATGAGAAGCAAGGCTACGCGAAGGGCGTGCTCACACAGAAGCTCGGCCCGTGGAGGAGGCCAGTGGCCTATCTCTCCAAAAAACTCGATCCAGTGGCCGCCGGCTGGCCACCGTGTCTGCGCATGGTCGCCGCGATTGCCGTGCTCACAAAGGATGCCGGCAAACTCACAATGGGCCAGCCGCTGGTGATCCTCGCGCCACATGCCGTGGAAGCCCTCGTCAAACAGCCGCCGGATAGGTGGCTCTCCAATGCGCGCATGACCCATTACCAAGCGCTCCTCCTCGACACCGATCGCGTCCAGTTCGGCCCAGTGGTCGCCCTCAATCCGGCGACACTGCTGCCACTCCCAGAGGAGGGCCTCCAACACAACTGTCTGGATATTCTCGCGGAAGCGCATGGCACAAGGCCAGACCTCACAGATCAACCGCTC

**ePPE***

CCAGCCGCCAAGCGCGTCAAGCTGGACGGCGGAAAGCGCACCGCCGACGGATCCGAGTTCGAGAGCCCTAAGAAAAAGAGAAAAGTGGACAAGAAGTACTCGATCGGCCTCGATATTGGGACTAACTCTGTTGGCTGGGCCGTGATCACCGACGAGTACAAGGTGCCCTCAAAGAAGTTCAAGGTCCTGGGCAACACCGATCGGCATTCCATCAAGAAGAATCTCATTGGCGCTCTCCTGTTCGACAGCGGCGAGACGGCTGAGGCTACGCGGCTCAAGCGCACCGCCCGCAGGCGGTACACGCGCAGGAAGAATCGCATCTGCTACCTGCAGGAGATTTTCTCCAACGAGATGGCGAAGGTTGACGATTCTTTCTTCCACAGGCTGGAGGAGTCATTCCTCGTGGAGGAGGATAAGAAGCACGAGCGGCATCCAATCTTCGGCAACATTGTCGACGAGGTTGCCTACCACGAGAAGTACCCTACGATCTACCATCTGCGGAAGAAGCTCGTGGACTCCACAGATAAGGCGGACCTCCGCCTGATCTACCTCGCTCTGGCCCACATGATTAAGTTCAGGGGCCATTTCCTGATCGAGGGGGATCTCAACCCGGACAATAGCGATGTTGACAAGCTGTTCATCCAGCTCGTGCAGACGTACAACCAGCTCTTCGAGGAGAACCCCATTAATGCGTCAGGCGTCGACGCGAAGGCTATCCTGTCCGCTAGGCTCTCGAAGTCTCGGCGCCTCGAGAACCTGATCGCCCAGCTGCCGGGCGAGAAGAAGAACGGCCTGTTCGGGAATCTCATTGCGCTCAGCCTGGGGCTCACGCCCAACTTCAAGTCGAATTTCGATCTCGCTGAGGACGCCAAGCTGCAGCTCTCCAAGGACACATACGACGATGACCTGGATAACCTCCTGGCCCAGATCGGCGATCAGTACGCGGACCTGTTCCTCGCTGCCAAGAATCTGTCGGACGCCATCCTCCTGTCTGATATTCTCAGGGTGAACACCGAGATTACGAAGGCTCCGCTCTCAGCCTCCATGATCAAGCGCTACGACGAGCACCATCAGGATCTGACCCTCCTGAAGGCGCTGGTCAGGCAGCAGCTCCCCGAGAAGTACAAGGAGATCTTCTTCGATCAGTCGAAGAACGGCTACGCTGGGTACATTGACGGCGGGGCCTCTCAGGAGGAGTTCTACAAGTTCATCAAGCCGATTCTGGAGAAGATGGACGGCACGGAGGAGCTGCTGGTGAAGCTCAATCGCGAGGACCTCCTGAGGAAGCAGCGGACATTCGATAACGGCAGCATCCCACACCAGATTCATCTCGGGGAGCTGCACGCTATCCTGAGGAGGCAGGAGGACTTCTACCCTTTCCTCAAGGATAACCGCGAGAAGATCGAGAAGATTCTGACTTTCAGGATCCCGTACTACGTCGGCCCACTCGCTAGGGGCAACTCCCGCTTCGCTTGGATGACCCGCAAGTCAGAGGAGACGATCACGCCGTGGAACTTCGAGGAGGTGGTCGACAAGGGCGCTAGCGCTCAGTCGTTCATCGAGAGGATGACGAATTTCGACAAGAACCTGCCAAATGAGAAGGTGCTCCCTAAGCACTCGCTCCTGTACGAGTACTTCACAGTCTACAACGAGCTGACTAAGGTGAAGTATGTGACCGAGGGCATGAGGAAGCCGGCTTTCCTGTCTGGGGAGCAGAAGAAGGCCATCGTGGACCTCCTGTTCAAGACCAACCGGAAGGTCACGGTTAAGCAGCTCAAGGAGGACTACTTCAAGAAGATTGAGTGCTTCGATTCGGTCGAGATCTCTGGCGTTGAGGACCGCTTCAACGCCTCCCTGGGGACCTACCACGATCTCCTGAAGATCATTAAGGATAAGGACTTCCTGGACAACGAGGAGAATGAGGATATCCTCGAGGACATTGTGCTGACACTCACTCTGTTCGAGGACCGGGAGATGATCGAGGAGCGCCTGAAGACTTACGCCCATCTCTTCGATGACAAGGTCATGAAGCAGCTCAAGAGGAGGAGGTACACCGGCTGGGGGAGGCTGAGCAGGAAGCTCATCAACGGCATTCGGGACAAGCAGTCCGGGAAGACGATCCTCGACTTCCTGAAGAGCGATGGCTTCGCGAACCGCAATTTCATGCAGCTGATTCACGATGACAGCCTCACATTCAAGGAGGATATCCAGAAGGCTCAGGTGAGCGGCCAGGGGGACTCGCTGCACGAGCATATCGCGAACCTCGCTGGCTCGCCAGCTATCAAGAAGGGGATTCTGCAGACCGTGAAGGTTGTGGACGAGCTGGTGAAGGTCATGGGCAGGCACAAGCCTGAGAACATCGTCATTGAGATGGCCCGGGAGAATCAGACCACGCAGAAGGGCCAGAAGAACTCACGCGAGAGGATGAAGAGGATCGAGGAGGGCATTAAGGAGCTGGGGTCCCAGATCCTCAAGGAGCACCCGGTGGAGAACACGCAGCTGCAGAATGAGAAGCTCTACCTGTACTACCTCCAGAATGGCCGCGATATGTATGTGGACCAGGAGCTGGATATTAACAGGCTCAGCGATTACGACGTCGATGCCATCGTTCCACAGTCATTCCTGAAGGATGACTCCATTGACAACAAGGTCCTCACCAGGTCGGACAAGAACCGGGGCAAGTCTGATAATGTTCCTTCAGAGGAGGTCGTTAAGAAGATGAAGAACTACTGGCGCCAGCTCCTGAATGCCAAGCTGATCACGCAGCGGAAGTTCGATAACCTCACAAAGGCTGAGAGGGGCGGGCTCTCTGAGCTGGACAAGGCGGGCTTCATCAAGAGGCAGCTGGTCGAGACACGGCAGATCACTAAGCACGTTGCGCAGATTCTCGACTCACGGATGAACACTAAGTACGATGAGAATGACAAGCTGATCCGCGAGGTGAAGGTCATCACCCTGAAGTCAAAGCTCGTCTCCGACTTCAGGAAGGATTTCCAGTTCTACAAGGTTCGGGAGATCAACAATTACCACCATGCCCATGACGCGTACCTGAACGCGGTGGTCGGCACAGCTCTGATCAAGAAGTACCCAAAGCTCGAGAGCGAGTTCGTGTACGGGGACTACAAGGTTTACGATGTGAGGAAGATGATCGCCAAGTCGGAGCAGGAGATTGGCAAGGCTACCGCCAAGTACTTCTTCTACTCTAACATTATGAATTTCTTCAAGACAGAGATCACTCTGGCCAATGGCGAGATCCGGAAGCGCCCCCTCATCGAGACGAACGGCGAGACGGGGGAGATCGTGTGGGACAAGGGCAGGGATTTCGCGACCGTCAGGAAGGTTCTCTCCATGCCACAAGTGAATATCGTCAAGAAGACAGAGGTCCAGACTGGCGGGTTCTCTAAGGAGTCAATTCTGCCTAAGCGGAACAGCGACAAGCTCATCGCCCGCAAGAAGGACTGGGATCCGAAGAAGTACGGCGGGTTCGACAGCCCCACTGTGGCCTACTCGGTCCTGGTTGTGGCGAAGGTTGAGAAGGGCAAGTCCAAGAAGCTCAAGAGCGTGAAGGAGCTGCTGGGGATCACGATTATGGAGCGCTCCAGCTTCGAGAAGAACCCGATCGATTTCCTGGAGGCGAAGGGCTACAAGGAGGTGAAGAAGGACCTGATCATTAAGCTCCCCAAGTACTCACTCTTCGAGCTGGAGAACGGCAGGAAGCGGATGCTGGCTTCCGCTGGCGAGCTGCAGAAGGGGAACGAGCTGGCTCTGCCGTCCAAGTATGTGAACTTCCTCTACCTGGCCTCCCACTACGAGAAGCTCAAGGGCAGCCCCGAGGACAACGAGCAGAAGCAGCTGTTCGTCGAGCAGCACAAGCATTACCTCGACGAGATCATTGAGCAGATTTCCGAGTTCTCCAAGCGCGTGATCCTGGCCGACGCGAATCTGGATAAGGTCCTCTCCGCGTACAACAAGCACCGCGACAAGCCAATCAGGGAGCAGGCTGAGAATATCATTCATCTCTTCACCCTGACGAACCTCGGCGCCCCTGCTGCTTTCAAGTACTTCGACACAACTATCGATCGCAAGAGGTACACAAGCACTAAGGAGGTCCTGGACGCGACCCTCATCCACCAGTCGATTACCGGCCTCTACGAGACGCGCATCGACCTGTCTCAGCTCGGGGGCGACGAATTCTCCGGGAGCGAGACGCCAGGCACCTCCGAGTCGGCCACCCCAGAATCTGCCACAGTGGTGTCCGGCCAAAAGCAGGACCGCCAGGGCGGAGAACGCAGAAGGTCCCAGCTCGATAGGGATCAGTGTGCCTACTGCAAGGAGAAGGGCCACTGGGCCAAAGACTGCCCGAAAAAGCCGCGCGGCCCACGCGGCCCAAGGCCACAAACATCCCTCCTTCCAAAGAAGAAGCGGAAGGTGGAGCTCAGCGGAGGATCTTCCGGAGGATCTAGCGGCTCCGAGACACCAGGAACATCCGAAAGCGCTACACCAGAATCTAGCGGAGGCTCTTCCGGAGGATCTAGGCCTACCCTCAACATCGAGGATGAGTATCGCCTCCACGAAACCTCCAAAGAACCGGACGTGTCCCTCGGCAGCACATGGCTCAGCGACTTCCCACAAGCGTGGGCCGAAACCGGCGGCATGGGCCTCGCCGTCCGCCAAGCCCCACTCATTATCCCGCTGAAGGCGACCTCCACACCGGTGTCCATCAAGCAGTACCCGATGAGCCAAGAGGCGAGGCTCGGGATTAAGCCGCACATTCAGCGCCTCCTCGATCAAGGCATTCTCGTGCCGTGCCAATCCCCGTGGAATACACCACTCCTCCCGGTCAAAAAGCCGGGCACCAACGACTATCGCCCGGTCCAAGATCTCCGCGAGGTCAACAAGCGCGTGGAAGACATCCACCCGACCGTCCCGAACCCGTATAATCTGCTCTCCGGGCTCCCACCATCCCACCAGTGGTATACAGTGCTGGACCTCAAAGACGCCTTCTTCTGTCTCCGCCTCCACCCAACAAGCCAGCCGCTCTTCGCCTTCGAGTGGCGCGACCCGGAGATGGGCATCTCCGGCCAACTGACATGGACACGCCTCCCGCAAGGCTTCAAGAACAGCCCGACACTCTTCAACGAGGCGCTCCATAGGGACCTCGCGGATTTTCGCATCCAGCATCCGGACCTCATCCTCCTCCAGTATGTGGATGATCTCCTCCTCGCCGCGACCTCCGAGCTGGATTGTCAACAAGGCACACGCGCGCTCCTCCAAACACTCGGGAACCTCGGCTATCGCGCGTCCGCGAAAAAGGCCCAAATCTGCCAGAAGCAAGTGAAGTACCTCGGGTATCTGCTCAAGGAAGGCCAACGCTGGCTCACCGAAGCGCGCAAAGAAACAGTGATGGGGCAACCGACACCGAAAACACCACGCCAGCTGCGCGAGTTTCTCGGCAAAGCCGGCTTCTGTCGCCTCTTCATCCCGGGCTTTGCCGAGATGGCCGCGCCACTCTACCCACTCACCAAGCCGGGCACACTGTTTAACTGGGGGCCGGATCAGCAGAAAGCCTACCAAGAGATCAAACAAGCGCTCCTCACCGCCCCAGCGCTCGGGCTCCCAGATCTCACAAAGCCGTTCGAGCTGTTCGTCGATGAGAAGCAAGGCTACGCGAAGGGCGTGCTCACACAGAAGCTCGGCCCGTGGAGGAGGCCAGTGGCCTATCTCTCCAAAAAACTCGATCCAGTGGCCGCCGGCTGGCCACCGTGTCTGCGCATGGTCGCCGCGATTGCCGTGCTCACAAAGGATGCCGGCAAACTCACAATGGGCCAGCCGCTGGTGATCCTCGCGCCACATGCCGTGGAAGCCCTCGTCAAACAGCCGCCGGATAGGTGGCTCTCCAATGCGCGCATGACCCATTACCAAGCGCTCCTCCTCGACACCGAAGCGGCGGCAGCAAAAGGACCGCCGATTCCCAACATTCCACCCCACCAAAAACCAAGAGGAAGGTGGGCTCTGGCCCAAAGAAGAAGAGGAAGGTCTGA

**ePPEmax**

AAGCGCACCGCCGACGGATCCGAGTTCGAGAGCCCTAAGAAAAAGAGAAAAGTGGACAAGAAGTACTCGATCGGCCTCGATATTGGGACTAACTCTGTTGGCTGGGCCGTGATCACCGACGAGTACAAGGTGCCCTCAAAGAAGTTCAAGGTCCTGGGCAACACCGATCGGCATTCCATCAAGAAGAATCTCATTGGCGCTCTCCTGTTCGACAGCGGCGAGACGGCTGAGGCTACGCGGCTCAAGCGCACCGCCCGCAGGCGGTACACGCGCAGGAAGAATCGCATCTGCTACCTGCAGGAGATTTTCTCCAACGAGATGGCGAAGGTTGACGATTCTTTCTTCCACAGGCTGGAGGAGTCATTCCTCGTGGAGGAGGATAAGAAGCACGAGCGGCATCCAATCTTCGGCAACATTGTCGACGAGGTTGCCTACCACGAGAAGTACCCTACGATCTACCATCTGCGGAAGAAGCTCGTGGACTCCACAGATAAGGCGGACCTCCGCCTGATCTACCTCGCTCTGGCCCACATGATTAAGTTCAGGGGCCATTTCCTGATCGAGGGGGATCTCAACCCGGACAATAGCGATGTTGACAAGCTGTTCATCCAGCTCGTGCAGACGTACAACCAGCTCTTCGAGGAGAACCCCATTAATGCGTCAGGCGTCGACGCGAAGGCTATCCTGTCCGCTAGGCTCTCGAAGTCTCGGaagCTCGAGAACCTGATCGCCCAGCTGCCGGGCGAGAAGAAGAACGGCCTGTTCGGGAATCTCATTGCGCTCAGCCTGGGGCTCACGCCCAACTTCAAGTCGAATTTCGATCTCGCTGAGGACGCCAAGCTGCAGCTCTCCAAGGACACATACGACGATGACCTGGATAACCTCCTGGCCCAGATCGGCGATCAGTACGCGGACCTGTTCCTCGCTGCCAAGAATCTGTCGGACGCCATCCTCCTGTCTGATATTCTCAGGGTGAACACCGAGATTACGAAGGCTCCGCTCTCAGCCTCCATGATCAAGCGCTACGACGAGCACCATCAGGATCTGACCCTCCTGAAGGCGCTGGTCAGGCAGCAGCTCCCCGAGAAGTACAAGGAGATCTTCTTCGATCAGTCGAAGAACGGCTACGCTGGGTACATTGACGGCGGGGCCTCTCAGGAGGAGTTCTACAAGTTCATCAAGCCGATTCTGGAGAAGATGGACGGCACGGAGGAGCTGCTGGTGAAGCTCaagCGCGAGGACCTCCTGAGGAAGCAGCGGACATTCGATAACGGCAGCATCCCACACCAGATTCATCTCGGGGAGCTGCACGCTATCCTGAGGAGGCAGGAGGACTTCTACCCTTTCCTCAAGGATAACCGCGAGAAGATCGAGAAGATTCTGACTTTCAGGATCCCGTACTACGTCGGCCCACTCGCTAGGGGCAACTCCCGCTTCGCTTGGATGACCCGCAAGTCAGAGGAGACGATCACGCCGTGGAACTTCGAGGAGGTGGTCGACAAGGGCGCTAGCGCTCAGTCGTTCATCGAGAGGATGACGAATTTCGACAAGAACCTGCCAAATGAGAAGGTGCTCCCTAAGCACTCGCTCCTGTACGAGTACTTCACAGTCTACAACGAGCTGACTAAGGTGAAGTATGTGACCGAGGGCATGAGGAAGCCGGCTTTCCTGTCTGGGGAGCAGAAGAAGGCCATCGTGGACCTCCTGTTCAAGACCAACCGGAAGGTCACGGTTAAGCAGCTCAAGGAGGACTACTTCAAGAAGATTGAGTGCTTCGATTCGGTCGAGATCTCTGGCGTTGAGGACCGCTTCAACGCCTCCCTGGGGACCTACCACGATCTCCTGAAGATCATTAAGGATAAGGACTTCCTGGACAACGAGGAGAATGAGGATATCCTCGAGGACATTGTGCTGACACTCACTCTGTTCGAGGACCGGGAGATGATCGAGGAGCGCCTGAAGACTTACGCCCATCTCTTCGATGACAAGGTCATGAAGCAGCTCAAGAGGAGGAGGTACACCGGCTGGGGGAGGCTGAGCAGGAAGCTCATCAACGGCATTCGGGACAAGCAGTCCGGGAAGACGATCCTCGACTTCCTGAAGAGCGATGGCTTCGCGAACCGCAATTTCATGCAGCTGATTCACGATGACAGCCTCACATTCAAGGAGGATATCCAGAAGGCTCAGGTGAGCGGCCAGGGGGACTCGCTGCACGAGCATATCGCGAACCTCGCTGGCTCGCCAGCTATCAAGAAGGGGATTCTGCAGACCGTGAAGGTTGTGGACGAGCTGGTGAAGGTCATGGGCAGGCACAAGCCTGAGAACATCGTCATTGAGATGGCCCGGGAGAATCAGACCACGCAGAAGGGCCAGAAGAACTCACGCGAGAGGATGAAGAGGATCGAGGAGGGCATTAAGGAGCTGGGGTCCCAGATCCTCAAGGAGCACCCGGTGGAGAACACGCAGCTGCAGAATGAGAAGCTCTACCTGTACTACCTCCAGAATGGCCGCGATATGTATGTGGACCAGGAGCTGGATATTAACAGGCTCAGCGATTACGACGTCGATGCCATCGTTCCACAGTCATTCCTGAAGGATGACTCCATTGACAACAAGGTCCTCACCAGGTCGGACAAGAACCGGGGCAAGTCTGATAATGTTCCTTCAGAGGAGGTCGTTAAGAAGATGAAGAACTACTGGCGCCAGCTCCTGAATGCCAAGCTGATCACGCAGCGGAAGTTCGATAACCTCACAAAGGCTGAGAGGGGCGGGCTCTCTGAGCTGGACAAGGCGGGCTTCATCAAGAGGCAGCTGGTCGAGACACGGCAGATCACTAAGCACGTTGCGCAGATTCTCGACTCACGGATGAACACTAAGTACGATGAGAATGACAAGCTGATCCGCGAGGTGAAGGTCATCACCCTGAAGTCAAAGCTCGTCTCCGACTTCAGGAAGGATTTCCAGTTCTACAAGGTTCGGGAGATCAACAATTACCACCATGCCCATGACGCGTACCTGAACGCGGTGGTCGGCACAGCTCTGATCAAGAAGTACCCAAAGCTCGAGAGCGAGTTCGTGTACGGGGACTACAAGGTTTACGATGTGAGGAAGATGATCGCCAAGTCGGAGCAGGAGATTGGCAAGGCTACCGCCAAGTACTTCTTCTACTCTAACATTATGAATTTCTTCAAGACAGAGATCACTCTGGCCAATGGCGAGATCCGGAAGCGCCCCCTCATCGAGACGAACGGCGAGACGGGGGAGATCGTGTGGGACAAGGGCAGGGATTTCGCGACCGTCAGGAAGGTTCTCTCCATGCCACAAGTGAATATCGTCAAGAAGACAGAGGTCCAGACTGGCGGGTTCTCTAAGGAGTCAATTCTGCCTAAGCGGAACAGCGACAAGCTCATCGCCCGCAAGAAGGACTGGGATCCGAAGAAGTACGGCGGGTTCGACAGCCCCACTGTGGCCTACTCGGTCCTGGTTGTGGCGAAGGTTGAGAAGGGCAAGTCCAAGAAGCTCAAGAGCGTGAAGGAGCTGCTGGGGATCACGATTATGGAGCGCTCCAGCTTCGAGAAGAACCCGATCGATTTCCTGGAGGCGAAGGGCTACAAGGAGGTGAAGAAGGACCTGATCATTAAGCTCCCCAAGTACTCACTCTTCGAGCTGGAGAACGGCAGGAAGCGGATGCTGGCTTCCGCTGGCGAGCTGCAGAAGGGGAACGAGCTGGCTCTGCCGTCCAAGTATGTGAACTTCCTCTACCTGGCCTCCCACTACGAGAAGCTCAAGGGCAGCCCCGAGGACAACGAGCAGAAGCAGCTGTTCGTCGAGCAGCACAAGCATTACCTCGACGAGATCATTGAGCAGATTTCCGAGTTCTCCAAGCGCGTGATCCTGGCCGACGCGAATCTGGATAAGGTCCTCTCCGCGTACAACAAGCACCGCGACAAGCCAATCAGGGAGCAGGCTGAGAATATCATTCATCTCTTCACCCTGACGAACCTCGGCGCCCCTGCTGCTTTCAAGTACTTCGACACAACTATCGATCGCAAGAGGTACACAAGCACTAAGGAGGTCCTGGACGCGACCCTCATCCACCAGTCGATTACCGGCCTCTACGAGACGCGCATCGACCTGTCTCAGCTCGGGGGCGACGAATTCTCCGGGAGCGAGACGCCAGGCACCTCCGAGTCGGCCACCCCAGAATCTGCCACAGTGGTGTCCGGCCAAAAGCAGGACCGCCAGGGCGGAGAACGCAGAAGGTCCCAGCTCGATAGGGATCAGTGTGCCTACTGCAAGGAGAAGGGCCACTGGGCCAAAGACTGCCCGAAAAAGCCGCGCGGCCCACGCGGCCCAAGGCCACAAACATCCCTCCTTCCAAAGAAGAAGCGGAAGGTGGAGCTCAGCGGAGGATCTTCCGGAGGATCTAGCGGCTCCGAGACACCAGGAACATCCGAAAGCGCTACACCAGAATCTAGCGGAGGCTCTTCCGGAGGATCTAGGCCTACCCTCAACATCGAGGATGAGTATCGCCTCCACGAAACCTCCAAAGAACCGGACGTGTCCCTCGGCAGCACATGGCTCAGCGACTTCCCACAAGCGTGGGCCGAAACCGGCGGCATGGGCCTCGCCGTCCGCCAAGCCCCACTCATTATCCCGCTGAAGGCGACCTCCACACCGGTGTCCATCAAGCAGTACCCGATGAGCCAAGAGGCGAGGCTCGGGATTAAGCCGCACATTCAGCGCCTCCTCGATCAAGGCATTCTCGTGCCGTGCCAATCCCCGTGGAATACACCACTCCTCCCGGTCAAAAAGCCGGGCACCAACGACTATCGCCCGGTCCAAGATCTCCGCGAGGTCAACAAGCGCGTGGAAGACATCCACCCGACCGTCCCGAACCCGTATAATCTGCTCTCCGGGCTCCCACCATCCCACCAGTGGTATACAGTGCTGGACCTCAAAGACGCCTTCTTCTGTCTCCGCCTCCACCCAACAAGCCAGCCGCTCTTCGCCTTCGAGTGGCGCGACCCGGAGATGGGCATCTCCGGCCAACTGACATGGACACGCCTCCCGCAAGGCTTCAAGAACAGCCCGACACTCTTCAACGAGGCGCTCCATAGGGACCTCGCGGATTTTCGCATCCAGCATCCGGACCTCATCCTCCTCCAGTATGTGGATGATCTCCTCCTCGCCGCGACCTCCGAGCTGGATTGTCAACAAGGCACACGCGCGCTCCTCCAAACACTCGGGAACCTCGGCTATCGCGCGTCCGCGAAAAAGGCCCAAATCTGCCAGAAGCAAGTGAAGTACCTCGGGTATCTGCTCAAGGAAGGCCAACGCTGGCTCACCGAAGCGCGCAAAGAAACAGTGATGGGGCAACCGACACCGAAAACACCACGCCAGCTGCGCGAGTTTCTCGGCAAAGCCGGCTTCTGTCGCCTCTTCATCCCGGGCTTTGCCGAGATGGCCGCGCCACTCTACCCACTCACCAAGCCGGGCACACTGTTTAACTGGGGGCCGGATCAGCAGAAAGCCTACCAAGAGATCAAACAAGCGCTCCTCACCGCCCCAGCGCTCGGGCTCCCAGATCTCACAAAGCCGTTCGAGCTGTTCGTCGATGAGAAGCAAGGCTACGCGAAGGGCGTGCTCACACAGAAGCTCGGCCCGTGGAGGAGGCCAGTGGCCTATCTCTCCAAAAAACTCGATCCAGTGGCCGCCGGCTGGCCACCGTGTCTGCGCATGGTCGCCGCGATTGCCGTGCTCACAAAGGATGCCGGCAAACTCACAATGGGCCAGCCGCTGGTGATCCTCGCGCCACATGCCGTGGAAGCCCTCGTCAAACAGCCGCCGGATAGGTGGCTCTCCAATGCGCGCATGACCCATTACCAAGCGCTCCTCCTCGACACCGAAGCGGCGGCAGCAAGCGCACCGCCGACGGATCCGAGTTCGAGAGCCCAAAGAAGAAGAGGAAGGTCGGCTCTGGCCCAGCCGCCAAGCGCGTCAAGCTGGACTGA

**CMPE-PPE**

ATGGACCACTACCTCGACATCAGGCTCAGGCCAGACCCAGAGTTCCCACCAGCCCAGCTCATGTCCGTCCTCTTCGGCAAGCTCCACCAGGCCCTCGTGGCCCAGGGCGGCGACAGGATCGGCGTGTCCTTCCCAGACCTCGACGAGTCCAGGTCCAGGCTCGGCGAGAGGCTCCGCATCCACGCCTCCGCCGACGACCTCAGGGCCCTCCTCGCCAGGCCGTGGCTGGAGGGCCTCAGGGACCACCTCCAGTTCGGCGAGCCAGCCGTGGTGCCACACCCAACCCCATACAGGCAAGTGTCCAGGGTGCAAGCCAAGTCCAACCCAGAGAGGCTCAGGAGGAGGCTCATGAGGAGGCACGACCTCTCCGAGGAAGAGGCCAGGAAGCGCATCCCAGACACCGTGGCCAGGGCCCTCGACCTCCCATTCGTGACCCTCAGGTCCCAGTCCACCGGCCAGCACTTCCGCCTCTTCATCAGGCACGGCCCACTCCAGGTGACCGCCGAGGAGGGCGGCTTTACCTGCTACGGCCTCTCCAAGGGCGGCTTCGTGCCGTGGTTCGGCTCCGGCGCCACCAACTTCTCCCTCCTCAAGCAAGCCGGCGACGTGGAGGAGAACCCAGGCCCAATGGACAAGAAGTACTCGATCGGCCTCGATATTGGGACTAACTCTGTTGGCTGGGCCGTGATCACCGACGAGTACAAGGTGCCCTCAAAGAAGTTCAAGGTCCTGGGCAACACCGATCGGCATTCCATCAAGAAGAATCTCATTGGCGCTCTCCTGTTCGACAGCGGCGAGACGGCTGAGGCTACGCGGCTCAAGCGCACCGCCCGCAGGCGGTACACGCGCAGGAAGAATCGCATCTGCTACCTGCAGGAGATTTTCTCCAACGAGATGGCGAAGGTTGACGATTCTTTCTTCCACAGGCTGGAGGAGTCATTCCTCGTGGAGGAGGATAAGAAGCACGAGCGGCATCCAATCTTCGGCAACATTGTCGACGAGGTTGCCTACCACGAGAAGTACCCTACGATCTACCATCTGCGGAAGAAGCTCGTGGACTCCACAGATAAGGCGGACCTCCGCCTGATCTACCTCGCTCTGGCCCACATGATTAAGTTCAGGGGCCATTTCCTGATCGAGGGGGATCTCAACCCGGACAATAGCGATGTTGACAAGCTGTTCATCCAGCTCGTGCAGACGTACAACCAGCTCTTCGAGGAGAACCCCATTAATGCGTCAGGCGTCGACGCGAAGGCTATCCTGTCCGCTAGGCTCTCGAAGTCTCGGCGCCTCGAGAACCTGATCGCCCAGCTGCCGGGCGAGAAGAAGAACGGCCTGTTCGGGAATCTCATTGCGCTCAGCCTGGGGCTCACGCCCAACTTCAAGTCGAATTTCGATCTCGCTGAGGACGCCAAGCTGCAGCTCTCCAAGGACACATACGACGATGACCTGGATAACCTCCTGGCCCAGATCGGCGATCAGTACGCGGACCTGTTCCTCGCTGCCAAGAATCTGTCGGACGCCATCCTCCTGTCTGATATTCTCAGGGTGAACACCGAGATTACGAAGGCTCCGCTCTCAGCCTCCATGATCAAGCGCTACGACGAGCACCATCAGGATCTGACCCTCCTGAAGGCGCTGGTCAGGCAGCAGCTCCCCGAGAAGTACAAGGAGATCTTCTTCGATCAGTCGAAGAACGGCTACGCTGGGTACATTGACGGCGGGGCCTCTCAGGAGGAGTTCTACAAGTTCATCAAGCCGATTCTGGAGAAGATGGACGGCACGGAGGAGCTGCTGGTGAAGCTCAATCGCGAGGACCTCCTGAGGAAGCAGCGGACATTCGATAACGGCAGCATCCCACACCAGATTCATCTCGGGGAGCTGCACGCTATCCTGAGGAGGCAGGAGGACTTCTACCCTTTCCTCAAGGATAACCGCGAGAAGATCGAGAAGATTCTGACTTTCAGGATCCCGTACTACGTCGGCCCACTCGCTAGGGGCAACTCCCGCTTCGCTTGGATGACCCGCAAGTCAGAGGAGACGATCACGCCGTGGAACTTCGAGGAGGTGGTCGACAAGGGCGCTAGCGCTCAGTCGTTCATCGAGAGGATGACGAATTTCGACAAGAACCTGCCAAATGAGAAGGTGCTCCCTAAGCACTCGCTCCTGTACGAGTACTTCACAGTCTACAACGAGCTGACTAAGGTGAAGTATGTGACCGAGGGCATGAGGAAGCCGGCTTTCCTGTCTGGGGAGCAGAAGAAGGCCATCGTGGACCTCCTGTTCAAGACCAACCGGAAGGTCACGGTTAAGCAGCTCAAGGAGGACTACTTCAAGAAGATTGAGTGCTTCGATTCGGTCGAGATCTCTGGCGTTGAGGACCGCTTCAACGCCTCCCTGGGGACCTACCACGATCTCCTGAAGATCATTAAGGATAAGGACTTCCTGGACAACGAGGAGAATGAGGATATCCTCGAGGACATTGTGCTGACACTCACTCTGTTCGAGGACCGGGAGATGATCGAGGAGCGCCTGAAGACTTACGCCCATCTCTTCGATGACAAGGTCATGAAGCAGCTCAAGAGGAGGAGGTACACCGGCTGGGGGAGGCTGAGCAGGAAGCTCATCAACGGCATTCGGGACAAGCAGTCCGGGAAGACGATCCTCGACTTCCTGAAGAGCGATGGCTTCGCGAACCGCAATTTCATGCAGCTGATTCACGATGACAGCCTCACATTCAAGGAGGATATCCAGAAGGCTCAGGTGAGCGGCCAGGGGGACTCGCTGCACGAGCATATCGCGAACCTCGCTGGCTCGCCAGCTATCAAGAAGGGGATTCTGCAGACCGTGAAGGTTGTGGACGAGCTGGTGAAGGTCATGGGCAGGCACAAGCCTGAGAACATCGTCATTGAGATGGCCCGGGAGAATCAGACCACGCAGAAGGGCCAGAAGAACTCACGCGAGAGGATGAAGAGGATCGAGGAGGGCATTAAGGAGCTGGGGTCCCAGATCCTCAAGGAGCACCCGGTGGAGAACACGCAGCTGCAGAATGAGAAGCTCTACCTGTACTACCTCCAGAATGGCCGCGATATGTATGTGGACCAGGAGCTGGATATTAACAGGCTCAGCGATTACGACGTCGATGCCATCGTTCCACAGTCATTCCTGAAGGATGACTCCATTGACAACAAGGTCCTCACCAGGTCGGACAAGAACCGGGGCAAGTCTGATAATGTTCCTTCAGAGGAGGTCGTTAAGAAGATGAAGAACTACTGGCGCCAGCTCCTGAATGCCAAGCTGATCACGCAGCGGAAGTTCGATAACCTCACAAAGGCTGAGAGGGGCGGGCTCTCTGAGCTGGACAAGGCGGGCTTCATCAAGAGGCAGCTGGTCGAGACACGGCAGATCACTAAGCACGTTGCGCAGATTCTCGACTCACGGATGAACACTAAGTACGATGAGAATGACAAGCTGATCCGCGAGGTGAAGGTCATCACCCTGAAGTCAAAGCTCGTCTCCGACTTCAGGAAGGATTTCCAGTTCTACAAGGTTCGGGAGATCAACAATTACCACCATGCCCATGACGCGTACCTGAACGCGGTGGTCGGCACAGCTCTGATCAAGAAGTACCCAAAGCTCGAGAGCGAGTTCGTGTACGGGGACTACAAGGTTTACGATGTGAGGAAGATGATCGCCAAGTCGGAGCAGGAGATTGGCAAGGCTACCGCCAAGTACTTCTTCTACTCTAACATTATGAATTTCTTCAAGACAGAGATCACTCTGGCCAATGGCGAGATCCGGAAGCGCCCCCTCATCGAGACGAACGGCGAGACGGGGGAGATCGTGTGGGACAAGGGCAGGGATTTCGCGACCGTCAGGAAGGTTCTCTCCATGCCACAAGTGAATATCGTCAAGAAGACAGAGGTCCAGACTGGCGGGTTCTCTAAGGAGTCAATTCTGCCTAAGCGGAACAGCGACAAGCTCATCGCCCGCAAGAAGGACTGGGATCCGAAGAAGTACGGCGGGTTCGACAGCCCCACTGTGGCCTACTCGGTCCTGGTTGTGGCGAAGGTTGAGAAGGGCAAGTCCAAGAAGCTCAAGAGCGTGAAGGAGCTGCTGGGGATCACGATTATGGAGCGCTCCAGCTTCGAGAAGAACCCGATCGATTTCCTGGAGGCGAAGGGCTACAAGGAGGTGAAGAAGGACCTGATCATTAAGCTCCCCAAGTACTCACTCTTCGAGCTGGAGAACGGCAGGAAGCGGATGCTGGCTTCCGCTGGCGAGCTGCAGAAGGGGAACGAGCTGGCTCTGCCGTCCAAGTATGTGAACTTCCTCTACCTGGCCTCCCACTACGAGAAGCTCAAGGGCAGCCCCGAGGACAACGAGCAGAAGCAGCTGTTCGTCGAGCAGCACAAGCATTACCTCGACGAGATCATTGAGCAGATTTCCGAGTTCTCCAAGCGCGTGATCCTGGCCGACGCGAATCTGGATAAGGTCCTCTCCGCGTACAACAAGCACCGCGACAAGCCAATCAGGGAGCAGGCTGAGAATATCATTCATCTCTTCACCCTGACGAACCTCGGCGCCCCTGCTGCTTTCAAGTACTTCGACACAACTATCGATCGCAAGAGGTACACAAGCACTAAGGAGGTCCTGGACGCGACCCTCATCCACCAGTCGATTACCGGCCTCTACGAGACGCGCATCGACCTGTCTCAGCTCGGGGGCGACGAATTCCCAAAGAAGAAGCGGAAGGTGGAGCTCAGCGGAGGATCTTCCGGAGGATCTAGCGGCTCCGAGACACCAGGAACATCCGAAAGCGCTACACCAGAATCTAGCGGAGGCTCTTCCGGAGGATCTAGGCCTACCCTCAACATCGAGGATGAGTATCGCCTCCACGAAACCTCCAAAGAACCGGACGTGTCCCTCGGCAGCACATGGCTCAGCGACTTCCCACAAGCGTGGGCCGAAACCGGCGGCATGGGCCTCGCCGTCCGCCAAGCCCCACTCATTATCCCGCTGAAGGCGACCTCCACACCGGTGTCCATCAAGCAGTACCCGATGAGCCAAGAGGCGAGGCTCGGGATTAAGCCGCACATTCAGCGCCTCCTCGATCAAGGCATTCTCGTGCCGTGCCAATCCCCGTGGAATACACCACTCCTCCCGGTCAAAAAGCCGGGCACCAACGACTATCGCCCGGTCCAAGATCTCCGCGAGGTCAACAAGCGCGTGGAAGACATCCACCCGACCGTCCCGAACCCGTATAATCTGCTCTCCGGGCTCCCACCATCCCACCAGTGGTATACAGTGCTGGACCTCAAAGACGCCTTCTTCTGTCTCCGCCTCCACCCAACAAGCCAGCCGCTCTTCGCCTTCGAGTGGCGCGACCCGGAGATGGGCATCTCCGGCCAACTGACATGGACACGCCTCCCGCAAGGCTTCAAGAACAGCCCGACACTCTTCAACGAGGCGCTCCATAGGGACCTCGCGGATTTTCGCATCCAGCATCCGGACCTCATCCTCCTCCAGTATGTGGATGATCTCCTCCTCGCCGCGACCTCCGAGCTGGATTGTCAACAAGGCACACGCGCGCTCCTCCAAACACTCGGGAACCTCGGCTATCGCGCGTCCGCGAAAAAGGCCCAAATCTGCCAGAAGCAAGTGAAGTACCTCGGGTATCTGCTCAAGGAAGGCCAACGCTGGCTCACCGAAGCGCGCAAAGAAACAGTGATGGGGCAACCGACACCGAAAACACCACGCCAGCTGCGCGAGTTTCTCGGCAAAGCCGGCTTCTGTCGCCTCTTCATCCCGGGCTTTGCCGAGATGGCCGCGCCACTCTACCCACTCACCAAGCCGGGCACACTGTTTAACTGGGGGCCGGATCAGCAGAAAGCCTACCAAGAGATCAAACAAGCGCTCCTCACCGCCCCAGCGCTCGGGCTCCCAGATCTCACAAAGCCGTTCGAGCTGTTCGTCGATGAGAAGCAAGGCTACGCGAAGGGCGTGCTCACACAGAAGCTCGGCCCGTGGAGGAGGCCAGTGGCCTATCTCTCCAAAAAACTCGATCCAGTGGCCGCCGGCTGGCCACCGTGTCTGCGCATGGTCGCCGCGATTGCCGTGCTCACAAAGGATGCCGGCAAACTCACAATGGGCCAGCCGCTGGTGATCCTCGCGCCACATGCCGTGGAAGCCCTCGTCAAACAGCCGCCGGATAGGTGGCTCTCCAATGCGCGCATGACCCATTACCAAGCGCTCCTCCTCGACACCGATCGCGTCCAGTTCGGCCCAGTGGTCGCCCTCAATCCGGCGACACTGCTGCCACTCCCAGAGGAGGGCCTCCAACACAACTGTCTGGATATTCTCGCGGAAGCGCATGGCACAAGGCCAGACCTCACAGATCAACCGCTCCCGGATGCGGATCACACATGGTATACCGACGGCTCCTCTCTGCTCCAAGAGGGCCAAAGGAAAGCCGGCGCCGCGGTGACCACAGAAACAGAAGTGATCTGGGCCAAGGCCCTCCCAGCCGGCACATCCGCGCAAAGGGCGGAACTCATCGCGCTCACACAAGCCCTCAAGATGGCCGAGGGCAAGAAGCTCAACGTCTACACAGACTCCCGCTATGCCTTCGCCACCGCCCACATTCACGGCGAAATCTATAGGAGGCGCGGCTGGCTCACAAGCGAGGGGAAGGAGATCAAGAACAAGGATGAGATCCTCGCGCTGCTCAAGGCCCTCTTTCTCCCGAAGCGCCTCTCCATCATCCACTGTCCGGGCCACCAAAAGGGGCACTCCGCGGAAGCGAGGGGCAATAGGATGGCCGATCAAGCCGCGCGCAAAGCCGCGATTACCGAAACCCCAGACACATCCACCCTCCTCATCGAAAACTCCTCCCCAAAGCGGCGGCAGCCCGAAGAAGAAAAGGAAGGTGTGA

**CMPE-ePPE**

ATGGACCACTACCTCGACATCAGGCTCAGGCCAGACCCAGAGTTCCCACCAGCCCAGCTCATGTCCGTCCTCTTCGGCAAGCTCCACCAGGCCCTCGTGGCCCAGGGCGGCGACAGGATCGGCGTGTCCTTCCCAGACCTCGACGAGTCCAGGTCCAGGCTCGGCGAGAGGCTCCGCATCCACGCCTCCGCCGACGACCTCAGGGCCCTCCTCGCCAGGCCGTGGCTGGAGGGCCTCAGGGACCACCTCCAGTTCGGCGAGCCAGCCGTGGTGCCACACCCAACCCCATACAGGCAAGTGTCCAGGGTGCAAGCCAAGTCCAACCCAGAGAGGCTCAGGAGGAGGCTCATGAGGAGGCACGACCTCTCCGAGGAAGAGGCCAGGAAGCGCATCCCAGACACCGTGGCCAGGGCCCTCGACCTCCCATTCGTGACCCTCAGGTCCCAGTCCACCGGCCAGCACTTCCGCCTCTTCATCAGGCACGGCCCACTCCAGGTGACCGCCGAGGAGGGCGGCTTTACCTGCTACGGCCTCTCCAAGGGCGGCTTCGTGCCGTGGTTCGGCTCCGGCGCCACCAACTTCTCCCTCCTCAAGCAAGCCGGCGACGTGGAGGAGAACCCAGGCCCAATGCCTAAGAAAAAGAGAAAAGTGGACAAGAAGTACTCGATCGGCCTCGATATTGGGACTAACTCTGTTGGCTGGGCCGTGATCACCGACGAGTACAAGGTGCCCTCAAAGAAGTTCAAGGTCCTGGGCAACACCGATCGGCATTCCATCAAGAAGAATCTCATTGGCGCTCTCCTGTTCGACAGCGGCGAGACGGCTGAGGCTACGCGGCTCAAGCGCACCGCCCGCAGGCGGTACACGCGCAGGAAGAATCGCATCTGCTACCTGCAGGAGATTTTCTCCAACGAGATGGCGAAGGTTGACGATTCTTTCTTCCACAGGCTGGAGGAGTCATTCCTCGTGGAGGAGGATAAGAAGCACGAGCGGCATCCAATCTTCGGCAACATTGTCGACGAGGTTGCCTACCACGAGAAGTACCCTACGATCTACCATCTGCGGAAGAAGCTCGTGGACTCCACAGATAAGGCGGACCTCCGCCTGATCTACCTCGCTCTGGCCCACATGATTAAGTTCAGGGGCCATTTCCTGATCGAGGGGGATCTCAACCCGGACAATAGCGATGTTGACAAGCTGTTCATCCAGCTCGTGCAGACGTACAACCAGCTCTTCGAGGAGAACCCCATTAATGCGTCAGGCGTCGACGCGAAGGCTATCCTGTCCGCTAGGCTCTCGAAGTCTCGGCGCCTCGAGAACCTGATCGCCCAGCTGCCGGGCGAGAAGAAGAACGGCCTGTTCGGGAATCTCATTGCGCTCAGCCTGGGGCTCACGCCCAACTTCAAGTCGAATTTCGATCTCGCTGAGGACGCCAAGCTGCAGCTCTCCAAGGACACATACGACGATGACCTGGATAACCTCCTGGCCCAGATCGGCGATCAGTACGCGGACCTGTTCCTCGCTGCCAAGAATCTGTCGGACGCCATCCTCCTGTCTGATATTCTCAGGGTGAACACCGAGATTACGAAGGCTCCGCTCTCAGCCTCCATGATCAAGCGCTACGACGAGCACCATCAGGATCTGACCCTCCTGAAGGCGCTGGTCAGGCAGCAGCTCCCCGAGAAGTACAAGGAGATCTTCTTCGATCAGTCGAAGAACGGCTACGCTGGGTACATTGACGGCGGGGCCTCTCAGGAGGAGTTCTACAAGTTCATCAAGCCGATTCTGGAGAAGATGGACGGCACGGAGGAGCTGCTGGTGAAGCTCAATCGCGAGGACCTCCTGAGGAAGCAGCGGACATTCGATAACGGCAGCATCCCACACCAGATTCATCTCGGGGAGCTGCACGCTATCCTGAGGAGGCAGGAGGACTTCTACCCTTTCCTCAAGGATAACCGCGAGAAGATCGAGAAGATTCTGACTTTCAGGATCCCGTACTACGTCGGCCCACTCGCTAGGGGCAACTCCCGCTTCGCTTGGATGACCCGCAAGTCAGAGGAGACGATCACGCCGTGGAACTTCGAGGAGGTGGTCGACAAGGGCGCTAGCGCTCAGTCGTTCATCGAGAGGATGACGAATTTCGACAAGAACCTGCCAAATGAGAAGGTGCTCCCTAAGCACTCGCTCCTGTACGAGTACTTCACAGTCTACAACGAGCTGACTAAGGTGAAGTATGTGACCGAGGGCATGAGGAAGCCGGCTTTCCTGTCTGGGGAGCAGAAGAAGGCCATCGTGGACCTCCTGTTCAAGACCAACCGGAAGGTCACGGTTAAGCAGCTCAAGGAGGACTACTTCAAGAAGATTGAGTGCTTCGATTCGGTCGAGATCTCTGGCGTTGAGGACCGCTTCAACGCCTCCCTGGGGACCTACCACGATCTCCTGAAGATCATTAAGGATAAGGACTTCCTGGACAACGAGGAGAATGAGGATATCCTCGAGGACATTGTGCTGACACTCACTCTGTTCGAGGACCGGGAGATGATCGAGGAGCGCCTGAAGACTTACGCCCATCTCTTCGATGACAAGGTCATGAAGCAGCTCAAGAGGAGGAGGTACACCGGCTGGGGGAGGCTGAGCAGGAAGCTCATCAACGGCATTCGGGACAAGCAGTCCGGGAAGACGATCCTCGACTTCCTGAAGAGCGATGGCTTCGCGAACCGCAATTTCATGCAGCTGATTCACGATGACAGCCTCACATTCAAGGAGGATATCCAGAAGGCTCAGGTGAGCGGCCAGGGGGACTCGCTGCACGAGCATATCGCGAACCTCGCTGGCTCGCCAGCTATCAAGAAGGGGATTCTGCAGACCGTGAAGGTTGTGGACGAGCTGGTGAAGGTCATGGGCAGGCACAAGCCTGAGAACATCGTCATTGAGATGGCCCGGGAGAATCAGACCACGCAGAAGGGCCAGAAGAACTCACGCGAGAGGATGAAGAGGATCGAGGAGGGCATTAAGGAGCTGGGGTCCCAGATCCTCAAGGAGCACCCGGTGGAGAACACGCAGCTGCAGAATGAGAAGCTCTACCTGTACTACCTCCAGAATGGCCGCGATATGTATGTGGACCAGGAGCTGGATATTAACAGGCTCAGCGATTACGACGTCGATGCCATCGTTCCACAGTCATTCCTGAAGGATGACTCCATTGACAACAAGGTCCTCACCAGGTCGGACAAGAACCGGGGCAAGTCTGATAATGTTCCTTCAGAGGAGGTCGTTAAGAAGATGAAGAACTACTGGCGCCAGCTCCTGAATGCCAAGCTGATCACGCAGCGGAAGTTCGATAACCTCACAAAGGCTGAGAGGGGCGGGCTCTCTGAGCTGGACAAGGCGGGCTTCATCAAGAGGCAGCTGGTCGAGACACGGCAGATCACTAAGCACGTTGCGCAGATTCTCGACTCACGGATGAACACTAAGTACGATGAGAATGACAAGCTGATCCGCGAGGTGAAGGTCATCACCCTGAAGTCAAAGCTCGTCTCCGACTTCAGGAAGGATTTCCAGTTCTACAAGGTTCGGGAGATCAACAATTACCACCATGCCCATGACGCGTACCTGAACGCGGTGGTCGGCACAGCTCTGATCAAGAAGTACCCAAAGCTCGAGAGCGAGTTCGTGTACGGGGACTACAAGGTTTACGATGTGAGGAAGATGATCGCCAAGTCGGAGCAGGAGATTGGCAAGGCTACCGCCAAGTACTTCTTCTACTCTAACATTATGAATTTCTTCAAGACAGAGATCACTCTGGCCAATGGCGAGATCCGGAAGCGCCCCCTCATCGAGACGAACGGCGAGACGGGGGAGATCGTGTGGGACAAGGGCAGGGATTTCGCGACCGTCAGGAAGGTTCTCTCCATGCCACAAGTGAATATCGTCAAGAAGACAGAGGTCCAGACTGGCGGGTTCTCTAAGGAGTCAATTCTGCCTAAGCGGAACAGCGACAAGCTCATCGCCCGCAAGAAGGACTGGGATCCGAAGAAGTACGGCGGGTTCGACAGCCCCACTGTGGCCTACTCGGTCCTGGTTGTGGCGAAGGTTGAGAAGGGCAAGTCCAAGAAGCTCAAGAGCGTGAAGGAGCTGCTGGGGATCACGATTATGGAGCGCTCCAGCTTCGAGAAGAACCCGATCGATTTCCTGGAGGCGAAGGGCTACAAGGAGGTGAAGAAGGACCTGATCATTAAGCTCCCCAAGTACTCACTCTTCGAGCTGGAGAACGGCAGGAAGCGGATGCTGGCTTCCGCTGGCGAGCTGCAGAAGGGGAACGAGCTGGCTCTGCCGTCCAAGTATGTGAACTTCCTCTACCTGGCCTCCCACTACGAGAAGCTCAAGGGCAGCCCCGAGGACAACGAGCAGAAGCAGCTGTTCGTCGAGCAGCACAAGCATTACCTCGACGAGATCATTGAGCAGATTTCCGAGTTCTCCAAGCGCGTGATCCTGGCCGACGCGAATCTGGATAAGGTCCTCTCCGCGTACAACAAGCACCGCGACAAGCCAATCAGGGAGCAGGCTGAGAATATCATTCATCTCTTCACCCTGACGAACCTCGGCGCCCCTGCTGCTTTCAAGTACTTCGACACAACTATCGATCGCAAGAGGTACACAAGCACTAAGGAGGTCCTGGACGCGACCCTCATCCACCAGTCGATTACCGGCCTCTACGAGACGCGCATCGACCTGTCTCAGCTCGGGGGCGACGAATTCTCCGGGAGCGAGACGCCAGGCACCTCCGAGTCGGCCACCCCAGAATCTGCCACAGTGGTGTCCGGCCAAAAGCAGGACCGCCAGGGCGGAGAACGCAGAAGGTCCCAGCTCGATAGGGATCAGTGTGCCTACTGCAAGGAGAAGGGCCACTGGGCCAAAGACTGCCCGAAAAAGCCGCGCGGCCCACGCGGCCCAAGGCCACAAACATCCCTCCTTCCAAAGAAGAAGCGGAAGGTGGAGCTCAGCGGAGGATCTTCCGGAGGATCTAGCGGCTCCGAGACACCAGGAACATCCGAAAGCGCTACACCAGAATCTAGCGGAGGCTCTTCCGGAGGATCTAGGCCTACCCTCAACATCGAGGATGAGTATCGCCTCCACGAAACCTCCAAAGAACCGGACGTGTCCCTCGGCAGCACATGGCTCAGCGACTTCCCACAAGCGTGGGCCGAAACCGGCGGCATGGGCCTCGCCGTCCGCCAAGCCCCACTCATTATCCCGCTGAAGGCGACCTCCACACCGGTGTCCATCAAGCAGTACCCGATGAGCCAAGAGGCGAGGCTCGGGATTAAGCCGCACATTCAGCGCCTCCTCGATCAAGGCATTCTCGTGCCGTGCCAATCCCCGTGGAATACACCACTCCTCCCGGTCAAAAAGCCGGGCACCAACGACTATCGCCCGGTCCAAGATCTCCGCGAGGTCAACAAGCGCGTGGAAGACATCCACCCGACCGTCCCGAACCCGTATAATCTGCTCTCCGGGCTCCCACCATCCCACCAGTGGTATACAGTGCTGGACCTCAAAGACGCCTTCTTCTGTCTCCGCCTCCACCCAACAAGCCAGCCGCTCTTCGCCTTCGAGTGGCGCGACCCGGAGATGGGCATCTCCGGCCAACTGACATGGACACGCCTCCCGCAAGGCTTCAAGAACAGCCCGACACTCTTCAACGAGGCGCTCCATAGGGACCTCGCGGATTTTCGCATCCAGCATCCGGACCTCATCCTCCTCCAGTATGTGGATGATCTCCTCCTCGCCGCGACCTCCGAGCTGGATTGTCAACAAGGCACACGCGCGCTCCTCCAAACACTCGGGAACCTCGGCTATCGCGCGTCCGCGAAAAAGGCCCAAATCTGCCAGAAGCAAGTGAAGTACCTCGGGTATCTGCTCAAGGAAGGCCAACGCTGGCTCACCGAAGCGCGCAAAGAAACAGTGATGGGGCAACCGACACCGAAAACACCACGCCAGCTGCGCGAGTTTCTCGGCAAAGCCGGCTTCTGTCGCCTCTTCATCCCGGGCTTTGCCGAGATGGCCGCGCCACTCTACCCACTCACCAAGCCGGGCACACTGTTTAACTGGGGGCCGGATCAGCAGAAAGCCTACCAAGAGATCAAACAAGCGCTCCTCACCGCCCCAGCGCTCGGGCTCCCAGATCTCACAAAGCCGTTCGAGCTGTTCGTCGATGAGAAGCAAGGCTACGCGAAGGGCGTGCTCACACAGAAGCTCGGCCCGTGGAGGAGGCCAGTGGCCTATCTCTCCAAAAAACTCGATCCAGTGGCCGCCGGCTGGCCACCGTGTCTGCGCATGGTCGCCGCGATTGCCGTGCTCACAAAGGATGCCGGCAAACTCACAATGGGCCAGCCGCTGGTGATCCTCGCGCCACATGCCGTGGAAGCCCTCGTCAAACAGCCGCCGGATAGGTGGCTCTCCAATGCGCGCATGACCCATTACCAAGCGCTCCTCCTCGACACCGATCGCGTCCAGTTCGGCCCAGTGGTCGCCCTCAATCCGGCGACACTGCTGCCACTCCCAGAGGAGGGCCTCCAACACAACTGTCTGGATATTCTCGCGGAAGCGCATGGCACAAGGCCAGACCTCACAGATCAACCGCTCAGCGGCGGCAGCCCGAAGAAGAAAAGGAAGGTGTGA
