# Supplementary material for: Efficient and versatile multiplex prime editing in hexaploid wheat
Source: Genome Biol. 2023 Jun 29;24:156. doi: 10.1186/s13059-023-02990-1 (PMC10308706; doi:10.1186/s13059-023-02990-1)
Supplement: Supplementary file 4 — Additional file 4. Review history. [file 13059_2023_2990_MOESM4_ESM.docx]

Review History

**First round of review**

**Reviewer 1**

**Were you able to assess all statistics in the manuscript, including the appropriateness of statistical tests used?**

No.

**Comments to author:**

In this manuscript (GBIO-D-23-00035), Ni et al. reported three independent methods to improve PE editing efficiency in wheat protoplast, including the 3' terminus modifications (tevopreQ1), V223A mutation into the M-MLV RT, and NLS optimization. Furthermore, the combination of the above three methods can synergistically improve PE efficiency in wheat, thus generated the ePPEplus. Based on the upgraded ePPEplus, Csy4-mediated multiplex PE editing obtained simultaneous editing of up to ten or eight genes in both wheat protoplasts and plants. The data of the manuscript is sufficient to support the conclusions. The enhanced ePPEplus overcomes the allohexaploidy of wheat and make it possible to edit multiple genes in wheat. Therefore, it is important for improving the agronomic traits in wheat in future. However, the tevopreQ1 and bpNLS has already been reported in plants (Li et al, 2022; Zhou et al, 2022) and human cells (Chen et al, 2021; Liu et al, 2021) to improve the PE editing efficiency. The main innovation of this manuscript was the V223A mutation of M-MLV RT, which makes the novelty limited. Collectively, I think this manuscript may not be suitable for publication in GB journal with the controversial novelty.

Here are some minor concerns:

1. The testing of the four additional RNA structural motifs seems meaningless, because four or five consecutive Ts existed in the motifs, which may terminate the transcription of the U6 or U3 promoter.

2. In Fig2d, ePPEmax has two elements (NLS and Cas9max) different from the other vectors and cannot be regarded as a rigorous control. The ePPEmax* was not equal the combination of ePPEmax and ePPE*. The data of ePPEmax can be removed.

3. Fold change in Fig 1d, 2c, 2f, 3c was confused. Please explain the detail calculation in the method.

4. Line 191-193, the "up to 109.3 fold, 1399.7-fold" can not be found in FigS6c and S6d, please explain the calculation method.

**Reviewer 2**

**Were you able to assess all statistics in the manuscript, including the appropriateness of statistical tests used?**

No.

**Comments to author:**

This study systematically optimizes various aspect of prime editing in wheat. The authors identify several optimizations and demonstrate the potential for using these for multiplexing in wheat protoplasts and plants. All optimizations/mutants were previously known, but they are the first to put them all together and show an improvement in prime editing activity. Such studies are urgently required as prime editing has relatively low efficiency in plants and a lot of plant researchers want to be able to use the technology. These results will be very interesting to the plant biotechnology community for sure. I am less certain for non-plant fields, but this is not my area.

Overall I find the experiments fairly well designed and executed. The results are clear and well defined. I do have a number of textual and experimental requests that should be addressed for it to be suitable for publication. Below, please find my specific comments. They are in order line by line, not by importance.

L32: what does it mean to downregulate genes with CRISPR? As in CRISPR interference? I also don't understand the emphasis at the start of the sentence "even for common wheat". Wheat was one of the first plant species reported to be CRISPR edited back in 2013 (one of the first three papers, Shan et al., 2013). This reference should be used instead of reviews.

L35-37: "However, these studies are limited in that they mainly produce a single mutation type (e.g., gene knockout), yield imprecise mutations (usually random small insertions or deletions) and can edit only a limited number of genes at a time (generally just one)." Gene knockout via NHEJ is the same thing as imprecise mutations. References 8-10 all have multiplexing in their titles, therefore "generally just one" is incorrect.

L53-54: Prime editing is inefficient in all plant species, common wheat and polyploids are not special. For this comment to stay, please provide a reference to experimental evidence that prime editing is more difficult in wheat/polyploids. I know a lot of people like to say it, but I do not think there is much (good) data to back this up.

The crystal structure does not need to be included. All AA changes were previously identified.

L111: claim that the V223A increases processivity is not supported by the data. The data just show increased PE efficiency. How that is actually done is unclear. I think: to alanine probably plays an important role… would be more accurate.

L123: "Among these engineered ePPEs, ePPEmax* showed the highest editing efficiency, followed by ePPEmax and ePPE*, resulting in 3.1-fold, 3.0-fold and 2.6-fold higher activity than ePPE, respectively (Fig. 2e,f)." I am doubtful of this claim. They look more or less equivalent to me. Especially ePPEmax* and ePPEmax. Statistics are needed to support this claim.

L165: "pegRNAs or epegRNAs were co-transformed with ePPEplus into wheat protoplasts. Targeted amplicon sequencing demonstrated that epegRNA induces higher activity for multiplex genome editing, from 1.3-fold to 4.2-fold greater than with pegRNA regardless of the processing strategy used". I disagree with this interpretation. As stated in the next sentence, the csy4 treatment with the standard pegRNA appears to work quite well, possibly even the same as the epegRNA. A statistical test is needed. These results are very interesting. It is curious why the Csy4 tail did not help in the experiments at the start (Figure 1). Perhaps pol II vs III? A combination of csy4 and the evopreq1? Be sure to discuss.

178-179: I don't follow the problems about Pol III promoter cloning. I can understand insert size at a certain point, once there are ~6 or so, @500 bp per Pol III, that's 3 kb in a plasmid. But Golden Gate cloning is rather efficient. And cumbersome delivery? Of what, protoplasts or agro transformation? For protoplasts pools of plasmids can be used. For agro, unless the plasmid is already ~25kb, it won't be a problem. Plant transformation efficiency usually only goes down after ~30 kb or so. I would just remove this comment. The csy4 results speak for themselves, the efficiency is highest and efficient, so we go with that.

L185: The authors need to be careful on claims about multiplex genome editing when using protoplasts. As the results are based on a population of individual cells, it is not possible to determine if any of the cells contain all or at least >1 edits.

Figure 5: I think the T0 data would be better represented as a scattered bar plot as done for all figures preceding it, with mutation frequency on the y-axis and individual plants as dots. Then the readers can appreciate the level of mutagenesis in the plants. This also clarifies how the authors define het and homozygous (it looks like it is done by eye, not very accurate and subject to Sanger quality). Thresholds can be drawn on the graph. It is unfortunate the authors decided to use Sanger sequencing for this part of the analysis. It is much harder to get accurate quantifications than with NGS, but tools like ICE or TIDE should work.

L212: I'm not sure why the subgenome analysis is only done in the plant work? Why was it omitted from the protoplast work?

To demonstrate the utility of the tool for plant improvement/functional genomics, the authors need to demonstrate transmission of the edits in next generation. Transgene free, null segregants are required for this analysis. This is standard in the field.

L272: replace "diploid plants" with "rice".

L333: I would like to see the full plasmid sequences used (for the key variants and binary plasmids) rather than just the components. That is the only way to ensure reproducibility. Ideally .gb files would be included. Reduce the line spacing so the file is not huge. Or submit at a repository.

L346: I do not know how the wheat protoplast transfections were performed. The reference 52 is a review article. Most important, are the transfections normalized for transfection efficiency? Many of the differences reported may be due to transfection efficiency.

**Reviewer 3**

**Were you able to assess all statistics in the manuscript, including the appropriateness of statistical tests used?**

No.

**Comments to author:**

The manuscript entitled "Efficient and versatile multiplex prime editing in hexaploid wheat" (GBIO-D-23-00035) submitted by Ni et al., tested a series of reassembled prime editors by engineering both the pegRNA and the protein components of PE and demonstrated multiple prime editing activity in common wheat. Because PE strategy can perform precise editing without DNA double-strand break and donor DNA templates, it has attracted extensive attention of researchers. A lot of effort has been put into PE improvement, but so far, the overall efficiency of PE is still low and site-dependent, especially in plants. In this manuscript, the authors provided diverse data to demonstrate that reassembled PE vectors can created multi-site prime editing mutants in wheat, a polyploid plant. In general, although the paper does not show obvious innovations in the construction of PE vectors and application strategies, the result that the authors can obtain stable multi-edited wheat mutants based on their CMPE-ePPEplus system is still worthy of affirmation. Together, I think this manuscript could be considered for publication in Genome Biology with appropriate and necessary revisions.

Here are some comments:

1- The author needs to describe more about why they chose V223A mutation for the M-MLV reverse transcriptase. Although they wrote "Previous studies have reported that mutations at positions F156, V223 and F309 in the M-MLV RT can enhance the processivity and fidelity of reverse transcription" in line 99-100 and listed the reference, I still think this part needs more information. How to make the mutation? Why V223A not V223X (X refers other amino acids)? So does the F156 and F309 sites. I think these key data should be supplemented as much as possible. It will be more useful to the readers if more information added.

2- I noticed the F309N also showed an enhanced frequency, why the author did not try F309N+V223A? A corresponding explanation or discussion should be given for the choice of this design.

3- The ePPEplus brought an increased byproducts compared with other PE editors, did you analyze the mutation type? What kind of mutation increase caused by M-MLV mutation? These data should also be supplemented, if experimental conditions permit.

4- In multiplex PE part, the tRNA shown a limited efficiency. Please noted which tRNA used here. How this tRNA worked on Cas9 NHEJ in wheat protoplasts used the same vector structure without reverse transcriptase and H840.

5- The multi-site editing strategy of the Csy4 and its efficiency comparison with tRNA and ribozyme elements have been clearly compared (Tang et al., 2019; DOI: 10.1111/pbi.13068). Based on a fuller and more accurate research background introduction, the author should cite and discuss the research work of Tang et al. (2019) in the paper.

**Authors Response**

**Point-by-point responses to the reviewers’ comments:**

Reviewer #1:

In this manuscript (GBIO-D-23-00035), Ni et al. reported three independent methods to improve PE editing efficiency in wheat protoplast, including the 3' terminus modifications (tevopreQ1), V223A mutation into the M-MLV RT, and NLS optimization. Furthermore, the combination of the above three methods can synergistically improve PE efficiency in wheat, thus generated the ePPEplus. Based on the upgraded ePPEplus, Csy4-mediated multiplex PE editing obtained simultaneous editing of up to ten or eight genes in both wheat protoplasts and plants. The data of the manuscript is sufficient to support the conclusions. The enhanced ePPEplus overcomes the allohexaploidy of wheat and make it possible to edit multiple genes in wheat. Therefore, it is important for improving the agronomic traits in wheat in future. However, the tevopreQ1 and bpNLS has already been reported in plants (Li et al, 2022; Zhou et al, 2022) and human cells (Chen et al, 2021; Liu et al, 2021) to improve the PE editing efficiency. The main innovation of this manuscript was the V223A mutation of M-MLV RT, which makes the novelty limited. Collectively, I think this manuscript may not be suitable for publication in GB journal with the controversial novelty.

*Response: Prime editing, a versatile genome editing tool, has been successfully demonstrated to introduce flexible mutations in several targets in plants. However, this technique has rarely been applied for crop trait improvement due to its limited efficiency. Considerable efforts have been devoted to optimizing the activities of plant prime editing systems. For example, driving pegRNA expression with enhanced promoters resulted in editing rates ranging from 0 to 43.8% in maize (Jiang et al., Genome Biology, 2020); using a melting temperature of 30°C for the prime binding site (PBS) sequence in pegRNA and editing the target with dual pegRNAs increased the editing efficiency from 2.9- to 17.4-fold in rice protoplasts (Lin et al., Nature Biotechnology, 2020); an improved PE3 system combining a PE with N-fusion M-MLV and synonymous mutations in the RT template increased editing activity from 5.2% to 53% in transgenic rice plants and two- to three-fold in maize protoplasts (Wen et al., Nature plants, 2021); engineering the nCas9-RT fusion protein by deleting the RNase H domain and adding nucleic capsid protein improved the editing efficiency ~5.4-fold in transgenic rice plants (Zong et al., Nature Biotechnology, 2022); PEmax architecture and the use of epegRNA greatly improved prime editing efficiency (from 64.58% to 77.08%) and increased the rate of homozygous mutations in rice plants (Li et al., Genome Biology, 2022; Jiang et al., Molecular Plant, 2022). Nevertheless, the editing efficiencies of these engineered prime editors are still low and site dependent. In addition, these prime editors tend to exhibit poor capability for targeting multiple sites at the same time and primarily limiting their use to diploid crops (e.g., rice and maize).*

*The allohexaploidy and functional gene redundancy of common wheat make it a daunting task to induce any mutations efficiently in this crop compared to diploid plants. Therefore, to date, prime editing has only been reported for wheat protoplasts, and the frequency was very low, with a maximum of only 1.8% and an average of ~0.9% (Lin et al., Nature Biotechnology, 2020; Zong et al., Nature Biotechnology, 2022). Moreover, no stable, precisely prime-edited wheat plants have been obtained, severely limiting the use of prime editing in wheat. These observations highlight the urgent need for improving this technique for common wheat and other polyploid plants. In addition, multiplex prime editing would be important for trait improvement and multiple traits stacking in hexaploid wheat, but such studies have not yet been reported. Therefore, in this study, we focused on overcoming the difficulties of applying prime editing to wheat and developed a more powerful prime editing system for hexaploid wheat.*

*We systematically optimized the prime editing system by focusing on three issues: modifying the 3' terminus of pegRNA, introducing point mutations into the M-MLV RT, and NLS optimization. Indeed, tevopreQ1 and NLS optimization performed well for diploid rice plants and human cells (Chen et al., Cell, 2021; Liu et al., Nature Communications, 2021; Li et al., Genome Biology, 2022; Jiang et al., Molecular Plant, 2022; Zong et al., Nature Biotechnology, 2022; Zou et al., Science China Life Sciences, 2022), but it was unclear if these two strategies would be viable for wheat, making it worthwhile to investigate their performance. Besides these two methods, we determined that introducing the V223A mutation into M-MLV RT improved PE efficiency, which has never been reported. Individually, these three methods enhanced the editing efficiency of wheat cells ~2.8-fold to 3.1-fold. Importantly, we stacked these three independent modifications together, resulting in ePPEplus, which induced an average editing frequency of 6.6% and up to 18.9%, representing a substantial average of 33.0-fold improvement in editing efficiency compared to PPE and an average of 6.4-fold compared to ePPE. Therefore, the combination of epegRNA with ePPEplus developed in this study makes it possible to edit formerly challenging target sites in common wheat, thus expanding the scope and capabilities of prime editing.*

*Importantly, we demonstrated the potential for using upgraded ePPEplus for multiplexing in wheat protoplasts and generated stable transgenic plants for the first time, which is highly desirable for plants, especially polyploid species. After thoroughly comparing the four different pegRNA processing strategies, we achieved simultaneous editing of ten genes at frequencies ranging from 7.4% to 10.3% in protoplasts (an average of 49.1-fold higher than PPE and an average of 10.5-fold higher than PPE) and up to eight genes at frequencies of up to 74.5% in transgenic wheat plants using the Csy4-processing system. In addition, we demonstrated that the mutations could be stably transmitted to the next generation and that a transgene-free plant carrying only the desired DNA sequence change could be obtained through genetic segregation, as described in the revised manuscript. To our knowledge, this is the first report demonstrating efficient prime editing and the production of stable, transgene-free multiple prime-edited hexaploid wheat plants, which expands the scope of prime editing and provides a powerful tool for stacking superior traits in plants, especially polyploid crop species.*

*Thus, we share the view of the two other reviewers, who noted that our work is “urgently required”, “very interesting”, and “worthy of affirmation” and would therefore be of great interest to the plant biotechnology community.*

Here are some minor concerns:

1. The testing of the four additional RNA structural motifs seems meaningless, because four or five consecutive Ts existed in the motifs, which may terminate the transcription of the U6 or U3 promoter.

*Response: Thank you for this comment. In this study, we tested a total of six different motifs joined to the 3' end of pegRNA to enhance its stability, including two previously reported modifications (Csy4 and tevopreQ1) and four additional RNA structural motifs (ENE, dENEs, U-A·U and Vc2), but only tevopreQ1 (epegRNA) enhanced the prime editing efficiency compared to that using pegRNA. Why the five other motifs decreased the editing efficiency is currently unclear. We agree with the reviewer that the presence of multiple T's in ENE, dENEs, and the U-A·U motif (but not Vc2) may terminate the transcription of the Pol III promoter, which could be one reason for the reduced editing efficiency. There are also other possible explanations. For example, these motifs may affect the secondary structure and stability of pegRNA, or perhaps the difference in pegRNA processing between wheat cells and animal cells (e.g., the presence of the Csy4 motif, which is more efficient in human cells) could help explain the reduced editing efficiency.*

*Therefore, we added the following explanation to the Discussion in the revised manuscript (L308-L313): “There are several possible reasons why the five other motifs decreased the editing efficiency. Perhaps these motifs affect the secondary structure and stability of pegRNA, or influence the transcription of pegRNA (e.g., the presence of four or five consecutive Ts in ENE, dENEs and U-A·U motifs might terminate the transcription of the Pol III promoter), or perhaps the differences in editing efficiency reflect a difference in pegRNA processing between wheat cells and animal cells (e.g., the Csy4 motif is more efficient in human cells).”*

2. In Fig2d, ePPEmax has two elements (NLS and Cas9max) different from the other vectors and cannot be regarded as a rigorous control. The ePPEmax* was not equal the combination of ePPEmax and ePPE*. The data of ePPEmax can be removed.

*Response: Sorry for the confusion. In Fig.2, we used ePPE as a control. Each engineered prime editor (ePPE*, ePPEmax and ePPEmax*) was optimized based on ePPE architecture and was thus compared with ePPE. PEmax with an R221K/N394K nSpCas9 (H840A) mutant and three NLSs was previously considered to be the most effective version for prime editing in human cells and rice cells (Chen et al., Cell, 2021; Li et al., Genome Biology, 2022; Jiang et al., Molecular Plant, 2022), so we updated ePPE to use the PEmax architecture, resulting in ePPEmax, to examine whether prime editing could be improved via a similar strategy in wheat cells. Therefore, we believe that the data for ePPEmax are important. On the one hand, such data can demonstrate whether this strategy works in wheat like in human and rice cells, and on the other hand, these data can be used as a reference to gauge whether our other engineered prime editors (e.g., ePPEmax*) are indeed better than ePPEmax.*

*Considering the superior performance of ePPE* (with one more NLS than ePPEmax) and Cas9max (with R221K/N394K), we suspect that introducing the R221K/N394K mutation of Cas9 into the ePPE* architecture may further improve editing efficiency. Therefore, ePPEmax* is not equal to the combination of ePPEmax and ePPE* but can be regarded as a combination of ePPE* with the R221K/N394K nSpCas9 (H840A) mutant .*

3. Fold change in Fig 1d, 2c, 2f, 3c was confused. Please explain the detail calculation in the method.

*Response: Sorry for the confusion. As there were large differences in editing efficiencies across different target sites, when we calculated the fold change, we normalized these efficiencies. If we did not normalize the data, the results in Fig.1d, 2c, 2f, and 3c (and several other similar plots) would be dominated by the target sites with higher editing efficiency. Therefore, we used the normalization approach to reduce this bias and obtain more general results.*

*Specifically, for the fold change calculations in Fig 1d, the average editing frequencies using pegRNA for each target were normalized to 1, and the frequencies using other pegRNA forms for each target were adjusted accordingly. For the fold change calculations in 2c, 2f, and 3c, the average editing frequencies using ePPE for each target were normalized to 1, and the frequencies using other prime editors for each target were adjusted accordingly. The editing efficiencies of all three repeats are presented in related figures. We added descriptions to the Methods (L416-425) and related figure legends (L641-643 for Fig.1d, L652-654 for Fig.2c, L659-660 for Fig.2f, L669-671 for Fig.3c).*

4. Line 191-193, the "up to 109.3 fold, 1399.7-fold" can not be found in FigS6c and S6d, please explain the calculation method.

*Response: We added two figures (revised Supplementary S7c and S7e) corresponding to the "up to 109.3-fold” and “1399.7-fold" change, respectively. We also added the method used for calculation to the figure legend: "The average editing frequencies using CMPE-PPE for each target were normalized to 1, and the frequencies using CMPE-ePPE and CMPE-ePPEplus prime editors for each target were adjusted accordingly.”*

Reviewer #2:

This study systematically optimizes various aspect of prime editing in wheat. The authors identify several optimizations and demonstrate the potential for using these for multiplexing in wheat protoplasts and plants. All optimizations/mutants were previously known, but they are the first to put them all together and show an improvement in prime editing activity. Such studies are urgently required as prime editing has relatively low efficiency in plants and a lot of plant researchers want to be able to use the technology. These results will be very interesting to the plant biotechnology community for sure. I am less certain for non-plant fields, but this is not my area.

*Response: We thank the reviewer for the positive comments on our work.*

Overall I find the experiments fairly well designed and executed. The results are clear and well defined. I do have a number of textual and experimental requests that should be addressed for it to be suitable for publication. Below, please find my specific comments. They are in order line by line, not by importance.

L32: what does it mean to downregulate genes with CRISPR? As in CRISPR interference? I also don't understand the emphasis at the start of the sentence "even for common wheat". Wheat was one of the first plant species reported to be CRISPR edited back in 2013 (one of the first three papers, Shan et al., 2013). This reference should be used instead of reviews.

*Response: We agree with this comment. To express more clearly and accurately, we changed this sentence to “Some reports describe the use of CRISPR-Cas9 and base editing systems for gene editing to improve various agronomic traits in common wheat [6-16]. However, CRISPR-Cas9 generates double-strand breaks (DSBs) in DNA that disrupt genes by inducing mixtures of random insertions and deletions (indels) at target sites. Base editing can efficiently install C•G-to-T•A and A•T-to-G•C transitions without requiring DSBs but usually induces bystander mutations when more than one C or A is present in the deamination window, and importantly, base editing cannot currently generate transversion mutations in the wheat genome. Therefore, more powerful, precise genome editing tools are urgently needed for functional genomics and the genetic improvement of common wheat.” We added the description in L31-39 and now cite the first report of CRISPR editing in wheat (Shan et al., Nature Biotechnology, 2013) as reference 8 in the revised manuscript.*

L35-37: "However, these studies are limited in that they mainly produce a single mutation type (e.g., gene knockout), yield imprecise mutations (usually random small insertions or deletions) and can edit only a limited number of genes at a time (generally just one)." Gene knockout via NHEJ is the same thing as imprecise mutations. References 8-10 all have multiplexing in their titles, therefore "generally just one" is incorrect.

*Response: Thank you for this comment. We changed the text to L31-39 in the revised manuscript “Some reports describe the use of CRISPR-Cas9 and base editing systems for gene editing to improve various agronomic traits in common wheat [6-16]. However, CRISPR-Cas9 generates double-strand breaks (DSBs) in DNA that disrupt genes by inducing mixtures of random insertions and deletions (indels) at target sites. Base editing can efficiently install C•G-to-T•A and A•T-to-G•C transitions without requiring DSBs but usually induces bystander mutations when more than one C or A is present in the deamination window, and importantly, base editing cannot currently generate transversion mutations in the wheat genome. Therefore, more powerful, precise genome editing tools are urgently needed for functional genomics and the genetic improvement of common wheat”.*

L53-54: Prime editing is inefficient in all plant species, common wheat and polyploids are not special. For this comment to stay, please provide a reference to experimental evidence that prime editing is more difficult in wheat/polyploids. I know a lot of people like to say it, but I do not think there is much (good) data to back this up.

*Response: Thank you for bringing up this point. Wheat is an allohexaploid species with a complex genome comprising three subgenomes. This feature and its high repetitive DNA content make it relatively difficult to manipulate using genome editing technology compared with diploid plants (Awan et al., Biotechnology Advances, 2022; Li et al., Plant Communications, 2021). Specifically, for diploid plants (e.g., rice), only two alleles must be edited for one gene, but for polyploids (e.g., common wheat), six alleles must be edited simultaneously since a single gene is present in three copies, which undoubtedly increases the difficulty of editing. Thus, much more highly efficient genome editing tools are needed for polyploid vs. diploid plants. In other words, a less efficient genome editing tool that can edit diploid genomes might not be able to edit polyploid genomes due to the need to edit more copies of a gene. Therefore, more efficient editing tools can provide a much greater chance to edit more alleles simultaneously in polyploid plants.*

*Due to its low efficiency, prime editing in common wheat has only been reported in wheat protoplasts (Lin et al., Nature Biotechnology, 2020; Zong et al., Nature Biotechnology, 2022), with an editing efficiency of only up to 1.8% (Zong et al, Nature Biotechnology, 2022). This editing efficiency is much lower than that in rice protoplasts (up to 19.5%; average of ~4.0%) despite their similar protoplast transformation efficiencies (approximately 40%) (Zong et al, Nature Biotechnology, 2022). In addition, no stable, precisely edited wheat plants have been obtained using prime editing, whereas edited rice and maize plants have been obtained in many studies at frequencies of up to 77.08% (Li et al., Genome Biology, 2022; Jiang et al., Molecular Plant, 2022; Qiao et al., Journal of Integrative Plant Biology, 2022). Of course, the low efficiency of genetic transformation in wheat is likely another reason for the difficulty in obtaining prime-edited wheat plants. Nevertheless, current prime editing systems displayed poor editing efficiency in wheat, i.e., much lower than CRISPR/Cas9 and base editing system-mediated genome editing in this crop (Shan et al., Nature Biotechnology, 2013; Zong et al., Nature Biotechnology, 2017; Li et al., Genome Biology, 2018), highlighting the need for the improvement of prime editing in wheat.*

*In addition, Perroud et al. used a prime editing system in tetraploid potatoes, but with very low efficiency: “None of the 20 kanamycin-regenerated transgenic plants were mutated at the target loci (StALS1 and StALS2), and only one plant regenerated from the chlorsulfuron-containing medium harbored a desired mutation”. Based on these results, the authors emphasized that such systems must be improved and that “This need for improvement is especially true for vegetatively propagated and polyploid species where editing efficiency needs to be high in the first generation.”*

*Therefore, we think it is reasonable to say that prime editing is inefficient, especially for wheat or other polyploid plants. We cited the above reports (Lin et al., Nature Biotechnology, 2020; Zong et al., Nature Biotechnology, 2022; and Perroud et al., Plant Science, 2022 as reference 25, 29 and 36, respectively) in the revised manuscript.*

The crystal structure does not need to be included. All AA changes were previously identified.

*Response: We deleted the crystal structure and added a schematic representation of M-MLV RT without the RNase H domain structure showing the functionally important amino acids residues including F156 in the palm region, V223 in the plam region and F309 in the thumb region that we mutated in this study to the revised Fig.2a.*

L111: claim that the V223A increases processivity is not supported by the data. The data just show increased PE efficiency. How that is actually done is unclear. I think: to alanine probably plays an important role… would be more accurate.

*Response: We sincerely appreciate this comment. In this study, we focused on the applications of prime editing in wheat, and improving prime editing efficiency was our first priority. Our deep amplicon sequencing data showed that ePPE-V223A significantly enhanced the frequencies of prime editing (~2.8-fold higher) compared to ePPE in wheat protoplasts. However, as indicated by the reviewer, why V223A increased the prime editing efficiency is unclear, and the underlying mechanisms might be complex. According to previous studies, the position of V223 in the active site motif YVDD of M-MLV RT is important for the processivity and fidelity of reverse transcription (Oscorbin et al., Computational and Structural Biotechnology Journal, 2021). The V223A mutation has been demonstrated to provide lower kinetic parameters (Km) for dNTPs in vitro, thus enabling the RT to perform fast and efficient cDNA synthesis with higher processivity than the wild-type enzyme (Palikša, Protein Engineering Design & Selection, 2018). Based on this observation, we speculate that the V223A mutation in ePPE likely plays an important role in enhancing RT processivity during reverse transcription and thus improves prime editing. Of course, biochemical experiments are needed to define the mechanism more clearly in the future. Therefore, based on the reviewer’s suggestion, we changed the statement that “indicating that the conversion of codon 223 from valine to alanine specifically plays an important role in enhancing RT processivity and thus improving prime editing ” to “the conversion of codon 223 from valine to alanine probably plays an important role in enhancing RT processivity and thus improving prime editing” in L120-121 of the revised manuscript. We hope that the reviewer finds the revised description to be more accurate than the original text.*

L123: "Among these engineered ePPEs, ePPEmax* showed the highest editing efficiency, followed by ePPEmax and ePPE*, resulting in 3.1-fold, 3.0-fold and 2.6-fold higher activity than ePPE, respectively (Fig. 2e,f)." I am doubtful of this claim. They look more or less equivalent to me. Especially ePPEmax* and ePPEmax. Statistics are needed to support this claim.

*Response: Thank you for this comment. We compared these three editors via statistical analysis and found that there were no significant differences between ePPE* and ePPEmax, ePPE* and ePPEmax*, or ePPEmax and ePPEmax*. However, among these engineered ePPEs, ePPEmax* had slightly higher average editing efficiency (9.7%) than ePPEmax (9.1%) and ePPE* (7.6%). We added the results of this analysis and the data for overall editing efficiency to Additional file 1 Fig. S3e. In the manuscript, we changed this sentence to “At the seven target sites tested, all three optimized prime editors’ architectures outperformed ePPE, with approximately 2.6-3.1-fold higher activity in wheat protoplasts (Fig. 2e,f) and with no apparent change in the proportion of byproducts (Additional file 1: Fig. S3d). Among these engineered ePPEs, ePPEmax* offered modestly higher editing efficiency, with an average of 9.7% across all sites tested, compared to ePPEmax (average 9.1%) and ePPE* (average 7.6%) (Fig. 2e,f and Additional file 1: Fig. S3e).”*

L165: "pegRNAs or epegRNAs were co-transformed with ePPEplus into wheat protoplasts. Targeted amplicon sequencing demonstrated that epegRNA induces higher activity for multiplex genome editing, from 1.3-fold to 4.2-fold greater than with pegRNA regardless of the processing strategy used". I disagree with this interpretation. As stated in the next sentence, the csy4 treatment with the standard pegRNA appears to work quite well, possibly even the same as the epegRNA. A statistical test is needed. These results are very interesting. It is curious why the Csy4 tail did not help in the experiments at the start (Figure 1). Perhaps pol II vs III? A combination of csy4 and the evopreq1? Be sure to discuss.

*Response: Thank you for your comment and sorry for the confusion. As you suggested, we performed a statistical test between epegRNA and pegRNA for each strategy; the results are presented in the revised Additional File 1: Fig. S6a,b. For the fold change calculations and statistical analysis, the average editing efficiencies of three repeated experiments of pegRNA to 1 to each target induced by each processing strategy and then efficiencies of epegRNA were adjust accordingly. The results showed that epegRNA had significant higher efficiency than pegRNAfor each strategy, about 2.7-fold, 4.2-fold, 3.2-fold, and 1.3- fold for Pol III promoter-, tRNA-, ribozyme-, and Csy4-processing strategies, respectively. Notably, the Csy4 treatment had significantly lower efficiently with the standard pegRNA than with epegRNA, which is consistent with the results showing that the values for pegRNA-Csy4 were significantly lower than epegRNA in Fig.1d. Therefore, it's probably not curious that the csy4 tail does not help in Fig.1d. We have added the normalized data and significance tests in the revised Additional File 1: Fig.S6b.*

*We apologize for the confusion around our previous wording of “Regardless of whether pegRNA or epegRNA was used, the Csy4-based processing system and Pol III promoter-based processing system each exhibited comparable efficiency….”, we meant that when using pegRNA, the Csy4-based processing system has comparable efficiency to the Pol III promoter-based processing system, and when using epegRNA, the Csy4-based processing system also has comparable efficiency to the Pol III promoter-based processing system, and whatever, the Csy4-based processing system and the Pol III promoter-based processing system both had much higher efficiency than the tRNA system and the ribozyme system. In conjunction with the reviewer's next comment, we have changed this sentence to “Among these four epegRNA-processing strategies, the Csy4 expression system had slightly higher efficiency (averaging 13.8%) than the use of individual Pol III promoters system (average 12.7%), and both of them performed much better than the tRNA system (average 6.2%) and the ribozyme system (average 4.9%) (Fig. 4b,c). In addition, there were no obvious differences in the edit:by-product ratio among these systems (Fig. 4b and Additional file 1: Fig. S6c,d). Based on these results, we selected Csy4-mediated multiplex prime editing (CMPE) for further study”.*

178-179: I don't follow the problems about Pol III promoter cloning. I can understand insert size at a certain point, once there are ~6 or so, @500 bp per Pol III, that's 3 kb in a plasmid. But Golden Gate cloning is rather efficient. And cumbersome delivery? Of what, protoplasts or agro transformation? For protoplasts pools of plasmids can be used. For agro, unless the plasmid is already ~25kb, it won't be a problem. Plant transformation efficiency usually only goes down after ~30 kb or so. I would just remove this comment. The csy4 results speak for themselves, the efficiency is highest and efficient, so we go with that.

*Response: Thank you for the suggestion. Because there were no statistically significant differences in the overall frequency of mutagenesis between the Csy4 sample and the Pol III promoter sample across three replicates (Fig.4c), and we changed the sentence to “Among these four epegRNA-processing strategies, the Csy4 expression system had slightly higher efficiency (averaging 13.8%) than the use of individual Pol III promoters system (average 12.7%), and both of them performed much better than the tRNA system (average 6.2%) and the ribozyme system (average 4.9%) (Fig. 4b,c). In addition, there were no obvious differences in the edit:by-product ratio among these systems (Fig. 4b and Additional file 1: Fig. S6c,d). Based on these results, we selected Csy4-mediated multiplex prime editing (CMPE) for further study”.*

L185: The authors need to be careful on claims about multiplex genome editing when using protoplasts. As the results are based on a population of individual cells, it is not possible to determine if any of the cells contain all or at least >1 edits.

*Response: Thank you for the comment. To tone down our claim, we changed the text to “CMPE-ePPEplus showed outstanding performance, introducing desired edits with 10.3% (5.8–22.5%), 9.6% (2.4–24.9%), 7.4% (1.5–20.9%), 7.5% (1.5–20.9%), 8.0% (1.5–23.3%) and 8.0% (0.7–21.9%) efficiencies at all four, five, six, eight, nine and ten target genes, respectively.”*

Figure 5: I think the T0 data would be better represented as a scattered bar plot as done for all figures preceding it, with mutation frequency on the y-axis and individual plants as dots. Then the readers can appreciate the level of mutagenesis in the plants. This also clarifies how the authors define het and homozygous (it looks like it is done by eye, not very accurate and subject to Sanger quality). Thresholds can be drawn on the graph. It is unfortunate the authors decided to use Sanger sequencing for this part of the analysis. It is much harder to get accurate quantifications than with NGS, but tools like ICE or TIDE should work.

*Response: We apologize for the confusion. We described how we genotyped the transgenic wheat plants in the original manuscript (L212 and L430-442 in the revised manuscript). Specifically, to detect the mutations in prime-edited wheat plants, we performed deep amplicon sequencing with universal primers and/or specific primers spanning the target sites. We characterized the edited wheat plants into five genotypes, including homozygous, heterozygous, chimeric, byproducts and wild type, following these criteria (Li et al., Genome Biology, 2022; Pan et al., Nature Plants, 2022): homozygous, mutation frequency ≥ 70% without undesired edits; heterozygous, mutation frequency ≥ 30% and < 70% without undesired edits; chimeric, mutation frequency ≥ 5% and< 30% according previous studies without undesired edits; byproducts, i.e., when the main mutation type in a homozygous/heterozygous/chimeric line contained undesired edits; and wild type, mutation frequency < 5%. We then performed Sanger sequencing using primers specific for subgenome A, B, or D to confirm the genotype at each target site and analyzed the chromatograms using SnapGene software.*

*While tools like ICE or TIDE are often used, we found that these two tools work for knockout but not point mutations. Moreover, based on the reviewer’s suggestion, we represented our T0 data as a scatter bar plot with mutation frequency on the y-axis and individual plants represented by dots for each targeted gene, with thresholds drawn on the graph, in the revised Additional file 1: Fig. S8.*

L212: I'm not sure why the subgenome analysis is only done in the plant work? Why was it omitted from the protoplast work?

*Response: Thank you for this comment. Transient protoplast transformation is a widely used assay for evaluating genome editing efficiency and assessing the frequency of generating editing by-products in plants, such as rice, Arabidopsis thaliana, wheat and so on. This system is more convenient and less time-consuming than plant transformation (which takes 3-4 months in wheat). Protoplasts are a good, scalable system to rapidly evaluate multiple mutagenesis parameters such as the efficiency, specificity, and precision of various genome editors, generating reliable and accurate results (Shan et al., Nature Biotechnology, 2013; Zong et al., Nature Biotechnology, 2018; Li et al., Genome Biology, 2018; Lin al., Nature Biotechnology, 2020; Zong et al., Nature Biotechnology, 2022). Since the results using protoplasts are based a population of individual cells, it is not possible to determine if any of the cells contain all or at least >1 subgenome edit, in wheat as the reviewer said, so overall mutation frequencies are normally evaluated. Furthermore, in theory, the trends and results are consistent regardless of whether the overall efficiency or the efficiency of separate subgenomes is analyzed. Therefore, it would be difficult and not meaningful to analyze the editing efficiencies of specific subgenomes for polyploid species.*

*In this study, we used protoplasts to evaluate the overall mutation efficiencies of various versions of pegRNAs or prime editors that we modified to identify the most efficient tools for editing the wheat genome, thus determining which prime editor would be best for the transformation of wheat plants. For stably transformed wheat plants, it is necessary to identify which subgenome has been edited for an individual plant, and thus can help us to comprehensively evaluate the editing properties of prime editors in wheat. Collectively, as described above, subgenome analysis is only done when analyzing stable transformed plants and is not performed when using transient protoplasts.*

To demonstrate the utility of the tool for plant improvement/functional genomics, the authors need to demonstrate transmission of the edits in next generation. Transgene free, null segregants are required for this analysis. This is standard in the field.

*Response: Thank you for the suggestion. We demonstrated transmission of the edits and analyzed the transgene-free and null segregants in the T1 generation. We added these data to Additional File 2: Table S6 and added a detailed description to L276-299 in the revised manuscript as follows:*

*“To investigate whether the mutations could be transmitted to the next generation, we self-fertilized T0-1 (with mutations in three genes), T0-13 (with mutations in all eight genes), T0-20 and T0-29 (with mutations in five genes), T0-43 and T0-46 (with mutations in four genes) plants. We screened 15 to 46 T1 seedlings from each T0 parent for mutations in the respective genes by PCR and DNA sequencing. For homozygous mutations, the transmission rates were 100%; for the majority of heterozygous mutants, Mendelian segregation occurred; for chimeric mutations in the T0 plants, the transmission rates ranged from 0% to <63.2%. For example, in plant T0-1, mutations in TaDME-D, TaGASR7-B, and TaGASR7-D that were homozygous in the T0 plant were present in all T1 progenies; mutations in TaALS-T2-B, TaDME-A, TaDME-B and TaGASR7-A that were heterozygous in T0 plants segregated at a 1:2:1 ratio in T1 progeny. By contrast, mutation in TaALS-T2-D that was chimeric in T0 plants resulted in only two plants in the T1 generation harboring chimeric mutations (Additional File 2: Table S6). Notably, some new mutations were detected in the T1 plants for some targets whereas the T0 plants were wild type (e.g., TaACC-T2-B site of the T0-20 line) (Additional File 2: Table S6), suggesting that the prime editors remained active in T0 and/or T1 plants. Furthermore, to examine the possibility of achieving targeted modifications without incorporating foreign DNA into the common wheat genome, we identified these T1 progenies with four primer sets specific for pB-CMPE-ePPEplus, and found that the frequencies of mutants without detectable transgenes were 4.2% (T0-1), 15.8% (T0-13), 21.0% (T0-20), 0.0% (T0-29), 6.7% (T0-43), and 4.3% (T0-46) (Additional File 2: Table S6). Collectively, these results demonstrate the feasibility of using the Csy4-mediated multiplex prime editing system to effectively generate heritable mutations in multiple genes, and a transgene-free plant carrying only the desired DNA sequence change can be obtained through genetic segregation.*

*In addition, we also added the description in the line of following text to the revised Discussion: “In addition, segregation analysis of six T0 lines indicated that the precisely edited genes could be inherited by the following subsequent generation and transgene-free prime-edited mutants could be obtained.”*

L272: replace "diploid plants" with "rice".

*Response: We replaced "diploid plants" with "rice", as suggested.*

L333: I would like to see the full plasmid sequences used (for the key variants and binary plasmids) rather than just the components. That is the only way to ensure reproducibility. Ideally .gb files would be included. Reduce the line spacing so the file is not huge. Or submit at a repository.

*Response: We added the full plasmid sequences used for the key variants and binary plasmids to the revised Additional file 3: Sequences 3, including ePPE-V223A, ePPEmax*, ePPEplus, CMPE-ePPEplus, pUC57-CmYLCV and pB-CMPE-ePPEplus. We also submitted these plasmids to a public repository, which will be available at Addgene.*

L346: I do not know how the wheat protoplast transfections were performed. The reference 52 is a review article. Most important, are the transfections normalized for transfection efficiency? Many of the differences reported may be due to transfection efficiency.

*Response: Sorry for the mistake; we replaced the old reference with the correct one (Wang et al., Nature Biotechnology, 2014) as reference 62 in the revised manuscript. In our study, the transfections were not normalized for transfection efficiency as in other papers (Shan et al., Nature Biotechnology, 2013; Zong et al., Nature Biotechnology, 2018; Li et al., Genome Biology, 2018; Lin al., Nature Biotechnology, 2020; Zong et al., Nature Biotechnology, 2022).*

*Specifically, we used a polyethylene glycol (PEG)-mediated method to transformation wheat protoplasts. For each transformation event, we used GFP as a control to evaluate the approximate transformation efficiency via microscopy. The efficiency of wheat protoplast transformation in our study was relatively stable, i.e., an average of ~40% (data not shown). To measure PE efficiency, the pegRNA vectors and PE vectors were simultaneously transformed into protoplasts (~5 µg per plasmid) as the experimental group using 200 µL protoplasts (~5106 cells) in 250 µL PEG for each transformation; we repeated this treatment three times (three biological replicates). It is worth mentioning that when testing the performance of different pegRNA forms or different engineered prime editors on each target, the different treatments were performed side-by-side and in parallel during protoplast transfection. Therefore, in theory, the possibility that the different editing frequencies reported in this study were due to different transfection efficiencies is very small or even negligible and does not influence our conclusions.*

Reviewer #3:

The manuscript entitled "Efficient and versatile multiplex prime editing in hexaploid wheat" (GBIO-D-23-00035) submitted by Ni et al., tested a series of reassembled prime editors by engineering both the pegRNA and the protein components of PE and demonstrated multiple prime editing activity in common wheat. Because PE strategy can perform precise editing without DNA double-strand break and donor DNA templates, it has attracted extensive attention of researchers. A lot of effort has been put into PE improvement, but so far, the overall efficiency of PE is still low and site-dependent, especially in plants. In this manuscript, the authors provided diverse data to demonstrate that reassembled PE vectors can created multi-site prime editing mutants in wheat, a polyploid plant. In general, although the paper does not show obvious innovations in the construction of PE vectors and application strategies, the result that the authors can obtain stable multi-edited wheat mutants based on their CMPE-ePPEplus system is still worthy of affirmation. Together, I think this manuscript could be considered for publication in Genome Biology with appropriate and necessary revisions.

*Response: We thank the reviewer for the positive comments on our work.*

Here are some comments:

1- The author needs to describe more about why they chose V223A mutation for the M-MLV reverse transcriptase. Although they wrote "Previous studies have reported that mutations at positions F156, V223 and F309 in the M-MLV RT can enhance the processivity and fidelity of reverse transcription" in line 99-100 and listed the reference, I still think this part needs more information. How to make the mutation? Why V223A not V223X (X refers other amino acids)? So does the F156 and F309 sites. I think these key data should be supplemented as much as possible. It will be more useful to the readers if more information added.

*Response: Thank you for your comments. We provided more information about the design of the editing system in the revised manuscript. We added a more thorough explanation of why we chose the F156W, V223A, V223H, V223I, F309N and V223H-F309N mutations for M-MLV reverse transcriptase to L98-111 to the revised manuscript as follows: “Previous studies have shown that several mutations in position F156 in the palm region of M-MLV RT, position V223 of the highly conserved YVDD motif in the palm region, and position F309N in the thumb region of M-MLV RT are important for the processivity and fidelity of RT [42-46] (Fig. 2a). Specifically, F156W could increase the fidelity of RT and might stabilize the interaction of Q190 residue with dNTP substrate, thus facilitating reverse transcription [43]; the V223A mutation has been demonstrated to enable the RT to perform faster and more efficient cDNA synthesis and higher processivity than the wild-type enzyme [44]; the V223H mutant increases the fidelity of RT and makes this enzyme more accurate [45]; the enzyme with V223I mutation increased polymerase activity compared to wild type in the extension assay using specific template-primers [46]; RT containing F309N and V223H/F309N was less likely to incorporate incorrect nucleotides and thus had higher fidelity than the wild type [45]. We therefore introduced these five single and one double amino-acid substitutions to the corresponding positions, resulting in six new prime editors: ePPE-F156W, ePPE-V223A, ePPE-V223H, ePPE-V223I, ePPE-F309N and ePPE-V223H-F309N”. We hope that these descriptions are more informative to readers.*

2- I noticed the F309N also showed an enhanced frequency, why the author did not try F309N+V223A? A corresponding explanation or discussion should be given for the choice of this design.

*Response: Sorry for the confusion. We compared ePPE-F309N and ePPE editors via statistical analysis, and found that ePPE-F309N had significant lower efficiency than ePPE across three replicates. So logically, there is no need to combine the F309N and V223A mutations with ePPE. We added the results of this analysis to Fig.2c and added related description to the main text (L116-118): “However, the five other point-mutant prime editors displayed lower or nearly no activity compared to ePPE (Fig. 2b,c). Therefore, these five amino-acid substitutions were not considered in further analysis”.*

3- The ePPEplus brought an increased byproducts compared with other PE editors, did you analyze the mutation type? What kind of mutation increase caused by M-MLV mutation? These data should also be supplemented, if experimental conditions permit.

*Response: Thank you for the suggestions. In this study, we used the previous method to calculate the percentages of by-products using 10-15 bp upstream and downstream of RT sequences as reference. We have reviewed the reads of by-products induced by PPE, ePPE, ePPE-V223A, ePPEmax*, and ePPEplus in detail and found that the mutation type and distribution of by-products induced by these prime editors were various and complex and showed no obvious regularity at first glance. In order to find patterns more clearly, we divided these by-products into three types: i) pegRNA scaffold-derived byproducts, which acted as extended RT templates and thus can introduce unwanted edits. Surprisingly, we observed low frequency of pegRNA scaffold mutation type of by-products only in TaBCH2, TaLOX2, TaSINA, and TaWTK3 targets, suggesting that pegRNA scaffold mutations are infrequently induced by prime editors in wheat cells. ii) RT template-related byproducts, including RT template duplication, insertion, deletion, or replacement occurring at RT template position. All 12 tested sites had these mutation by-products. We speculate that these mutations may occur because the newly synthesized strand competes with the original strand, leading to faulty repairs, or because the wrong nucleotides are introduced during reverse transcription, which are finally manifested as duplication, replacement, insertion, or deletion on the area of RT templates. iii) Other byproducts, usually harboring random SNPs included in the calculation, occurred far from the flanking regions outside spacer and RTPBS, generally positioned at 2-15 bp upstream and downstream of RT template. These types were probably introduced by amplification and deep sequencing because of the complexity and redundancy of the wheat genome, since we also found some of these mutations in the negative control sample.*

*Based on this analysis, we found that increased byproducts induced by ePPEplus at 2 of 12 target sites (TaSINA and TaWTK3) result from introducing pegRNA scaffold insertion, whereas PPE or ePPE had no corresponding mutation. And in most cases, increased values of byproducts induced by ePPEplus, ePPEmax*, or ePPE-V223A resulted from the increased percentages of these three mutation types of byproducts (e.g., TaBCH2 and TaLOX2) Nevertheless, although byproducts marginally increased at some tested targets, the ratio of edit:by-product at most tested sites was not significantly changed. We have added details on the byproducts of four represented targeted sites in the revised Additional file 1: Fig.S5 and added the description in L154-158 in the revised manuscript “Furthermore, although the exact values of byproducts including pegRNA scaffold-derived byproducts, RT template-related byproducts and some other random undesired mutations marginally increased at some target sites, the overall ratio of edit:byproduct at most tested sites was greater or comparable to that when using ePPEplus as compared to other prime editors (Fig. 3b, Additional file 1: Fig. S4b,c and Additional file 1: Fig. S5)”.*

4- In multiplex PE part, the tRNA shown a limited efficiency. Please noted which tRNA used here. How this tRNA worked on Cas9 NHEJ in wheat protoplasts used the same vector structure without reverse transcriptase and H840.

*Response: We used 77 bp tRNA^Gly^ genes to process multiple pegRNAs based on the report by Xie et al. (reference 49); we added this information to the revised figure legend (line 680).*

*We compared the knockout efficiencies of the Pol III promoter-, tRNA-, and Csy4-based sgRNA processing systems in wheat protoplasts using the same vector structure without reverse transcriptase and H840A. The Csy4-processing system displayed comparable (average of 30.9%) or slightly higher efficiency compared to the tRNA system (average of 26.7%) across all four tested sites. Both of these two systems had increased average efficiency than the Pol III promoter strategy (average of 21.2%) (Fig. R1). Our results were consistent with previous studies, which showed that Csy4 and tRNA systems are more robust than the individual Pol III promoters for targeted mutagenesis in tomato and rice (Cermak et al., The Plant Cell, 2017; Tang et al., Plant Biotechnology Journal, 2019). Therefore, tRNA-mediated knockout is efficient in wheat protoplasts. However, for multiplex prime editing, the tRNA-processing system had much lower efficiency than the Csy4- and Pol III promoter-processing systems (Fig. 4c). Perhaps the use of the tRNA-processing system generated different results for Cas9- vs. prime editing-mediated multiplex editing due to the different structures of pegRNA vs. sgRNA or the different processes involved in gene knockout vs. prime editing.*

Fig. R1. Multiplex gene editing mediated by Cas9 in wheat protoplasts.

a. Schematic diagrams of the constructs used for three distinct multiple esgRNA processing strategies. b. Comparison of the three multiplex editing systems across four genes. c Overall mutation frequencies mediated by the Pol III promoter-, tRNA- and Csy4-processing systems. P-values were obtained using the two-tailed Student’s t-test: *P < 0.05.

5- The multi-site editing strategy of the Csy4 and its efficiency comparison with tRNA and ribozyme elements have been clearly compared (Tang et al., 2019; DOI: 10.1111/pbi.13068). Based on a fuller and more accurate research background introduction, the author should cite and discuss the research work of Tang et al. (2019) in the paper.

*Response: Thank you for the suggestion. We cited this work as reference 53 and discussed it in L331-337 of the revised manuscript.*

*“Many convenient, efficient multiplexed sgRNA systems for CRISPR-Cas9 have been developed in plants involving the use of several Pol III promoters (U3 and U6) to express multiple sgRNAs in a single construct, the production of numerous sgRNAs via the endogenous tRNA-processing system, and the use of self-cleaving ribozyme and Csy-type ribonuclease 4 (Csy4)-processing systems. Comparisons of these four strategies in plant cells revealed that the Csy4 and tRNA systems showed more robust genome editing efficiencies than the self-cleaving ribozyme and conventional mixed dual promoter systems. In the current study, in order to leverage the versatility of prime editing to achieve multiplex genome editing in wheat, after evaluating these four strategies, we developed an efficient Csy4-mediated multiplex prime editing platform (CMPE) with ePPEplus in wheat”.*

**Second round of review**

**Reviewer 1**

I accept the urgent requirement and the practical application of the research in prime editing genes in hexaploid wheat plants, but the essential innovation of the components is still with limited innovation. Nevertheless, the author added clear description on all of my questions and did their best to improve their manuscript.

After read the related information carefully, I think the authors have addressed the concerns from reviewer 3.

**Reviewer 2**

L37: “base editing cannot currently generate transversion mutations in the wheat genome”. This is incorrect. Cytosine base editors readily make C-to-G transitions in plants. I’m not sure if it has been demonstrated in wheat, but I see no reason why it would not.

L55: References 25, 29 and 36 were included to argue that prime editing is especially low in wheat and polyploids in general. However, looking at the results in the two wheat papers (25 and 29): no direct comparison is made by the authors of those papers and no differences are tested. Further, the scale of editing in wheat overlaps with that observed in rice. An absence of data in the literature (prime editing in wheat) is not a solid foundation on which to build an argument. The results in reference 36 are from a single pegRNA; these results cannot be extrapolated to all polyploids. I maintain my original point, we do not have data that indicate genome editing reagents have lower efficiency is wheat and polyploids. Do not confuse editing efficiency in polyploids with multiplexing. In some cases one might only want to edit a single subgenomic region.

L120 (previously 111): The word ‘probably’ was added to address my comment, however I think this construction is not accurate. The data are clear, V223A is more efficient. Adding the word probably only diminishes the result. I think it would be most accurate for the authors to state:
“…indicating that the conversion of codon 223 from valine to alanine plays an important role in improving prime editing, probably due to enhanced RT processivity.”

I would have preferred seeing the scatter bar graph presented in the main figure. It would need to be compressed of course. It’s not clear to me where the data for the scatter come from. Are these NGS or Sanger? The text says both were used. It would help to specify in the legend which comes from what.

How was transgene presence/absence determined in the T1 plants? Need to include in the methods.

Just to note that it is a very risky assumption to make that all transfection efficiencies in an experiment are equal.

**Authors Response**

**Point-by-point responses to the reviewers’ comments:**

Reviewer #1: I accept the urgent requirement and the practical application of the research in prime editing genes in hexaploid wheat plants, but the essential innovation of the components is still with limited innovation. Nevertheless, the author added clear description on all of my questions and did their best to improve their manuscript.
After reading the related information carefully, I think the authors have addressed the concerns from reviewer 3.
*Response: We sincerely thank the referee for the kind encouragement.*

Reviewer #2:
L37: “base editing cannot currently generate transversion mutations in the wheat genome”. This is incorrect. Cytosine base editors readily make C-to-G transitions in plants. I’m not sure if it has been demonstrated in wheat, but I see no reason why it would not.
*Response: Thank you for this comment and sorry for mistake. Cytosine base editors readily make C-to-G transitions in plants including wheat (ref 15), so we changed the sentence to “Base editing can install C•G-to-T•A, A•T-to-G•C and C•G-to-G•C point mutations without requiring DSBs, but it usually induces bystander mutations when more than one C or A is present in the deamination window, and importantly, base editing cannot currently generate most transversions in the wheat genome” in the revised manuscript.*

L55: References 25, 29 and 36 were included to argue that prime editing is especially low in wheat and polyploids in general. However, looking at the results in the two wheat papers (25 and 29): no direct comparison is made by the authors of those papers and no differences are tested. Further, the scale of editing in wheat overlaps with that observed in rice. An absence of data in the literature (prime editing in wheat) is not a solid foundation on which to build an argument. The results in reference 36 are from a single pegRNA; these results cannot be extrapolated to all polyploids. I maintain my original point, we do not have data that indicate genome editing reagents have lower efficiency is wheat and polyploids. Do not confuse editing efficiency in polyploids with multiplexing. In some cases one might only want to edit a single subgenomic region.
*Response: Thank you for this comment and we apologize for our less rigorous statement. Although there are articles reporting the low prime editing efficiency in hexaploid wheat and tetraploid potato, as the reviewer said, these data are not enough and solid to support that the efficiency in polyploids is much less efficient than in diploid plants. So, our previous statement is not very rigorous and scientific. We changed the sentence in abstract “Prime editing is limited by low efficiency, especially in polyploids” to “Prime editing is limited by low efficiency in plants” and changed the sentence in the introduction “Nevertheless,* *the resulting engineered prime editors tend to exhibit highly variable efficiencies at different sites, poor capability for targeting multiple genes at the same time and poor efficacy in polyploid species, especially common wheat, suggesting that significant improvements will be needed to develop more efficient and universal plant prime editors, especially for polyploid plants.” to “Nevertheless, the resulting engineered prime editors tend to exhibit highly variable efficiencies at different sites and poor capability for targeting multiple genes at the same time. Furthermore, their applicability is primarily restricted to rice and maize. These limitations underscore the necessity for significant advancements to be made to develop more efficient and universal prime editors in plants, including hexaploid wheat”. We hope to answer the question of whether prime editing is more inefficient in polyploids than in diploids through direct comparison on large-scale targets in the future.*

L120 (previously 111): The word ‘probably’ was added to address my comment, however I think this construction is not accurate. The data are clear, V223A is more efficient. Adding the word probably only diminishes the result. I think it would be most accurate for the authors to state:
“…indicating that the conversion of codon 223 from valine to alanine plays an important role in improving prime editing, probably due to enhanced RT processivity.”
*Response: We sincerely appreciate this comment. We changed the construction to “…indicating that the conversion of codon 223 from valine to alanine plays an important role in improving prime editing, probably due to enhanced RT processivity” in the revised manuscript.*

I would have preferred seeing the scatter bar graph presented in the main figure. It would need to be compressed of course. It’s not clear to me where the data for the scatter come from. Are these NGS or Sanger? The text says both were used. It would help to specify in the legend which comes from what.
*Response: Thank you for this comment. We added the scatter bar graph in the Fig.5c in the revised manuscript, and we also specified the data were collected from NGS in the figure legend of Fig.5c and Additional File:1 Fig.S8.*

How was transgene presence/absence determined in the T1 plants? Need to include in the methods.
*Response: Thank you for this comment. We added the detail method of detecting of transgene presence/absence in the Method section in the revised manuscript.*

Just to note that it is a very risky assumption to make that all transfection efficiencies in an experiment are equal.
*Response: We agree the reviewer comment. It is very difficult or even impossible to guarantee the all transfection efficiencies in an experiment are equal. So in our study in order to reduce the impact of transfection efficiency difference in an experiment as much as possible, when testing the performance of different pegRNA forms or different engineered prime editors on each target, the different treatments were performed side-by-side and in parallel during protoplast transfection and repeated three times as in other papers in this field (Shan et al., Nature Biotechnology, 2013; Zong et al., Nature Biotechnology, 2018; Li et al., Genome Biology, 2018; Lin al., Nature Biotechnology, 2020; Tang et al., Molecular Plant, 2020; Zong et al., Nature Biotechnology, 2022; Jiang et al., Molecular Plant, 2022).*
